# Metal-Free Synthesis of Polysubstituted Triazoloquinoxalines Using Alkynols as the Key Building Blocks

**DOI:** 10.1021/acsomega.4c03979

**Published:** 2024-09-05

**Authors:** Berenika Masaryk, Miroslav Soural

**Affiliations:** Department of Organic Chemistry, Faculty of Science, Palacký University, 17. listopadu 12, 779 00 Olomouc, Czech Republic

## Abstract

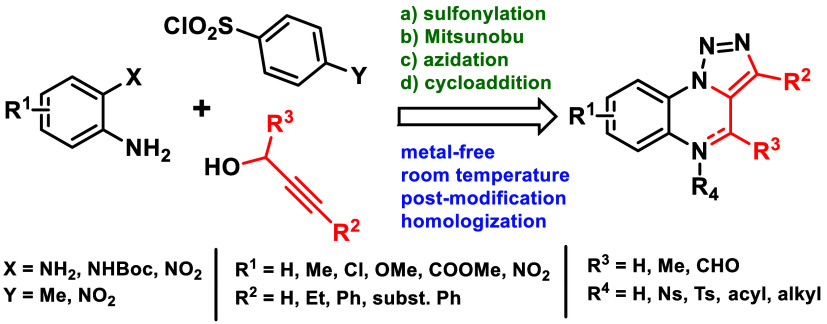

*o*-Phenylenediamines, *o*-nitroanilines, and Boc-*o*-phenylenediamines were converted to *N*-Ts/Ns-*o*-phenylenediamines, followed by Mitsunobu alkylation with prop-2-yn-1-ols. After one-pot azidation, the resulting intermediates underwent Huisgen cycloaddition, which yielded Ts/Ns-dihydrotriazoloquinoxalines. Cleavage of the arylsulfonyl moiety provided (dihydro)triazoloquinoxalines with the possibility of modifying the *N*^5^ position. The application of but-3-yn-1-ols and pent-4-yn-1-ols allowed the preparation of benzotriazolodiazepines and benzotriazolodiazocines. The developed protocols enable the preparation of diversely substituted products from readily available starting materials under mild and metal-free conditions.

## Introduction

In recent decades, the 1,2,3-triazole scaffold has been extensively used by medicinal chemists to modify the structures of biologically active compounds. In addition to being directly included in pharmacophores and/or their peripheral substitution to fine-tune the properties of drugs,^1,2^ conjugation *via* triazoles by bioorthogonal reactions between alkynes and azides has been widely utilized to study intracellular processes.^3,4^ An individual group of bioactive triazoles consists of derivatives bearing additional fused heterocyclic scaffolds. In this regard, [1,2,3]triazolo[1,5-*a*]quinoxalines (TQs) were reported as ligands of benzodiazepine and adenosine receptors^5,6^ and agonists of the G-protein-coupled niacin receptor.^7^ Consequently, various synthetic pathways leading to TQs have been investigated. Different approaches starting from aryl iodides have been developed, with Cu catalysis^8−12^ or Ru catalysis^13^ required to accomplish azidation and/or the cycloaddition step. Eventually, the combination of both Cu and Pd catalysis within the reaction sequence was reported.^14^ A general method based on Ugi multicomponent reaction and noncatalyzed Huisgen cyclization was recently introduced for 4-oxo derivatives, however, with a high temperature needed for the cycloaddition step.^15^ Preparation of one TQ derivative using a metal-free and azide-free protocol was also described.^16,17^

In our ongoing research, we focused on synthetic pathways leading to fused triazoles using alkynols for the installation of alkyne moieties. Recently, we developed Huisgen cycloadditions for triazolodiazepines^18^ and triazolodiazepinones^19^ starting from Ns-(homo)azidoalanine. In this article, the application of a noncatalyzed Huisgen cycloaddition to yield TQs from Ns/Ts-phenylenediamines is reported.

## Results and Discussion

**Scheme 1:**
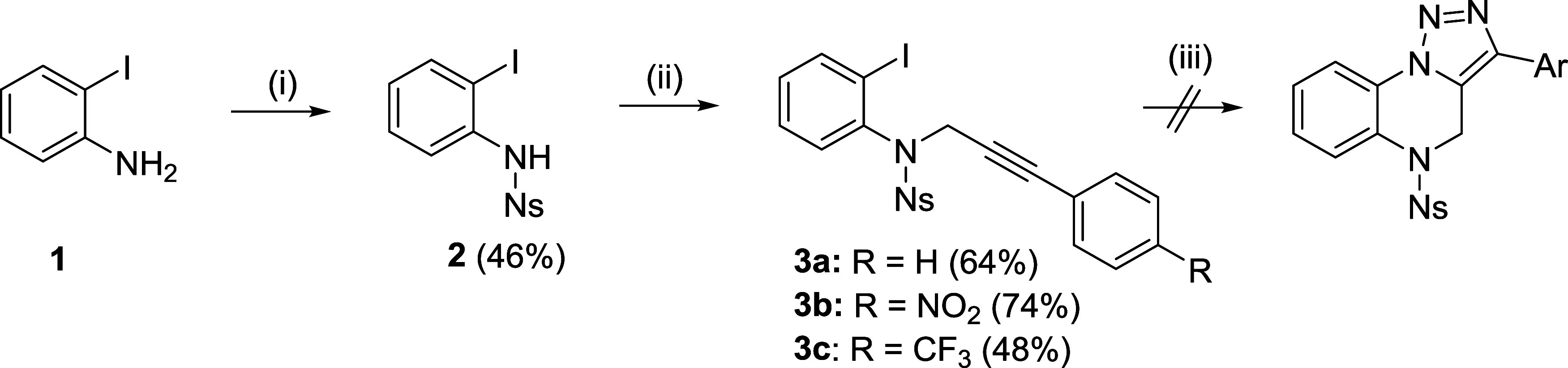
Attempts to Prepare TQs from Alkynol and Iodoaniline Using Cu Catalysis Reagents and conditions: (i) 4-nitrobenzenesulfonyl chloride, dichloromethane (DCM), pyridine, rt, 2 h; (ii) alkynol, triphenylphosphine (TPP), diisopropylazadicarboxylate (DIAD), anh. THF, rt, 2 h; and (iii) various conditions adopted from the literature.^9^

Although primarily targeting a metal-free pathway, we initially tested a previously reported approach^9^ in which copper catalysis was used for the conversion of *N*-acyl-2-iodoanilines to TQs. To mimic this strategy for *N*-sulfonyl analogs, 2-iodoaniline was reacted with 4-nitrobenzenesulfonyl chloride (4-NsCl), and the resulting Ns-2-iodoaniline **2** was alkylated with 3-phenylprop-2-yn-1-ol using the Mitsunobu procedure (Scheme 1). Intermediate **3a** was subjected to Cu-catalyzed cycloaddition with sodium azide under previously reported conditions;^9^ however, the formation of TQs (or the corresponding triazoles) was not observed, and only decomposition to a mixture of unknown compounds occurred. Intermediates **3b** and **3c** bearing electron-withdrawing groups provided the same results.

**Scheme 2:**
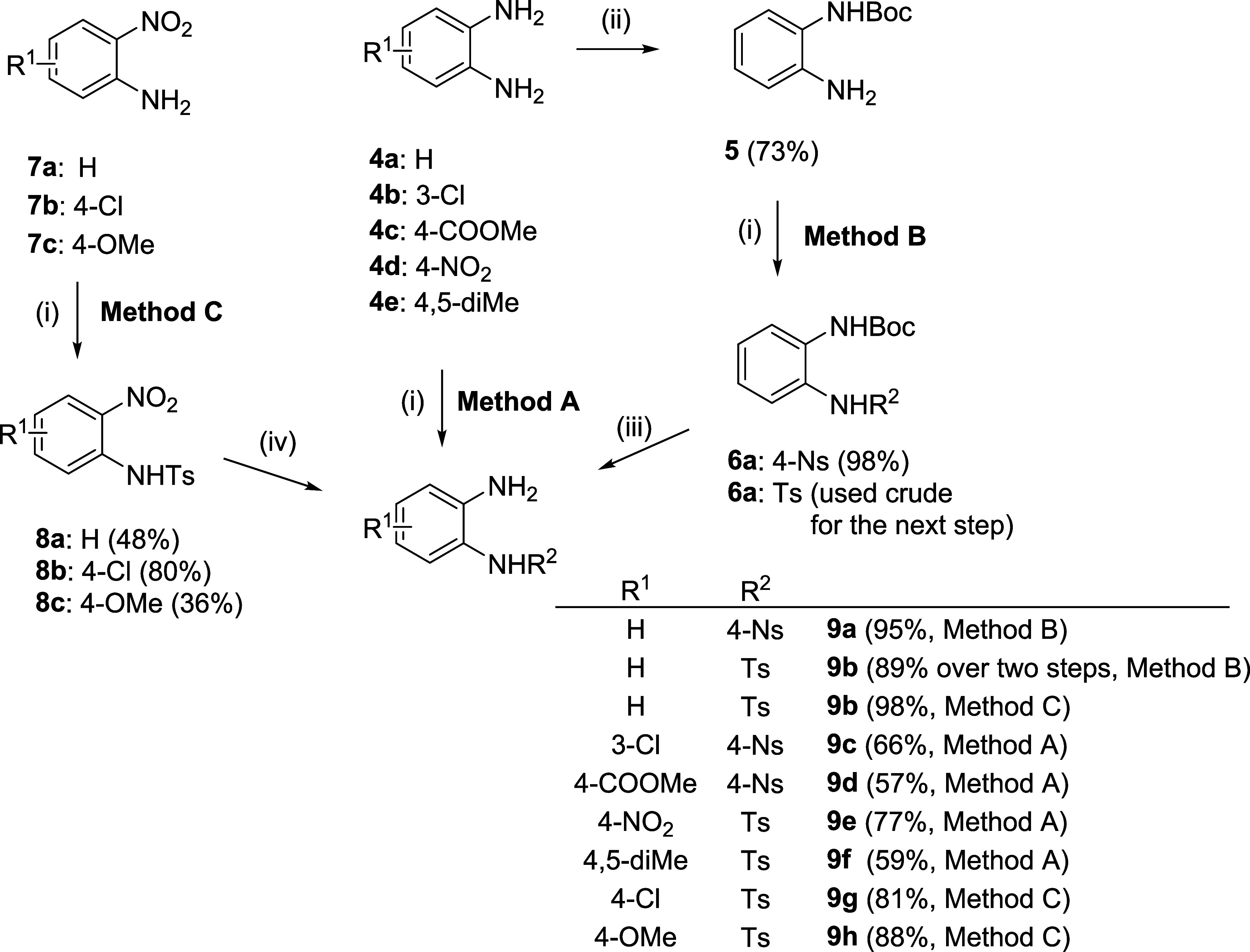
Alternative Starting Materials and Their Conversion to Sulfonamides Reagents and conditions: (i) 4-NsCl or TsCl, pyridine (DCM), rt; for individual reaction times, see the Experimental Section; (ii) Boc_2_O, DCM, 0 °C; (iii) 25% TFA/DCM, rt; and (iv) H_2_, Pd/C, EtOH, 2 or 16 h (for **8c**).

Consequently, we switched from 2-iodoaniline to *o*-phenylenediamines as the starting materials. Notably, *o*-azidoaniline was not considered a suitable alternative due to its problematic availability and due to interference of the azido group with triphenylphosphine during Mitsunobu alkylation. Direct sulfonylation (Method A, Scheme 2) of *o*-phenylenediamine **4a** with 4-NsCl resulted in limited regioselectivity and led to the formation of disulfonylated byproducts that were difficult to separate (yields < 40%). Alternatively, diamines substituted with suitable directing functional groups (3-Cl: **4b**, 4-COOMe: **4c**, 4-NO_2_: **4d**) furnished the desired sulfonamides in acceptable crude purities and isolated yields. Method A was also successfully applied to symmetrical diamine **4e**. For unsubstituted *o*-phenylenediamine and its derivatives lacking sufficient regioselectivity, we developed two alternative approaches. Method B was based on monoprotection of **4a** with di-*t*-butyl dicarbonate, followed by sulfonylation. Although this alternative requires two additional steps compared to Method A, its simplicity and almost quantitative yield make it the method of choice. Method C consisted of the use of nitroanilines as precursors of *o*-phenylenediamines. Although the reactivity was compromised and the sulfonylation of **7a** and **7b** was difficult to complete, the subsequent catalytic hydrogenation furnished desired sulfonamides **9b**, **9g**, and **9h** in high yields.

**Scheme 3:**
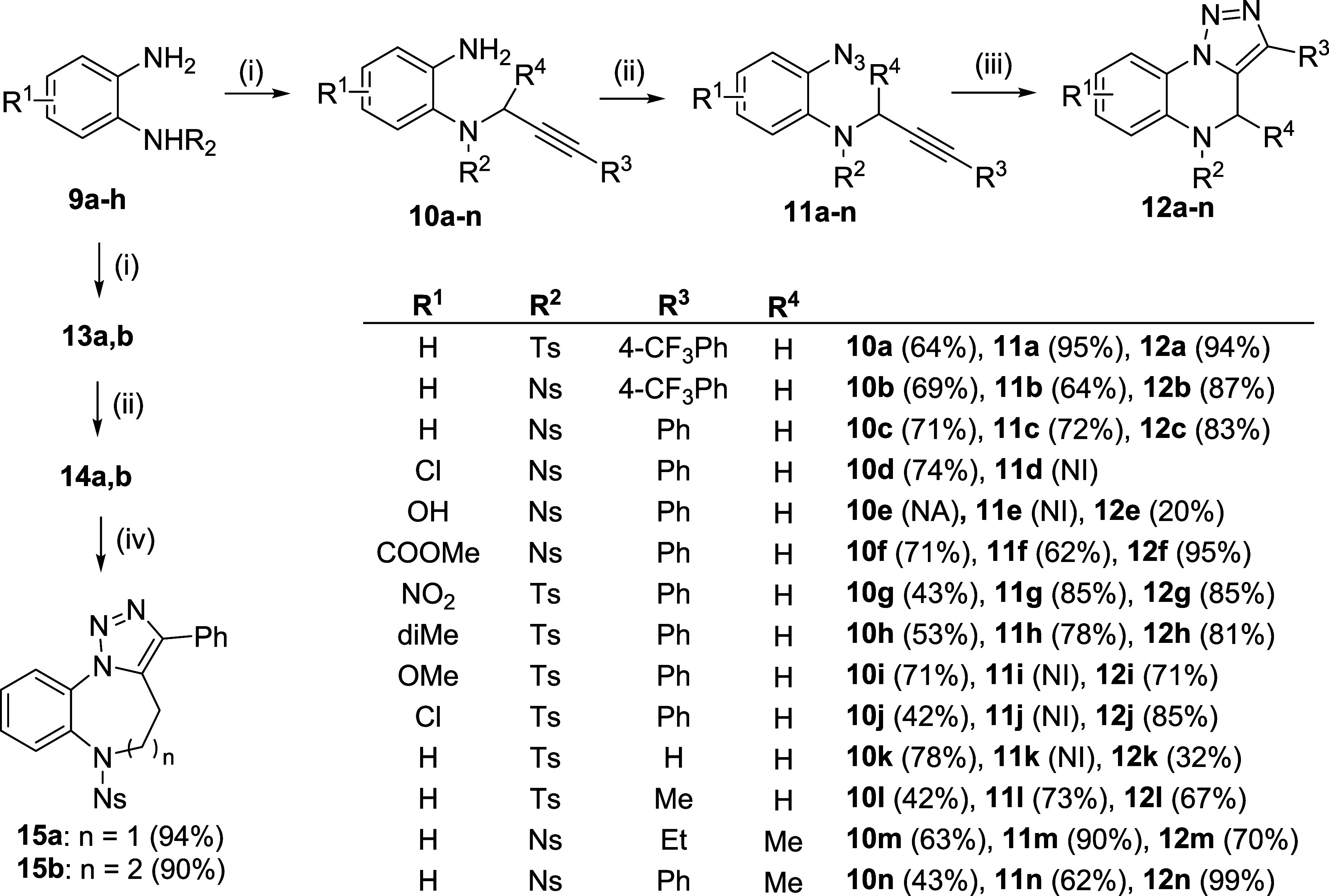
Synthesis of DihydroTQs and Homological Scaffolds Reagents and conditions: (i) alkynol, triphenylphosphine (TPP), diisopropylazadicarboxylate (DIAD), anh. THF, rt, 2 h; (ii) (a) HCl/ACN/H_2_O, NaNO_2_, 0 °C, 30 min; (b) NaN_3_, H_2_O, rt, 30 min; (iii) DMSO, 45 °C, 3–5 h; and (iv) DMSO, 90 °C, 3 h (**15a**) or 48 h (**15b**).

The obtained sulfonamides **9a–h** were reacted with different primary or secondary alkynols: propargyl alcohol, 3-phenylprop-2-yn-1-ol, 3-(4-(trifluoromethyl)phenyl)prop-2-yn-1-ol, but-2-yn-1-ol, 4-phenylbut-3-yn-2-ol, hex-3-yn-2-ol and 4-phenylbut-3-yn-1-ol (Scheme 3). The last two building blocks mentioned were included to test the possible preparation of larger cycles: benzotriazolodiazepines and benzotriazolodiazocines. Mitsunobu alkylation smoothly afforded the corresponding intermediate **10**. In some cases, the separation of diisopropylhydrazine-1,2-dicarboxylate (DIHD) was problematic, but traces of DIHD were easily and quantitatively removed after the next reaction step. The formation of azides was performed in one pot *via* the corresponding diazonium salts. Under developed and optimized conditions using 10% HCl/acetonitrile 2:3 (v/v) as the solvent, products **11** spontaneously precipitated from the reaction mixture and were isolated by simple filtration in excellent crude purity and high yields. Only in the cases of **11i–k**, the oily products were extracted.

**Scheme 4:**
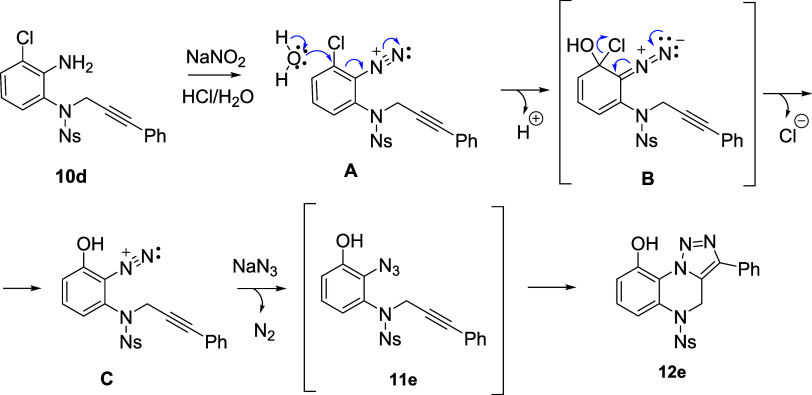
Spontaneous Formation of the Hydroxyl Derivative **12e** from **10d***via* Meisenheimer Complex **A**

An interesting result was obtained after azidation of intermediate **10d** (R^1^ = Cl): analysis of the isolated product revealed the formation of cyclic product **12e** bearing hydroxy instead of chloro substitution. This result indicated spontaneous quantitative cycloaddition and nucleophilic substitution *via* the formation of Meiseinheimer complex **B** in the diazonium salt stage (Scheme 4). This hypothesis was supported by the detection of hydroxydiazonium intermediate **C** with LC–MS analysis prior to reaction with sodium azide.

Not only intermediate **11e** but also all azides **11** were highly prone to cycloaddition: when standing in DMSO-*d*_*6*_ solution prior to NMR analysis, the corresponding Ts/Ns-TQs **12** were detected in the 1H spectra. For this reason, only selected intermediates were fully characterized. The completion to TQs at room temperature was observed after 48–72 h; however, the reaction time could be shortened to 3–5 h by heating of intermediates **11** in DMSO to 45 °C. The products were quantitatively formed without any impurities, precipitated by water, and isolated as single compounds. The formation of larger cycles was substantially more demanding and required heating to 90 °C for 3 h (benzotriazolodiazepine **15a**) or 48 h (benzotriazolodiazocine **15b**); however, no side products were detected.

**Scheme 5:**
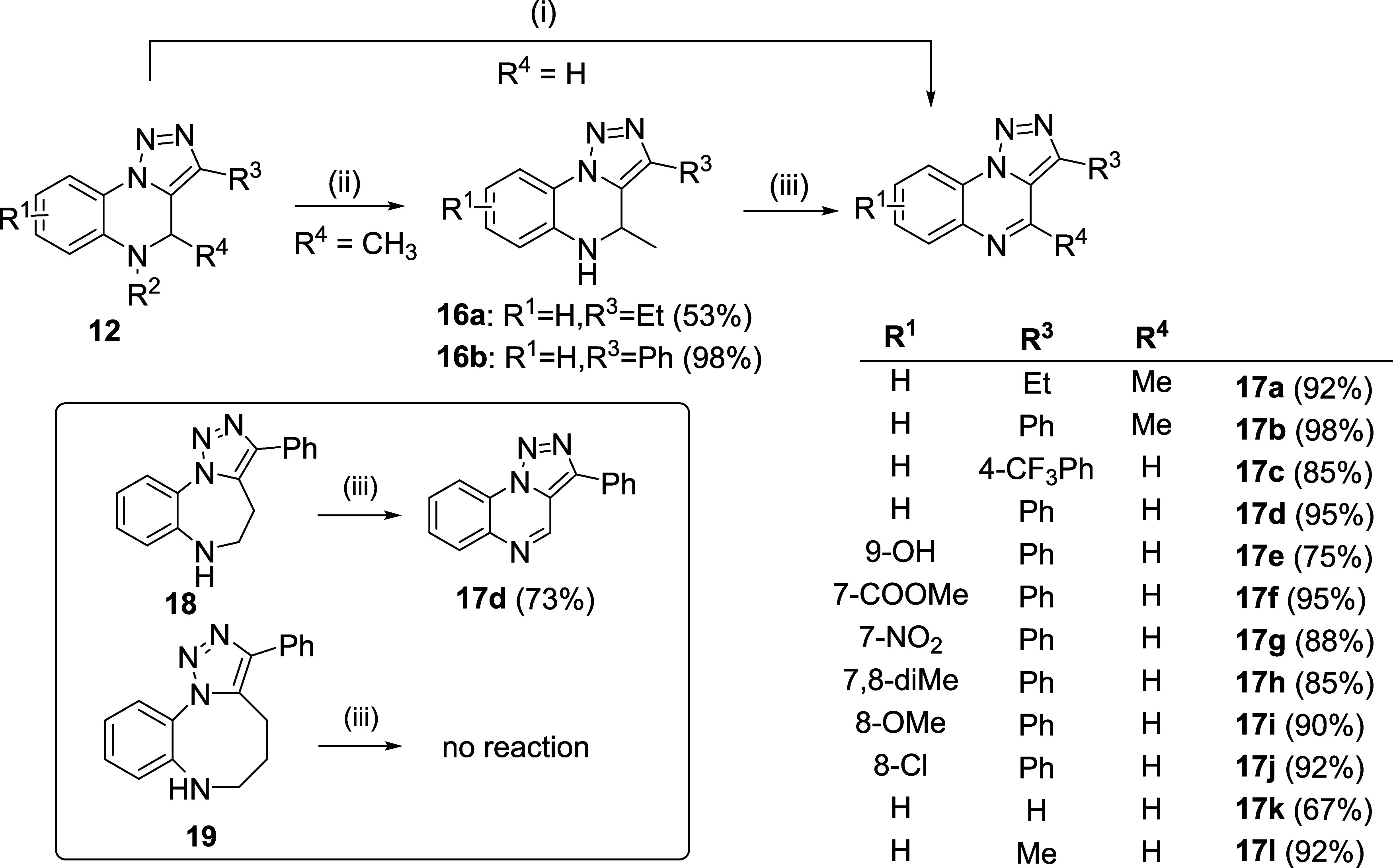
Desulfonylation and Oxidation of Ns/Ts-TQs to Fully Aromatic Analogs Reagents and conditions: (i) DBU, DMSO, rt or 90 °C, 3–15 h; (ii) mercaptoethanol, DBU, DMSO, rt, 30 min; and (iii) MnO_2_, toluene, 110 °C, 24 h (**17d** from **18**) or 80 °C, 24 h (**17a**,**b** from **16a**,**b**).

Next, we proceeded with the cleavage of the Ns/Ts group, leading to full aromatization of the scaffold by using 1,8-diazabicyclo[5.4.0]undec-7-ene (Scheme 5).

**Scheme 6:**
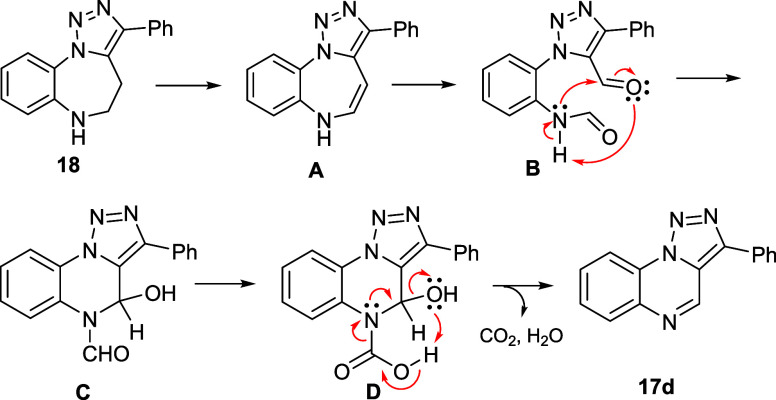
Hypothetical Mechanism for Oxidative Ring Contraction of **18**

Notably, the isolation of Ts/Ns-TQs prior to this step was not necessary because the direct cleavage of sulfonamides to produce **17** was feasible by the addition of DBU to the reaction mixture after Huisgen cycloaddition. This alternative shortened the reaction pathway and had a positive effect on the overall yield. The reaction conditions for desulfonylation strongly depended on the C–H acidity at the C^4^ position: unsubstituted derivatives were smoothly converted using DBU at room temperature (compounds with R^3^ = aryl) or at elevated temperature (compounds with R^3^ = alkyl), whereas derivatives **12m**,**n** (R^4^ = Me) and intermediates **15a,b** were resistant to this protocol. In such cases, the cleavage of Ns with thiolate was performed, followed by the aromatization of **16a**,**b** with MnO_2_. This alternative furnished products **17a**,**b**, whereas *N*-unsubstituted benzotriazolodiazocine **19** was completely resistant to oxidation. A surprising result was obtained from the oxidation of benzotriazolodiazepine **18**, which was converted to triazoloquinoxaline **17d**. Scheme 6 shows a plausible mechanism for this conversion. After oxidation to conjugated enamine **A**, this intermediate can undergo further oxidative attack, leading to the formation of *N*-formyl derivatives and aldehydes as previously reported for MnO_2_ oxidative cleavage of substituted anilines.^20^ Intermediate **B** can cyclize to **C** followed by further oxidation, decarboxylation, and dehydration (or *vice versa*) resulting in aromatic scaffold **17d**. The reason for unreactivity of compound **19** toward the analogical conversion may be its impossibility to form the fully conjugated enamine of type **A**. We also tested the reactivity of compound **15a** under identical conditions as for the oxidation of **18** (MnO_2_, toluene, 110 °C, 24 h) but compound **17d** was not obtained, and only a mixture of unknown products was detected. On the other hand, LCMS analysis of the reaction after 6 h indicated the partial conversion of **15a** to the oxidized analog (ESI+ 446, corresponds to Ns-derivative type A, see Supporting Information). Further heating led to decomposition; however, this observation partially supports the suggested mechanism.

**Scheme 7:**
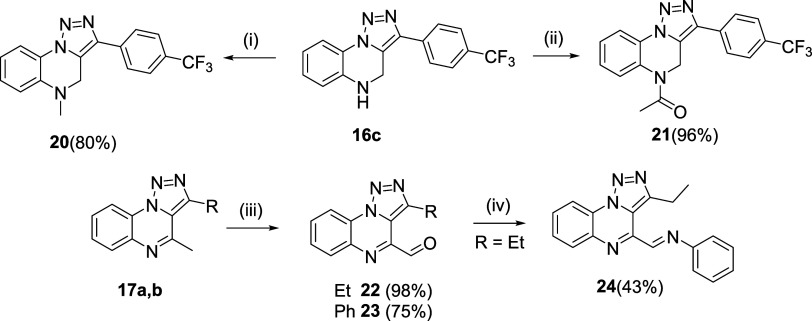
Further Modification of TQs at C^4^ and N^5^ Positions Reagents and conditions: (i) MeI, DBU, DMSO, 50 °C, 16 h; (ii) Ac_2_O, DBU, DMSO, 50 °C, 3 h; (iii) SeO_2_, THF, reflux, 3 h; and (iv) aniline, EtOH, 70 °C, 3 h.

In contrast to direct DBU cleavage, the removal of sulfonamides with thiolate resulted in the liberation of the secondary amine, which was used as a strategy for *N*^5^ modification of the TQ scaffold with various electrophiles. This possibility was demonstrated by the acetylation and methylation of compound **16c**, which was synthesized using conditions identical to those used for **16a** and **16b** (Scheme 7). Furthermore, C^4^-methyl derivatives **17a** and **17b** were smoothly converted to the corresponding aldehydes, which opens the door to a wide range of transformations with different nucleophiles.^21^ As an illustrative example, Schiff base **24** was synthesized by using aniline.

## Conclusions

We developed a simple synthetic strategy for accessing [1,2,3]triazolo[1,5-*a*]quinoxalines starting from various alkynols and *o*-phenylenediamines or 2-nitroanilines. In contrast to previous approaches, triazole formation is based on a noncatalyzed Huisgen cycloaddition, which can be accomplished at room temperature. In the case of higher alkynols, the preparation of benzotriazolodiazepines and benzotriazolodiazocines is possible, although under harsh conditions. Importantly, the synthesis starts from inexpensive and readily available building blocks, and the developed protocols can be used to variously modify the target scaffold at positions C^2^, C^4^, and N^5^ and the benzene ring. Consequently, the method can be utilized to synthesize diverse TQs in the search for compounds for different applications.

## Experimental Section

Solvents and chemicals were purchased from Sigma-Aldrich (Milwaukee, WI, www.sigmaaldrich.com), Acros Organic (Geel, Belgium, www.acros.com), and Fluorochem (Hadfield, United Kingdom, www.fluorochem.co.uk). Anhydrous solvents were dried over 4 Å molecular sieves or stored as received from commercial suppliers. Reactions were performed in round-bottom flasks fitted with rubber septa under positive pressure of nitrogen or in pressure ampules, unless stated otherwise.

Reactions were monitored by LCMS analysis or thin-layer chromatography (TLC) using aluminum plates precoated with silica gel (silica gel 60 F_254_, Merck, The United States) impregnated with a fluorescent indicator. TLC plates were visualized by exposure to ultraviolet light (λ = 254 nm) and/or by submersion in aqueous ceric ammonium molybdate (CAM) solution and/or potassium permanganate (KMnO_4_) and/or vanillin solution, followed by brief heating. The LCMS analyses were carried out on a UHPLC-MS system consisting of UHPLC chromatograph Acuity with a photodiode array detector and single quadrupole mass spectrometer (Waters), using X-Select C18 silica gel with the mobile phase consisting of 10 mM ammonium acetate (AmAc) in H_2_O and CH_3_CN. The ESI source operated at a discharge current of 5 μA, vaporizer temperature of 350 °C, and capillary temperature of 200 °C. For the LCMS analysis, samples were extracted into CH_3_CN/H_2_O (20% or 50%; 1 mL).

All 1D NMR experiments were performed using a Jeol ECA400II (400 MHz) and/or ECX500 spectrometer (JEOL RESONANCE, Tokyo, Japan) at magnetic field strengths of 9.39 and 11.75 T, corresponding to resonance frequencies of 400 MHz (for ^1^ H), 100.6 MHz (for ^13^C), 500.16 MHz (for ^1^H), and 125.77 MHz (for ^13^C) at 26–27 °C. Chemical shifts (δ) are reported in parts per million (ppm) and coupling constants (*J*) are reported in Hertz (Hz). The signals of CDCl_3_, DMSO-*d*_*6*_, and TFA*-d* signals were set at 7.26, 2.50, and 11.50 ppm in ^1^H NMR spectra and to 77.16, 39.52, and 164.2 ppm in ^13^C NMR spectra, respectively. Abbreviations in NMR spectra: brs, broad singlet; s, singlet; d, doublet; dd, doublet of doublets; ddd, doublet of doublets of doublets; dt, doublet of triplets; t, triplet; td, triplet of doublets; m, multiplet; p, pentet; q, quartet; qd, quartet of doublets; qt, quartet of triplets.

HRMS analysis was performed using LCMS (Dionex Ultimate 3000, Thermo Fischer Scientific, MA, USA) with an Exactive Plus Orbitrap high-resolution mass spectrometer (Thermo Exactive Plus, Thermo Fischer Scientific, MA, USA) operating at a positive or negative full scan mode (120 000 FWMH) in the range of 100–1000 *m*/*z* with electrospray ionization working at 150 °C and a source voltage of 3.6 kV. Chromatographic separation was performed on C18 silica gel (Phenomenex Gemini, 50 × 2 mm, 3 μm particle) with isocratic elution and mobile phase (MP) containing CH_3_CN/10 mM AmAc (80/20; v/v). The samples were dissolved in CH_3_CN or CH_3_CN/H_2_O (1/1; v/v).

### Synthetic Procedures

#### Synthesis of *N*-(2-Iodophenyl)-4-nitrobenzenesulfonamide **2**

2-Iodoaniline **1** (2.5 g, 11.4 mmol, 1 equiv), pyridine (1 mL, 12.5 mmol, 1.1 equiv), and 4-NsCl (2.78 g, 12.5 mmol, 1.1 equiv) were dissolved in DCM (20 mL) and the reaction mixture was stirred at room temperature for 1 h. The reaction mixture was then washed with 2 × 50 mL of 10% AcOH and the combined aqueous layers were washed with 2 × 150 mL of DCM. The combined organic layers were dried using MgSO_4_, filtered, and evaporated to dryness *in vacuo*. Crystallization of the crude product from DCM/EtOAc (20:1, v/v) afforded the pure product **2** (2.1 g, 46% yield) as a pale orange solid. ^1^H NMR (500 MHz, DMSO-*d*_6_) δ 10.26 (s, 1H), 8.44–8.38 (m, 2H), 7.98–7.91 (m, 2H), 7.85 (dd, *J* = 7.9, 1.4 Hz, 1H), 7.34 (ddd, *J* = 8.0, 7.4, 1.5 Hz, 1H), 7.07–7.00 (m, 2H). ^13^C NMR (126 MHz, DMSO-*d*_6_) δ 149.8, 146.3, 139.8, 137.5, 129.1, 129.1, 128.4, 128.4, 124.7, 99.2.

#### Synthesis of *tert*-Butyl(2-aminophenyl)carbamate **5a**

*o*-Phenylenediamine **4a** (1.08 g, 10 mmol, 1 equiv) was dissolved in 25 mL of DCM and the reaction flask was placed in an ice bath. Boc_2_O (2.08 g, 10 mmol, 1 equiv) was added in portion to the stirring solution over an interval of 2 h. After the last portion was added, the reaction mixture was removed from the ice bath and left at room temperature on a magnetic stirrer for an additional 16 h. The reaction mixture was evaporated to dryness *in vacuo*. The crude product was purified by silica gel chromatography using Hex:EtOAc (3:7, v/v) to give pure product **5a** (4.55 g, 73% yield) as a white solid. ^1^H NMR (400 MHz, CDCl_3_) δ 7.31–7.24 (m, 1H), 7.03–6.95 (m, 1H), 6.84–6.73 (m, 2H), 6.24 (bs, 1H), 3.62 (brs, 2H), 1.51 (s, 9H). ^13^C NMR (101 MHz, CDCl_3_) δ 154.0, 140.0, 126.3, 125.0, 119.8, 117.8, 91.0, 80.7, 28.5.

#### Synthesis of *tert*-Butyl(2-((4-nitrophenyl)sulfonamido)phenyl)carbamate **6a**

Compound **5a** (1.68 mg, 8.08 mmol, 1 equiv), 4-NsCl (1.883 g, 8.484 mmol, 1.05 equiv), and pyridine (682.5 μL, 8.484 mmol, 1.05 equiv) were dissolved in DCM (15 mL) and stirred at room temperature for 2 h. Later, the reaction mixture was evaporated to dryness *in vacuo*. The colorless oil was poured into 50 mL of water and vigorously shaken. The resulting white precipitate was filtered off under reduced pressure and washed several times with water. After being dried, pure product **6a** (3.12 g, 98% yield) was obtained as a white solid. ^1^H NMR (400 MHz, CDCl_3_) δ 8.40–8.09 (m, 2H), 7.99–7.65 (m, 3H), 7.26–7.07 (m, 4H), 6.48 (s, 1H), 1.51 (s, 9H). ^13^C NMR (101 MHz, CDCl_3_) δ 154.0, 150.3, 145.5, 133.5, 128.7, 128.6, 126.9, 125.8, 124.2, 123.3, 82.2, 28.4. HRMS (ESI neg.) *m*/*z*: [M + H]^+^ calcd for C_17_H_19_N_3_O_6_S, 393.0911; found, 393.0919

#### Synthesis of 4-Methyl*-N-*(2-nitrophenyl)benzenesulfonamide **8a**

Nitroaniline **7a** (200 mg, 1.45 mmol) was dissolved in 0.5 mL of pyridine, and the reaction mixture was stirred for 30 min at 100 °C. Subsequently, *p*TsCl (606 mg, 3.2 mmol) was added portionwise over 3 h. The reaction was then stirred at 100 °C for another 24 h. Later, the reaction mixture was poured onto crushed ice, yielding a deep orange solid precipitate, which was recrystallized in MeOH (10 mL) to give pure product **8a** (205 mg, 48% yield) as a deep yellow crystalline solid. ^1^H NMR (400 MHz, CDCl_3_) δ 9.84 (s, 1H), 8.10 (dd, *J* = 8.4, 1.6 Hz, 1H), 7.84 (dd, *J* = 8.4, 1.3 Hz, 1H), 7.77–7.68 (m, 2H), 7.57 (ddd, *J* = 8.6, 7.3, 1.6 Hz, 1H), 7.28–7.23 (m, 2H), 7.14 (ddd, *J* = 8.6, 7.3, 1.3 Hz, 1H), 2.38 (s, 3H). ^13^C NMR (101 MHz, CDCl_3_) δ 145.0, 137.2, 136.0, 135.9, 134.1, 130.2, 127.4, 126.3, 123.9, 121.2, 21.7.

#### Synthesis of *N*-(4-Chloro-2-nitrophenyl)-4-methylbenzenesulfonamide **8b**

4-Chloronitroaniline **7b** (688 mg, 4 mmol) and *p*TsCl (1.52 g, 8 mmol) were dissolved in dry DCM (10 mL) and dry pyridine (12 mmol, 1 mL) was added. The mixture was stirred at room temperature for 24 h, after which another portion of *p*TsCl (380 mg, 2 mmol) was added. The reaction was stirred at room temperature for another 48 h. Later, the reaction mixture was shaken with 100 mL of water, and the aqueous phase was extracted with 3 × 100 mL of EtOAc. The combined organic layers were dried using MgSO_4_, filtered, and evaporated to dryness *in vacuo* yielding an orange oil, which was participated from EtOH/H_2_O (4:1, v/v) to give product **8b** (467 mg, 36% yield) as an orange solid. ^1^H NMR (400 MHz, CDCl_3_) δ 9.71 (s, 1H), 8.09 (d, *J* = 2.5 Hz, 1H), 7.83 (d, *J* = 9.0 Hz, 1H), 7.75–7.65 (m, 2H), 7.53 (dd, *J* = 9.0, 2.5 Hz, 1H), 7.30–7.25 (m, 2H), 2.39 (s, 3H). ^13^C NMR (101 MHz, CDCl_3_) δ 145.2, 137.3, 136.0, 135.6, 132.7, 130.3, 129.4, 127.4, 125.9, 122.5, 21.7.

#### Synthesis of *N*-(4-Methoxy-2-nitrophenyl)-4-methylbenzenesulfonamide **8c**

4-Methoxynitroaniline **7c** (336 mg, 2 mmol) and *p*TsCl (380 mg, 2 mmol) were dissolved in 1 mL of pyridine and stirred at room temperature for 6 h. Subsequently, the reaction mixture was poured into 150 mL of saturated NH_4_Cl solution and extracted into EtOAC (3 × 100 mL). The combined organic layers were evaporated to dryness *in vacuo*. The yellow-orange oil was dissolved in 4 mL of EtOH followed by the addition of 4 mL of water to form turbidity, which was removed after brief heating. The mixture was allowed to stand overnight to form yellow crystals of product **8c** (514 mg, 80% yield). ^1^H NMR (400 MHz, CDCl_3_) δ 9.24 (s, 1H), 7.79 (d, *J* = 9.2 Hz, 1H), 7.67–7.56 (m, 2H), 7.48 (d, *J* = 3.0 Hz, 1H), 7.25–7.14 (m, 3H), 3.81 (s, 3H), 2.37 (s, 3H). ^13^C NMR (101 MHz, CDCl_3_) δ 156.3, 144.7, 139.2, 135.7, 130.1, 127.3, 126.7, 124.9, 123.2, 109.2, 56.1, 21.7.

#### Synthesis of *N*-(2-Aminophenyl)-4-nitrobenzenesulfonamide **9a**

Compound **6a** (3.12 g, 8.08 mmol) was dissolved in 25 mL of a 25% TFA solution in DCM and the mixture stirred at room temperature for 2 h. TFA/DCM was removed under N_2_, and the orange oil was poured into 100 mL of saturated NaHCO_3_ solution and shaken vigorously until the reaction mixture stopped foaming. The resulting precipitate was filtered off under reduced pressure and dried to give the pure product **9a** (2.22 g, 95% yield) as an orange solid. ^1^H NMR (400 MHz, DMSO-*d*_6_) δ 8.49–8.22 (m, 2H), 8.01–7.82 (m, 2H), 6.91 (ddd, *J* = 8.0, 7.2, 1.5 Hz, 1H), 6.72 (dd, *J* = 7.8, 1.5 Hz, 1H), 6.62 (dd, *J* = 8.1, 1.5 Hz, 1H), 6.42 (ddd, *J* = 7.9, 7.2, 1.5 Hz, 1H), 4.91 (brs, 2H). ^13^C NMR (101 MHz, DMSO-*d*_6_) δ 149.7, 145.9, 144.4, 128.4, 127.9, 127.4, 124.3, 119.7, 116.2, 115.7

#### Synthesis of *N*-(2-Aminophenyl)-4-methylbenzenesulfonamide **9b**

**5a** (1.1 g, 5.3 mmol, 1 equiv) and *p*TsCl (1.1 g, 5.83 mmol, 1.1 equiv) was dissolved in 10 mL of DCM and pyridine (504 μL, 5.83 mmol, 1.1 equiv) was added to the stirring solution at room temperature. The reaction was monitored by LCMS. After 3 h, the reaction mixture was evaporated to dryness *in vacuo*. The clear oily product was shaken in 20 mL of water to precipitate a white crystalline substance, which was filtered off under reduced pressure. The crystalline product of **6b** was then transferred to a flask and dissolved in 20 mL of a 25% TFA solution in DCM. After the reaction was complete, the TFA/DCM solution was blown under N_2_. The purple oily substance was poured into 50 mL of a saturated NaHCO_3_ solution and vigorously shaken. The resulting precipitate was filtered off under reduced pressure and washed with water. After being dried, pure product **9b** (1.23 g, 89% yield) was obtained as a white solid. ^1^H NMR (400 MHz, CDCl_3_) δ 7.70–7.58 (m, 2H), 7.25 (d, *J* = 8.5 Hz, 2H), 7.04 (ddd, *J* = 8.1, 7.0, 1.8 Hz, 1H), 6.74 (dd, *J* = 8.1, 1.3 Hz, 1H), 6.59–6.47 (m, 2H), 3.35 (brs, 2H), 2.42 (s, 3H). ^13^C NMR (101 MHz, CDCl_3_) δ 144.5, 144.1, 136.1, 129.8, 129.1, 128.7, 127.7, 121.3, 118.8, 117.3, 21.7.

#### General Procedure for Direct Sulfonylation (Products **9c–f**)

The corresponding diamine **4b–e** (2 mmol) was dissolved in 15 mL of THF (**4b**, **4c**) or in 10 mL of DCM (**4d**, **4e**), and pyridine (160 μL, 2 mmol) was added to the solution. The reaction mixture was placed in an ice bath and cooled to 0 °C. Then, 4-NsCl (400 mg, 1.8 mmol, for synthesis of **9c** and **9d**) or *p*TsCl (380 mg, 2 mmol, for synthesis of **9e** and **9f**) was added in portion. The reaction was monitored by TLC. After 2 h, the reaction was quenched with 100 mL of water and extracted with 3 × 100 mL of DCM or 3 × 100 mL of EtOAc (**9e**). The combined organic layers were dried using MgSO_4_, filtered, and evaporated to dryness *in vacuo*. The crude products were purified by silica gel chromatography or crystallized from an appropriate solvent.

##### *N*-(2-Amino-3-chlorophenyl)-4-nitrobenzenesulfonamide **9c**

The crude product was purified by crystallization from EtOH to give pure product **9c** (430 mg, 66% yield) as a yellow-brown fine crystalline solid. ^1^H NMR (400 MHz, DMSO-*d*_*6*_) δ 8.44–8.28 (m, 2H), 8.01–7.82 (m, 2H), 7.12 (dd, *J* = 8.0, 1.5 Hz, 1H), 6.69 (dd, *J* = 7.9, 1.5 Hz, 1H), 6.46 (t, *J* = 8.0 Hz, 1H), 5.07 (brs, 2H). ^13^C NMR (101 MHz, DMSO-*d*_*6*_) δ 149.8, 145.5, 141.1, 128.4, 128.1, 126.2, 124.5, 121.3, 118.2, 116.2. HRMS (ESI pos.) *m*/*z*: [M + H]^+^ calcd for C_12_H_10_ClN_3_O_4_S, 328.0154; found, 328.0155

##### Methyl 4-Amino-3-((4-nitrophenyl)sulfonamido)benzoate **9d**

The crude product was purified by being suspended in EtOH and washed with EtOH/H_2_O (1:1, v/v) on a frit to give pure product **9d** (338 mg, 57% yield) as a beige solid. ^1^H NMR (400 MHz, DMSO-*d*_*6*_) δ 9.68 (brs, 1H), 8.46–8.28 (m, 2H), 7.98–7.84 (m, 2H), 7.52 (dd, *J* = 8.5, 2.1 Hz, 1H), 7.35 (d, *J* = 2.0 Hz, 1H), 6.63 (d, *J* = 8.5 Hz, 1H), 5.75 (s, 2H), 3.69 (s, 3H). ^13^C NMR (101 MHz, DMSO-*d*_6_) δ 165.7, 149.8, 149.1, 145.5, 129.5, 129.3, 128.5, 124.4, 118.7, 116.3, 114.6, 51.4. HRMS (ESI pos.) *m*/*z*: [M + H] ^+^ calculated for C_14_H_13_N_3_O_6_S, 352.0598; found, 352.0599

##### *N*-(2-Amino-5-nitrophenyl)-4-methylbenzenesulfonamide **9e**

The crude product was purified by crystallization in DCM to give **9e** (470 mg, 77%) as a yellow crystalline solid. ^1^H NMR (400 MHz, DMSO-*d*_6_) δ 9.48 (s, 1H), 7.81 (dd, *J* = 9.1, 2.7 Hz, 1H), 7.61 (d, *J* = 1.8 Hz, 1H), 7.60–7.58 (m, 2H), 7.36 (d, *J* = 8.1 Hz, 2H), 6.67 (d, *J* = 9.1 Hz, 1H), 6.46 (s, 2H), 2.36 (s, 3H). ^13^C NMR (101 MHz, DMSO-*d*_6_) δ 150.9, 143.5, 136.5, 135.3, 129.6, 126.9, 124.1, 122.8, 119.3, 113.8, 21.0.

##### *N*-(2-Amino-4,5-dimethylphenyl)-4-methylbenzenesulfonamide **9f**

The crude product was purified by silica gel chromatography using Hex/EtOAc with 1% AcOH (1:3, v/v) to give **9f** (355 mg, 59% yield) as a brown oil. ^1^H NMR (400 MHz, CDCl_3_) δ 7.67–7.60 (m, 2H), 7.26–7.23 (m, 2H), 6.53 (s, 1H), 6.31 (s, 1H), 2.42 (s, 3H), 2.11 (s, 3H), 1.95 (s, 3H). ^13^C NMR (101 MHz, CDCl_3_) δ 143.6, 141.8, 137.2, 136.7, 129.5, 129.5, 127.6, 126.9, 119.2, 118.6, 21.6, 19.5, 18.6.

#### 10 General Procedures for Catalytic Hydrogenation (Products **9b**, **9g**, and **9h**)

Compounds **8a–c** were dissolved in EtOH (1 mL per 0.1 mmol) and 10 mol% 10% Pd/C or 5 mol% PtO_2_ (for **9g**) was added to the mixture. Subsequently, the flask with the reaction mixture was sealed with a septum and bubbled with N_2_ and then with H_2_ for 15 min. The reaction mixture was stirred for an additional 2 h (H, Cl) or 16 h (OMe) at room temperature under a H_2_ atmosphere. The end of the reaction was indicated by LCMS. The reaction mixture was filtered through a microfilter, and the EtOH was evaporated *in vacuo*. The substance was either used without further purification or was purified by silica gel chromatography or crystallization.

##### *N*-(2-Aminophenyl)-4-methylbenzenesulfonamide **9b**

The product **9b** was obtained without further purification as a white solid (131 mg, 98%).

##### *N*-(2-Amino-4-methoxyphenyl)-4-methylbenzenesulfonamide **9g**

The crude product was purified by crystallization in EtOH/H_2_O (2:1, v/v) to give product **9g** (350 mg, 88% yield) as an orange solid. ^1^H NMR (400 MHz, CDCl_3_) δ 7.65–7.58 (m, 2H), 7.26–7.24 (m, 2H), 6.31 (d, *J* = 8.7 Hz, 1H), 6.24 (d, *J* = 2.7 Hz, 1H), 6.05 (dd, *J* = 8.7, 2.8 Hz, 1H), 5.94 (s, 1H), 3.71 (s, 3H), 2.42 (s, 3H). ^13^C NMR (101 MHz, CDCl_3_) δ 160.3, 146.5, 143.7, 136.2, 130.3, 129.7, 127.8, 127.1, 114.0, 104.4, 101.7, 55.4, 21.7.

##### *N*-(2-Amino-4-chlorophenyl)-4-methylbenzenesulfonamide **9h**

The crude product was purified by silica gel chromatography using Hex/EtOAc (7:3, v/v) to give pure product **9h** (330 mg, 81% yield) as a white solid. ^1^H NMR (400 MHz, CDCl_3_) δ 7.66–7.59 (m, 2H), 7.30–7.26 (m, 2H), 6.72 (d, *J* = 2.3 Hz, 1H), 6.47 (dd, *J* = 8.4, 2.3 Hz, 1H), 6.35 (d, *J* = 8.4 Hz, 1H), 5.92 (s, 1H), 4.18 (s, 2H), 2.43 (s, 3H). ^13^C NMR (101 MHz, CDCl_3_) δ 146.0, 144.3, 135.7, 134.6, 129.9, 129.9, 127.7, 119.5, 118.4, 116.6, 21.7.

#### General Procedure for *N*-Alkylation (Products **3a–c**, **10a–n**, and **13a,b**)

TPP (1.5–3 equiv) and DIAD (2–3 equiv) were dissolved in anhydrous THF (3 mL per 1 mmol TPP) and stirred for 30 min at 0 °C. The appropriate alkynol (1.5–2 equiv) and sulfonamide **2** (for **3a–c**) or **9a–h** (for **10a–n**) were added to the solution. The reaction was stirred at room temperature and monitored by LCMS or TLC. After 2 h, THF was evaporated *in vacuo*, and the reaction mixture was quenched with water (50 mL per 1 mmol of starting sulfonamide) and extracted with DCM (3x the same volume as the volume of water used). The combined organic layers were dried using MgSO_4_, filtered, and evaporated to dryness *in vacuo*. The crude product was purified by silica gel chromatography or recrystallized to remove the reaction byproduct, diisopropylhydrazine-1,2-dicarboxylate (DIHD).

##### *N*-(2-Iodophenyl)-4-nitro-*N*-(3-phenylprop-2-yn-1-yl)benzenesulfonamide **3a**

The crude product was purified by silica gel chromatography using DCM/Hex (5:1, v/v) to give pure product **3a** (641 mg, 64% yield) as a pale orange solid. ^1^H NMR (500 MHz, DMSO-*d*_6_) δ 8.49–8.36 (m, 2H), 8.14–8.06 (m, 2H), 8.03 (dd, *J* = 7.9, 1.4 Hz, 1H), 7.43 (ddd, *J* = 8.0, 7.4, 1.5 Hz, 1H), 7.39–7.28 (m, 3H), 7.24–7.17 (m, 3H), 7.07 (dd, *J* = 7.9, 1.5 Hz, 1H), 4.92 (d, *J* = 18.3 Hz, 1H), 4.54 (d, *J* = 18.3 Hz, 1H). ^13^C NMR (126 MHz, DMSO-*d*_6_) δ 150.1, 144.4, 140.4, 140.1, 131.1, 131.1, 130.0, 129.5, 129.3, 128.9, 128.6, 124.6, 121.3, 103.7, 85.5, 83.1, 41.8. HRMS (ESI pos.) *m*/*z*: [M + H]^+^ calcd for C_21_H_15_IN_2_O_4_S, 518.9870; found, 518.9878.

##### *N*-(2-Iodophenyl)-4-nitro-*N*-(3-(4-(trifluoromethyl)phenyl)prop-2-yn-1-yl)benzenesulfonamide **3b**

The crude product was purified by silica gel chromatography using DCM/hexane (5:2, v/v) to give the pure product **3b** (462 mg, 74% yield) as a yellow solid. ^1^H NMR (500 MHz, DMSO-*d*_6_) δ 8.45–8.38 (m, 2H), 8.13–8.07 (m, 2H), 8.03 (dd, *J* = 7.9, 1.5 Hz, 1H), 7.71–7.66 (m, 2H), 7.47–7.40 (m, 3H), 7.21 (td, *J* = 7.6, 1.6 Hz, 1H), 7.08 (dd, *J* = 7.9, 1.5 Hz, 1H), 4.94 (d, *J* = 18.4 Hz, 1H), 4.61 (d, *J* = 18.4 Hz, 1H). ^13^C NMR (101 MHz, DMSO-*d*_6_) δ 150.1, 144.3, 140.4, 140.1, 131.9, 131.1, 130.1, 129.5, 129.3, 128.9 (q, *J* = 32 Hz), 125.6, 125.5 (q, *J* = 3.7 Hz), 124.7, 123.8 (q, *J* = 273.5 Hz) 103.6, 86.1, 84.1, 41.8. (ESI pos.) *m*/*z*: [M + H]^+^ calcd for C_22_H_14_F_3_IN_2_O_4_S, 586.9744; found, 586.9745.

##### *N*-(2-Iodophenyl)-4-nitro-*N*-(3-(4-nitrophenyl)prop-2-yn-1-yl)benzenesulfonamide **3c**

The crude product was purified by silica gel chromatography using DCM to give product **3c** (135 mg, 48% yield) as an orange-yellow solid. ^1^H NMR (400 MHz, CDCl_3_) δ 8.39–8.30 (m, 2H), 8.21–8.09 (m, 2H), 8.09–7.97 (m, 2H), 7.94 (dd, *J* = 8.0, 1.4 Hz, 1H), 7.44–7.32 (m, 3H), 7.28 (dd, *J* = 7.9, 1.7 Hz, 1H), 7.14 (ddd, *J* = 7.9, 7.3, 1.7 Hz, 1H), 5.04 (d, *J* = 18.3 Hz, 1H), 4.46 (d, *J* = 18.3 Hz, 1H). ^13^C NMR (101 MHz, DMSO-*d*_6_) δ 150.2, 147.0, 144.2, 140.3, 140.2, 132.4, 131.1, 130.1, 129.5, 129.4, 128.0, 124.7, 123.8, 103.6, 88.5, 83.8, 41.8. HRMS (ESI pos.) *m*/*z*: [M + H]^+^ calcd for C_21_H_14_IN_3_O_6_S, 563.9721; found, 563.9736.

##### *N*-(2-Aminophenyl)-4-methyl-*N*-(3-(4-(trifluoromethyl)phenyl)prop-2-yn-1-yl)benzenesulfonamide **10a**

The crude product was purified by silica gel chromatography using Hex/EtOAc (3:1, v/v), followed by recrystallization from EtOH/H_2_O (1:1, v/v) to give pure product **10a** (725 mg, 64% yield) as a white crystalline solid. ^1^H NMR (400 MHz, CDCl_3_) δ 7.75–7.67 (m, 2H), 7.56–7.49 (m, 2H), 7.32–7.28 (m, 2H), 7.25 (dd, *J* = 8.6, 0.7 Hz, 2H), 7.13 (ddd, *J* = 8.1, 7.3, 1.5 Hz, 1H), 6.80 (dd, *J* = 8.1, 1.4 Hz, 1H), 6.67 (dd, *J* = 7.9, 1.5 Hz, 1H), 6.55 (ddd, *J* = 8.0, 7.3, 1.4 Hz, 1H), 4.82 (d, *J* = 17.8 Hz, 1H), 4.42 (d, *J* = 17.8 Hz, 1H), 2.41 (s, 3H). ^13^C NMR (101 MHz, CDCl_3_) δ 146.5, 144.1, 136.0, 131.8, 130.3 (q, *J* = 34.5 Hz), 130.1, 129.5, 129.3, 128.5, 126.3, 125.3 (q, *J* = 3.8 Hz), 124.7, 123.9 (q, *J* = 271.2 Hz), 118.0, 116.8, 86.4, 84.1, 41.7, 21.6. HRMS (ESI pos.) *m*/*z*: [M + H]^+^ calcd for C_23_H_19_F_3_N_2_O_2_S, 445.1192; found, 445.1189.

##### *N*-(2-Aminophenyl)-4-nitro*-N-*(3-(4-(trifluoromethyl)phenyl)prop-2-yn-1-yl)benzenesulfonamide **10b**

The crude product was purified by silica gel chromatography using Hex/EtOAc (3:1, v/v), followed by recrystallization from EtOH/H_2_O (2:1, v/v) to give pure product **10b** (793 mg, 69% yield) as a yellow crystalline solid. ^1^H NMR (400 MHz, CDCl_3_) δ 8.37–8.20 (m, 2H), 8.11–7.96 (m, 2H), 7.54 (d, *J* = 8.1 Hz, 2H), 7.30 (d, *J* = 8.1 Hz, 2H), 7.23–7.12 (m, 1H), 6.83 (dd, *J* = 8.1, 1.3 Hz, 1H), 6.71–6.48 (m, 2H), 4.94 (d, *J* = 17.9 Hz, 1H), 4.44 (d, *J* = 18.0 Hz, 1H), 4.23 (s, 2H). ^13^C NMR (101 MHz, CDCl_3_) δ 150.4, 146.3, 144.6, 131.8, 130.8 (q, *J* = 32.9 Hz), 130.7, 129.8, 129.1, 125.7, 125.5 (q, *J* = 38 Hz), 124.0, 123.8 (q, *J* = 272.3 Hz), 123.8, 118.3, 117.2, 85.5, 84.7, 42.1. HRMS (ESI pos.) *m*/*z*: [M + H]^+^ calcd for C_22_H_16_F_3_N_3_O_4_S, 476.0886; found, 476.0890.

##### *N*-(2-Aminophenyl)-4-nitro*-N-*(3-phenylprop-2-yn-1-yl)benzenesulfonamide **10c**

The crude product was purified by silica gel chromatography using Hex:EtOAc (4:1, v/v) followed by recrystallization from EtOH/H_2_O (1:1, v/v) to give **11c** (574 mg, 68% yield) as a beige solid. ^1^H NMR (400 MHz, CDCl_3_) δ 8.31–8.17 (m, 2H), 8.10–7.96 (m, 2H), 7.37–7.25 (m, 3H), 7.20–7.13 (m, 3H), 6.84 (dd, *J* = 8.1, 1.4 Hz, 1H), 6.70 (dd, *J* = 8.0, 1.5 Hz, 1H), 6.63–6.52 (m, 1H), 5.00 (d, *J* = 18.0 Hz, 1H), 4.34 (d, *J* = 18.0 Hz, 1H), 3.82 (bs, 2H). ^13^C NMR (101 MHz, CDCl_3_) δ 150.3, 146.1, 144.8, 131.5, 130.6, 130.0, 129.2, 129.1, 128.6, 124.6, 123.9, 121.9, 118.4, 117.2, 86.3, 8 3.1, 42.4. HRMS (ESI pos.) *m*/*z*: [M + H]^+^ calcd for C_21_H_17_N_3_O_4_S, 408.1013; found, 408.1012.

##### *N*-(2-Amino-6-chlorophenyl)-4-nitro*-N-*(3-phenylprop-2-yn-1-yl)benzenesulfonamide **10d**

The crude product was purified by silica gel chromatography using Hex/EtOAc (3:1, v/v) to give **10d** (335 mg, 74% yield) as a yellow solid. ^1^H NMR (400 MHz, CDCl_3_) δ 8.36–8.17 (m, 2H), 8.10–7.97 (m, 2H), 7.37–7.26 (m, 5H), 7.20–7.14 (m, 2H), 6.63 (dd, *J* = 8.0, 1.5 Hz, 1H), 6.51 (t, *J* = 8.0 Hz, 1H), 5.00 (d, *J* = 18.0 Hz, 1H), 4.66 (s, 2H), 4.34 (d, *J* = 18.0 Hz, 1H). ^13^C NMR (101 MHz, CDCl_3_) δ 150.4, 144.5, 143.6, 131.5, 130.7, 130.0, 129.2, 128.6, 127.7, 124.8, 124.0, 121.7, 120.8, 117.3, 86.6, 8 2.7, 42.3 HRMS (ESI pos.) *m*/*z*: [M + H]^+^ calcd for C_21_H_16_ClN_3_O_4_S, 442.0623; found, 442.0617.

##### Methyl 4-Amino-3-((4-nitro*-N-*(3-phenylprop-2-yn-1yl)phenyl)sulfonamido)benzoate **10f**

The crude product was purified by silica gel chromatography using Hex:EtOAc (7:3, v/v) followed by recrystallization from Hex/EtOAc (2:1, v/v) to give pure product **10f** (300 mg, 53% yield) as a yellow solid. ^1^H NMR (400 MHz, DMSO-*d*_6_) δ 8.39–8.29 (m, 2H), 8.09–8.01 (m, 2H), 7.64 (dd, *J* = 8.6, 2.0 Hz, 1H), 7.41–7.27 (m, 4H), 7.20 (dt, *J* = 6.7, 1.7 Hz, 2H), 6.77 (d, *J* = 8.7 Hz, 1H), 6.24 (s, 2H), 4.94 (d, *J* = 18.6 Hz, 1H), 4.34 (d, *J* = 18.6 Hz, 1H), 3.63 (s, 3H). ^13^C NMR (101 MHz, DMSO-*d*_6_) δ 165.4, 151.7, 149.9, 144.1, 131.9, 131.2, 131.0, 129.9, 128.9, 128.5, 124.2, 121.4, 120.7, 115.4, 114.7, 85.6, 83.9, 51.2, 41.2. HRMS (ESI pos.) *m*/*z*: [M + H]^+^ calcd for C_23_H_19_N_3_O_6_S, 466.1067; found, 466.1075.

##### *N*-(2-Amino-5-nitrophenyl)-4-methyl*-N-*(3-phenylprop-2-yn-1-yl)benzenesulfonamide **10g**

The crude product was purified by silica gel chromatography using Hex/EtOAc (7:3, v/v), followed by recrystallization from EtOH to give product **10g** (147 mg, 71% yield) as a white solid. ^1^H NMR (400 MHz, CDCl_3_) δ 8.05 (dd, *J* = 9.0, 2.5 Hz, 1H), 7.74–7.65 (m, 3H), 7.34–7.26 (m, 3H), 7.25–7.19 (m, 4H), 6.78 (d, *J* = 9.0 Hz, 1H), 5.05 (s, 2H), 4.79 (d, *J* = 17.8 Hz, 1H), 4.37 (d, *J* = 17.7 Hz, 1H), 2.37 (s, 3H). ^13^C NMR (101 MHz, CDCl_3_) δ 152.7, 145.1, 138.0, 134.2, 131.6, 129.8, 128.9, 128.7, 128.4, 126.4, 123.7, 121.9, 114.8, 86.8, 82.3, 42. 2, 21.7. HRMS (ESI pos.) *m*/*z*: [M + H]^+^ calcd for C_22_H_19_N_3_O_4_S, 422.1169; found, 422.1162.

##### *N*-(2-Amino-4,5-dimethylphenyl)-4-methyl*-N-*(3-phenylprop-2-yn-1-yl)benzenesulfonamide **10h**

The crude product was purified by silica gel chromatography, followed by recrystallization from EtOH to give product **11g** (119 mg; 43% yield) as a white solid. ^1^H NMR (400 MHz, CDCl_3_) δ 7.77–7.70 (m, 2H), 7.33–7.26 (m, 3H), 7.25–7.17 (m, 4H), 6.61 (s, 1H), 6.49 (s, 1H), 4.81 (d, *J* = 17.9 Hz, 1H), 4.34 (d, *J* = 17.9 Hz, 1H), 4.00 (s, 2H), 2.39 (s, 3H), 2.15 (s, 3H), 1.96 (s, 3H). ^13^C NMR (101 MHz, CDCl_3_) δ 143.9, 143.8, 138.6, 136.4, 131.5, 130.2, 129.3, 128.6, 128.5, 128.3, 126.1, 122.7, 122.7, 118.1, 85.4, 8 4.1, 42.0, 21.7, 19.8, 18.8. HRMS (ESI pos.) *m*/*z*: [M + H]^+^ calcd for C_24_H_24_N_2_O_2_S, 405.1631; found, 405.1627.

##### *N*-(2-Amino-4-methoxyphenyl)-4-methyl*-N-*(3-phenylprop-2-yn-1-yl)benzenesulfonamide **10i**

The crude product was purified by silica gel chromatography using Hex/EtOAc (7:3, v/v) followed by recrystallization from EtOH/water (2:1, v/v) to give product **10i** (270 mg, 71%) as an orange solid. ^1^H NMR (400 MHz, CDCl_3_) δ 7.80–7.64 (m, 2H), 7.31–7.13 (m, 7H), 6.59 (d, *J* = 8.8 Hz, 1H), 6.30 (d, *J* = 2.8 Hz, 1H), 6.09 (dd, *J* = 8.8, 2.8 Hz, 1H), 4.83 (d, *J* = 17.8 Hz, 1H), 4.32 (d, *J* = 17.8 Hz, 1H), 3.73 (s, 3H), 2.39 (s, 3H). ^13^C NMR (101 MHz, CDCl_3_) δ 160.8, 147.6, 143.8, 136.1, 131.6, 130.4, 129.4, 128.6, 128.5, 128.3, 122.6, 118.1, 104.1, 101.2, 85.4, 83.9, 55.3, 42.1, 21.6. HRMS (ESI pos.) *m*/*z*: [M + H]^+^ calcd for C_23_H_22_N_2_O_3_S, 407.1424; found, 407.1422.

##### *N*-(2-Amino-4-chlorophenyl)-4-methyl*-N-*(3-phenylprop-2-yn-1-yl)benzenesulfonamide **10j**

The crude product was purified by silica gel chromatography using Hex/EtOAc (7:3, v/v) followed by recrystallization from EtOH to give pure product **10j** (300 mg, 42% yield) as an orange solid. ^1^H NMR (400 MHz, CDCl_3_) δ 8.24–8.14 (m, 2H), 8.05–7.93 (m, 2H), 7.39–7.23 (m, 4H), 7.18 (ddd, *J* = 7.7, 7.2, 1.5 Hz, 1H), 7.12–7.10 (m, 1H), 6.84 (dd, *J* = 8.1, 1.4 Hz, 1H), 6.78 (dd, *J* = 8.0, 1.5 Hz, 1H), 6.56 (ddd, *J* = 8.5, 7.2, 1.4 Hz, 1H), 5.54 (q, *J* = 7.0 Hz, 1H), 4.23 (s, 2H), 1.39 (dd, *J* = 7.1, 0.8 Hz, 3H). ^13^C NMR (101 MHz, CDCl_3_) δ 150.2, 148.1, 144.2, 131.2, 130.6, 130.3, 129.8, 129.1, 128.6, 123.6, 121.9, 120.9, 118.0, 117.1, 87.7, 8 5.9, 48.9, 20.3. HRMS (ESI pos.) *m*/*z*: [M + H]^+^ calcd for C_22_H_19_ClN_2_O_2_S, 411.0929; found, 411.0934.

##### *N*-(2-Aminophenyl)-4-methyl*-N-*(prop-2-yn-1-yl)benzenesulfonamide **10k**

The crude product was purified by silica gel chromatography using Hex:EtOAc (7:3, v/v) followed by recrystallization from Et_2_O to give pure product **10k** (295 mg, 78% yield) as a white solid. ^1^H NMR (400 MHz, CDCl_3_) δ 8.04–7.85 (m, 2H), 7.57–7.53 (m, 2H), 7.38 (ddd, *J* = 8.2, 7.2, 1.6 Hz, 1H), 7.07 (dd, *J* = 8.1, 1.4 Hz, 1H), 6.87 (dd, *J* = 7.9, 1.6 Hz, 1H), 6.45 (ddd, *J* = 7.9, 7.2, 1.4 Hz, 1H), 4.81 (d, *J* = 17.6 Hz, 1H), 4.51 (d, *J* = 17.5 Hz, 1H), 3.03 (bs, 2H), 2.71 (s, 3H), 2.42 (t, *J* = 2.5 Hz, 1H). ^13^C NMR (101 MHz, CDCl_3_) δ 146.4, 144.1, 135.7, 130.0, 129.5, 129.4, 128.5, 124.7, 118.1, 116.8, 78.2, 73.7, 40.9, 21.7. HRMS (ESI pos.) *m*/*z*: [M + H]^+^ calcd for C_16_H_16_N_2_O_2_S, 301.1005; found, 301.1003.

##### *N*-(2-Aminophenyl)*-N-*(but-2-yn-1-yl)-4-methylbenzenesulfonamide **10l**

The crude product was purified by silica gel chromatography using Hex:EtOAc (7:3, v/v) to give **10l** (185 mg, 42% yield) as a brown oil. ^1^H NMR (400 MHz, CDCl_3_) δ 7.69–7.60 (m, 2H), 7.31–7.22 (m, 2H), 7.09 (ddd, *J* = 8.0, 7.2, 1.6 Hz, 1H), 6.77 (dd, *J* = 8.0, 1.4 Hz, 1H), 6.61 (dd, *J* = 7.9, 1.6 Hz, 1H), 6.53 (ddd, *J* = 8.0, 7.6, 1.4 Hz, 1H), 4.52 (d, *J* = 17.4 Hz, 1H), 4.11 (d, *J* = 17.6 Hz, 1H), 3.77 (bs, 2H), 2.44 (s, 3H), 1.66 (t, *J* = 2.4 Hz, 3H). ^13^C NMR (101 MHz, CDCl_3_) δ 146.4, 143.8, 136.1, 129.8, 129.4, 129.2, 128.6, 125.2, 118.0, 116.7, 81.5, 73.5, 41.7, 22.1, 21.8, 21.7, 3.6. HRMS (ESI poz.) *m*/*z*: [M + H]^+^ calcd for C_17_H_18_N_2_O_2_S, 315.1162; found, 315.1159.

##### *N*-(2-Aminophenyl)*-N-*(hex-3-yn-2-yl)-4-nitrobenzenesulfonamide **10m**

The crude product was purified by silica gel chromatography using Hex:EtOAc (2:1, v/v) followed by recrystallization from EtOH to give pure product **10m** (690 mg, 63% yield) as a yellow solid. ^1^H NMR (400 MHz, CDCl _3_) δ 8.32–8.27 (m, 2H), 8.00–7.96 (m, 2H), 7.15 (ddd, *J* = 8.1, 7.2, 1.5 Hz, 1H), 6.81 (dd, *J* = 8.1, 1.4 Hz, 1H), 6.69 (dd, *J* = 8.1, 1.5 Hz, 1H), 6.52 (ddd, *J* = 8.0, 7.3, 1.5 Hz, 1H), 5.30 (qt, *J* = 7.0, 2.1 Hz, 1H), 1.99 (qdd, *J* = 7.3, 2.1, 1.0 Hz, 2H), 1.28 (d, *J* = 7.1 Hz, 3H), 0.95 (t, *J* = 7.5 Hz, 3H). ^13^C NMR (101 MHz, CDCl_3_) δ 150.3, 148.0, 144.4, 130.5, 130.4, 130.0, 123.4, 120.9, 117.8, 117.0, 87.7, 78.1, 48.7, 20.8, 13.5, 12.3. HRMS (ESI pos.) *m*/*z*: [M + H]^+^ calcd for C_18_H_19_N_3_O_4_S, 374.1169; found, 374.1161.

##### *N*-(2-Aminophenyl)-4-nitro*-N-*(4-phenylbut-3-yn-2-yl)benzenesulfonamide **10n**

The crude product was purified by silica gel chromatography using Hex/EtOAc (7:3, v/v) and subsequent precipitation from Et_2_O to give pure product **10n** (180 mg, 43% yield) as a white solid. ^1^H NMR (400 MHz, CDCl_3_) δ 7.76–7.66 (m, 2H), 7.34–7.23 (m, 5H), 7.22–7.15 (m, 2H), 6.79 (d, *J* = 2.3 Hz, 1H), 6.61 (d, *J* = 8.5 Hz, 1H), 6.50 (dd, *J* = 8.5, 2.4 Hz, 1H), 4.81 (d, *J* = 17.7 Hz, 1H), 4.35 (d, *J* = 17.8 Hz, 1H), 2.41 (s, 3H). ^13^C NMR (101 MHz, CDCl_3_) δ 147.6, 144.2, 135.7, 135.6, 131.6, 130.5, 129.6, 128.7, 128.6, 128.4, 123.4, 122.4, 118.0, 116.3, 85.7, 83.4, 41.9, 21.7. HRMS (ESI pos.) *m*/*z*: [M + H]^+^ calcd for C_22_H_19_N_3_O_4_S, 422.1169; found, 422.1165.

##### *N*-(2-Aminophenyl)-4-nitro*-N-*(4-phenylbut-3-yn-1-yl)benzenesulfonamide **13a**

The crude product was purified by recrystallization in DCM to give pure product **13a** (280 mg, 67% yield) as a yellow solid. ^1^H NMR (400 MHz, DMSO-*d*_6_) δ 8.40–8.30 (m, 2H), 8.06–7.93 (m, 2H), 7.34 (s, 5H), 7.05 (ddd, *J* = 8.4, 7.1, 1.5 Hz, 1H), 6.76 (dd, *J* = 8.1, 1.4 Hz, 1H), 6.55 (dd, *J* = 7.9, 1.6 Hz, 1H), 6.44 (ddd, *J* = 8.2, 7.2, 1.4 Hz, 1H), 5.16 (s, 2H), 3.96 (dt, *J* = 13.2, 7.2 Hz, 1H), 3.69–3.50 (m, 1H), 2.62 (dt, *J* = 17.1, 7.2 Hz, 1H), 2.55–2.51 (m, 1H). ^13^C NMR (101 MHz, DMSO-*d*_6_) δ 149.8, 147.7, 144.2, 131.6, 129.6, 129.1, 128.5, 128.2, 124.5, 122.7, 121.5, 116.1, 115.9, 87.4, 82.0, 50.0, 19.0. HRMS (ESI pos.) *m*/*z*: [M + H]^+^ calcd for C_22_H_19_N_3_O_4_S, 422.1169; found, 422.1174.

##### *N*-(2-Aminophenyl)-4-nitro*-N-*(5-phenylpent-4-yn-1-yl)benzenesulfonamide **13b**

The crude product was purified by silica gel chromatography using Hex:EtOAc (3:1, v/v) to give product **13b** (305 mg, 71% yield) as an orange oil. ^1^H NMR (400 MHz, CDCl_3_) δ 8.36–8.27 (m, 2H), 7.92–7.82 (m, 2H), 7.37–7.32 (m, 2H), 7.32–7.21 (m, 3H), 7.12 (ddd, *J* = 8.1, 7.3, 1.5 Hz, 1H), 6.81 (dd, *J* = 8.1, 1.4 Hz, 1H), 6.55 (ddd, *J* = 7.9, 7.3, 1.4 Hz, 1H), 6.26 (dd, *J* = 7.9, 1.5 Hz, 1H), 4.25 (s, 2H), 4.14–4.01 (m, 1H), 3.35 (ddd, *J* = 13.0, 8.0, 5.2 Hz, 1H), 2.53–2.44 (m, 2H), 2.03–1.61 (m, 2H). ^13^C NMR (101 MHz, CDCl_3_) δ 150.4, 147.0, 143.7, 131.7, 130.1, 129.4, 128.4, 127.9, 127.9, 124.2, 124.0, 123.7, 118.6, 117.5, 88.4, 81.8, 51.5, 27.6, 17.0. HRMS (ESI pos.) *m*/*z*: [M + H]^+^ calcd for C_23_H_21_N_3_O_4_S, 436.1326; found, 436.1323.

#### 12 General Procedure for Azidation (Products **11a–n** and **14a,b**)

50 mg of **10a–n** or **14a,b** was dissolved in 2 mL of 50% AcOH/ACN (1:1, v/v) (**10a,b**) or in 2 mL of 90% AcOH/dioxane (1:1, v/v) (**10c, 10h, 10k, 10l, 13a, 13b**) or in 2–10 mL (depending on solubility) of 10% HCl/ACN (2:3, v/v) (**10f, 10g, 10i, 10j, 10m, 10n**). The solution was cooled in an ice bath to 0 °C and a cooled solution of NaNO_2_ (1.2 equiv in 1 mL of water) was added dropwise into the stirring mixture. The reaction was then stirred at 0 °C for 30 min, and then the solution of NaN_3_ (2 equiv in 1 mL of water) was added dropwise. If the reaction mixture was not clear, it was filtered through a microfilter before adding NaN_3_. The reaction was stirred for another 30 min and then the resulting precipitate was filtered off under reduced pressure. The oily products **11i, 11j, 11k**, and **14b** were isolated by addition of water (50 mL) followed by extraction into DCM (3 × 50 mL). The combined organic layers were dried using MgSO_4_, filtered, and evaporated to dryness *in vacuo*.

##### *N*-(2-Azidophenyl)-4-methyl*-N-*(3-(4-(trifluoromethyl)phenyl)prop-2-yn-1-yl)benzenesulfonamide **11a**

Pale yellow solid (201 mg, 95%). ^1^H NMR (400 MHz, CDCl_3_) δ 8.48–8.19 (m, 2H), 8.05–7.95 (m, 2H), 7.56–7.50 (m, 2H), 7.45 (ddd, *J* = 8.1, 7.4, 1.6 Hz, 1H), 7.34–7.26 (m, 3H), 7.24–7.12 (m, 2H), 4.71 (s, 2H).

##### *N*-(2-Azidophenyl)-4-nitro*-N-*(3-(4-(trifluoromethyl)phenyl)prop-2-yn-1-yl)benzenesulfonamide **11b**

Orange solid (540 mg, 64%). ^1^H NMR (400 MHz, DMSO-*d*_6_) δ 7.75–7.65 (m, 4H), 7.48–7.39 (m, 5H), 7.30 (dd, *J* = 8.1, 1.4 Hz, 1H), 7.18 (td, *J* = 7.6, 1.5 Hz, 1H), 7.07 (dd, *J* = 7.9, 1.5 Hz, 1H), 4.72 (s, 2H), 2.39 (s, 3H).

##### *N*-(2-Azidophenyl)-4-nitro*-N-*(3-phenylprop-2-yn-1-yl)benzenesulfonamide **11c**

Beige solid (115 mg, 72%). ^1^H NMR (400 MHz, CDCl_3_) δ 8.35–8.28 (m, 2H), 8.06–7.97 (m, 2H), 7.44 (td, *J* = 7.7, 1.6 Hz, 1H), 7.29 (ddd, *J* = 7.0, 5.5, 2.2 Hz, 2H), 7.25–7.10 (m, 6H), 4.69 (s, 2H). ^13^C NMR (101 MHz, CDCl_3_) δ 145.9, 132.4, 131.6, 130.9, 129.3, 129.0, 129.0, 128.5, 125.4, 124.1, 122.0, 120.0, 86.5, 82.6, 42.1.

##### Methyl 4-Azido-3-((4-nitro-N-(3-phenylprop-2-yn-1-yl)phenyl)sulfonamido)benzoate **11f**

White solid (66 mg, 62%). ^1^H NMR (400 MHz, CDCl_3_) δ 8.33–8.25 (m, 2H), 8.10 (dd, *J* = 8.4, 1.9 Hz, 1H), 8.06–7.99 (m, 2H), 7.87 (d, *J* = 1.9 Hz, 1H), 7.33–7.27 (m, 2H), 7.26–7.23 (m, 2H), 7.21–7.13 (m, 2H), 4.67 (s, 2H), 3.86 (s, 3H).

##### *N*-(2-Azido-5-nitrophenyl)-4-methyl*-N-*(3-phenylprop-2-yn-1-yl)benzenesulfonamide **11g**

Yellow solid (90 mg, 85%). ^1^H NMR (400 MHz, DMSO-*d*_6_) δ 8.30 (dd, *J* = 9.0, 2.7 Hz, 1H), 7.90 (d, *J* = 2.6 Hz, 1H), 7.76–7.67 (m, 2H), 7.55 (d, *J* = 9.0 Hz, 1H), 7.43 (d, *J* = 8.1 Hz, 2H), 7.40–7.31 (m, 3H), 7.23–7.14 (m, 2H), 4.75 (s, 2H), 2.38 (s, 3H).

##### *N*-(2-Azido-4,5-dimethylphenyl)-4-methyl*-N-*(3-phenylprop-2-yn-1-yl)benzenesulfonamide **11h**

Orange solid (67 mg, 78%). Not characterized due to partial spontaneous cyclization prior to NMR analysis.

##### *N*-(2-Azido-4-methoxyphenyl)-4-methyl*-N-*(3-phenylprop-2-yn-1-yl)benzenesulfonamide **11i**

Colorless oil. Used for the next step without isolation.

##### *N*-(2-Azido-4-chlorophenyl)-4-methyl*-N-*(3-phenylprop-2-yn-1-yl)benzenesulfonamide **11j**

Colorless oil. Used for the next step without isolation.

##### *N*-(2-Azidophenyl)-4-methyl*-N-*(prop-2-yn-1-yl)benzenesulfonamide **11k**

Orange oily substance. Used for the next step without isolation.

##### *N*-(2-Azidophenyl)*-N-*(but-2-yn-1-yl)-4-methylbenzenesulfonamide **11l**

Orange solid (110 mg, 73% yield). ^1^H NMR (400 MHz, CDCl_3_) δ 7.70–7.60 (m, 2H), 7.38–7.32 (m, 1H), 7.28 (d, *J* = 8.1 Hz, 2H), 7.19–7.03 (m, 3H), 4.35 (s, 2H), 2.43 (s, 3H), 1.66 (t, *J* = 2.4 Hz, 3H). ^13^C NMR 101 MHz, CDCl_3_ δ 143.8, 139.3, 136.9, 132.5, 130.1, 130.0, 129.4, 128.2, 125.0, 119.9, 81.8, 73.2, 41.3, 21.7, 3.6.

##### *N*-(2-Azidophenyl)*-N-*(hex-3-yn-2-yl)-4-nitrobenzenesulfonamide **11m**

Brown-yellow solid (155 mg, 90% yield). ^1^H NMR (400 MHz, CDCl_3_) δ 8.35–8.30 (m, 2H), 8.01–7.95 (m, 2H), 7.44 (ddd, *J* = 8.1, 5.5, 3.5 Hz, 1H), 7.25 (dd, *J* = 8.5, 1.0 Hz, 1H), 7.09–6.99 (m, 2H), 5.21 (qt, *J* = 7.0, 2.1 Hz, 1H), 1.96 (qd, *J* = 7.5, 2.2 Hz, 2H), 1.28 (d, *J* = 7.0 Hz, 3H), 0.92 (t, *J* = 7.5 Hz, 3H). ^13^C NMR (101 MHz, CDCl_3_) δ 145.5, 142.0, 131.7, 130.9, 129.9, 126.7, 124.7, 123.7, 120.1, 87.8, 77.8, 48.3, 21.3, 13.5, 12.2.

##### *N*-(2-Azidophenyl)-4-methyl-*N*-(4-phenylbut-3-yn-2-yl)benzenesulfonamide **11n**

White solid (66 mg, 62%). Not characterized due to partial spontaneous cyclization prior to NMR analysis.

##### *N*-(2-Azidophenyl)-4-nitro*-N-*(4-phenylbut-3-yn-1-yl)benzenesulfonamide **14a**

Yellow solid (126 mg, 79%). ^1^H NMR (400 MHz, CDCl_3_) δ 8.31–8.23 (m, 2H), 7.96–7.88 (m, 2H), 7.45–7.40 (m, 1H), 7.34 (dd, *J* = 7.9, 1.6 Hz, 1H), 7.31–7.25 (m, 5H), 7.20–7.13 (m, 2H), 3.87 (bs, 2H), 2.63 (t, *J* = 7.1 Hz, 2H). ^13^C NMR (101 MHz, CDCl_3_) 145.8, 139.0, 133.5, 131.7, 130.8, 129.0, 128.4, 128.4, 128.2, 125.6, 124.1, 123.2, 119.8, 86.0, 82.9, 50.1, 20.5.

##### *N*-(2-Azidophenyl)-4-nitro*-N-*(5-phenylpent-4-yn-1-yl)benzenesulfonamide **14b**

Brown oil (240 mg, 75% yield). Not characterized; used crude for the next step.

#### 13 General Procedure for Cyclization (**12a–n** and **15a,b**)

Azide intermediate was dissolved in DMSO (1 mL to 50 mg) and subsequently, the mixture was stirred at 45 °C for 5 h (**11a–n**) or at 90 °C for 3 h (**15a**) or at 90 °C for 48 h (**15b**). Products **12a–n** and **15a** were obtained by precipitation from DMSO after the addition of water (1–2 mL). The oily product **15b** was poured into 50 mL of water and subsequently extracted into EtOAc (3 × 50 mL). The combined organic layers were dried using MgSO_4_, filtered, and evaporated to dryness *in vacuo*.

##### 5-Tosyl-3-(4-(trifluoromethyl)phenyl)-4,5-dihydro-[1,2,3]triazolo[1,5-*a*]quinoxaline **12a**

Beige solid (75 mg, 94%). ^1^H NMR (400 MHz, CDCl_3_) δ 8.02–7.97 (m, 1H), 7.97–7.89 (m, 1H), 7.80 (s, 4H), 7.56–7.49 (m, 2H), 6.97–6.89 (m, 2H), 6.87–6.75 (m, 2H), 5.23 (s, 2H), 2.29 (s, 3H). ^13^C NMR (101 MHz, CDCl_3_) δ 145.0, 141.4, 133.4, 130.7 (q, *J* = 33.6 Hz), 129.5, 129.4, 129.1, 129.0, 128.7, 127.3, 126.7, 126.5, 126.4 (q, *J* = 3.5 Hz), 124.1 (q, *J* = 272 Hz), 125.2, 117.5, 42.6, 21.6. HRMS (ESI pos.) *m*/*z*: [M + H]^+^ calcd for C_23_H_17_F_3_N_4_O_2_S, 471.1097; found, 471.1101.

##### 5-((4-Nitrophenyl)sulfonyl)-3-(4-(trifluoromethyl)phenyl)-4,5-dihydro-[1,2,3]triazolo[1,5-*a*]quinoxaline **12b**

Beige solid (550 mg, 87%). ^1^H NMR (400 MHz, TFA-*d*) δ 8.15–8.06 (m, 3H), 8.00–7.90 (m, 3H), 7.82–7.67 (m, 4H), 7.55–7.47 (m, 2H), 5.47 (d, *J* = 1.6 Hz, 2H). ^13^C NMR (101 MHz, TFA-*d*) δ 153.3, 144.3, 141.5, 137.4 (q, *J* = 33.8 Hz), 135.0, 132.8, 131.5, 130.6, 130.5, 130.3, 129.7, 129.6 (q, *J* = 3.7 Hz), 129.5, 127.6, 126.9, 125.5 (q, *J* = 271.5 Hz) 120.9, 43.6. HRMS (ESI pos.) *m*/*z*: [M + H]^+^ calcd for C_22_H_14_F_3_N_5_O_4_S, 502.0791; found, 502.0793.

##### 5-((4-Nitrophenyl)sulfonyl)-3-phenyl-4,5-dihydro-[1,2,3]triazolo[1,5-*a*]quinoxaline **12c**

Beige solid (132 mg, 83%). ^1^H NMR (400 MHz, CDCl_3_) δ 8.06–7.98 (m, 1H), 7.95–7.90 (m, 1H), 7.89–7.83 (m, 2H), 7.67–7.45 (m, 7H), 7.23–7.16 (m, 2H), 5.25 (s, 2H). ^13^C NMR (101 MHz, CDCl_3_) δ 150.3, 143.2, 141.8, 129.8, 129.6, 129.5, 129.3, 129.2, 128.8, 128.8, 127.9, 126.6, 126.5, 124.0, 123.9, 117.8, 42.9. HRMS (ESI pos.) *m*/*z*: [M + H]^+^ calcd for C_21_H_15_N_5_O_4_S, 434.0918; found, 434.0908.

##### Methyl 5-((4-Nitrophenyl)sulfonyl)-3-phenyl-4,5-dihydro-[1,2,3]triazolo[1,5-*a*]quinoxaline-7-carboxylate **12f**

White solid (20 mg, 95%). ^1^H NMR (400 MHz, CDCl_3_) δ 8.57 (d, *J* = 1.7 Hz, 1H), 8.24 (dd, *J* = 8.4, 1.8 Hz, 1H), 8.08 (d, *J* = 8.4 Hz, 1H), 7.90–7.85 (m, 2H), 7.64–7.55 (m, 4H), 7.53–7.47 (m, 1H), 7.24–7.19 (m, 2H), 5.27 (s, 2H), 4.02 (s, 3H). ^13^C NMR (101 MHz, CDCl_3_) δ 165.2, 150.4, 143.5, 141.7, 132.2, 130.9, 130.7, 130.1, 129.7, 129.4, 129.0, 127.9, 126.7, 126.4, 124.1, 124.0, 117.8, 53.0, 42.8. HRMS (ESI pos.) *m*/*z*: [M + H]^+^ calcd for C_23_H_17_N_5_O_6_S, 492.0972; found, 492.0965.

##### 7-Nitro-3-phenyl-5-tosyl-4,5-dihydro-[1,2,3]triazolo[1,5-*a*]quinoxaline **12g**

Yellow solid (20 mg, 85%). ^1^H NMR (400 MHz, CDCl_3_) δ 8.78 (d, *J* = 2.4 Hz, 1H), 8.37 (dd, *J* = 8.9, 2.4 Hz, 1H), 8.17 (d, *J* = 8.9 Hz, 1H), 7.65–7.62 (m, 2H), 7.58–7.53 (m, 2H), 7.51–7.44 (m, 1H), 7.01–6.97 (m, 2H), 6.89–6.84 (m, 2H), 5.27 (s, 2H), 2.27 (s, 3H). ^13^C NMR (101 MHz, CDCl_3_) δ 146.9, 145.5, 143.4, 133.3, 129.8, 129.5, 129.2, 129.2, 128.7, 128.0, 126.7, 126.6, 126.4, 124.6, 124.4, 123.9, 118.0, 42.4, 21.7. HRMS (ESI pos.) *m*/*z*: [M + H]^+^ calcd for C_22_H_17_N_5_O_4_S, 448.1074; found, 448.1076.

##### 7,8-Dimethyl-3-phenyl-5-tosyl-4,5-dihydro-[1,2,3]triazolo[1,5-*a*]quinoxaline **12h**

Beige solid (16 mg, 81%). ^1^H NMR (400 MHz, CDCl_3_) δ 7.75 (s, 1H), 7.69–7.57 (m, 3H), 7.57–7.47 (m, 2H), 7.49–7.38 (m, 1H), 6.98–6.90 (m, 2H), 6.85–6.76 (m, 2H), 5.15 (s, 2H), 2.40 (s, 3H), 2.38 (s, 3H), 2.26 (s, 3H). ^13^C NMR (101 MHz, CDCl_3_) δ 144.6, 142.7, 138.2, 137.6, 133.6, 130.1, 129.4, 129.3, 128.6, 127.3, 126.7, 126.6, 124.8, 124.1, 118.0, 42.8, 21.6, 20.0, 19.9. HRMS (ESI pos.) *m*/*z*: [M + H]^+^ calcd for C_24_H_22_N_4_O_2_S, 431.1536; found, 431.1532.

##### 8-Methoxy-3-phenyl-[1,2,3]triazolo[1,5-*a*]quinoxaline **12i**

White solid (150 mg, 71% after two steps). ^1^H NMR (400 MHz, CDCl_3_) δ 7.79 (d, *J* = 9.0 Hz, 1H), 7.68–7.58 (m, 2H), 7.58–7.49 (m, 2H), 7.49–7.39 (m, 2H), 7.00 (dd, *J* = 9.0, 2.9 Hz, 1H), 6.97–6.86 (m, 2H), 6.87–6.77 (m, 2H), 5.16 (s, 2H), 3.91 (s, 3H), 2.26 (s, 3H). ^13^C NMR (101 MHz, CDCl_3_) δ 159.9, 144.7, 142.9, 133.3, 130.3, 130.1, 129.9, 129.4, 129.3, 128.7, 126.7, 126.6, 124.3, 119.8, 114.9, 101.9, 56.1, 42.7, 21.6. HRMS (ESI pos.) *m*/*z*: [M + H]^+^ calcd for C_23_H_20_N_4_O_3_S, 433.1329; found, 433.1328.

##### 8-Chloro-3-phenyl-[1,2,3]triazolo[1,5-*a*]quinoxaline **12j**

White solid (154 mg, 85% after two steps). ^1^H NMR (400 MHz, CDCl_3_) δ 7.98 (d, *J* = 2.3 Hz, 1H), 7.83 (d, *J* = 8.7 Hz, 1H), 7.65–7.59 (m, 2H), 7.56–7.50 (m, 2H), 7.48–7.41 (m, 2H), 7.01–6.88 (m, 2H), 6.88–6.77 (m, 2H), 5.18 (s, 2H), 2.26 (s, 3H). ^13^C NMR (101 MHz, CDCl_3_) δ 145.1, 143.0, 134.7, 133.3, 130.1, 130.0, 129.6, 129.6, 129.4, 128.9, 128.5, 126.6, 126.5, 125.7, 124.2, 117.6, 42.6, 21.6. HRMS (ESI pos.) *m*/*z*: [M + H]^+^ calcd for C_22_H_17_ClN_4_O_2_S, 437.0834; found, 437.0831.

##### 5-Tosyl-4,5-dihydro-[1,2,3]triazolo[1,5-*a*]quinoxaline **12k**

White solid (30 mg, 32% after two steps). ^1^H NMR (400 MHz, CDCl_3_) δ 8.00–7.88 (m, 2H), 7.53–7.43 (m, 2H), 7.40 (d, *J* = 0.9 Hz, 1H), 7.09–7.03 (m, 2H), 6.96–6.89 (m, 2H), 5.07 (d, *J* = 0.8 Hz, 2H), 2.25 (s, 3H). ^13^C NMR (101 MHz, CDCl_3_) δ 144.9, 133.5, 129.5, 129.3, 128.8, 128.7, 128.4, 128.3, 127.1, 126.7, 117.3, 42.1, 21.6. HRMS (ESI pos.) *m*/*z*: [M + H]^+^ calcd for C_16_H_14_N_4_O_2_S, 327.0910; found, 327.0900.

##### 3-Methyl-5-tosyl-4,5-dihydro-[1,2,3]triazolo[1,5-*a*]quinoxaline **12l**

White solid (28 mg, 67%). ^1^H NMR (400 MHz, CDCl_3_) δ 8.02–7.82 (m, 2H), 7.54–7.38 (m, 2H), 7.12–7.00 (m, 2H), 6.94 (d, *J* = 8.1 Hz, 2H), 4.94 (s, 2H), 2.26 (s, 3H), 2.26 (s, 3H). ^13^C NMR (101 MHz, CDCl_3_) δ 144.9, 138.3, 133.8, 129.6, 129.4, 128.8, 128.2, 127.0, 126.5, 124.9, 117.2, 41.8, 21.6, 9.9. HRMS (ESI pos.) *m*/*z*: [M + H]^+^ calcd for C_17_H1_6_N_4_O_2_S, 341.1067; found, 341.1064.

##### 3-Ethyl-4-methyl-5-((4-nitrophenyl)sulfonyl)-4,5-dihydro-[1,2,3]triazolo[1,5-*a*]quinoxaline **12m**

Yellow solid (20 mg, 70%). ^1^H NMR (400 MHz, CDCl_3_) δ 8.00–7.87 (m, 4H), 7.57–7.46 (m, 2H), 7.33–7.27 (m, 2H), 5.74 (q, *J* = 7.1 Hz, 1H), 2.68 (dd, *J* = 7.6, 2.5 Hz, 2H), 1.37 (d, *J* = 7.1 Hz, 3H), 1.32 (t, *J* = 7.6 Hz, 3H). ^13^C NMR (101 MHz, CDCl_3_) δ 150.3, 143.5, 142.0, 129.6, 129.5, 129.0, 128.6, 128.5, 127.8, 123.9, 123.8, 117.4, 49.0, 20.7, 18.3, 13. 8. HRMS (ESI pos.) *m*/*z*: [M + H]^+^ calcd for C_18_H_17_N_5_O_4_S, 400.1074; found, 400.1075.

##### 4-Methyl-3-phenyl-[1,2,3]triazolo[1,5-*a*]quinoxaline **12n**

White solid (27 mg, 99%). ^1^H NMR (400 MHz, DMSO-*d*_6_) δ 8.09–7.98 (m, 1H), 7.97–7.90 (m, 2H), 7.89–7.83 (m, 1H), 7.73–7.65 (m, 4H), 7.63–7.56 (m, 2H), 7.54–7.46 (m, 1H), 7.28–7.19 (m, 2H), 5.87 (q, *J* = 7.0 Hz, 1H), 1.46 (d, *J* = 7.0 Hz, 3H). ^13^C NMR (101 MHz, DMSO-*d*_6_) δ 149.8, 141.1, 140.9, 129.9, 129.4, 129.3, 129.1, 129.0, 128.7, 128.2, 128.1, 127.7, 126.1, 124.2, 123.7, 117.3, 48.8, 19.4. HRMS (ESI pos.) *m*/*z*: [M + H]^+^ calcd for C_22_H_17_N_5_O_4_S, 448.1074; found, 448.1074.

##### 6-((4-Nitrophenyl)sulfonyl)-3-phenyl-5,6-dihydro-4H-benzo[*b*][1,2,3]triazolo[1,5-*d*][1,4]diazepine **15a**

Orange solid (150 mg, 94%). ^1^H NMR (400 MHz, DMSO-*d*_6_) δ 8.06–7.95 (m, 2H), 7.85–7.68 (m, 4H), 7.61–7.52 (m, 4H), 7.52–7.45 (m, 2H), 7.45–7.38 (m, 1H), 4.36 b(s, 2H), 3.05 (brs, 2H). ^13^C NMR (101 MHz, DMSO-*d*_6_) δ 149.2, 143.9, 143.0, 134.5, 133.7, 131.1, 130.2, 130.1, 130.0, 128.9, 128.6, 128.1, 127.3, 126.6, 124.4, 51.9, 20.8. HRMS (ESI pos.) *m*/*z*: [M + H]^+^ calcd for C_22_H_17_N_5_O_4_S, 448.1074; found, 448.1076.

##### 7-((4-Nitrophenyl)sulfonyl)-3-phenyl-4,5,6,7-tetrahydrobenzo[*b*][1,2,3]triazolo[1,5-*d*][1,4]diazocine **15b**

Isolated as brown oil (216 mg, 68% yield after two steps). Recrystallization from EtOH gave a brown crystalline solid. ^1^H NMR (400 MHz, CDCl_3_) δ 8.16–8.01 (m, 2H), 7.75–7.68 (m, 1H), 7.68–7.60 (m, 2H), 7.60–7.50 (m, 5H), 7.49–7.42 (m, 2H), 7.42–7.36 (m, 1H), 4.72 (brs, 1H), 3.23 (brs, 2H), 2.61 (brs, 1H), 2.08–1.97 (p, 2H). ^1^H NMR (400 MHz, DMSO-*d*_6_) δ 8.30–8.19 (m, 2H), 7.81–7.60 (m, 7H), 7.50 (dd, *J* = 8.4, 6.8 Hz, 2H), 7.44–7.38 (m, 1H), 7.33 (dd, *J* = 7.8, 1.5 Hz, 1H), 4.43 (brs, 1H), 3.16 (brs, 1H), 2.38 (brs, 1H), 2.00 (brs, 2H). ^13^C NMR (101 MHz, CDCl_3_) δ 150.1, 145.2, 144.6, 136.0, 135.0, 133.9, 132.1, 131.6, 131.1, 130.7, 129.1, 128.4, 128.1, 127.8, 126.9, 124.5, 53.1, 27.8, 21.8. HRMS (ESI pos.) *m*/*z*: [M + H]^+^ calcd for C_23_H_19_N_5_O_4_S, 462.1231; found, 462.1226.

#### Synthesis of 5-((4-Nitrophenyl)sulfonyl)-3-phenyl-4,5-dihydro-[1,2,3]triazolo[1,5-*a*]quinoxalin-9-ol 12e

Compound **10d** (250 mg, 0.535 mmol, 1 equiv) was dissolved in a mixture of 10 mL of ACN and 3 mL of 75% AcOH. The solution was cooled to 0 °C and vigorously stirred on a magnetic stirrer. Subsequently, a solution of 3 mL of NaNO_2_ (74 mg, 1.07 mmol, 2 equiv) was added. The reaction was stirred in an ice bath for 30 min, and then 4 mL of aqueous NaN_3_ solution (139 mg, 2.14 mmol, 4 equiv) was added. The reaction was stirred for an additional 15 min and then quenched with 50 mL of water and extracted into DCM (3 × 50 mL). The combined organic layers were dried using MgSO_4_, filtered, and evaporated to dryness *in vacuo*. The crude product was purified by precipitation in 50% AcOH/ACN (5:2, v/v), followed by filtration under reduced pressure to give a pale pink solid (50 mg, 20% yield). ^1^H NMR (400 MHz, DMSO-*d*_6_) δ 10.44 (s, 1H), 8.04–7.90 (m, 2H), 7.71–7.56 (m, 4H), 7.54–7.47 (m, 1H), 7.43 (t, *J* = 8.2 Hz, 1H), 7.26 (dd, *J* = 8.0, 1.2 Hz, 1H), 7.23–7.11 (m, 3H), 5.25 (s, 2H). ^13^C NMR (101 MHz, DMSO-*d*_6_) δ 149.8, 148.8, 141.2, 140.6, 129.3, 129.2, 128.7, 128.6, 128.6, 127.7, 126.5, 125.9, 124.2, 118.4, 117.9, 117.7, 42.3. HRMS (ESI pos.) *m*/*z*: [M + H]^+^ calcd for C_21_H_15_N_5_O_5_S, 450.1097; found, 450.0866.

#### General Procedures for Desulfonylation (Products **17c–l**)

DBU (2 equiv) was added to a solution of **12a–l** (50–100 mg per mL) and the solution was stirred for 3–5 h at room temperature for (**12a–j**) or at 90 °C (**12k–l**) for 24 h. Subsequently, water was added (three to five times the volume compared to the DMSO used), resulting in precipitation of the products, which were obtained by filtration under reduced pressure.

##### 3-(4-(Trifluoromethyl)phenyl)-[1,2,3]triazolo[1,5-*a*]quinoxaline **17c**

White solid (17 mg, 85%).^1^H NMR (400 MHz, TFA-*d*) δ 9.99 (s, 1H), 8.97 (dd, *J* = 8.5, 1.3 Hz, 1H), 8.39 (dd, *J* = 8.4, 1.3 Hz, 1H), 8.24–8.17 (m, 1H), 8.12–8.04 (m, 3H), 7.94–7.86 (m, 2H). ^13^C NMR (101 MHz, TFA-*d*) δ 153.2, 145.5, 137.7, 137.6 (q, *J* = 33.7 Hz), 135.4, 131.3, 131.2, 129.6, 129.6 (q, *J* = 3,7 Hz), 128.8, 126.0 (q, *J* = 271.5 Hz), 125.9, 125.4, 119.7. HRMS (ESI pos.) *m*/*z*: [M + H]^+^ calcd for C_16_H_9_F_3_N_4_, 315.0.0852; found, 315.0.0838.

##### 3-Phenyl-[1,2,3]triazolo[1,5-*a*]quinoxaline **17d**

Brown solid (17 mg, 95%). ^1^H NMR (400 MHz, DMSO-*d*_6_) δ 9.64 (s, 1H), 8.66 (d, *J* = 8.2 Hz, 1H), 8.33–8.08 (m, 3H), 8.03–7.78 (m, 2H), 7.68–7.43 (m, 3H). ^13^C NMR (101 MHz, DMSO-*d*_6_) δ 144.1, 141.3, 136.0, 130.4, 129.4, 128.9, 126.9, 125.3, 123.2, 115.1, 114.9. HRMS (ESI pos.) *m*/*z*: [M + H]^+^ calcd for C_15_H_10_N_4_, 247.0978; found, 247.0977.

##### 3-Phenyl-[1,2,3]triazolo[1,5-*a*]quinoxalin-9-ol **17e**

Pale brown solid (15 mg, 75%). ^1^H NMR (400 MHz, CDCl_3_) δ 9.40 (s, 1H), 8.09–8.01 (m, 2H), 7.74 (dd, *J* = 8.2, 1.2 Hz, 1H), 7.69–7.58 (m, 3H), 7.56–7.51 (m, 1H), 7.41 (dd, *J* = 8.1, 1.3 Hz, 1H). ^13^C NMR (101 MHz, CDCl_3_) δ 148.4, 141.8, 138.2, 129.8, 129.6, 129.5, 127.6, 123.3, 120.6, 117.5, 114.9. HRMS (ESI pos.) *m*/*z*: [M + H]^+^ calcd for C_15_H_10_N_4_O, 263.0927; found, 263.0923.

##### Methyl 3-Phenyl-[1,2,3]triazolo[1,5-*a*]quinoxaline-7-carboxylate **17f**

White solid (18 mg, 95%). ^1^H NMR (400 MHz, CDCl_3_) δ 9.47 (s, 1H), 8.87 (d, *J* = 1.7 Hz, 1H), 8.79 (d, *J* = 8.5 Hz, 1H), 8.45 (dd, *J* = 8.6, 1.8 Hz, 1H), 8.13–7.97 (m, 2H), 7.63–7.56 (m, 2H), 7.56–7.49 (m, 1H), 4.04 (s, 3H). ^13^C NMR (101 MHz, CDCl_3_) δ 165.8, 144.7, 143.2, 136.4, 132.3, 131.2, 130.8, 129.8, 129.7, 129.6, 129.1, 127.6, 123.8, 116.2, 52.9. HRMS (ESI pos.) *m*/*z*: [M + H]^+^ calcd for C_17_H_12_N_4_O_2_, 305.1033; found, 305.1031.

##### 7-Nitro-3-phenyl-5-tosyl-4,5-dihydro-[1,2,3]triazolo[1,5-*a*]quinoxaline **17g**

Yellow solid (21 mg, 88%). ^1^H NMR (400 MHz, TFA-*d*) δ 9.94 (s, 1H), 9.32–9.20 (m, 1H), 9.13–8.95 (m, 1H), 8.92–8.74 (m, 1H), 7.97–7.88 (m, 2H), 7.68–7.56 (m, 3H). ^13^C NMR (101 MHz, TFA-*d*) δ 152.6, 151.3, 149.1, 135.2, 133.0, 132.6, 132.4, 130.7, 130.0, 127.1, 126.5, 124.1, 121.0. HRMS (ESI pos.) *m*/*z*: [M + H]^+^ calcd for C_15_H_9_N_5_O_2_, 292.0829; found, 292.0826.

##### 7,8-Dimethyl-3-phenyl-[1,2,3]triazolo[1,5-*a*]quinoxaline **17h**

Beige solid (18 mg, 85%). ^1^H NMR (400 MHz, CDCl_3_) δ 9.35 (s, 1H), 8.51 (s, 1H), 8.17–8.01 (m, 2H), 7.94 (s, 1H), 7.61–7.55 (m, 2H), 7.52–7.46 (m, 1H), 2.57 (s, 3H), 2.50 (s, 3H). ^13^C NMR (101 MHz, CDCl_3_) δ 142.5, 142.4, 141.1, 138.7, 135.2, 130.4, 130.0, 129.4, 129.2, 127.5, 124.4, 123.6, 115.9, 20.6, 20.1. HRMS (ESI pos.) *m*/*z*: [M + H]^+^ calcd for C_17_H_14_N_4_, 275.1291; found, 275.1289.

##### 8-Methoxy-3-phenyl-[1,2,3]triazolo[1,5-*a*]quinoxaline **17i**

Pink solid (34 mg, 90%). ^1^H NMR (400 MHz, CDCl_3_) δ 9.30 (s, 1H), 8.15–8.01 (m, 4H), 7.62–7.55 (m, 2H), 7.52–7.46 (m, 1H), 7.33 (dd, *J* = 9.1, 2.8 Hz, 1H), 4.06 (s, 3H). ^13^C NMR (101 MHz, CDCl_3_) δ 161.4, 142.2, 140.5, 131.5, 131.3, 130.2, 129.4, 129.2, 127.4, 127.3, 123.7, 118.9, 97.3, 56.4. HRMS (ESI pos.) *m*/*z*: [M + H]^+^ calcd for C_16_H_12_N_4_O, 277.1084; found, 277.1076.

##### 8-Chloro-3-phenyl-[1,2,3]triazolo[1,5-*a*]quinoxaline **17j**

White solid (35 mg, 92%). ^1^H NMR (400 MHz, CDCl_3_) δ 9.40 (s, 1H), 8.74 (d, *J* = 2.2 Hz, 1H), 8.13 (d, *J* = 8.7 Hz, 1H), 8.08–8.01 (m, 2H), 7.71 (dd, *J* = 8.8, 2.3 Hz, 1H), 7.66–7.55 (m, 2H), 7.55–7.45 (m, 1H). ^13^C NMR (101 MHz, CDCl_3_) δ 143.8, 143.7, 137.2, 134.5, 130.9, 130.1, 129.9, 129.6, 129.4, 127.7, 126.9, 123.6, 116.1. HRMS (ESI pos.) *m*/*z*: [M + H]^+^ calcd for C_15_H_9_ClN_4_, 281.0589; found, 281.0586.

##### [1,2,3]Triazolo[1,5-*a*]quinoxaline **17k**

White solid (100 mg, 67%). ^1^H NMR (400 MHz, CDCl_3_) δ 9.23 (s, 1H), 8.83–8.62 (m, 1H), 8.40 (s, 1H), 8.19 (dd, *J* = 8.1, 1.5 Hz, 1H), 7.97–7.64 (m, 2H). ^13^C NMR (101 MHz, CDCl_3_) δ 143.3, 136.6, 130.7, 130.4, 129.5, 129.0, 127.1, 126.3, 116.0. HRMS (ESI pos.) *m*/*z*: [M + H]^+^ calcd for C_9_H_6_N_4_, 171.0665; found, 171.0664.

##### 3-Methyl-[1,2,3]triazolo[1,5-*a*]quinoxaline **17l**

Beige solid (45 mg, 92%). ^1^H NMR (400 MHz, DMSO-*d*_6_) δ 9.43 (s, 1H), 8.64–8.56 (m, 1H), 8.20–8.09 (m, 1H), 7.91–7.85 (m, 1H), 7.84–7.79 (m, 1H), 2.71 (s, 3H). ^13^C NMR (101 MHz, DMSO-*d*_6_) δ 144.4, 139.2, 135.9, 130.2, 129.8, 128.8, 125.5, 124.6, 115.0, 10.0. HRMS (ESI pos.) *m*/*z*: [M + H]^+^ calcd for C_10_H_8_N_4_, 185.0822; found, 185.0819.

#### General Synthesis Procedure for Denosylation with Thiolate (Products **16a–16c**, **18**, and **19**)

Compounds **12b**, **12m**, **12n**, **15a**, and **15b** (1 equiv) were dissolved in DMSO. The reaction vessel was closed with a lid with a rubber stopper and the solution was subsequently bubbled with N_2_ for at least 2 min. An equimolar solution of DBU and mercaptoethanol in DMSO (2 equiv) was then added to the solution via a needle. After the addition, the mixture was stirred for 30 min. The end of the reaction was indicated by LCMS. The reaction mixture was quenched with water to precipitate products **16a–c**, which were obtained by filtering under reduced pressure and washing the filter several times with water. To obtain substances **18** and **19**, the reaction was quenched with 50 mL of water and extracted with 3 × 50 mL of DCM. The combined organic layers were dried with MgSO_4_, filtered, and evaporated to dryness *in vacuo*. The crude product was subsequently purified by silica gel chromatography using Hex/EtOAc (3:1–1:1, v/v).

##### 3-Ethyl-4-methyl-4,5-dihydro-[1,2,3]triazolo[1,5-*a*]quinoxaline **16a**

White solid (190 mg, 53%). ^1^H NMR (400 MHz, CDCl_3_) δ 8.01 (dd, *J* = 8.0, 1.4 Hz, 1H), 7.12 (ddd, *J* = 8.1, 7.5, 1.5 Hz, 1H), 6.88 (ddd, *J* = 8.0, 7.5, 1.2 Hz, 1H), 6.76 (dd, *J* = 8.0, 1.2 Hz, 1H), 4.90 (q, *J* = 6.6 Hz, 1H), 3.99 (s, 1H), 2.75 (q, *J* = 7.6 Hz, 2H), 1.50 (d, *J* = 6.6 Hz, 3H), 1.33 (t, *J* = 7.6 Hz, 3H). ^13^C NMR (101 MHz, CDCl_3_) δ 143.5, 134.6, 128.8, 128.3, 122.3, 119.4, 116.7, 115.3, 45.7, 22.5, 18.7, 14.1. HRMS (ESI pos.) *m*/*z*: [M + H]^+^ calcd for C_12_H_14_N_4_, 215.1291; found, 215.1290.

##### 4-Methyl-3-phenyl-4,5-dihydro-[1,2,3]triazolo[1,5-*a*]quinoxaline **16b**

White solid (115 mg, 98%). ^1^H NMR (400 MHz, CDCl_3_) δ 8.09 (dd, *J* = 8.0, 1.4 Hz, 1H), 7.84–7.71 (m, 2H), 7.53–7.43 (m, 2H), 7.42–7.34 (m, 1H), 7.17 (td, *J* = 7.7, 1.4 Hz, 1H), 6.93 (td, *J* = 7.8, 1.2 Hz, 1H), 6.82 (dd, *J* = 8.1, 1.2 Hz, 1H), 5.22 (qd, *J* = 6.6, 2.1 Hz, 1H), 4.10 (s, 1H), 1.48 (d, *J* = 6.6 Hz, 3H). HRMS (ESI pos.) *m*/*z*: [M + H]^+^ calculated for C_16_H_14_N_4_, 263.1291; found, 263.1289.

##### 3-(4-(Trifluoromethyl)phenyl)-4,5-dihydro-[1,2,3]triazolo[1,5-*a*]quinoxaline **16c**

White solid (82 mg, 91%). ^1^H NMR (400 MHz, DMSO-*d*_6_) δ 8.04–7.91 (m, 2H), 7.85 (dt, *J* = 8.2, 1.8 Hz, 3H), 7.16 (ddd, *J* = 8.2, 7.3, 1.5 Hz, 1H), 6.91 (dd, *J* = 8.2, 1.2 Hz, 1H), 6.86–6.74 (m, 1H), 6.61 (d, *J* = 1.9 Hz, 1H), 4.91 (d, *J* = 1.7 Hz, 2H).^13^C NMR (101 MHz, DMSO-*d*_6_) δ 139.8, 137.2, 134.3, 128.8, 128.0 (q, *J* = 31.9 Hz), 126.8, 126.7, 126.9 (q, J = 3,7 Hz), 124.2 (q, *J* = 272 Hz), 120.9, 117.6, 115.8, 115.0, 38.5. HRMS (ESI pos.) *m*/*z*: [M + H]^+^ calcd for C_16_H_11_F_3_N_4_, 317.1009; found, 317.1001.

##### 3-Phenyl-5,6-dihydro-4*H*-benzo[*b* ][1,2,3]triazolo[1,5-*d*][1,4]diazepine **18**

Orange solid (90 mg, 62%). ^1^H NMR (400 MHz, CDCl_3_) δ 8.09 (dd, *J* = 8.2, 1.5 Hz, 1H), 7.78–7.67 (m, 2H), 7.52–7.43 (m, 2H), 7.43–7.34 (m, 1H), 7.23 (ddd, *J* = 8.0, 7.2, 1.5 Hz, 1H), 7.01 (ddd, *J* = 8.4, 7.3, 1.4 Hz, 1H), 6.85 (dd, *J* = 8.1, 1.4 Hz, 1H), 4.07 (s, 1H), 3.70 (dd, *J* = 6.2, 5.1 Hz, 2H), 3.25 (dd, *J* = 6.3, 5.1 Hz, 2H). ^13^C NMR (101 MHz, CDCl_3_) δ 144.3, 138.4, 132.9, 131.4, 129.4, 128.9, 128.0, 127.7, 126.0, 124.6, 120.8, 120.7, 48.1, 24.1. HRMS (ESI pos.) *m*/*z*: [M + H]^+^ calcd for C_16_H_14_N_4_, 263.1291; found, 263.1288.

##### 3-Phenyl-4,5,6,7-tetrahydrobenzo[*b*][1,2,3]triazolo[1,5-*d*][1,4]diazocine **19**

Beige solid (50 mg, 33%). ^1^H NMR (400 MHz, CDCl_3_) δ 7.87–7.74 (m, 2H), 7.51–7.42 (m, 2H), 7.42–7.32 (m, 2H), 7.19 (ddd, *J* = 8.5, 7.1, 1.6 Hz, 1H), 6.78 (ddd, *J* = 8.3, 7.2, 1.3 Hz, 1H), 6.68 (dd, *J* = 8.3, 1.3 Hz, 1H), 4.11 (t, *J* = 6.7 Hz, 1H), 3.18 (t, *J* = 6.6 Hz, 2H), 3.08 (m, *J* = 5.8 Hz, 2H), 1.84 (brs, 2H). ^13^C NMR (101 MHz, CDCl_3_) 143.4, 142.5, 133.7, 131.6, 130.2, 129.9, 128.9, 127.9, 126.8, 120.5, 118.6, 118.0, 41.1, 28.3, 20.1. HRMS (ESI pos.) *m*/*z*: [M + H]^+^ calcd for C_17_ H_16_N_4_, 277.1448; found, 277.1446.

#### General Procedure for Ring Oxidation (Products **17a** and **17b**)

**16a** or **16b** (1 equiv) was dissolved in toluene and 5 equiv of MnO_2_ was added. The mixture was heated to 80 °C for 16 h. The end of the reaction was indicated by TLC with visualization by a KMnO_4_ solution. The mixture was filtered and evaporated to dryness *in vacuo*.

##### 3-Ethyl-4-methyl-[1,2,3]triazolo[1,5-*a*]quinoxaline **17a**

White solid (92%). ^1^H NMR (500 MHz, CDCl_3_) δ 8.70–8.52 (m, 1H), 8.14–7.89 (m, 1H), 7.75–7.56 (m, 2H), 3.24 (q, *J* = 7.6 Hz, 2H), 2.93 (s, 3H), 1.50 (t, *J* = 7.6 Hz, 3H). ^13^C NMR (101 MHz, CDCl_3_) δ 152.6, 145.0, 136.3, 129.0, 129.0, 128.5, 125.6, 124.3, 115.4, 23.5, 20.3, 14.6. HRMS (ESI pos.) *m*/*z*: [M + H]^+^ calcd for C_12_H_12_N_4_, 213.1135; found, 213.1133.

##### 4-Methyl-3-phenyl-[1,2,3]triazolo[1,5-*a*]quinoxaline **17b**

White solid (98%). ^1^H NMR (500 MHz, CDCl_3_) δ 8.78–8.64 (m, 1H), 8.11–8.03 (m, 1H), 7.77–7.69 (m, 2H), 7.69–7.65 (m, 2H), 7.57–7.52 (m, 3H), 2.68 (s, 3H). ^13^C NMR (101 MHz, CDCl_3_) δ 153.0, 143.9, 136.5, 130.8, 130.6, 129.4, 129.3, 129.2, 128.9, 128.6, 125.6, 124.3, 115.7, 24.3. HRMS (ESI pos.) *m*/*z*: [M + H] ^+^ calculated for C_16_H_12_N_4_, 261.1135; found, 261.1132.

#### Synthesis of 5-Methyl-3-(4-(trifluoromethyl)phenyl)-4,5-dihydro-[1,2,3]triazolo[1,5-*a*]quinoxaline **20**

Compound **16c** (31 mg, 0.1 mmol) was dissolved in 1 mL of DMSO, and DBU (40 μL, 0.27 mmol, 2.7 equiv) and MeI (60 μL, 0.96 mmol, 9.6 equiv) were added. The reaction was heated to 50 °C overnight. The next day, 3 mL of water was added to precipitate a brown powder. The crude product was purified by silica gel chromatography using Hex:EtOAc (7:3, v/v) to obtain compound **20** as a white solid (26 mg, 80%). ^1^H NMR (400 MHz, CDCl_3_) δ 8.07 (dd, *J* = 7.9, 1.5 Hz, 1H), 7.86–7.77 (m, 2H), 7.75–7.67 (m, 2H), 7.27 (ddd, *J* = 8.2, 7.5, 1.5 Hz, 1H), 6.98–6.90 (m, 1H), 6.80 (dd, *J* = 8.3, 1.2 Hz, 1H), 4.67 (s, 2H), 3.00 (s, 3H). HRMS (ESI pos.) *m*/*z*: [M + H]^+^ calcd for C_17_H_13_F_3_N_4_, 331.1165; found, 331.1164.

#### Synthesis of 1-(3-(4-(Trifluoromethyl)phenyl)-[1,2,3]triazolo[1,5-*a*]quinoxalin-5(4H)-yl)ethan-1-one **21**

Compound **16c** (20 mg, 0.064 mmol) was dissolved in 0.5 mL of DMSO, and DBU (40 μL, 0.26 mmol, 4 equiv) and Ac_2_O (40 μL, 0.42 mmol, 6.5 equiv) were added to the solution. The reaction mixture was heated to 50 °C for 3 h. Subsequently, 1 mL of water was added to precipitate the product. Compound **21** was obtained by filtration under reduced pressure and washing with 10 mL of water as a beige solid (22 mg, 96%). ^1^H NMR (400 MHz, CDCl_3_) δ 8.33–8.14 (m, 1H), 7.91 (d, *J* = 8.0 Hz, 2H), 7.76 (d, *J* = 8.0 Hz, 2H), 7.62–7.31 (m, 3H), 5.38 (s, 2H), 2.27 (s, 3H). ^13^C NMR (101 MHz, CDCl_3_) δ 169.71, 141.65, 133.63, 130.5 (q, *J* = 32.6 Hz), 129.00, 128.17, 128.13, 128.04, 127.63, 127.07, 126.21 (q, *J* = 3.8 Hz), 125.11, 124.2 (q, *J* = 272 Hz), 118.29, 38.28, 22.31. HRMS (ESI pos.) *m*/*z*: [M + H]^+^ calcd for C_18_H_13_F_3_N_4_O, 359.1114; found, 359.1109.

#### General Procedure for Methyl Oxidation (Products **22** and **23**)

Compound **17a** or **17b** was dissolved in THF, and then 1.5 equiv of SeO_2_ was added. The mixture was refluxed for 3 h and monitored by LCMS. Later, the reaction mixture was evaporated to dryness *in vacuo* and subsequently poured with 50 mL of water and extracted with 3 × 50 mL of EtOAc. The combined organic layers were dried over MgSO_4_, filtered, and evaporated to dryness *in vacuo*. The crude product was purified by silica gel chromatography using Hex:EtOAc (7:3, v/v).

##### 3-Ethyl-[1,2,3]triazolo[1,5-*a*]quinoxaline-4-carbaldehyde **22**

Orange solid (52 mg, 98% yield). ^1^H NMR (500 MHz, CDCl_3_) δ 10.12 (s, 1H), 8.74 (ddd, *J* = 8.3, 1.4, 0.5 Hz, 1H), 8.27 (ddd, *J* = 8.2, 1.4, 0.5 Hz, 1H), 7.91 (ddd, *J* = 8.4, 7.3, 1.4 Hz, 1H), 7.80 (ddd, *J* = 8.2, 7.3, 1.4 Hz, 1H), 3.48 (q, *J* = 7.5 Hz, 2H), 1.44 (t, *J* = 7.5 Hz, 3H). ^13^C NMR (101 MHz, CDCl_3_) δ 191.1, 148.3, 146.9, 135.6, 133.2, 131.1, 129.2, 126.9, 121.6, 116.1, 21.4, 15.0. HRMS (ESI pos.) *m*/*z*: [M + H]^+^ calcd for C_12_H_10_N_4_O, 227.0927; found, 227.0924.

##### 3-Phenyl-[1,2,3]triazolo[1,5-*a*]quinoxaline-4-carbaldehyde **23**

Yellow solid (21 mg, 75% yield). ^1^H NMR (400 MHz, DMSO-*d*_6_) δ 9.99 (s, 1H), 8.78 (dd, *J* = 8.3, 1.3 Hz, 1H), 8.39 (dd, *J* = 8.2, 1.3 Hz, 1H), 8.09 (ddd, *J* = 8.4, 7.3, 1.4 Hz, 1H), 7.97 (ddd, *J* = 8.4, 7.3, 1.4 Hz, 1H), 7.77–7.64 (m, 2H), 7.60–7.43 (m, 3H). ^13^C NMR spectra were not recorded due to poor solubility. HRMS (ESI pos.) *m*/*z*: [M + H]^+^ calcd for C_16_H_10_N_4_O, 275.0927; found, 275.0926.

#### Synthesis of 1-(3-Ethyl-[1,2,3]triazolo[1,5-*a*]quinoxalin-4-yl)*-N-*phenylmethanimine **24**

Compound **20** (5 mg, 0.027 mmol) was dissolved in 0.5 mL of dry EtOH, and aniline (5 μL, 0.053 mmol, 2 equiv) was added. The reaction mixture was heated to 70 °C for 2 h. Later, 200 μL of water was added and the solution was allowed to crystallize. The product was obtained as a yellow crystalline solid (3.5 mg, 43% yield). ^1^H NMR (400 MHz, CDCl_3_) δ 8.80 (s, 1H), 8.77–8.69 (m, 1H), 8.16 (dd, *J* = 8.1, 1.3 Hz, 1H), 7.89–7.67 (m, 2H), 7.55–7.44 (m, 2H), 7.43–7.31 (m, 3H), 3.58 (q, *J* = 7.5 Hz, 2H), 1.44 (t, *J* = 7.5 Hz, 3H). HRMS (ESI pos.) *m*/*z*: [M + H]^+^ calcd for C_18_H_15_N_5_, 302.1400; found, 302.1400.

## Supporting Information Available

^1^H and ^13^C spectra of the synthesized compounds (PDF)
