# Supplementary material for: Metal-Free Synthesis of Polysubstituted Triazoloquinoxalines Using Alkynols as the Key Building Blocks
Source: ACS Omega. 2024 Sep 5;9(37):38569–82. doi: 10.1021/acsomega.4c03979 (PMC11411547; doi:10.1021/acsomega.4c03979)

Supporting Information

Metal-free Synthesis of Polysubstituted Triazoloquinoxalines Using Alkynols as the Key Building Blocks

Berenika Masaryk, Miroslav Soural*

[a] *Palacký University, Faculty of Science, Department of Organic Chemistry, 17. listopadu 12, 771 46 Olomouc, Czech Republic*

Corresponding author:
miroslav.soural@upol.cz

Content

1. Copies of NMR spectra of sulfonamides and their precursors (products 2, 5a, 6a, 8a-b, 9a-h)....................2

2. Copies of NMR spectra of *N*-alkylation products (3a-c, 10a-n, 13a-b)..............S16

3. Copies of NMR spectra of azidation products (11a-m, 14a-b)...........................S34

4. Copies of NMR spectra of products 12a-n and 15a-b S40

5. Copies of NMR spectra of products 17a-l, 16a-c, 18, 19, 20, 21, 22, 23, 24.......S56

6. LC-UV-MS traces after heating 15a with MnO_2 (toluene, 110 ^oC) for 6 h……….S77

1. Copies of NMR spectra of sulfonamides and their precursors (products 2, 5a, 6a, 8a-b, 9a-h)

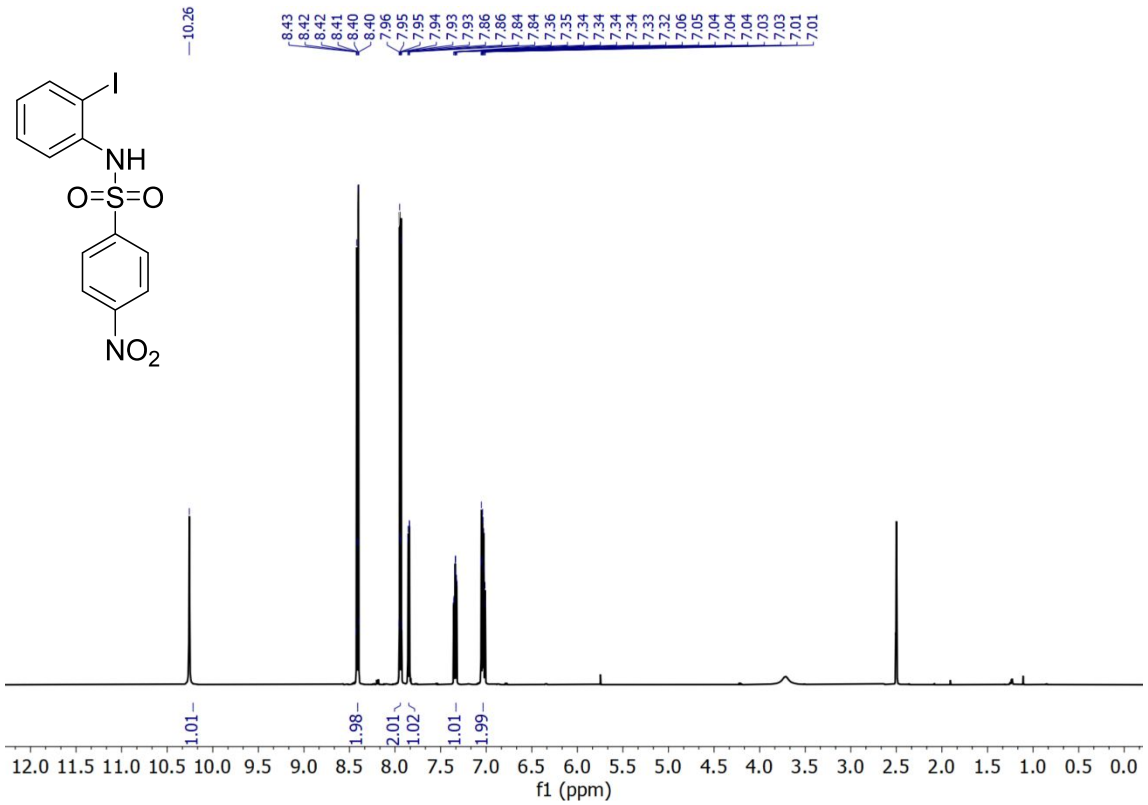

^{1}H NMR spectrum of **2** (500 MHz, DMSO-d_6)

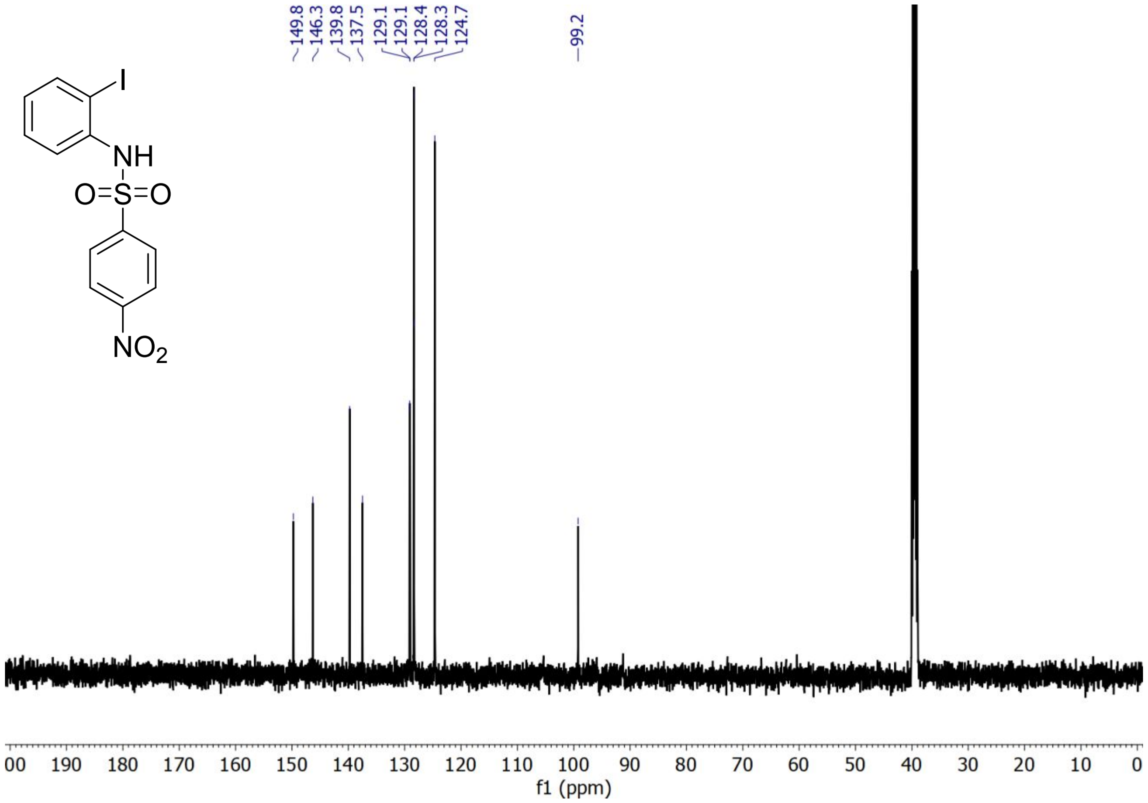

^{13}C NMR spectrum of **2** (126 MHz, DMSO-d_6)

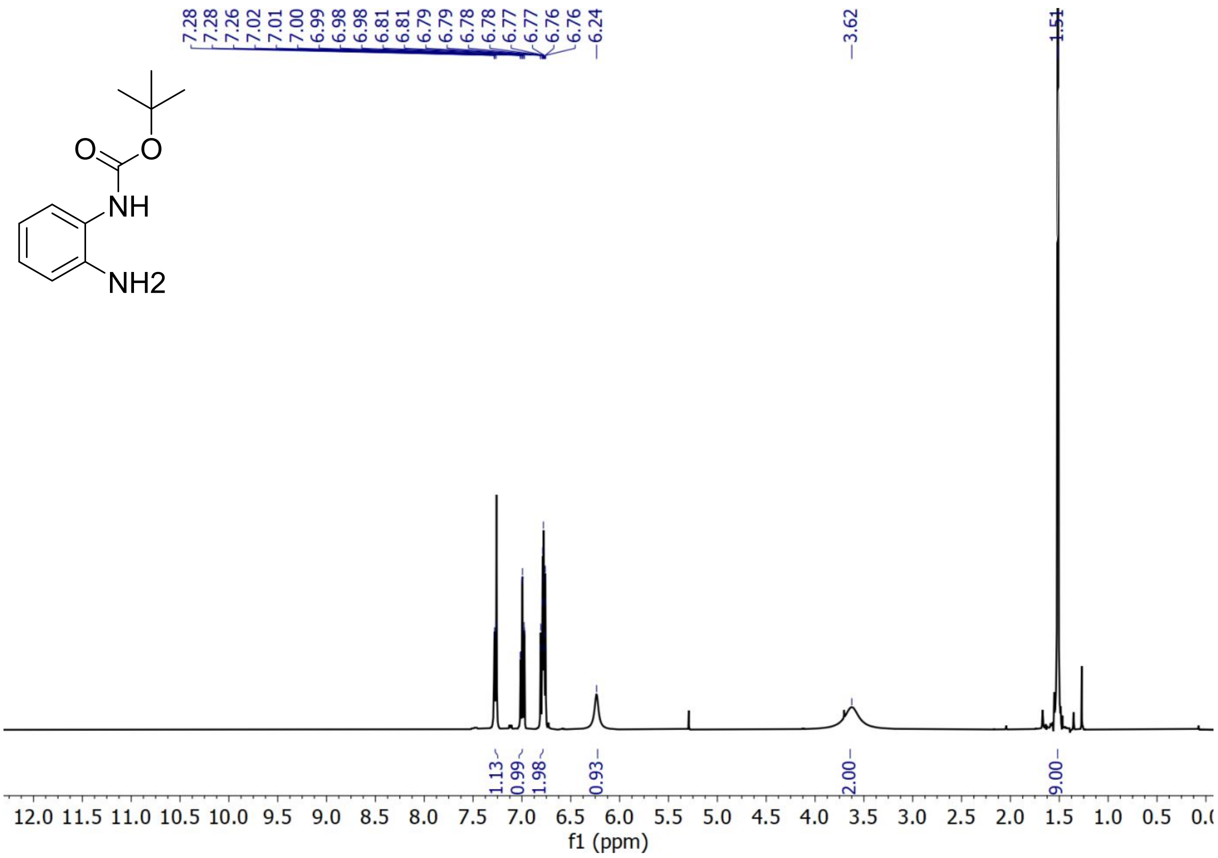

^{1}H NMR spectrum of **5a** (400 MHz, $CDCl_3$)

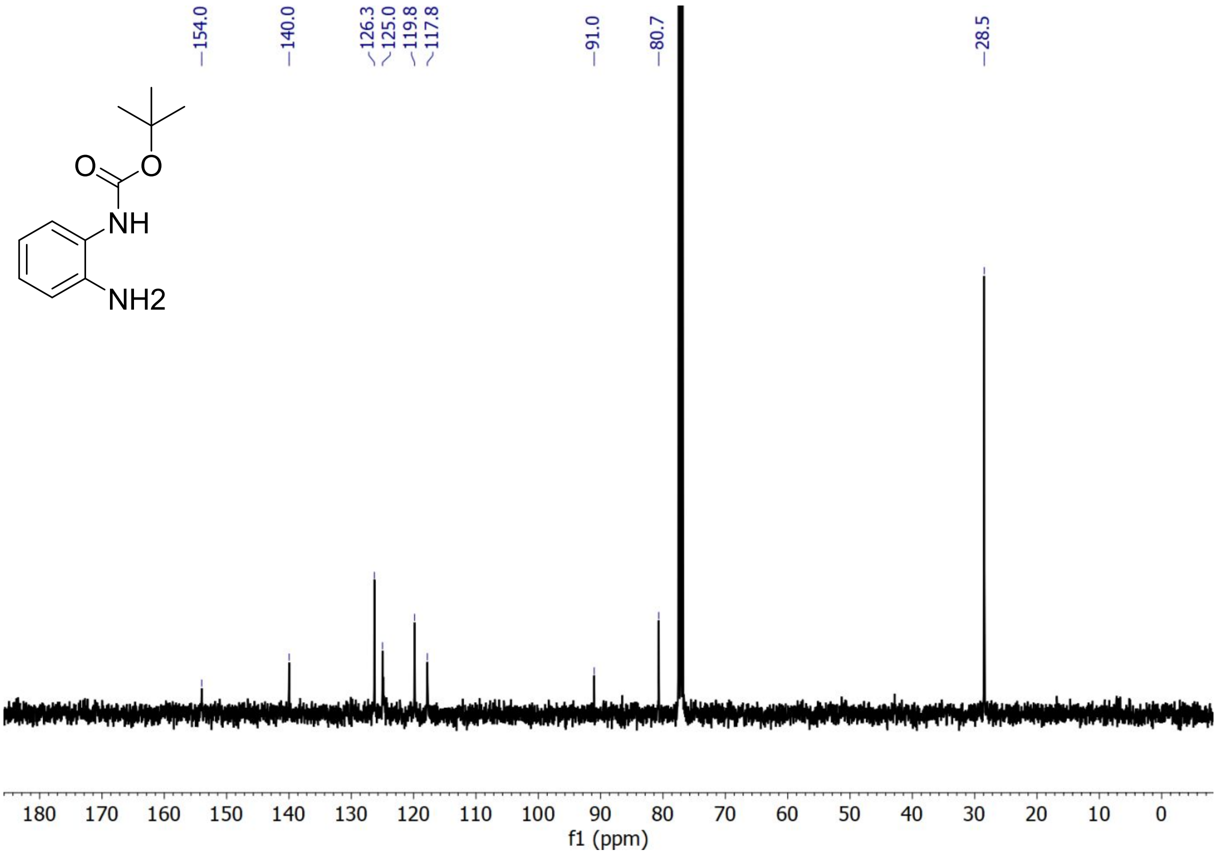

^{13}C NMR spectrum of **5a** (101 MHz, $CDCl_3$)

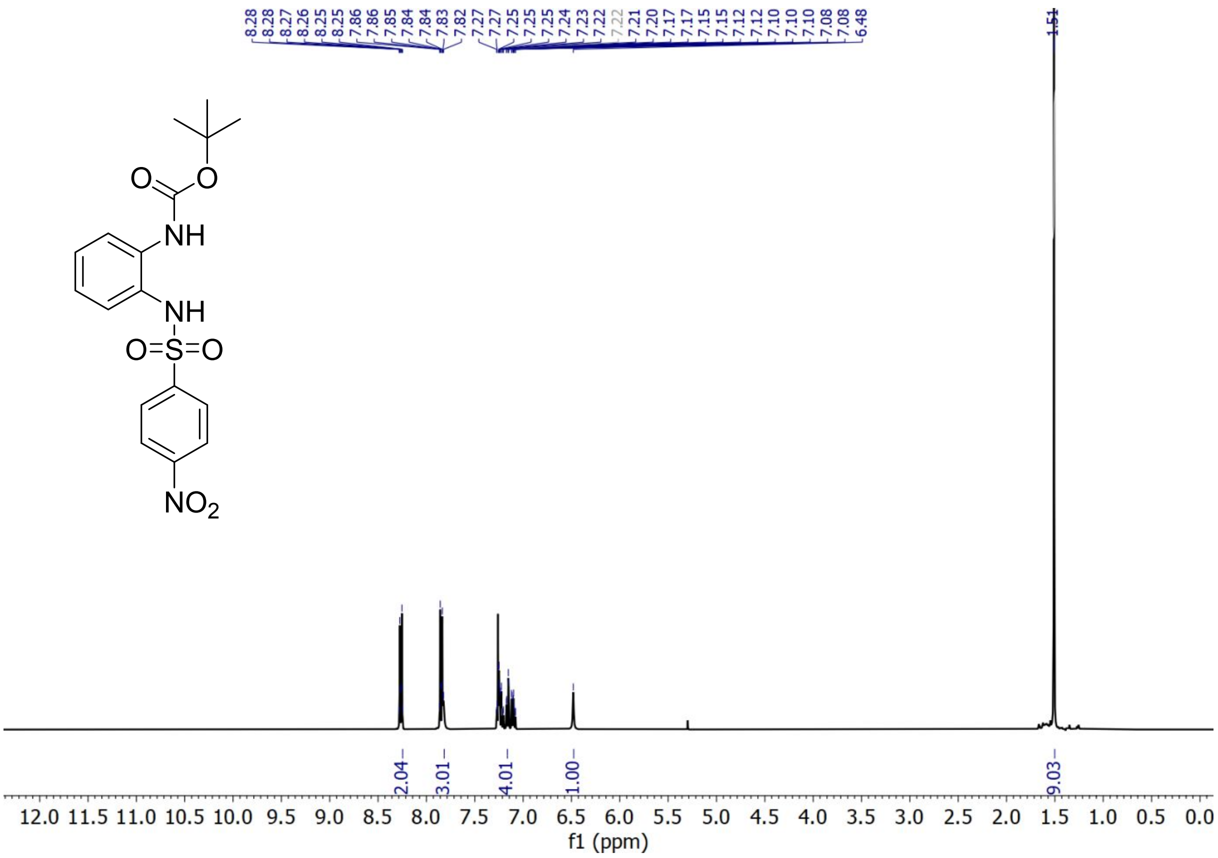

^{1}H NMR spectrum of **6a** (400 MHz, $CDCl_3$)

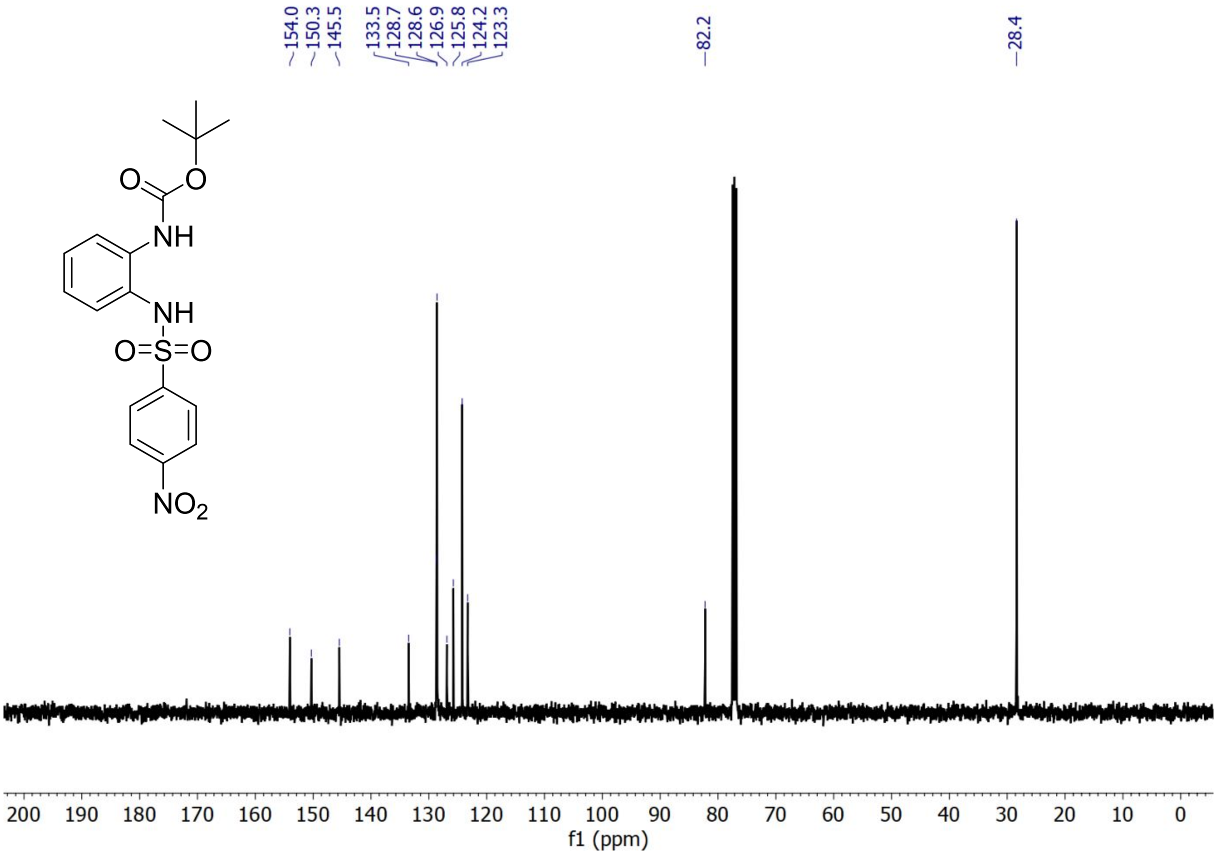

^{13}C NMR spectrum of **6a** (101 MHz, $CDCl_3$)

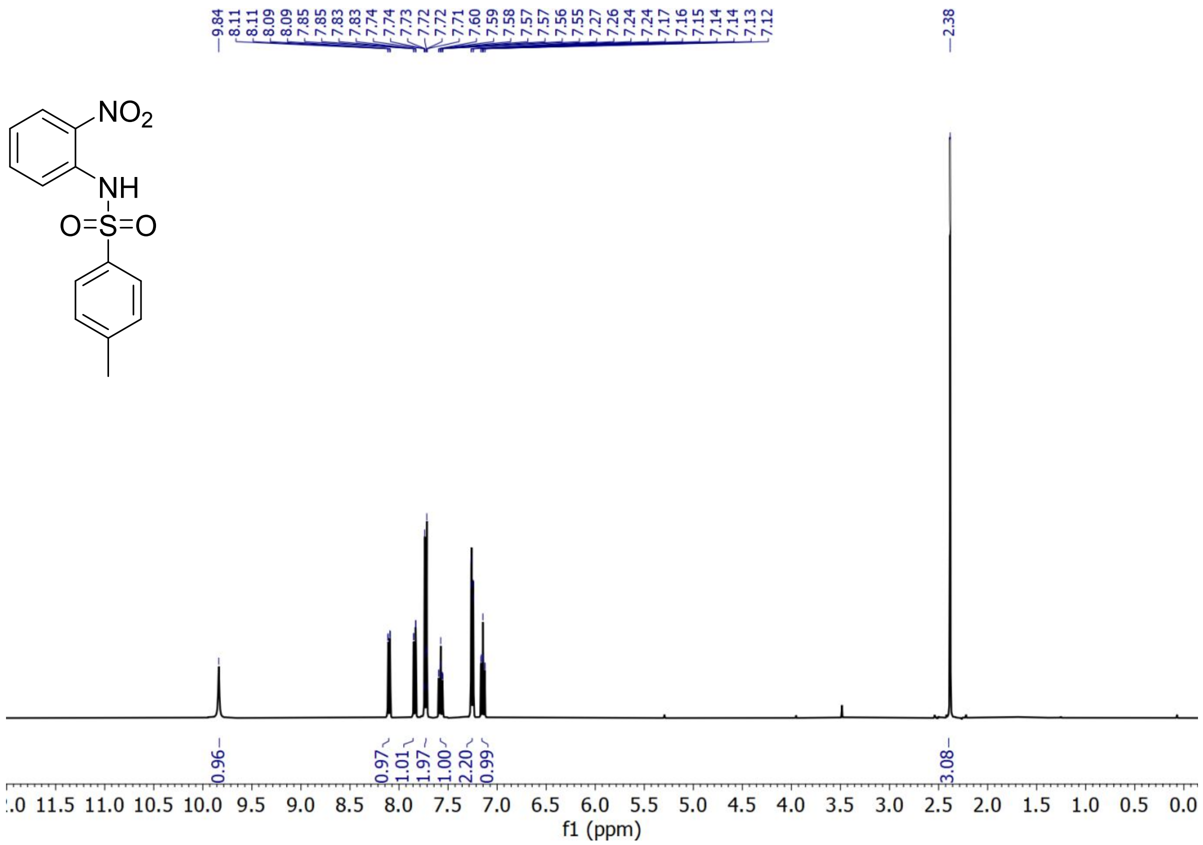

^{1}H NMR spectrum of **8a** (400 MHz, $CDCl_3$)

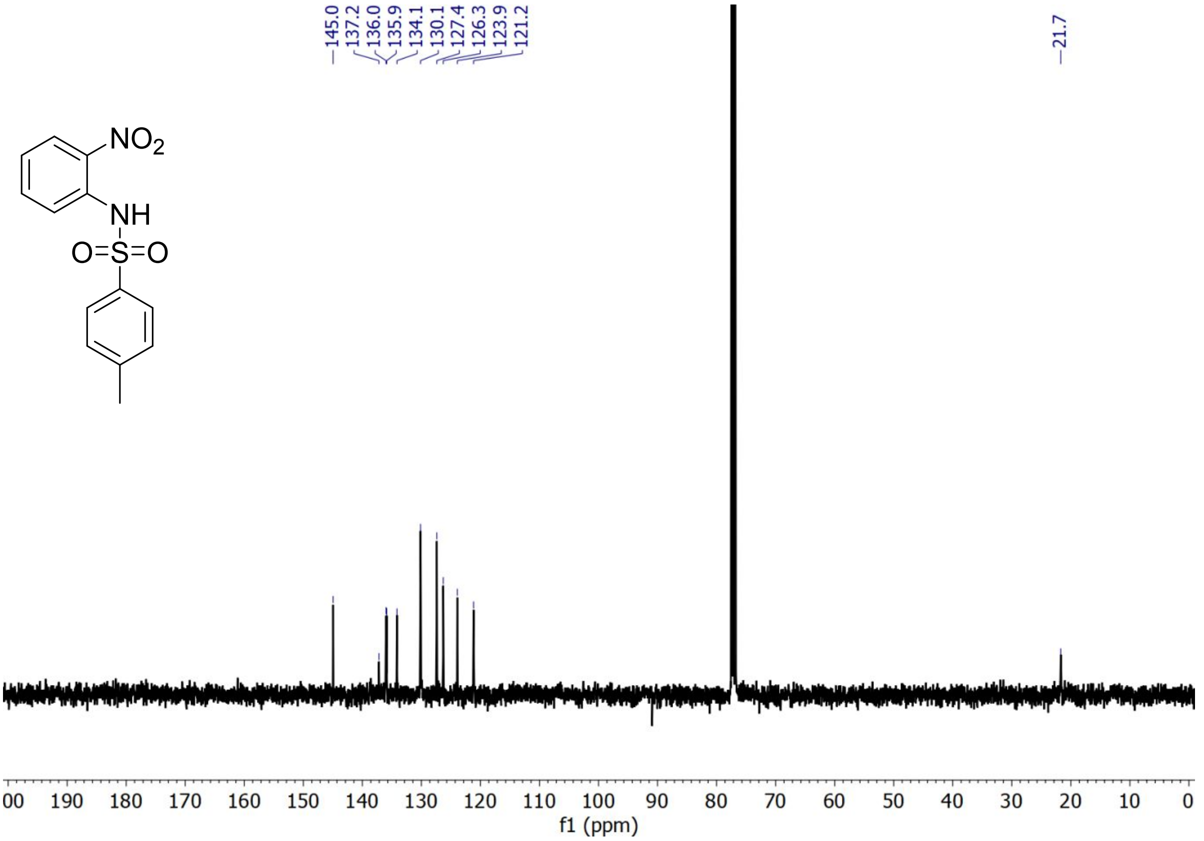

^{13}C NMR spectrum of **8a** (101 MHz, $CDCl_3$)

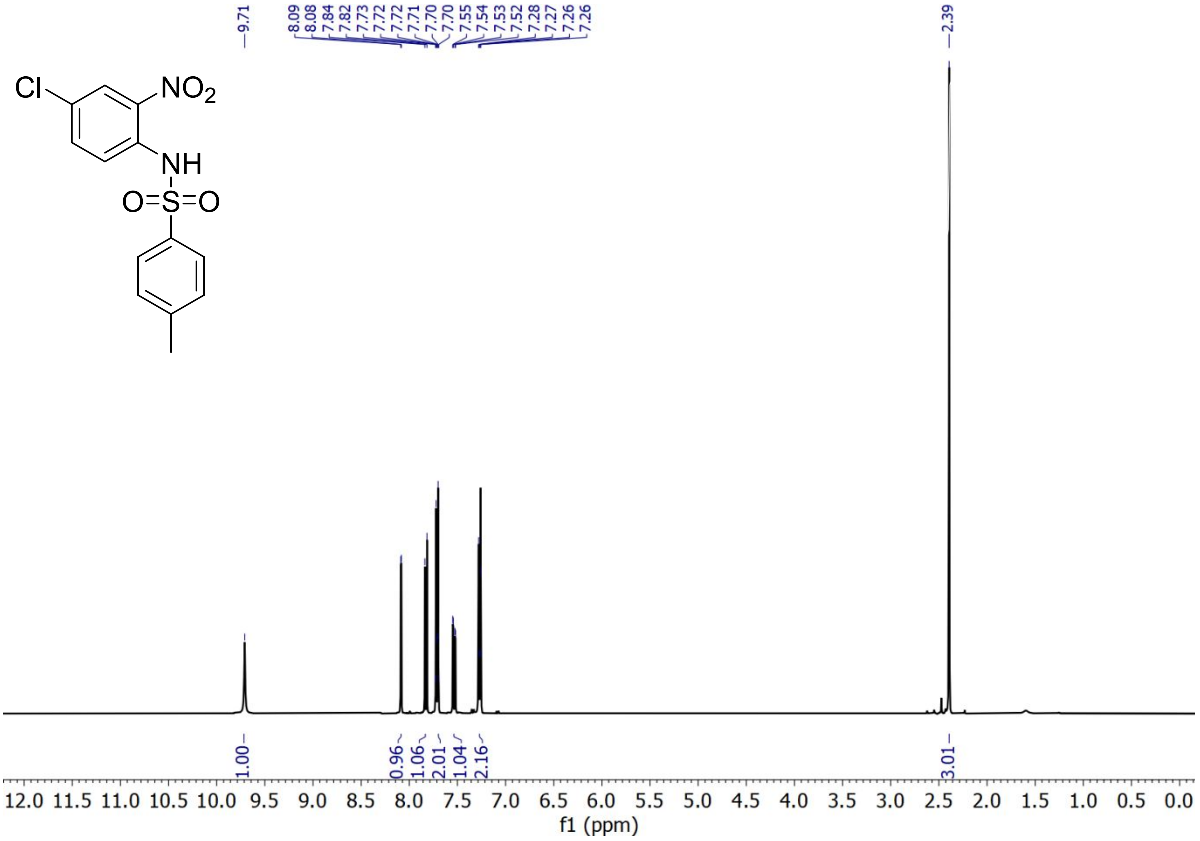

^{1}H NMR spectrum of **8b** (400 MHz, $CDCl_3$)

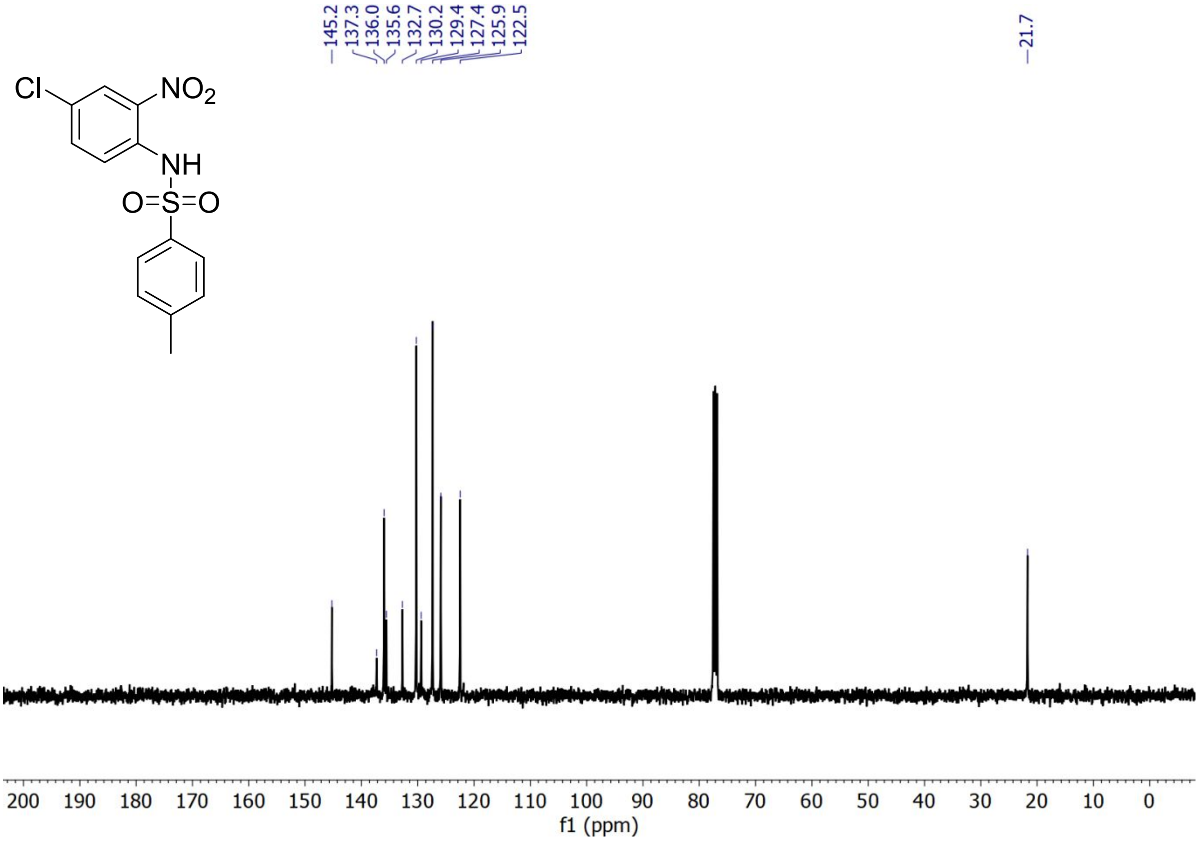

^{13}C NMR spectrum of **8b** (101 MHz, $CDCl_3$)

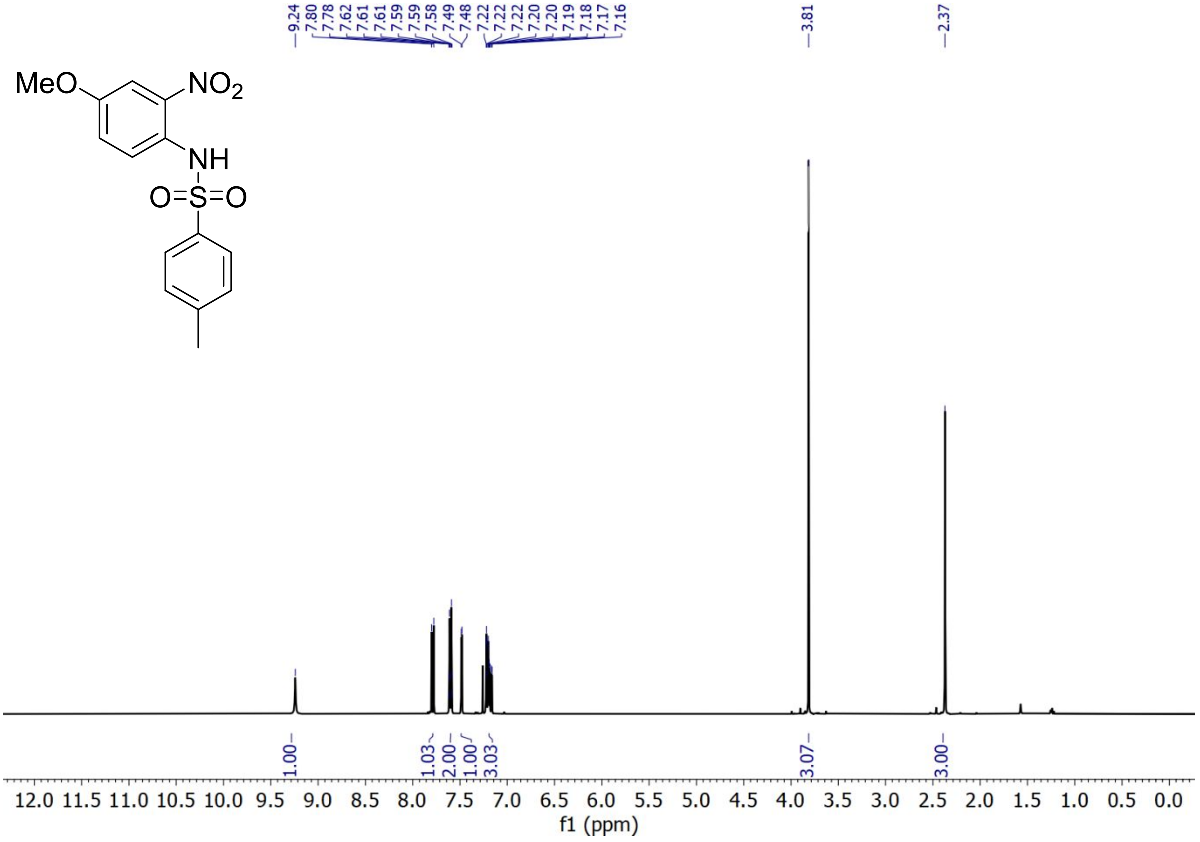

^{1}H NMR spectrum of **8c** (400 MHz, $CDCl_3$)

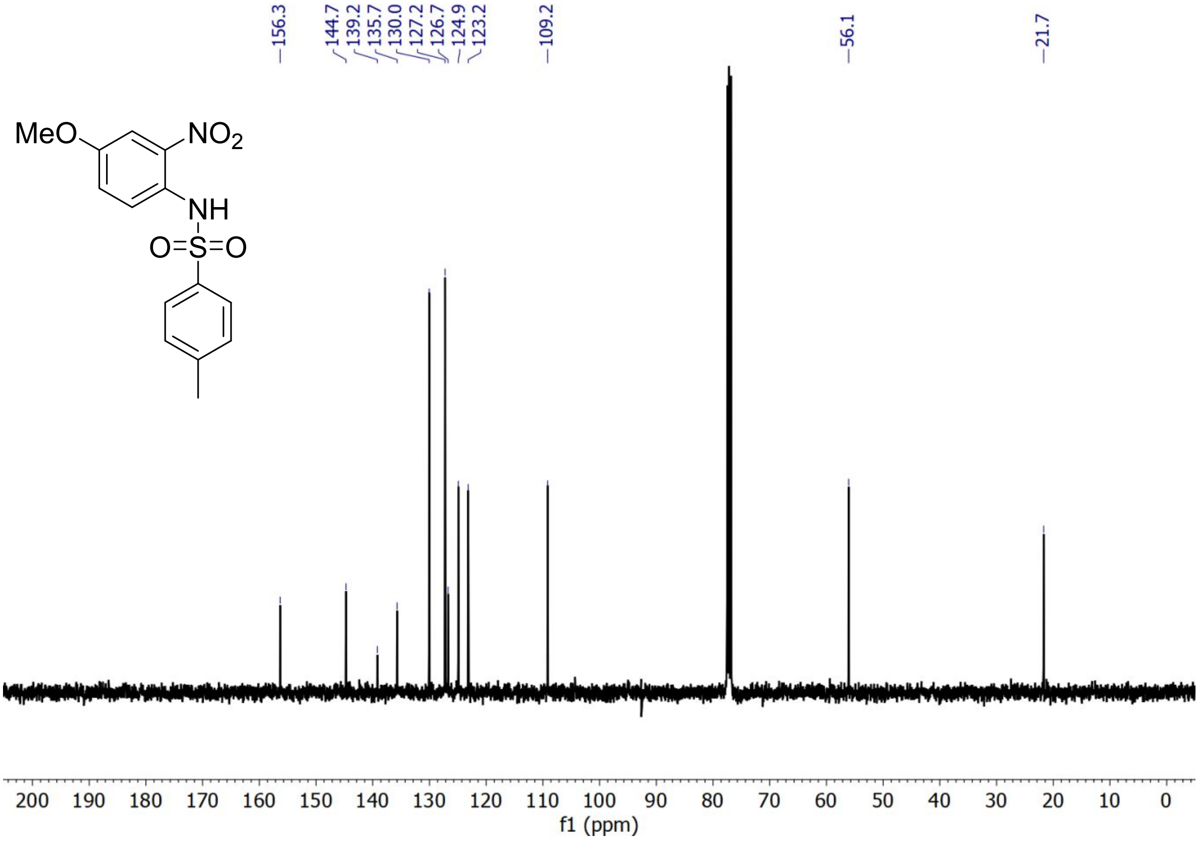

^{13}C NMR spectrum of **8c** (101 MHz, $CDCl_3$)

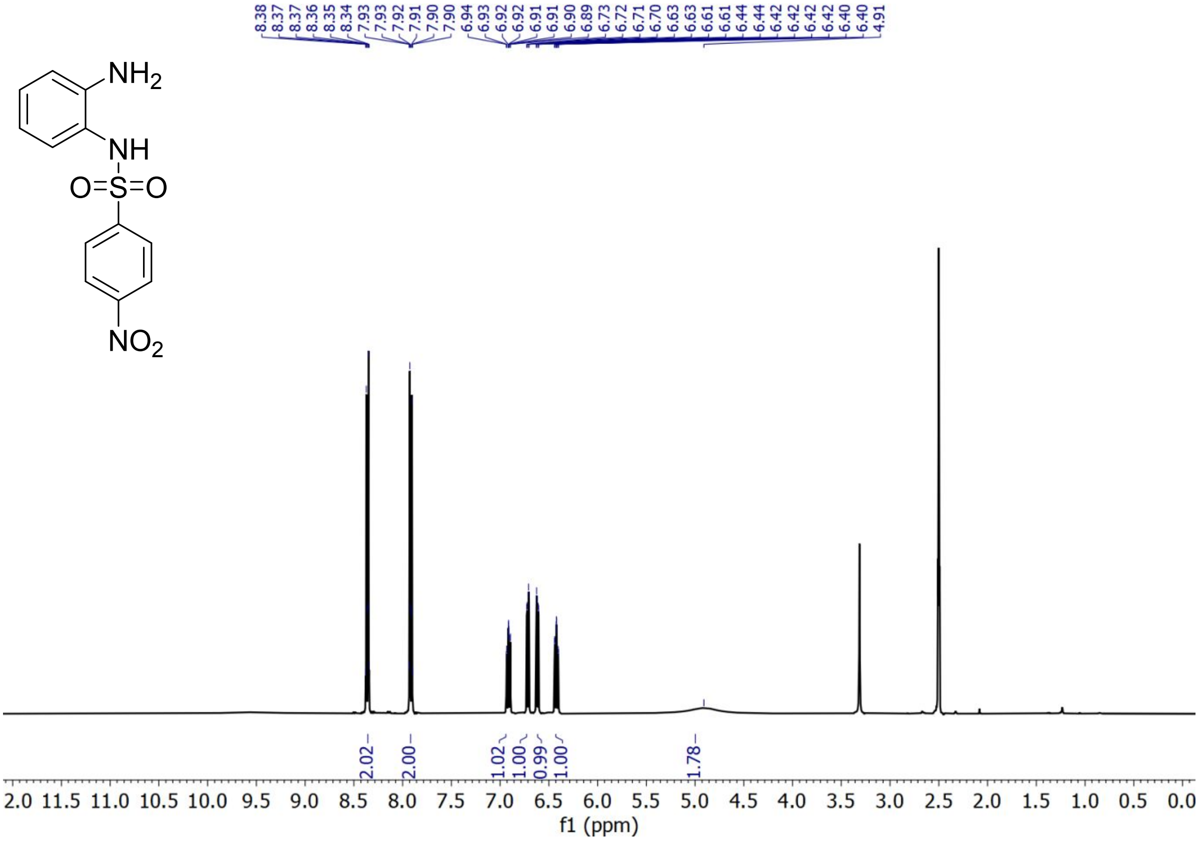

^{1}H NMR spectrum of **9a** (400 MHz, DMSO-d_6)

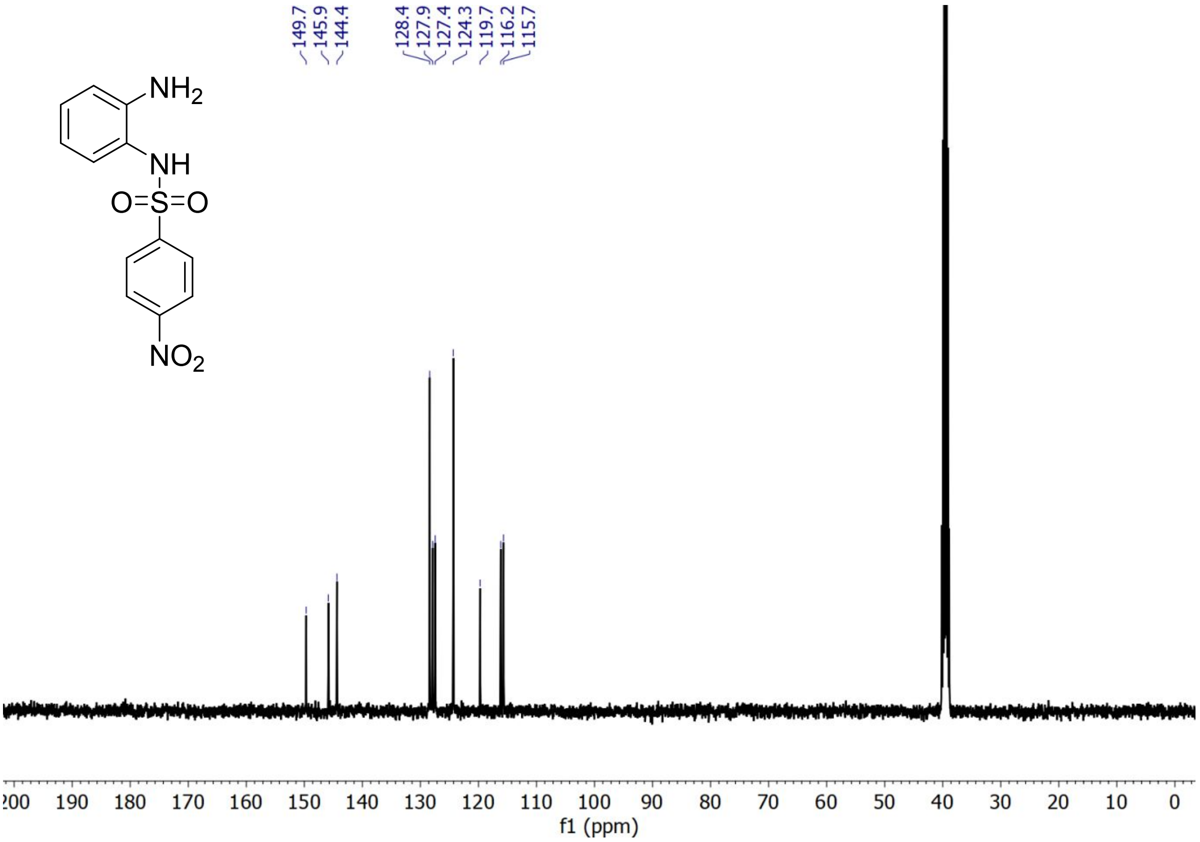

^{13}C NMR spectrum of **9a** (101 MHz, DMSO-d_6)

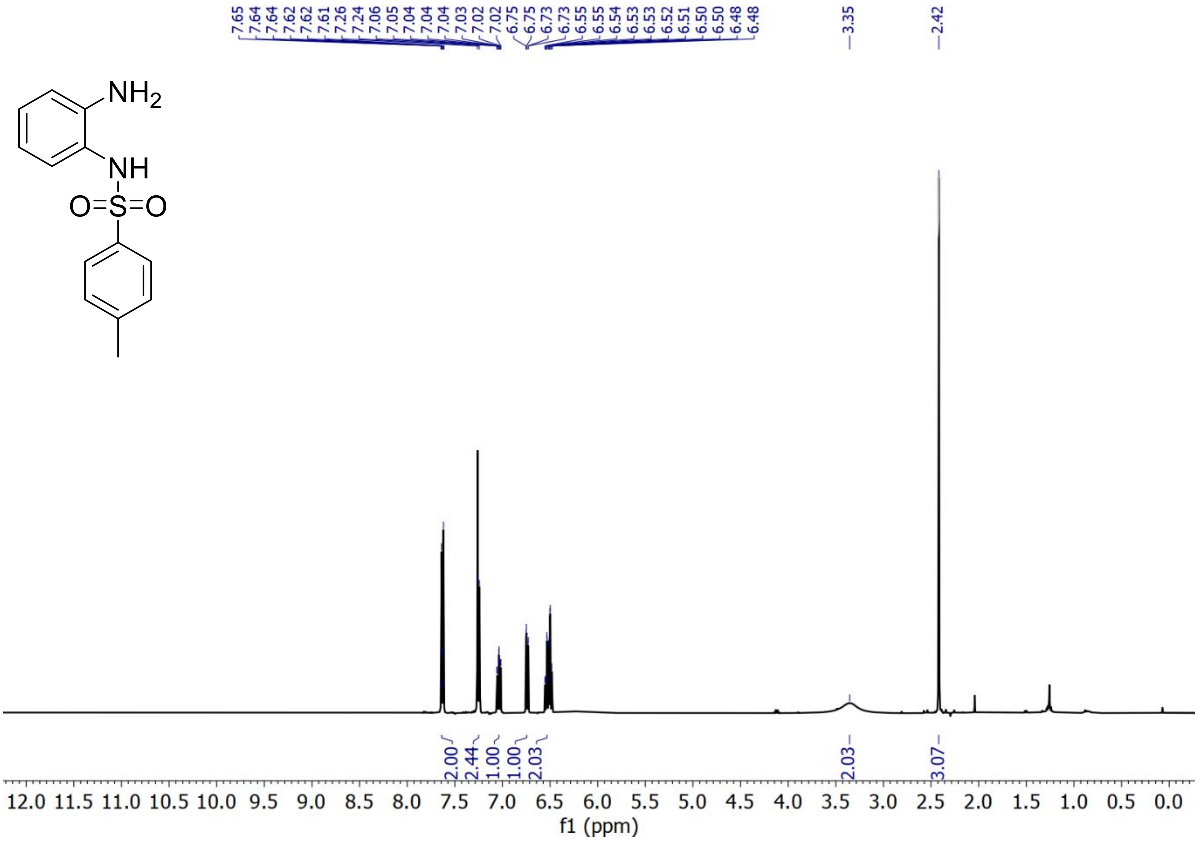

^{1}H NMR spectrum of **9b** (400 MHz, $CDCl_3$)

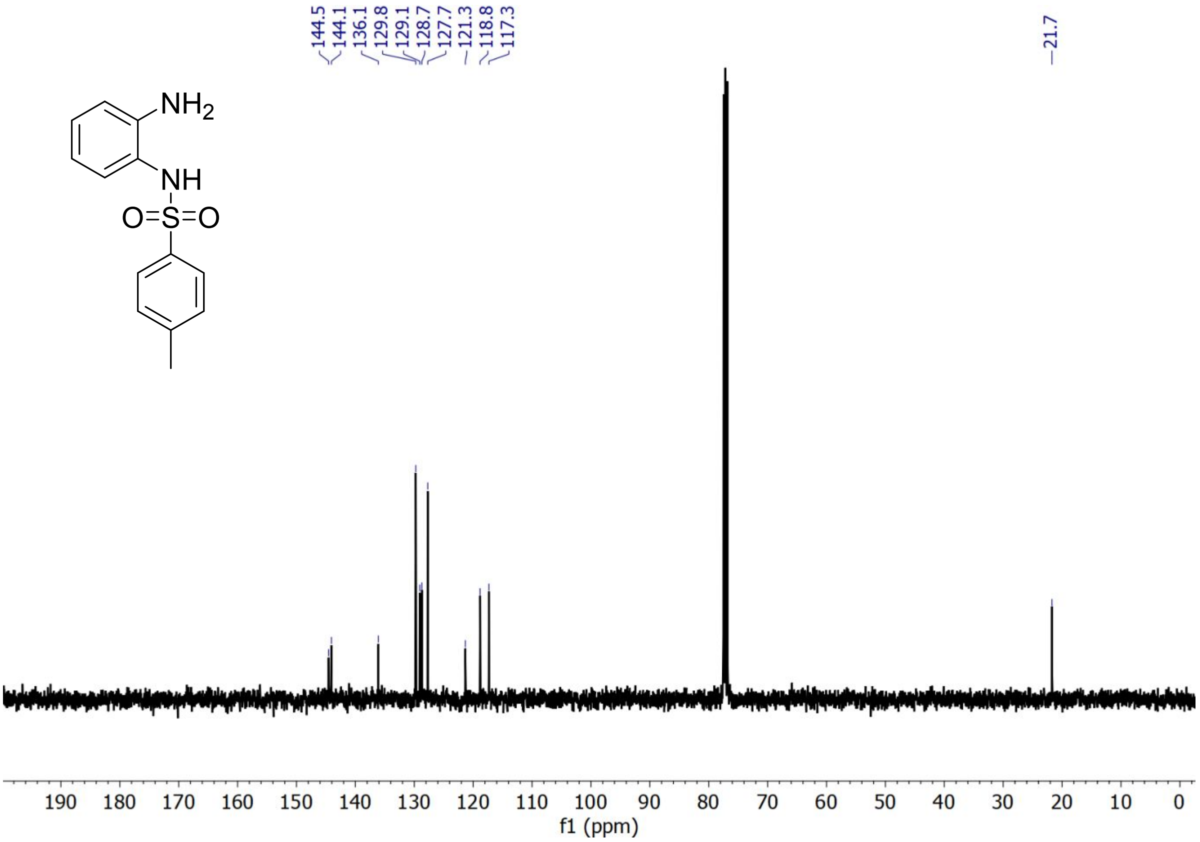

^{13}C NMR spectrum of **9b** (101 MHz, $CDCl_3$)

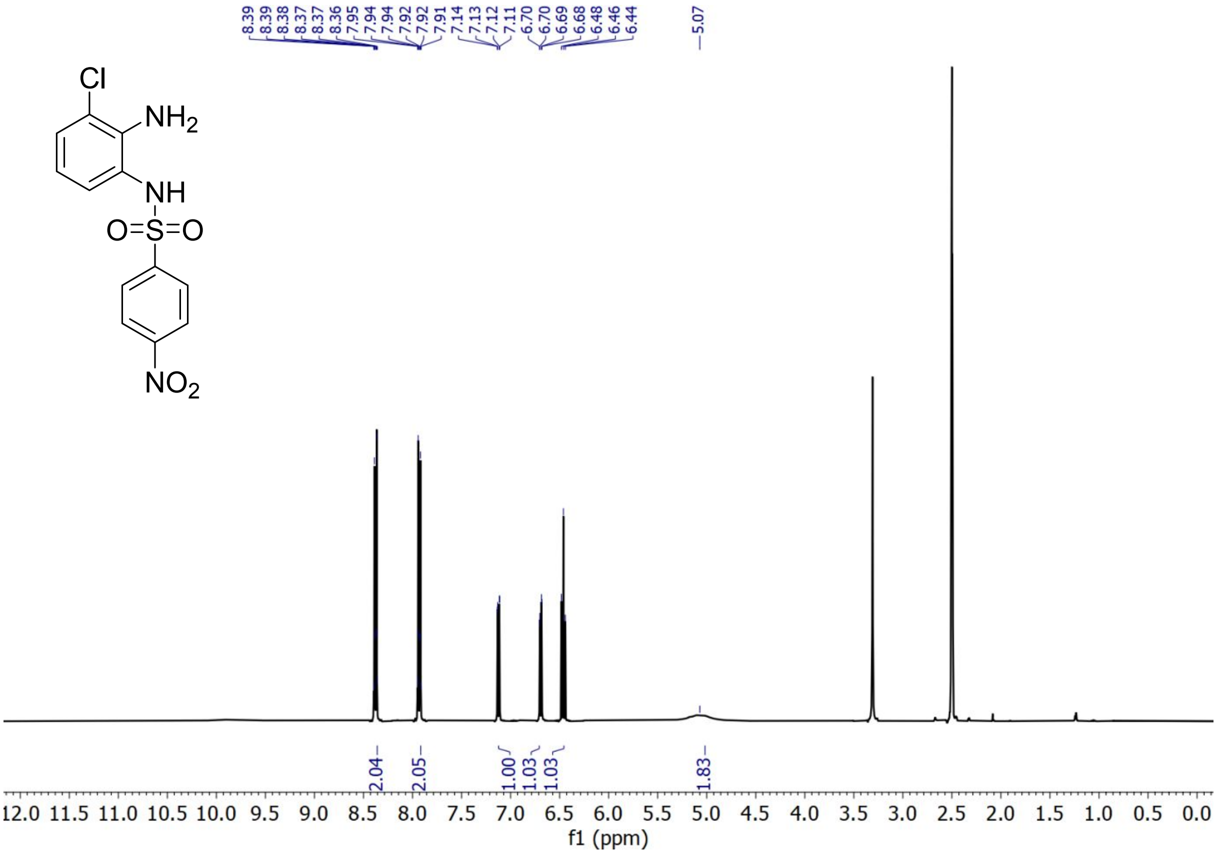

^{1}H NMR spectrum of **9c** (400 MHz, DMSO-d_6)

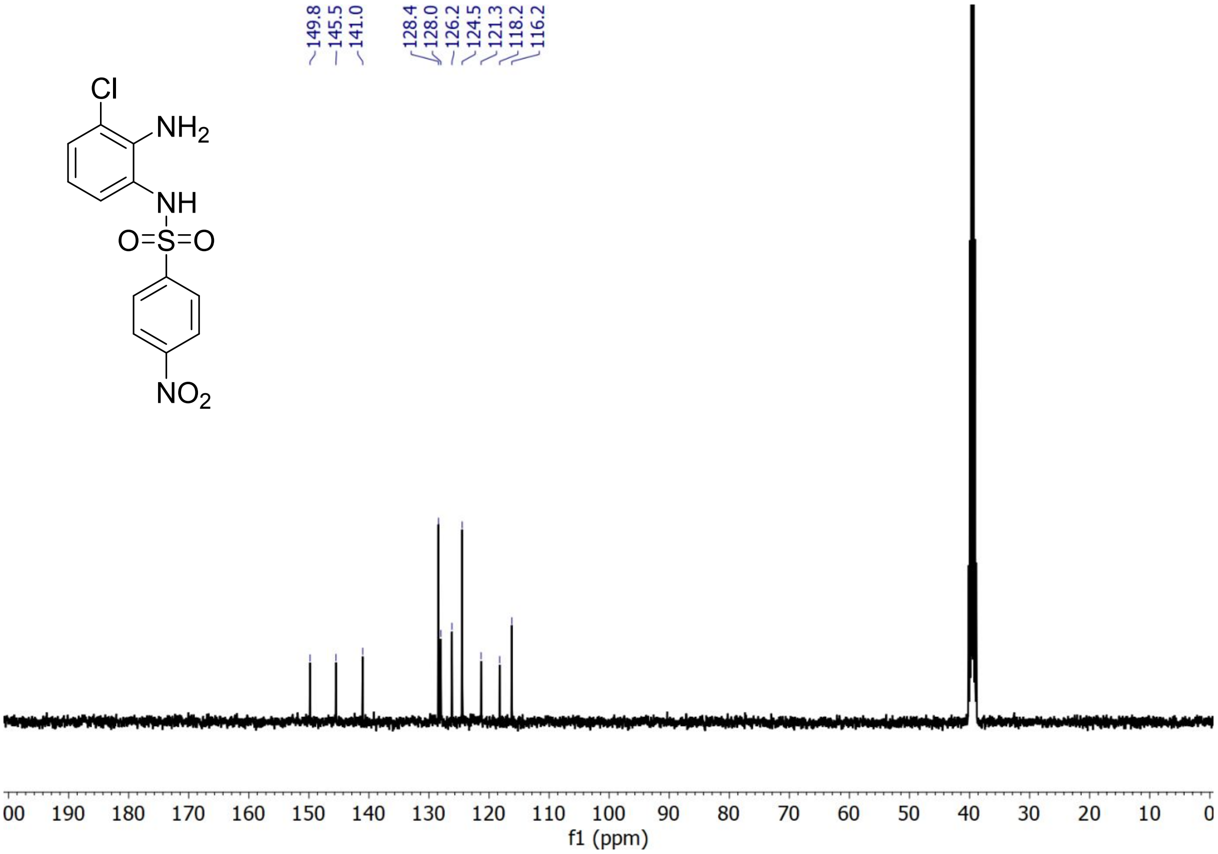

^{13}C NMR spectrum of **9c** (101 MHz, DMSO-d_6)

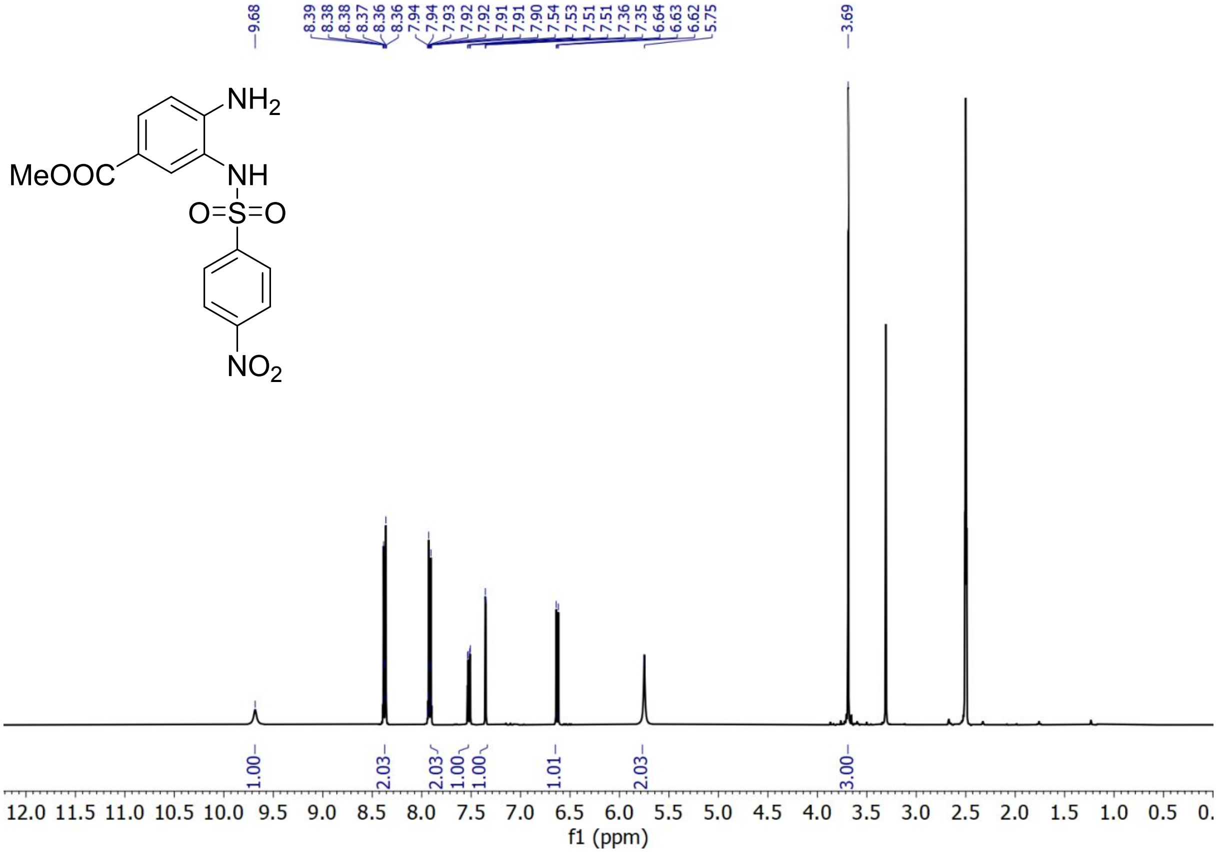

^{1}H NMR spectrum of **9d** (400 MHz, DMSO-d_6)

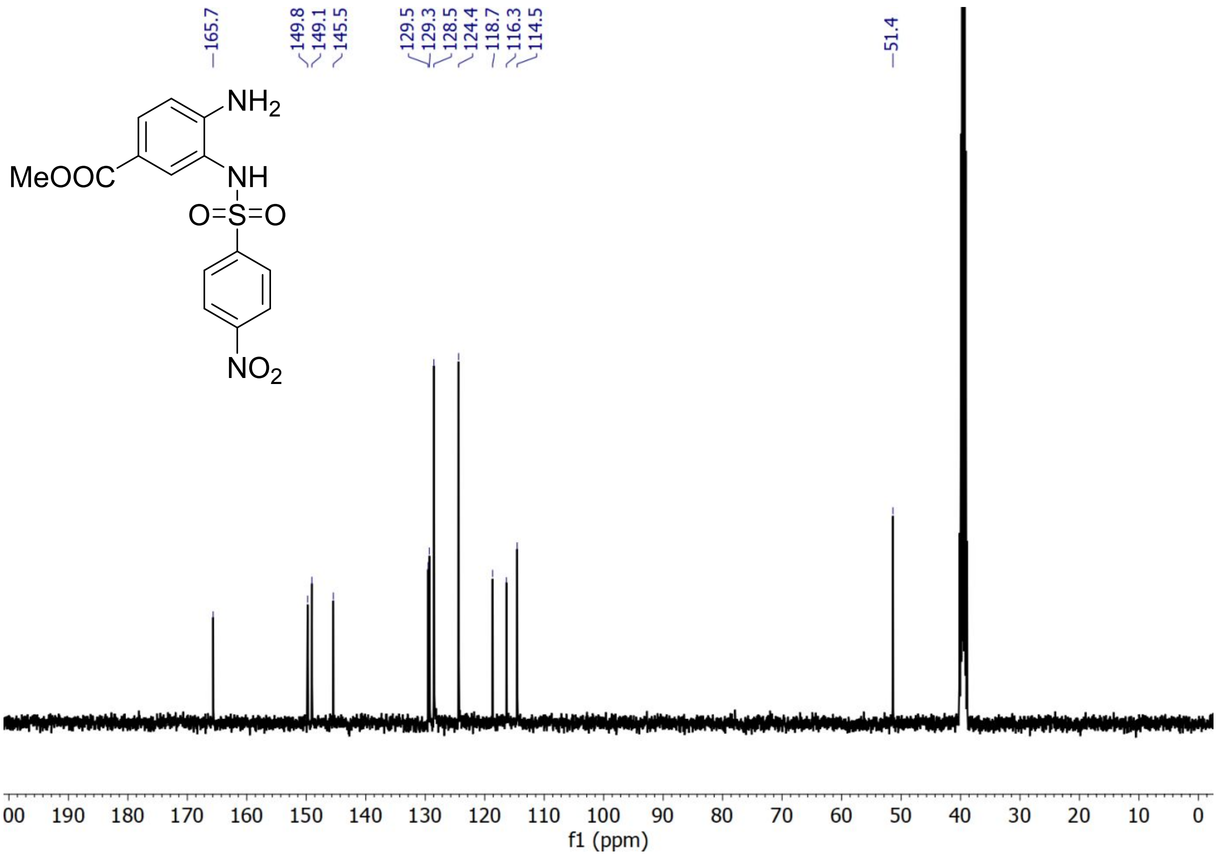

^{13}C NMR spectrum of **9d** (101 MHz, DMSO-d_6)

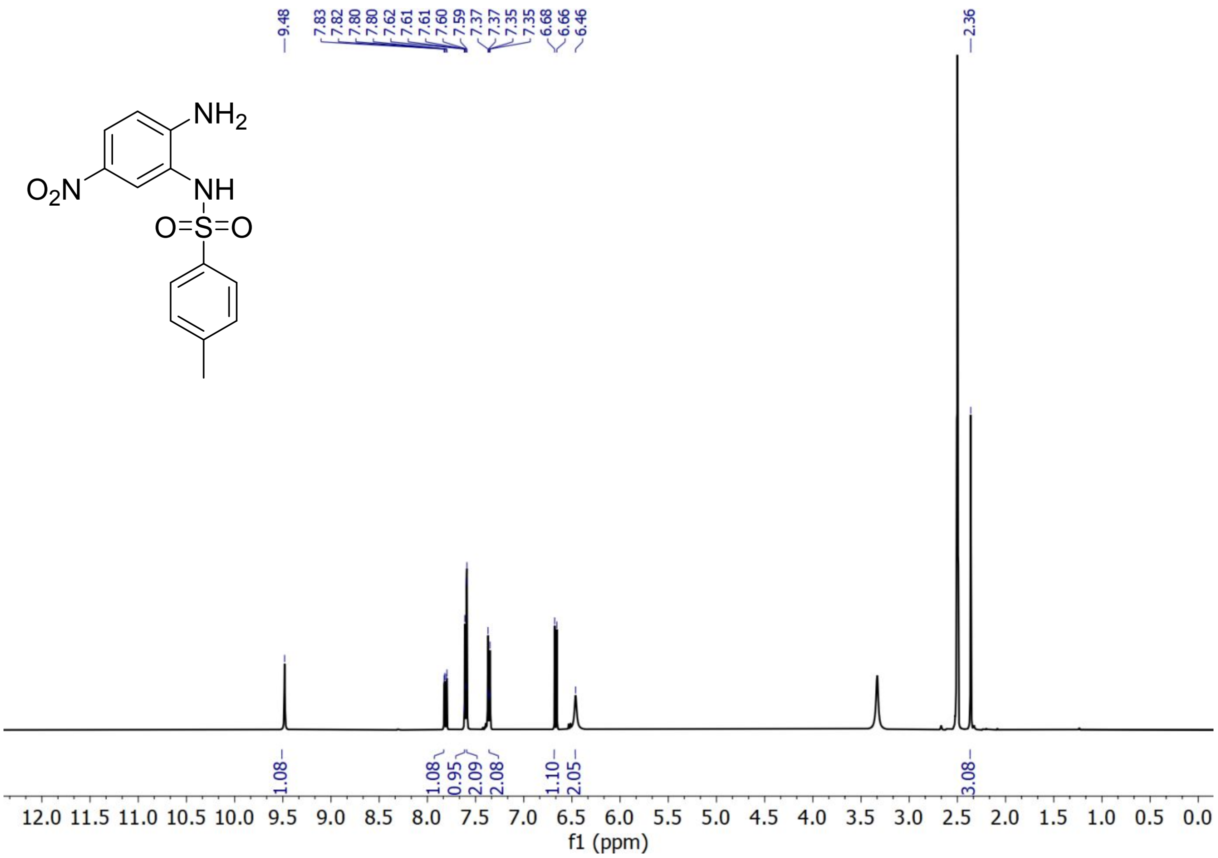

^{1}H NMR spectrum of **9e** (400 MHz, DMSO-d_6)

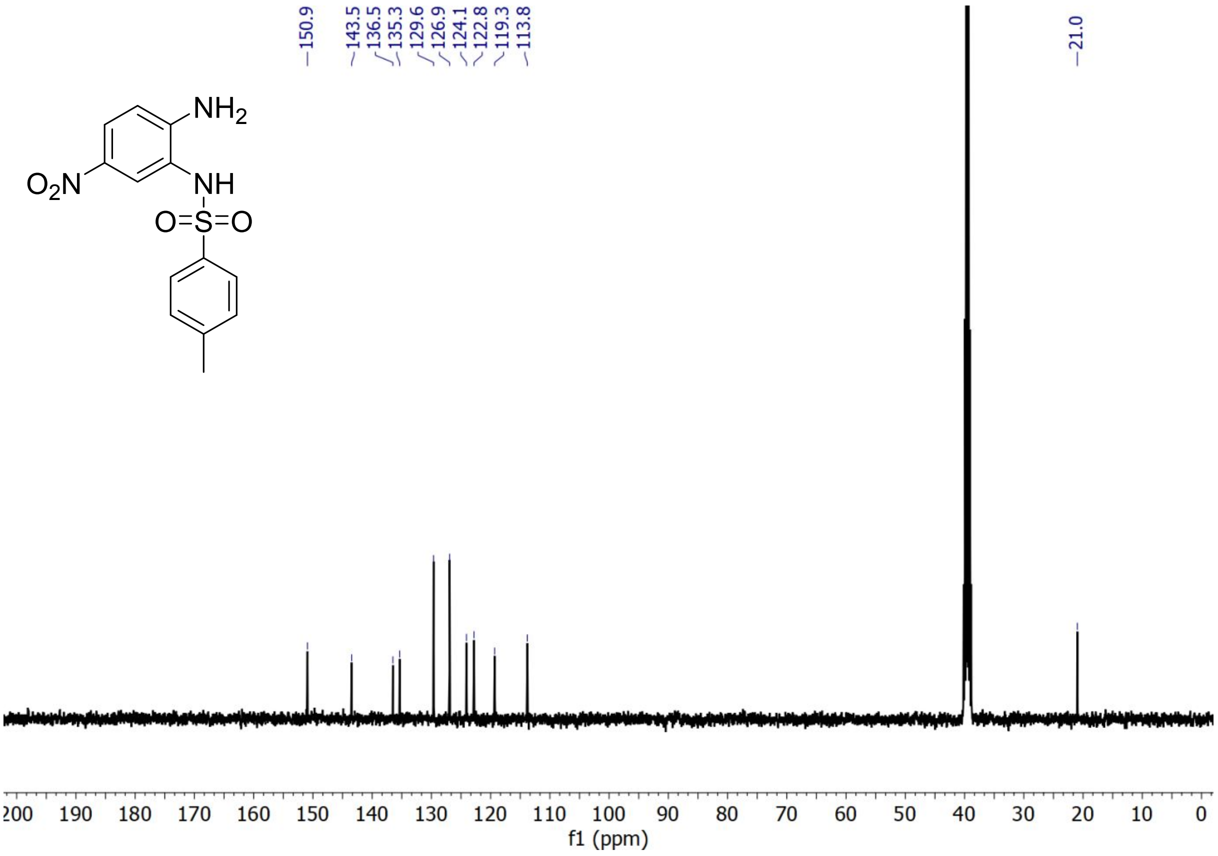

^{13}C NMR spectrum of **9e** (101 MHz, DMSO-d_6)

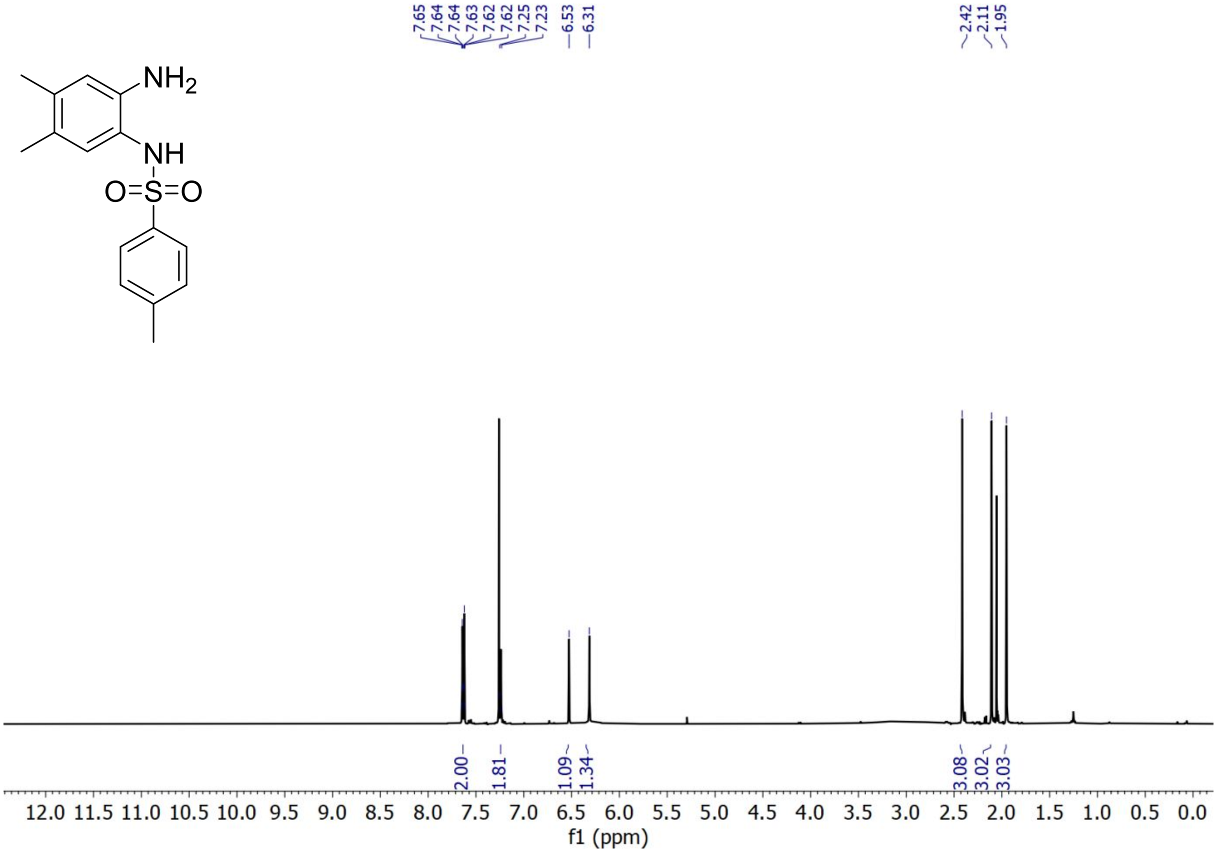

^{1}H NMR spectrum of **9f** (400 MHz, $CDCl_3$)

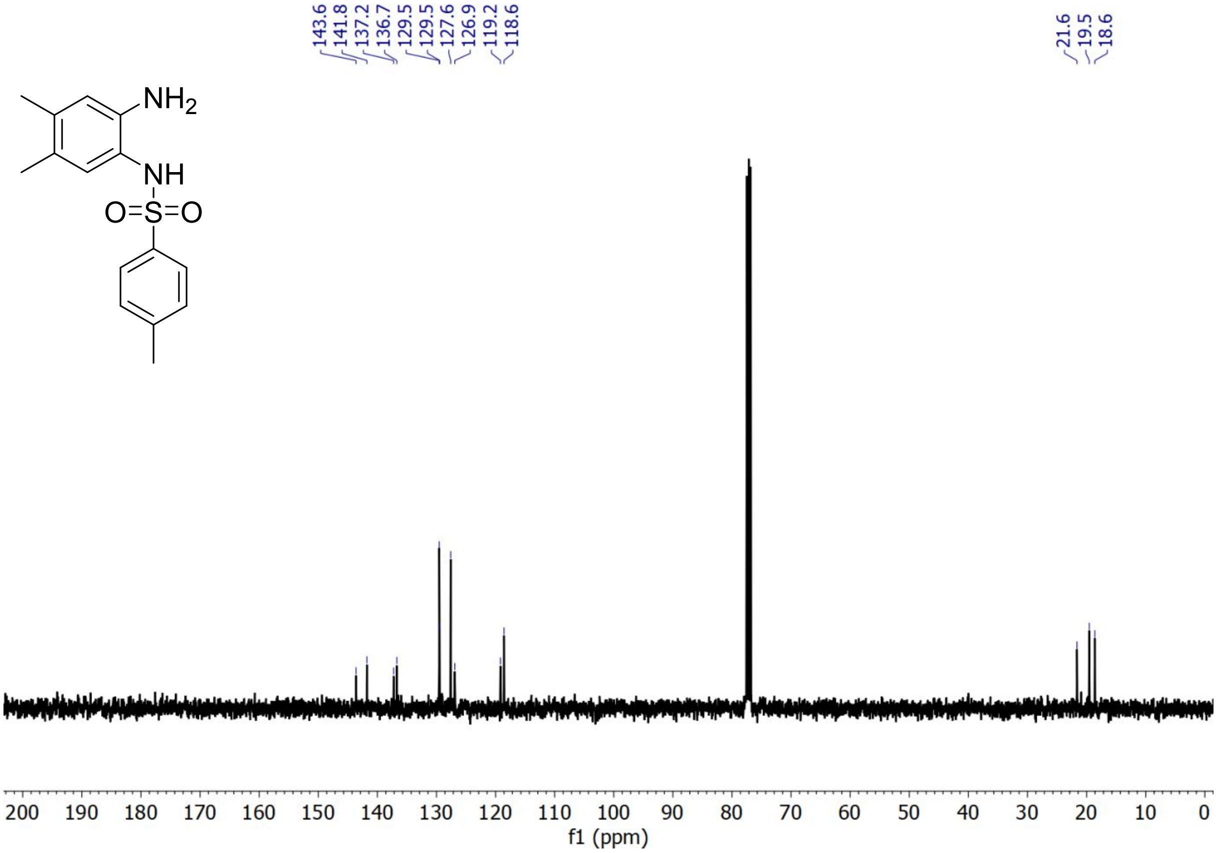

^{13}C NMR spectrum of **9f** (101 MHz, $CDCl_3$)

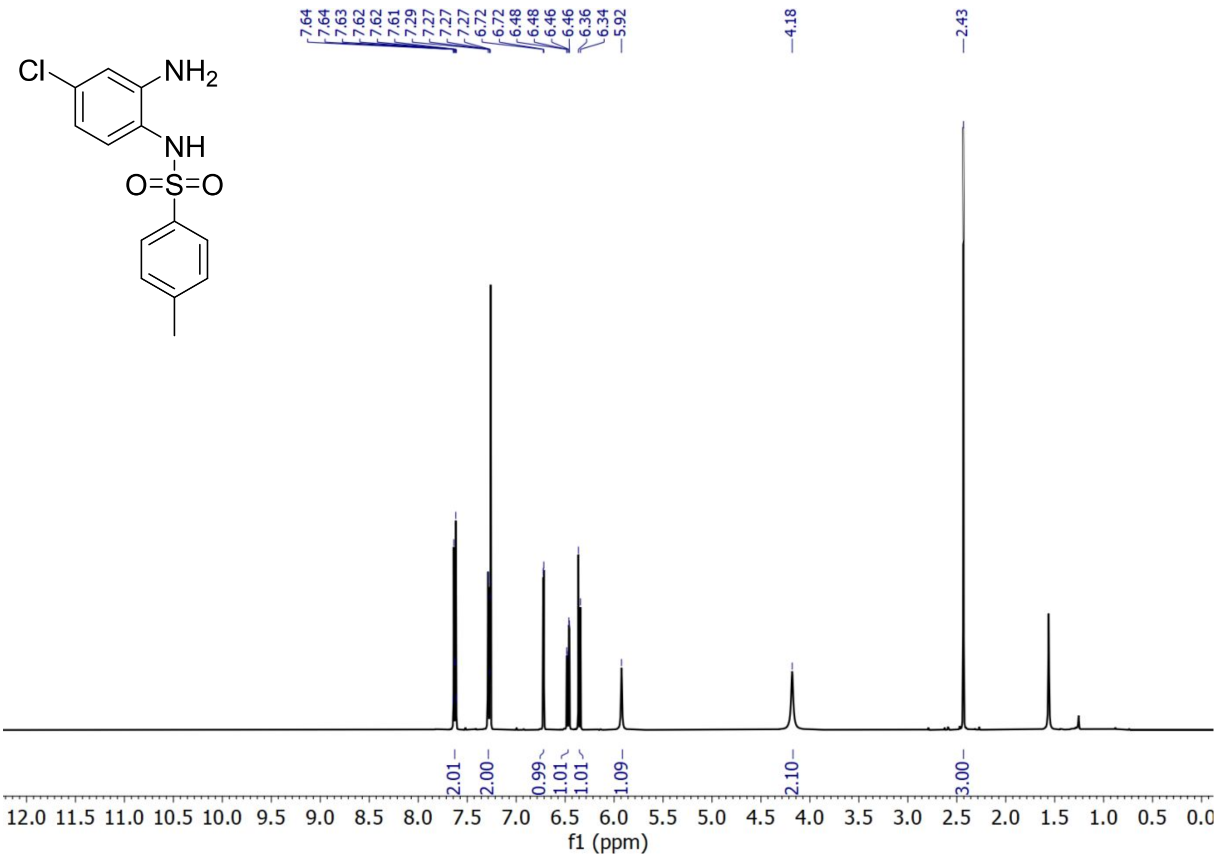

^{1}H NMR spectrum of **9g** (40 MHz, $CDCl_3$)

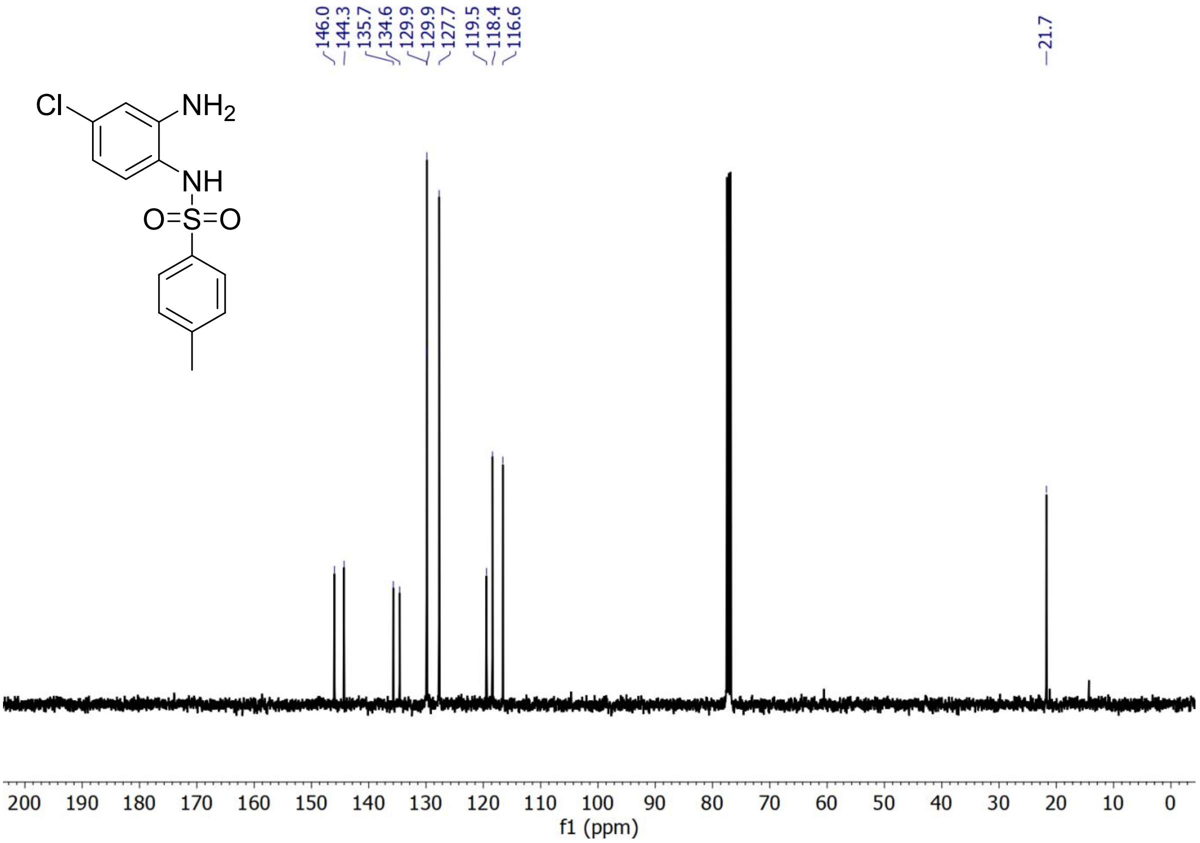

^{13}C NMR spectrum of **9g** (101 MHz, $CDCl_3$)

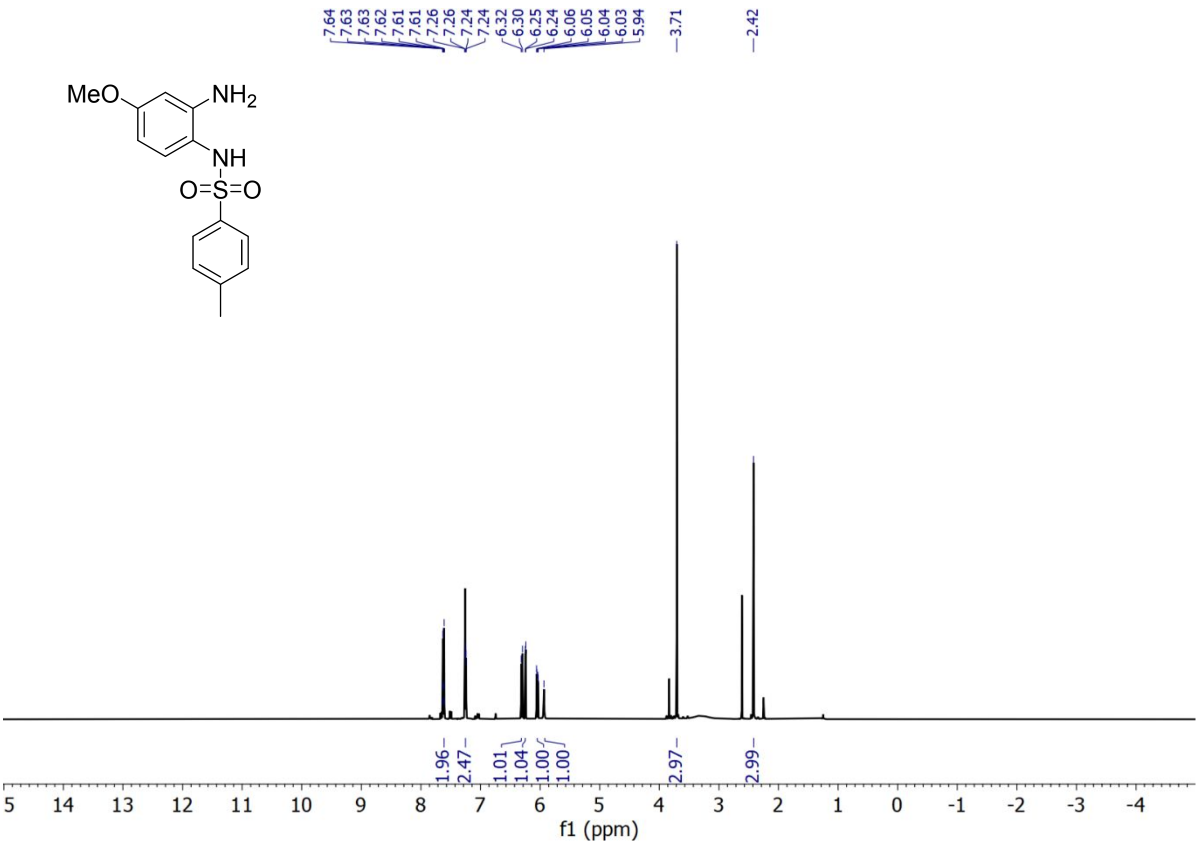

^{1}H NMR spectrum of **9h** (400 MHz, $CDCl_3$)

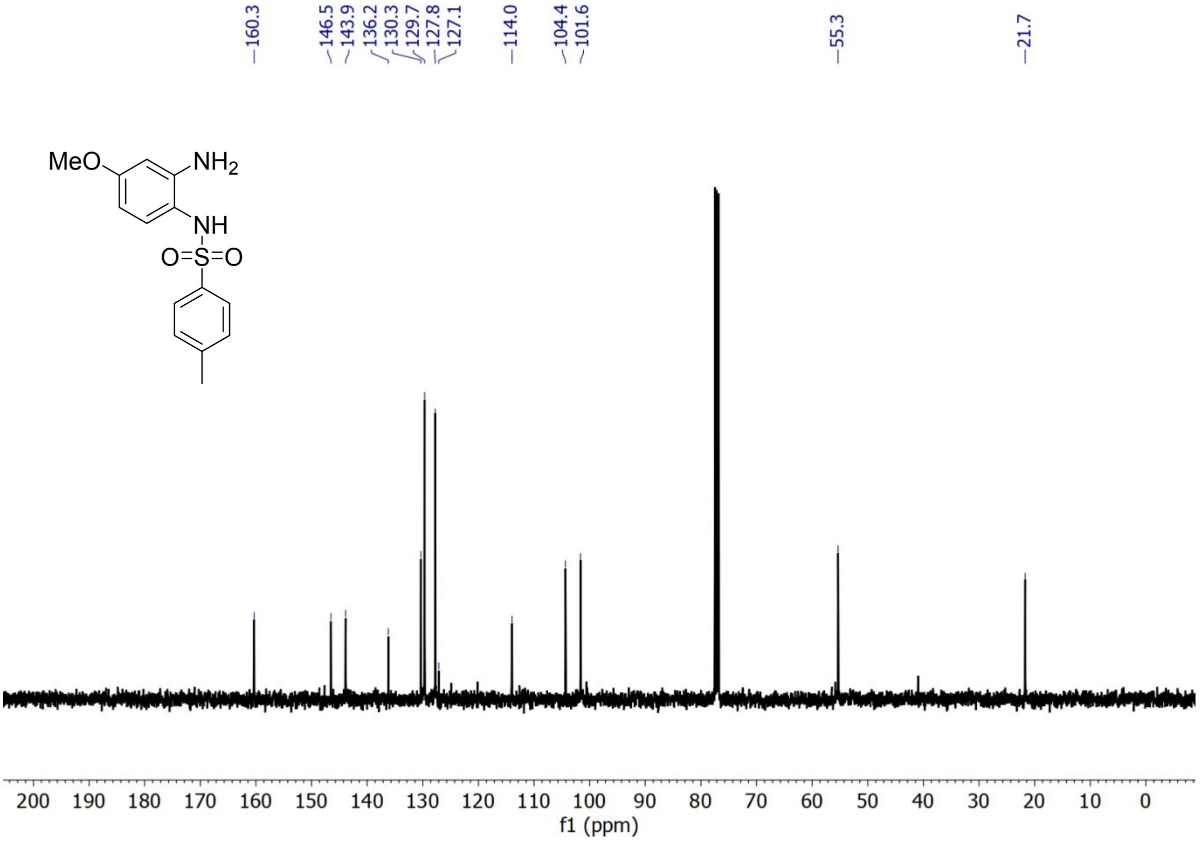

^{13}C NMR spectrum of **9h** (101 MHz, $CDCl_3$)

2. Copies of NMR spectra of *N*-alkylation products (3a-c, 10a-n, 13a-b)

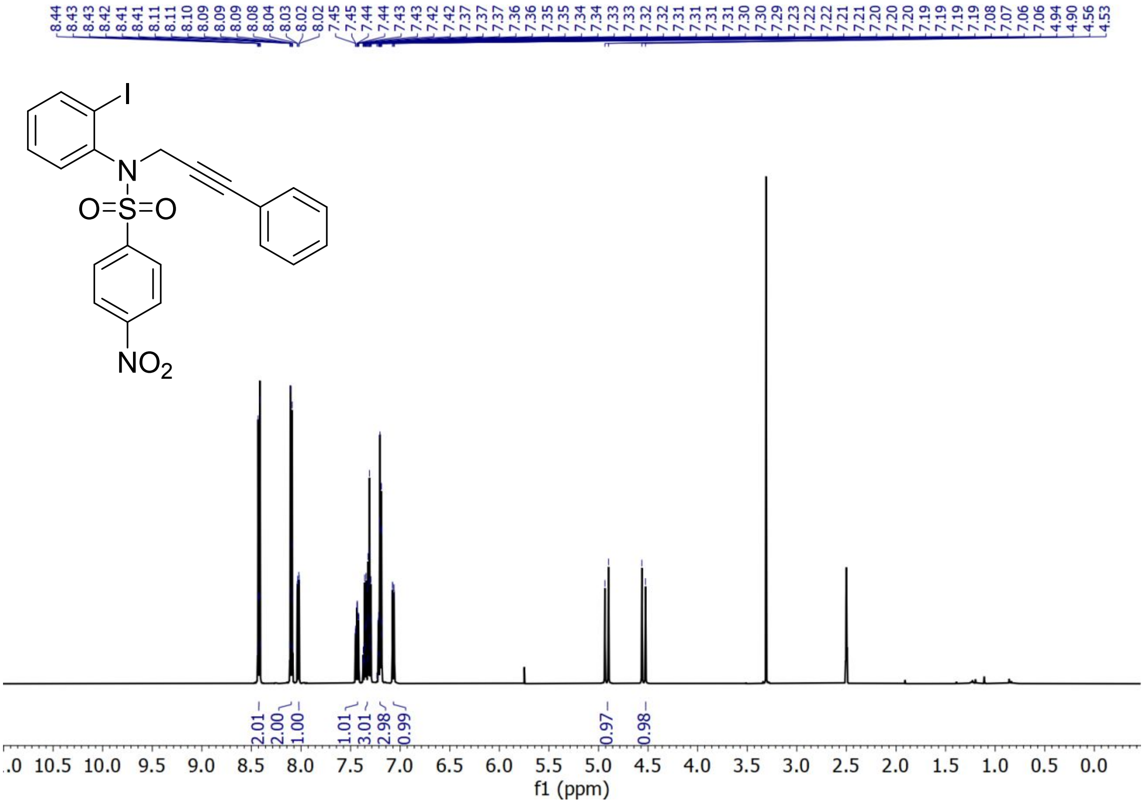

^{1}H NMR spectrum of **3a** (500 MHz, DMSO-d_6)

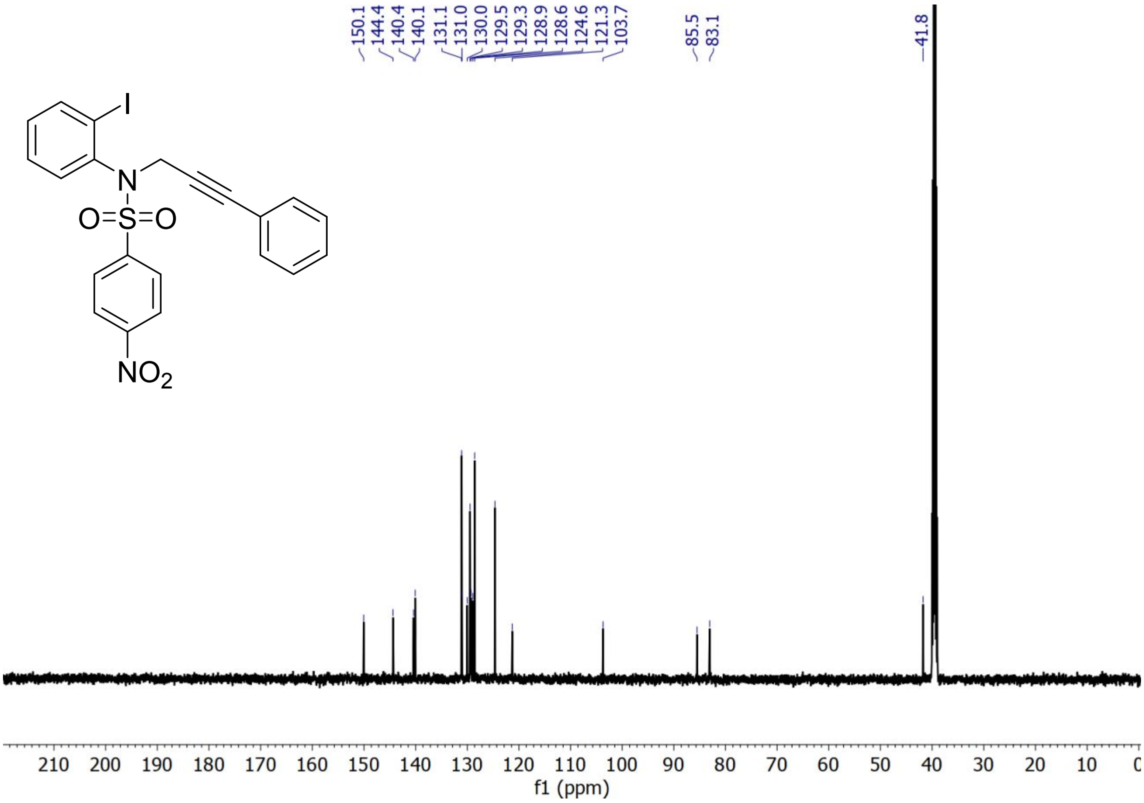

^{13}C NMR spectrum of **3a** (126 MHz, DMSO-d_6)

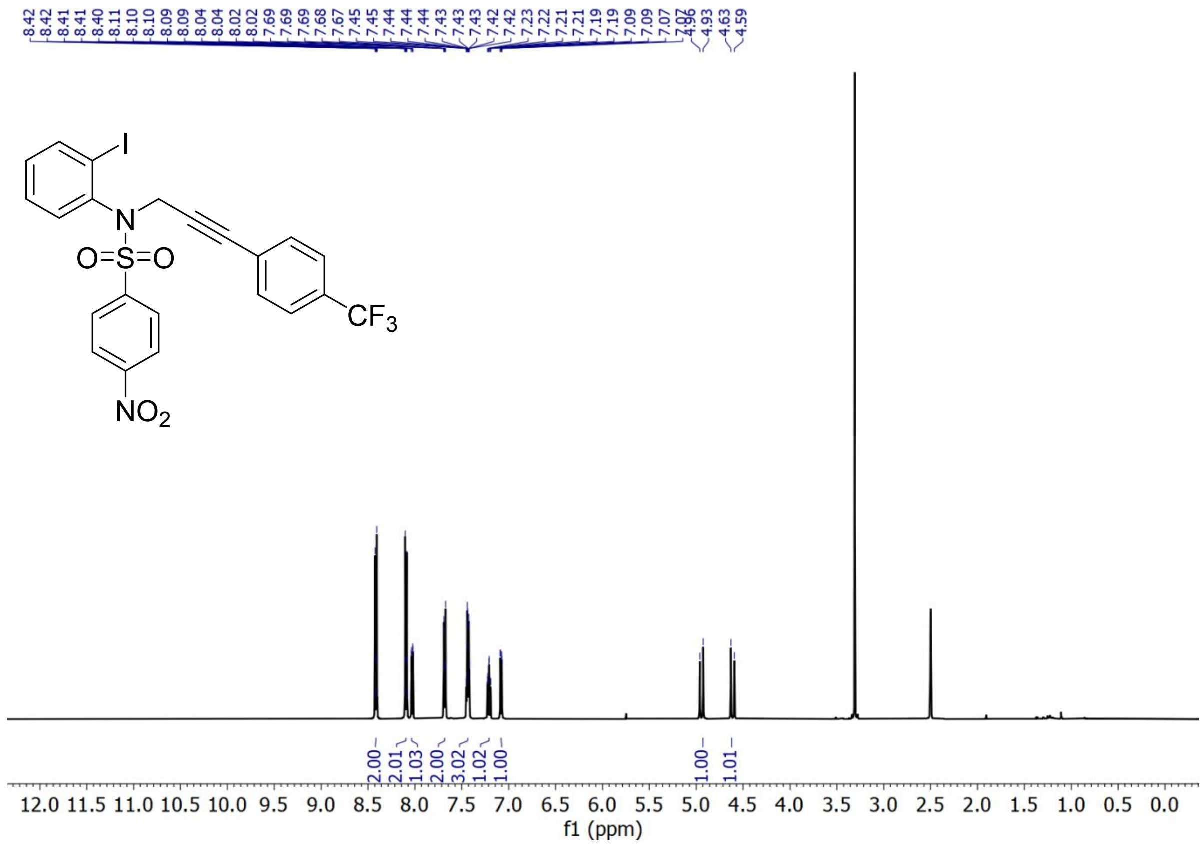

^{1}H NMR spectrum of **3b** (500 MHz, DMSO-d_6)

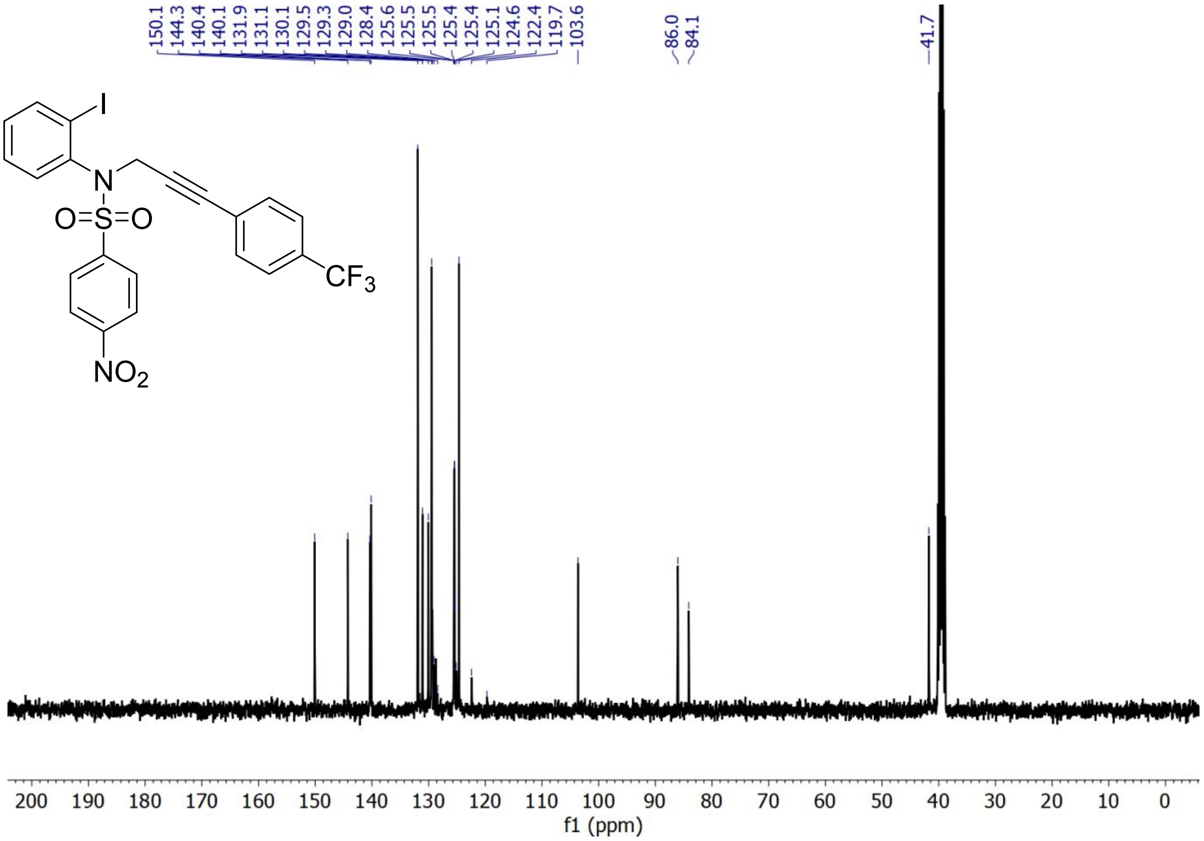

^{1}H NMR spectrum of **3b** (101 MHz, DMSO-d_6)

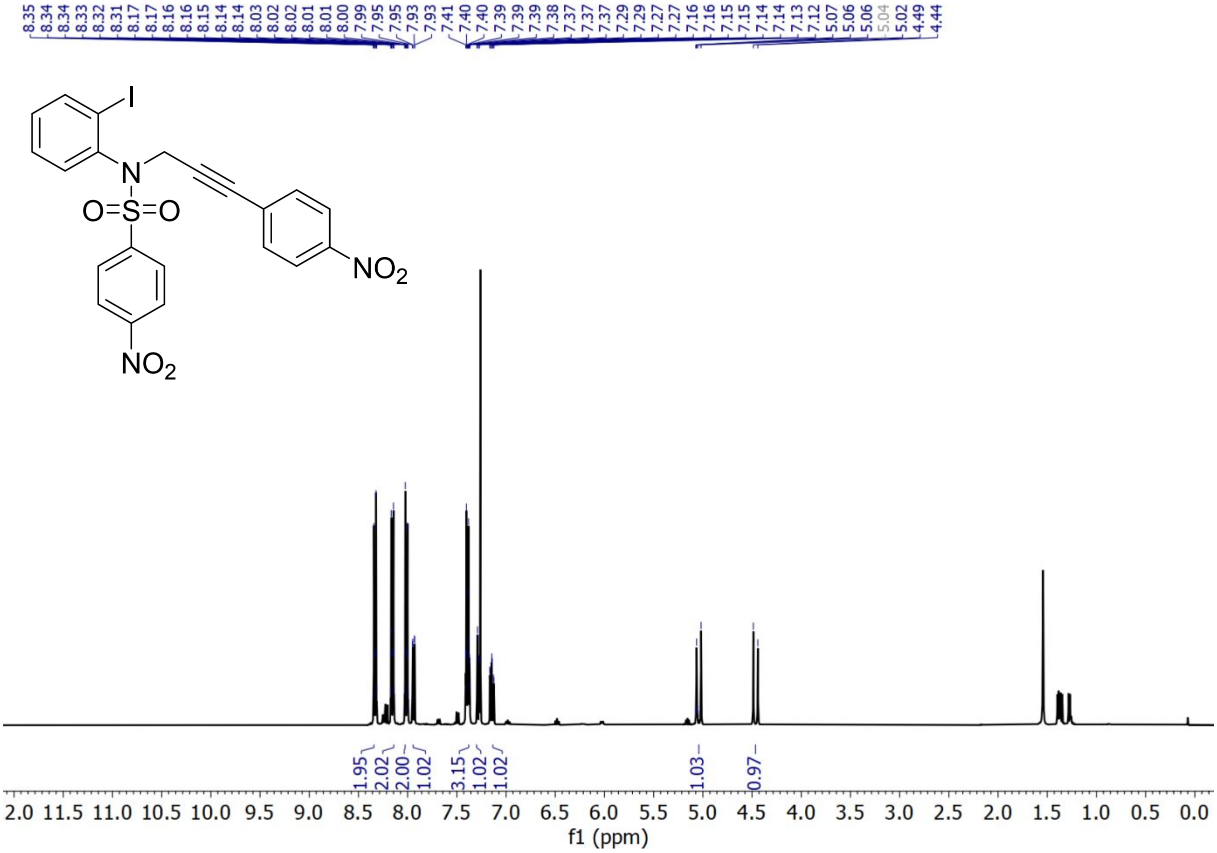

^{1}H NMR spectrum of **3c** (400 MHz, $CDCl_3$)

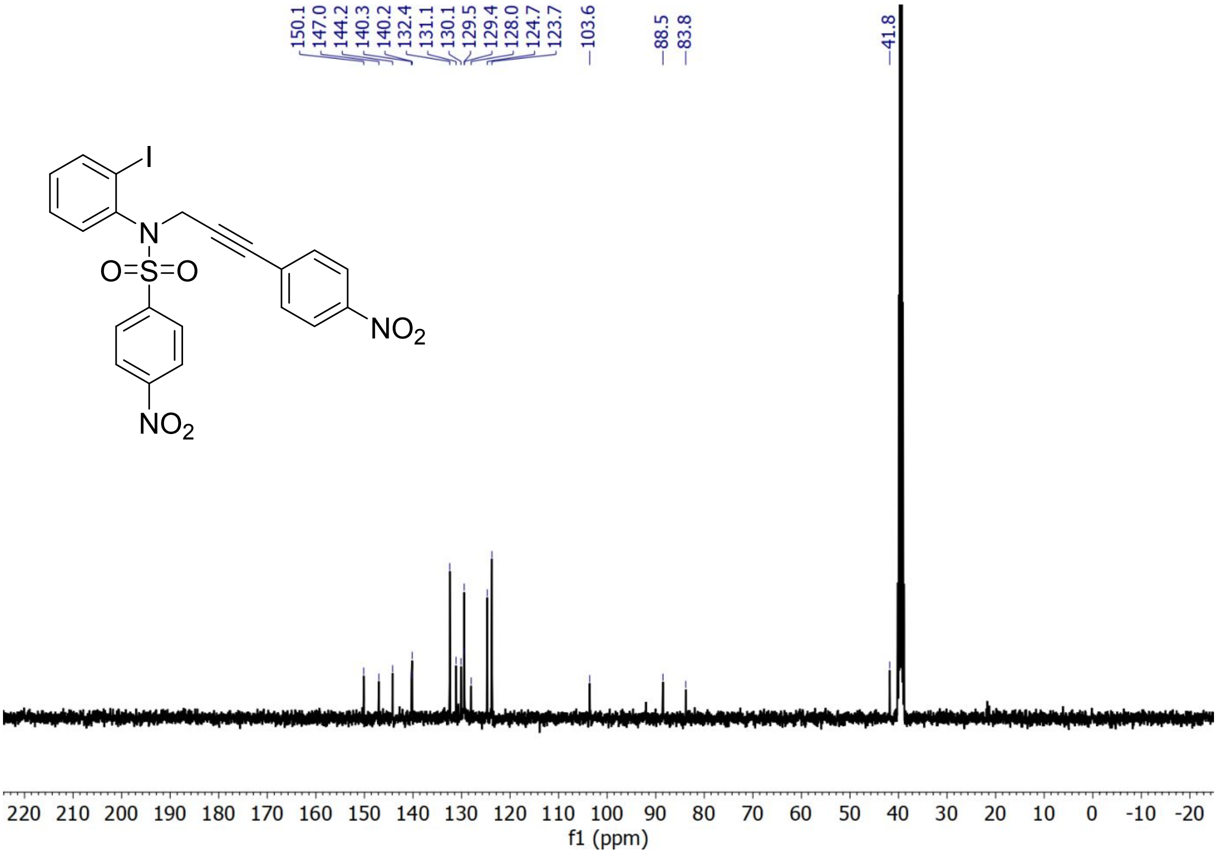

^{13}C NMR spectrum of **3c** (101 MHz, $CDCl_3$)

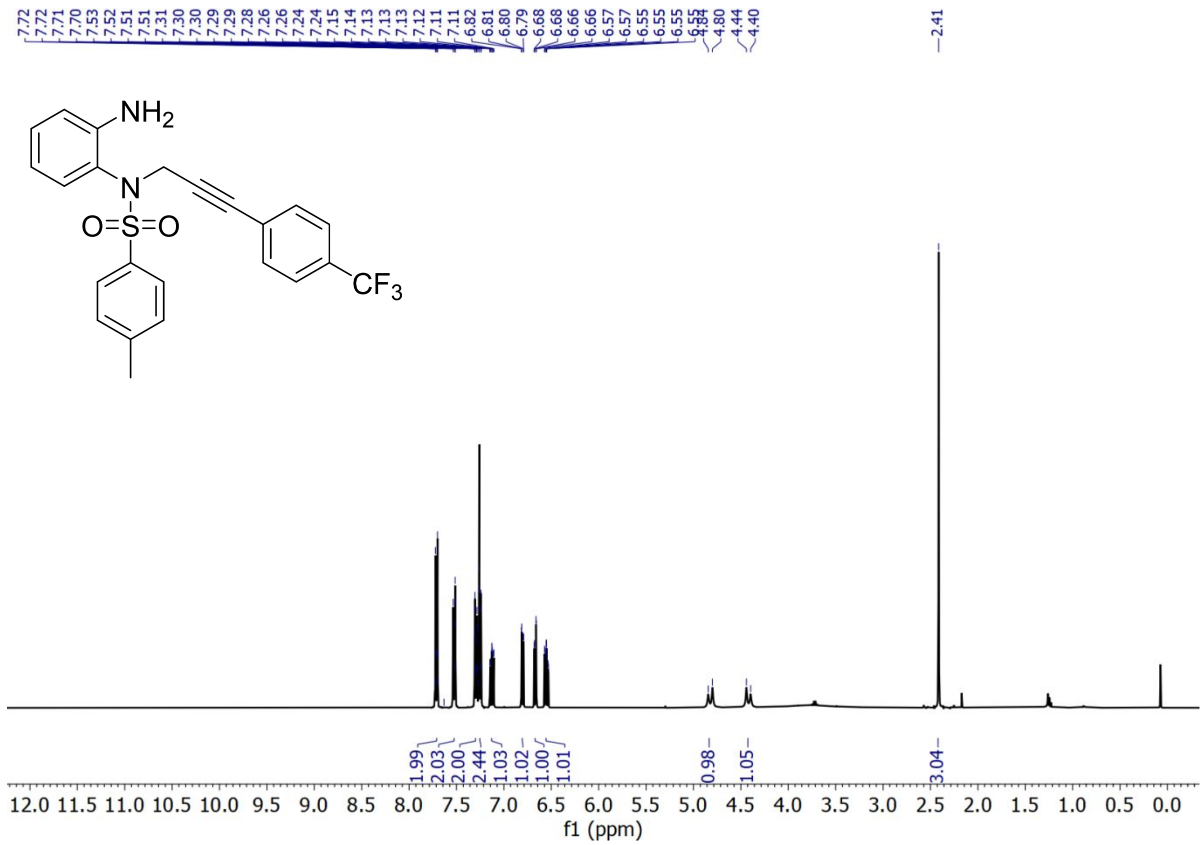

^{1}H NMR spectrum of **10a** (400 MHz, $CDCl_3$).

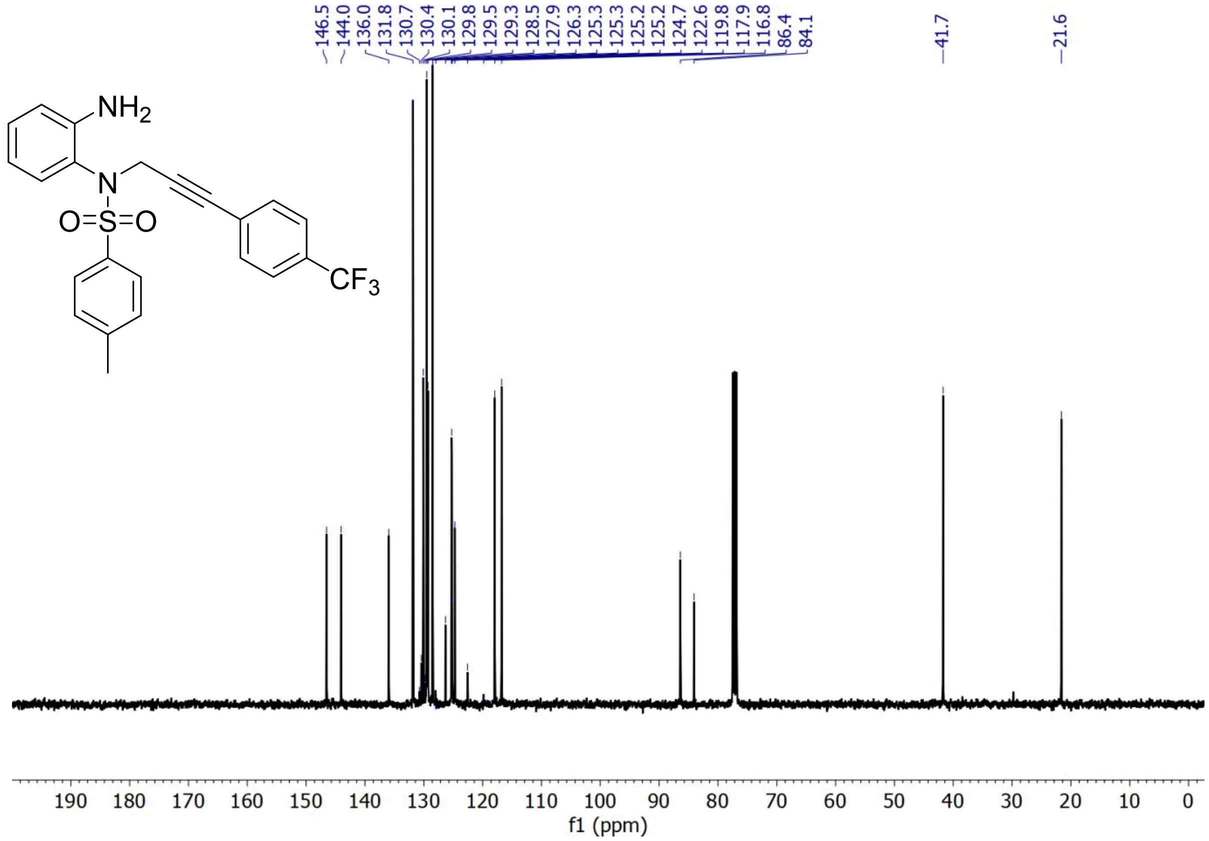

^{13}C NMR spectrum of **10a** (101 MHz, $CDCl_3$).

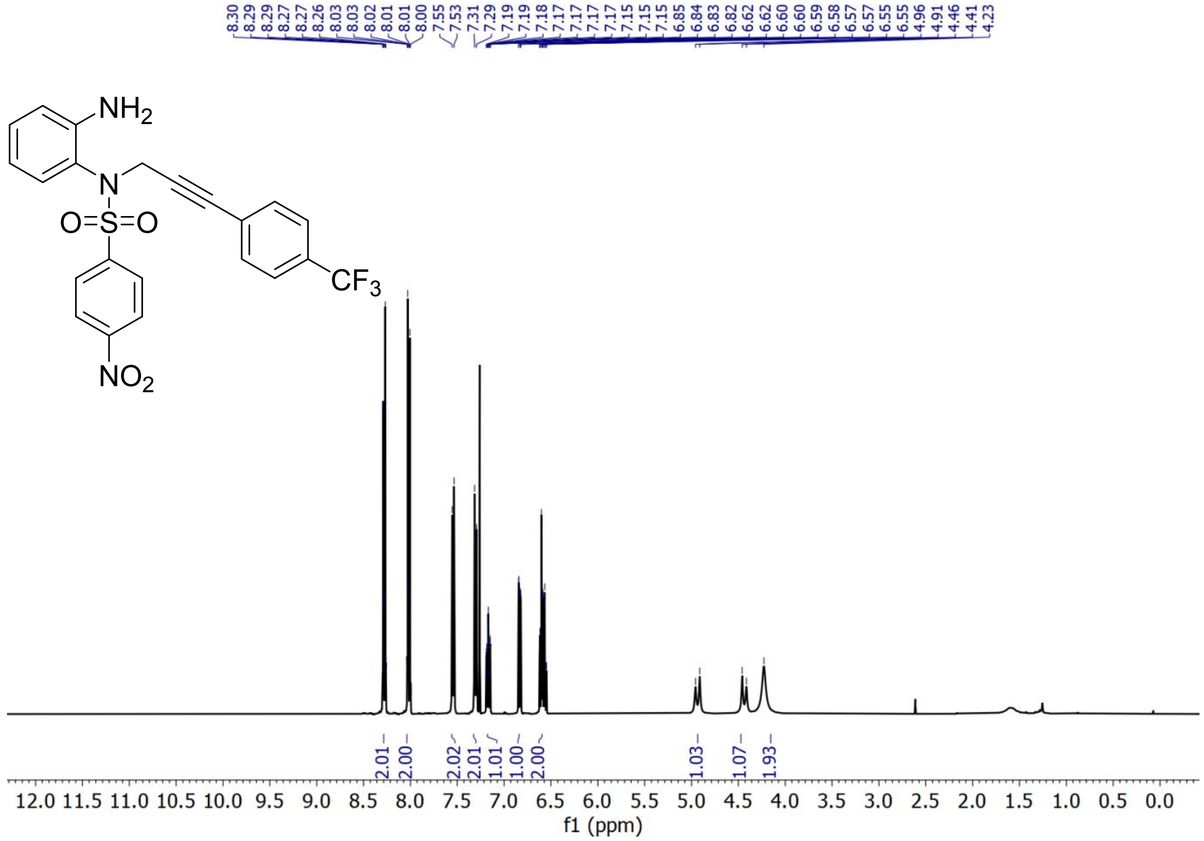

^{1}H NMR spectrum of **10b** (400 MHz, $CDCl_3$)

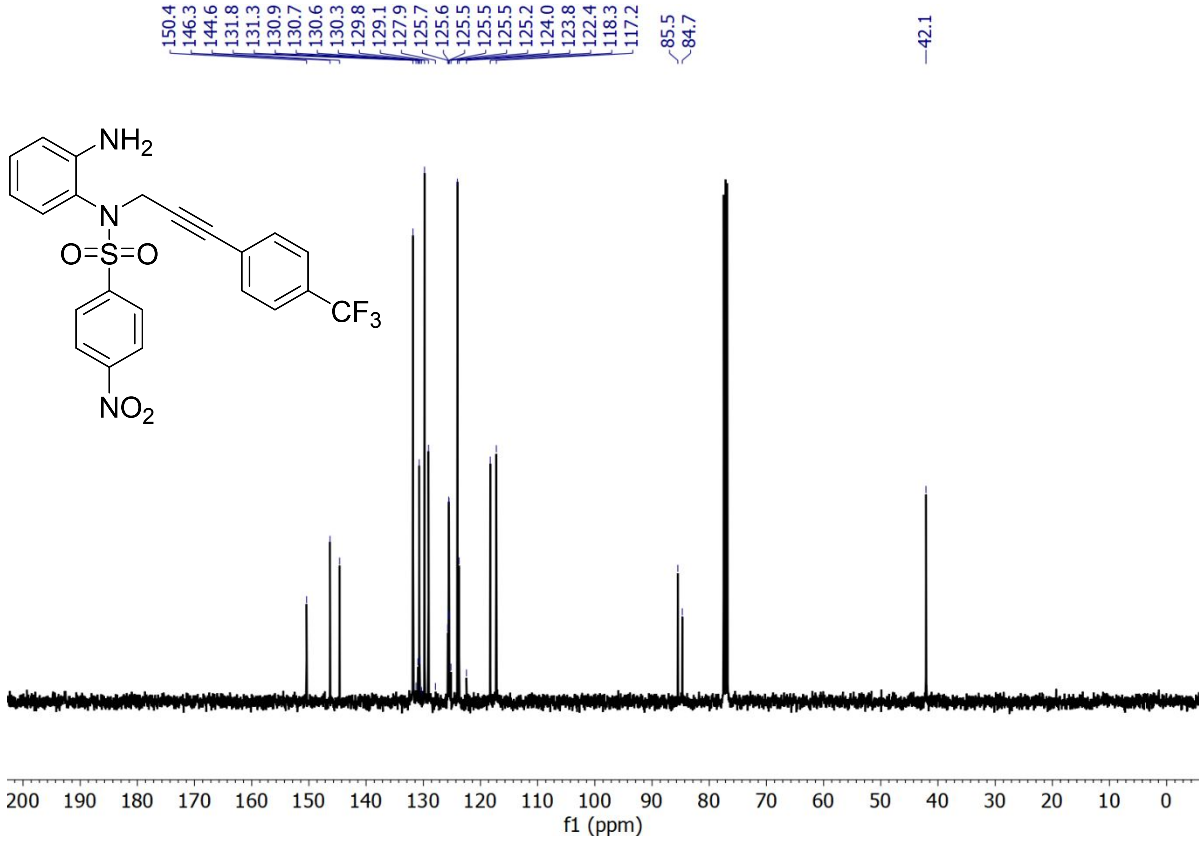

^{13}C NMR spectrum of **10b** (101 MHz, $CDCl_3$)

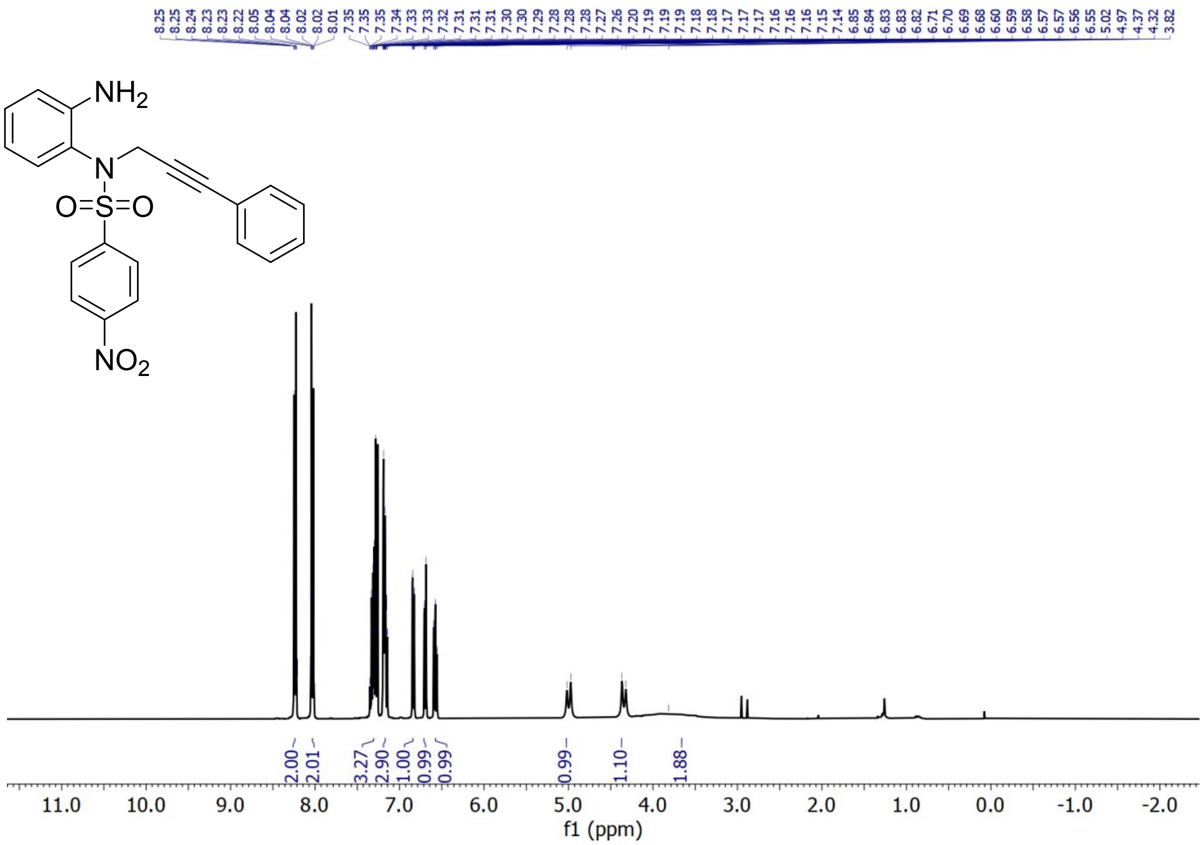

^{1}H NMR spectrum of **10c** (400 MHz, $CDCl_3$)

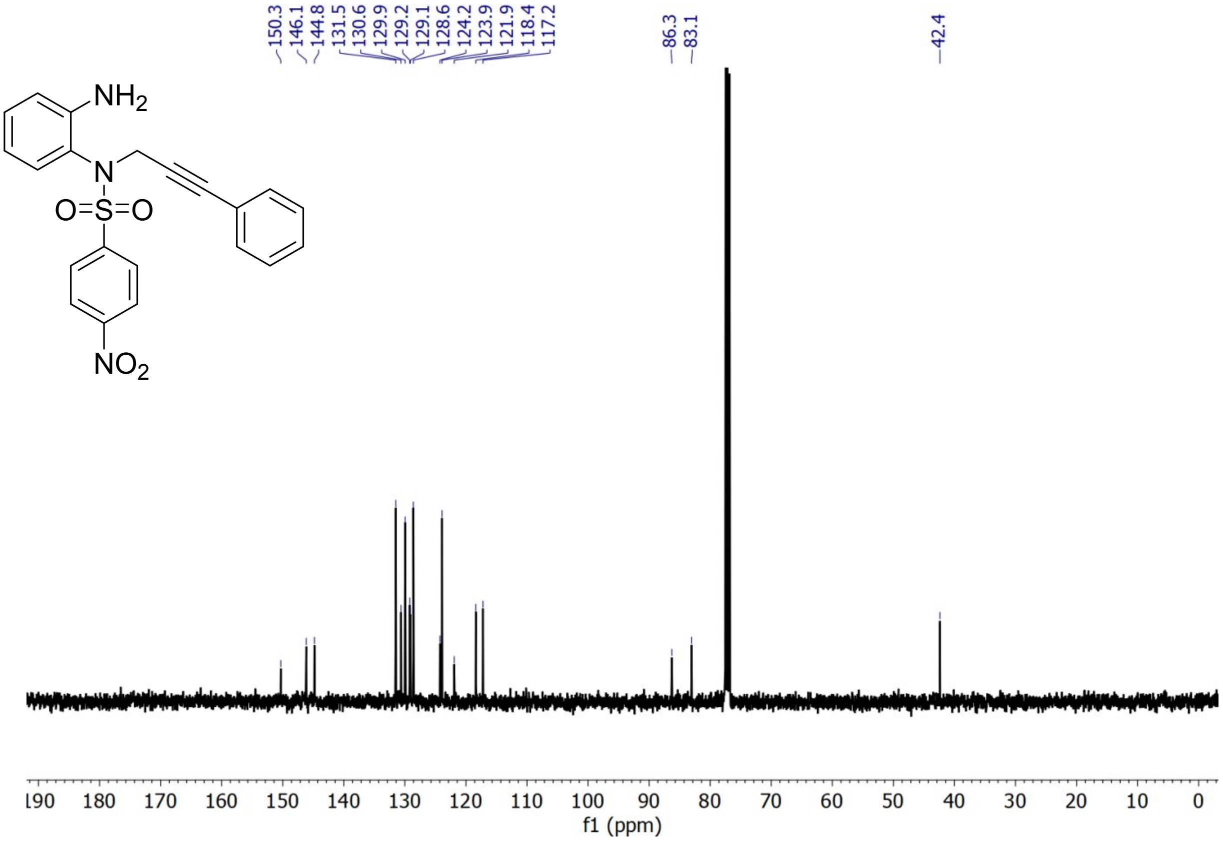

^{13}C NMR spectrum of **10c** (101 MHz, $CDCl_3$)

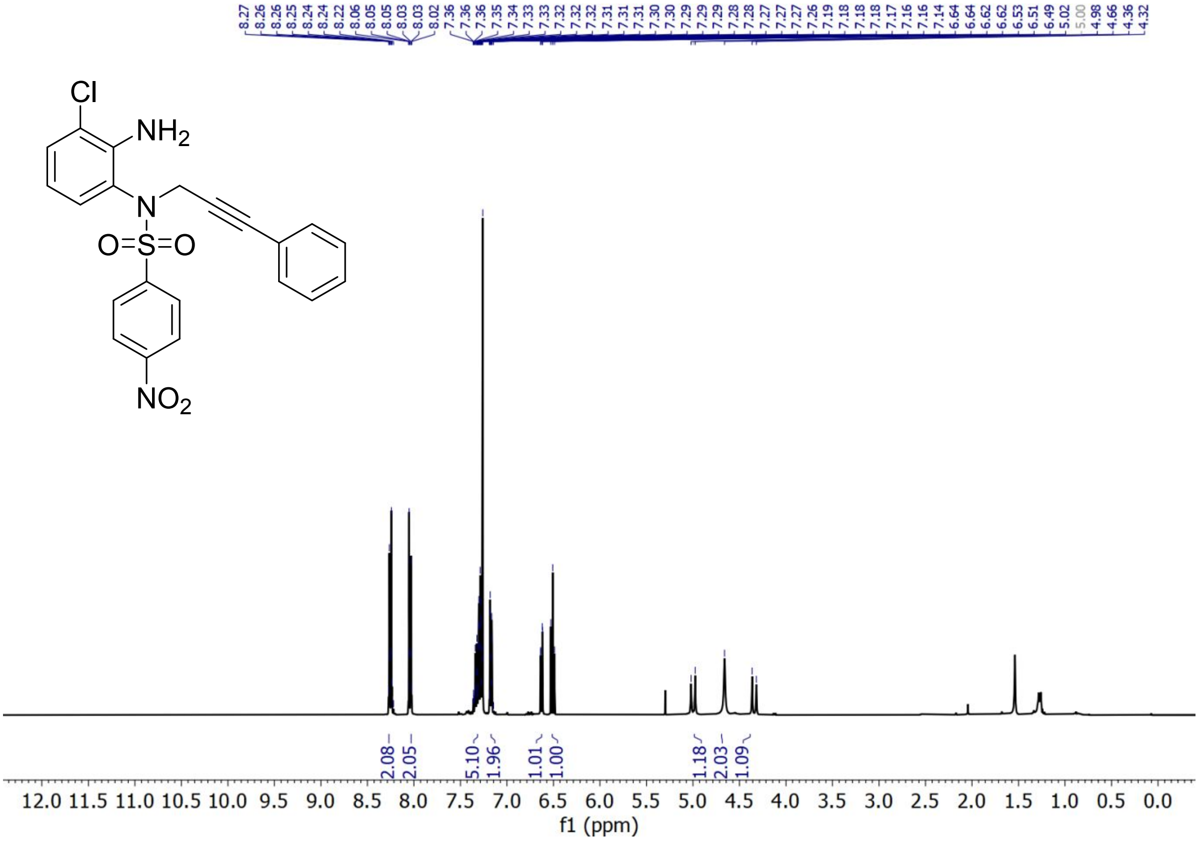

^{1}H NMR spectrum of **10d** (400 MHz, $CDCl_3$) signal at 1.27 ppm belongs to residual DIHD; yield of product **10d** was calculated excluding the mass of DIHD

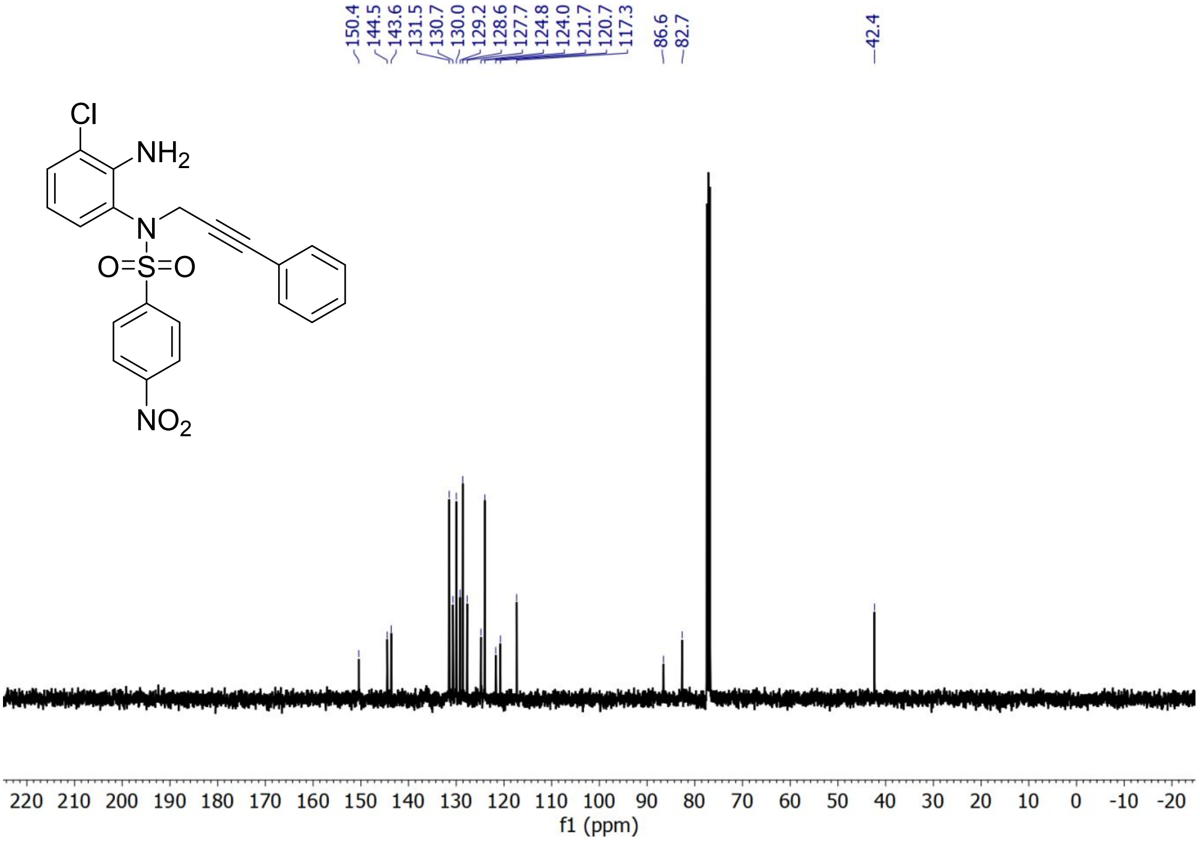

^{13}C NMR spectrum of **10d** (101 MHz, $CDCl_3$)

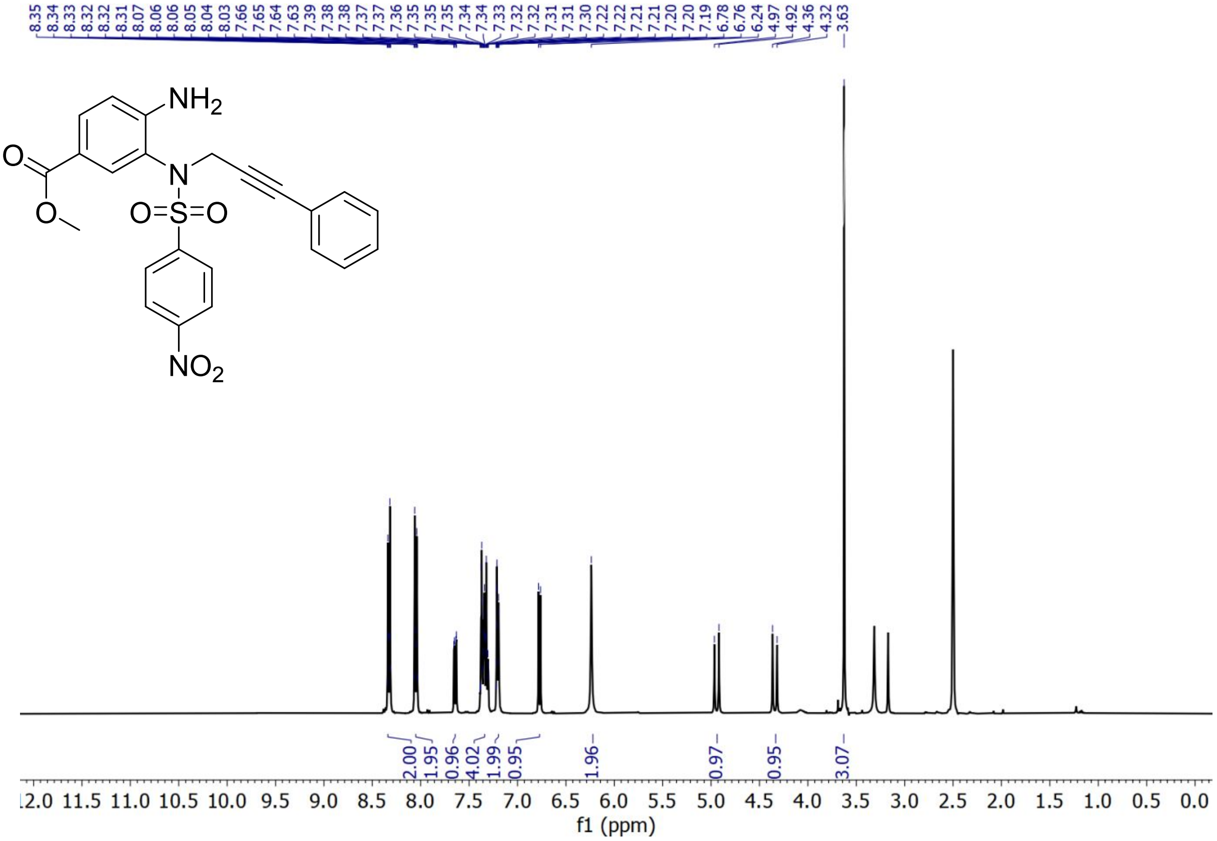

^{1}H NMR spectrum of **10f** (400 MHz, DMSO-d_6)

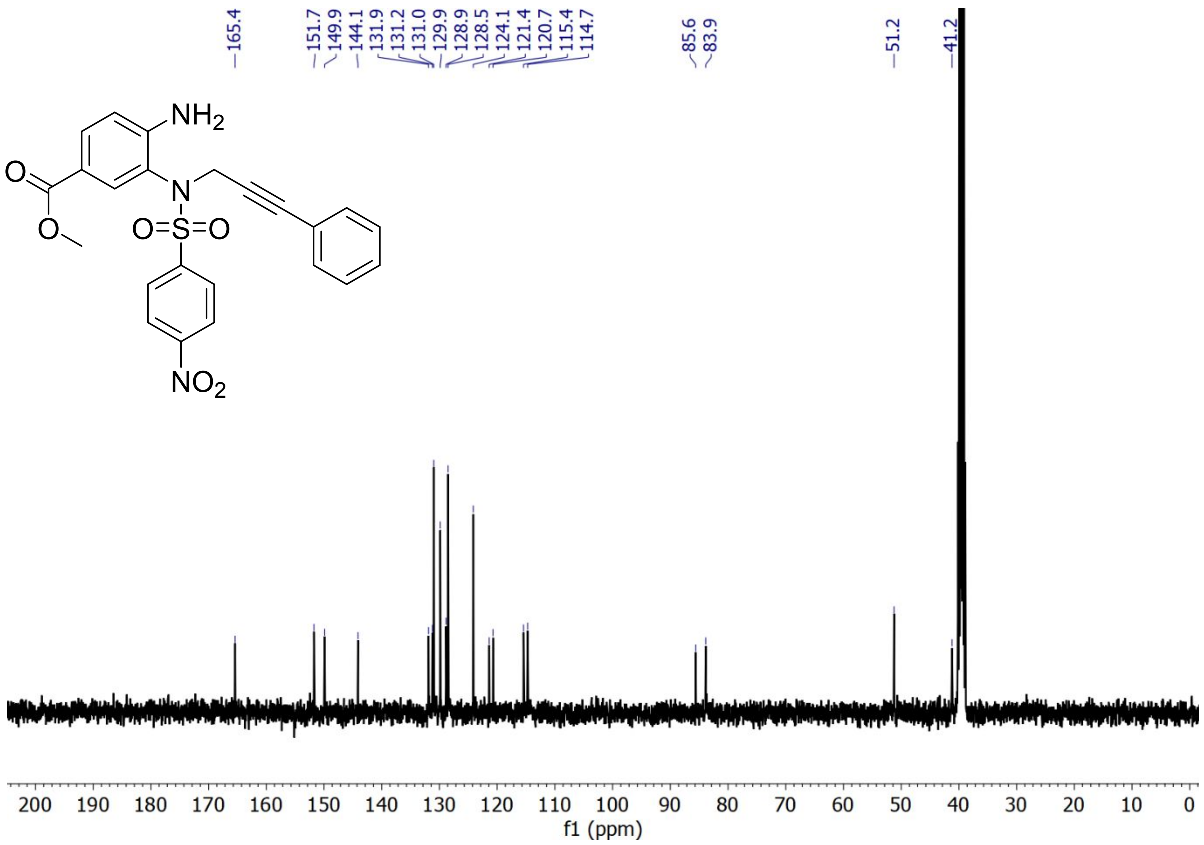

^{13}C NMR spectrum of **10f** (101 MHz, DMSO-d_6)

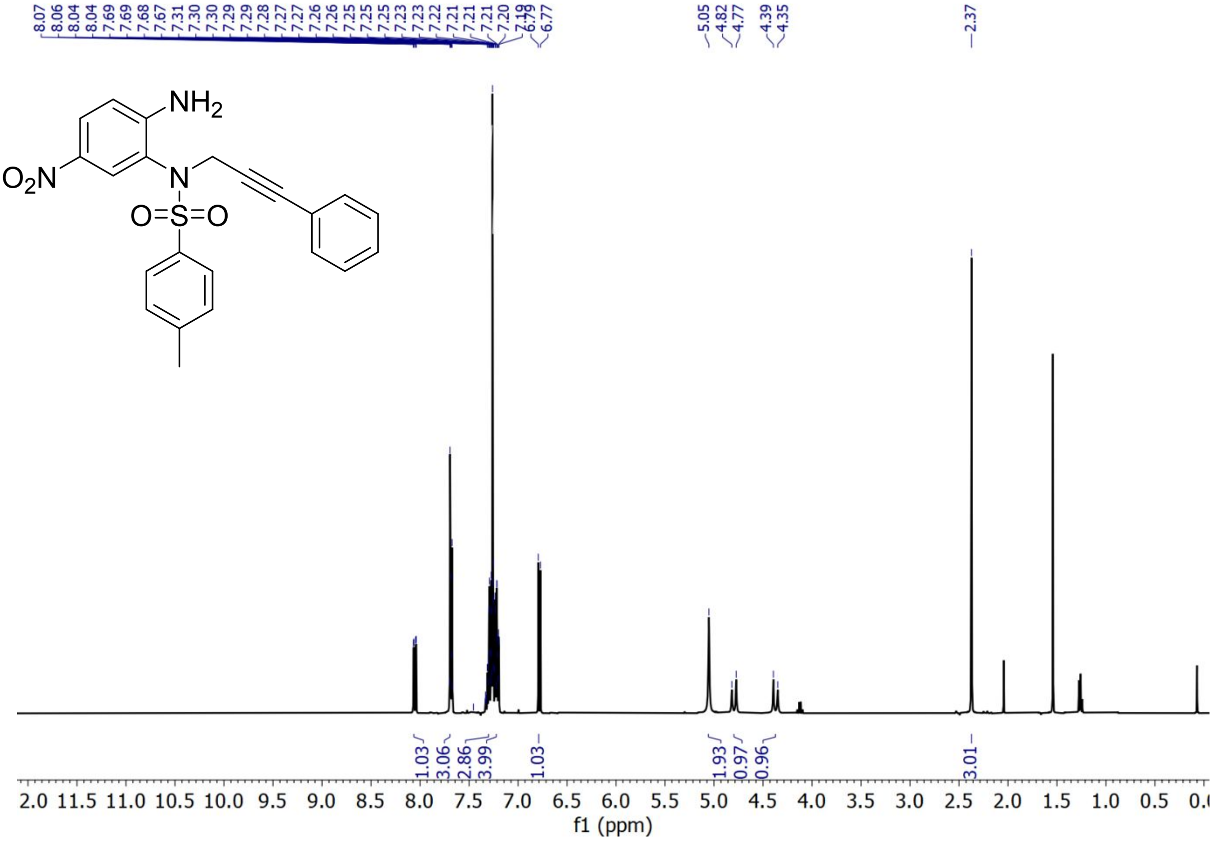

^{1}H NMR spectrum of **10g** (400 MHz, $CDCl_3$) signal at 1.27 ppm belongs to residual DIHD; yield of product **10g** was calculated excluding the mass of DIHD

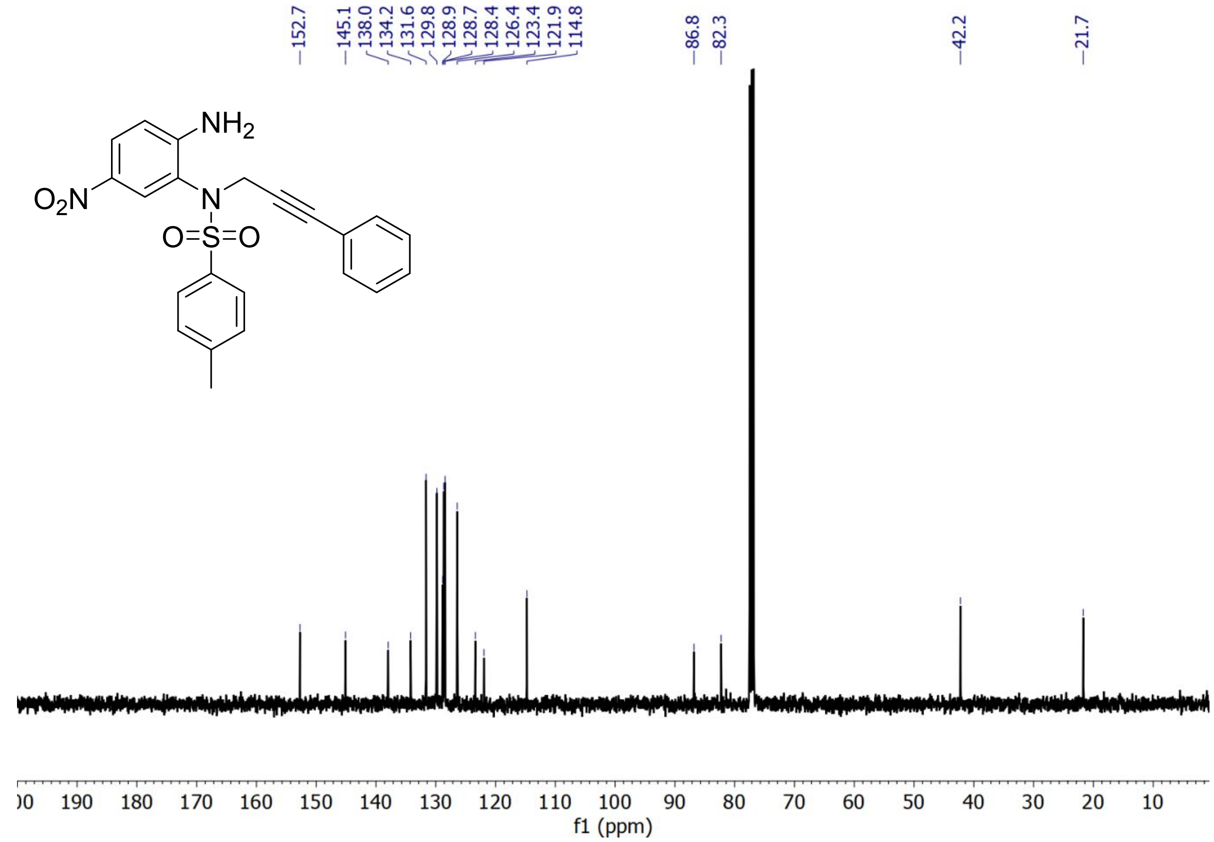

^{13}C NMR spectrum of **10g** (101 MHz, $CDCl_3$)

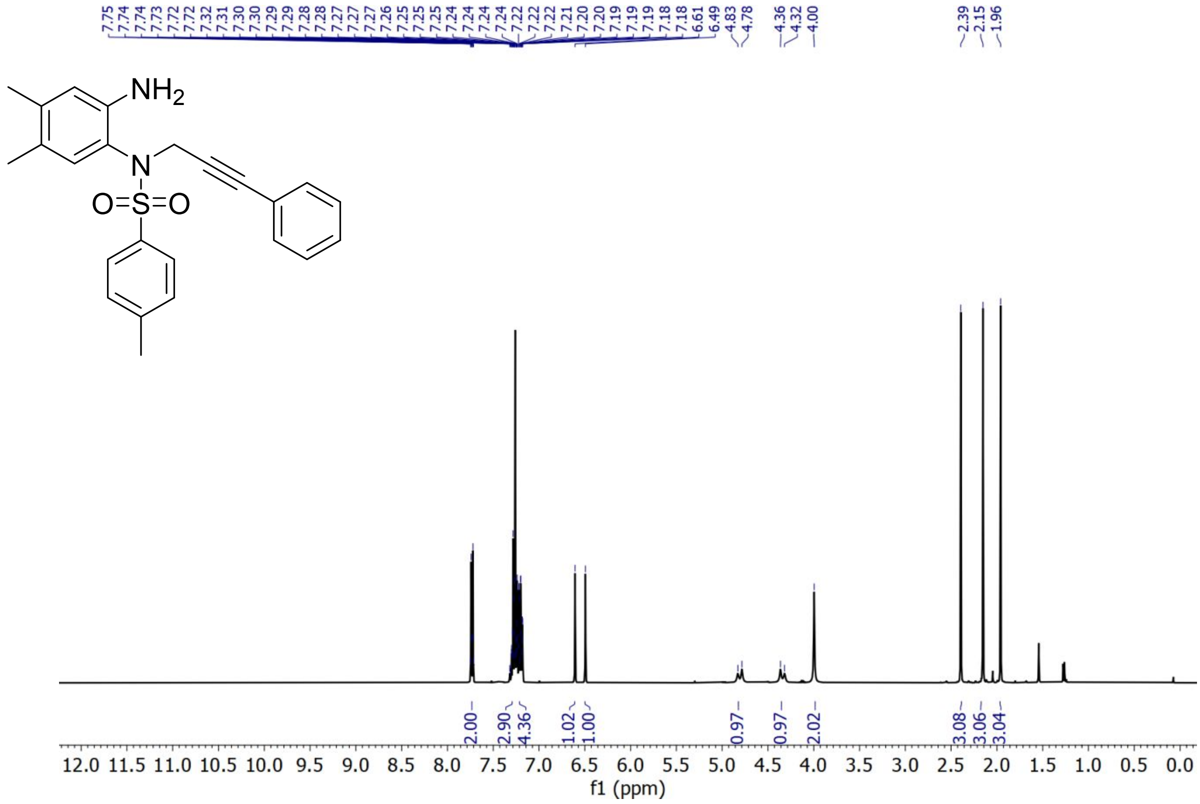

^{1}H NMR spectrum of **10h** (400 MHz, $CDCl_3$) signal at 1.27 ppm belongs to residual DIHD; yield of product **10h** was calculated excluding the mass of DIHD

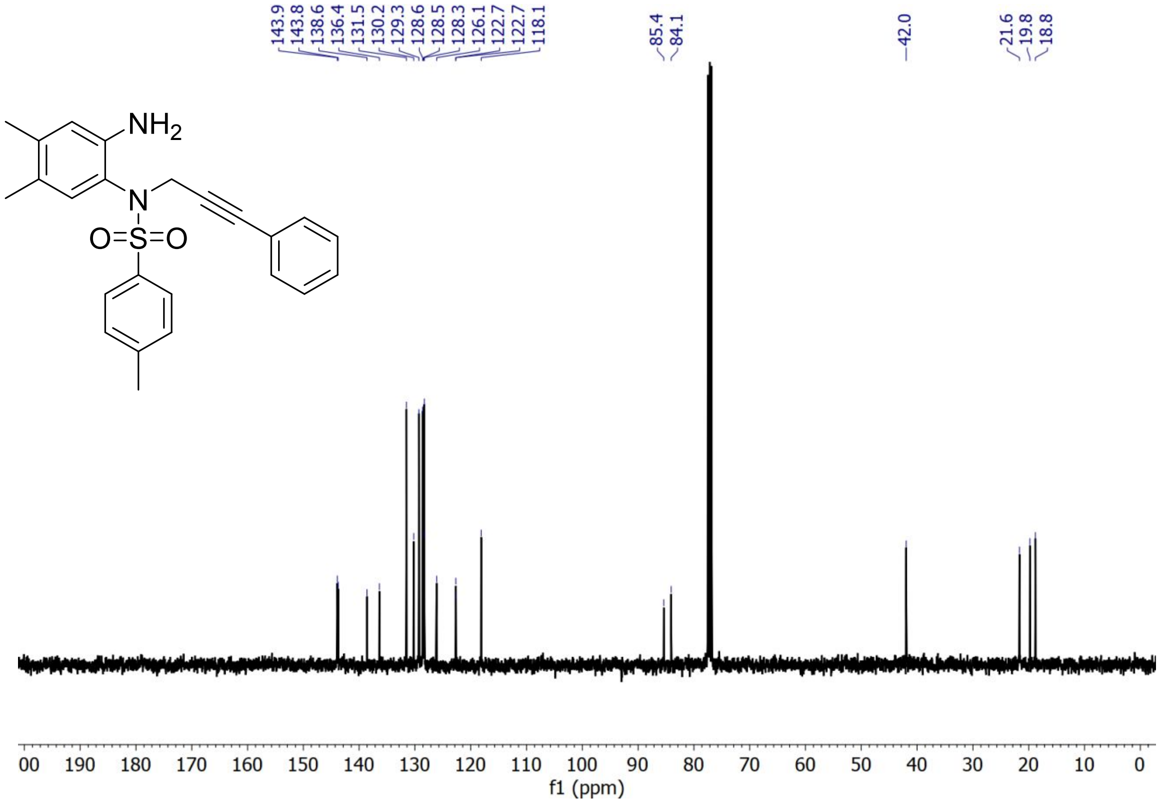

^{13}C NMR spectrum of **10h** (101 MHz, $CDCl_3$)

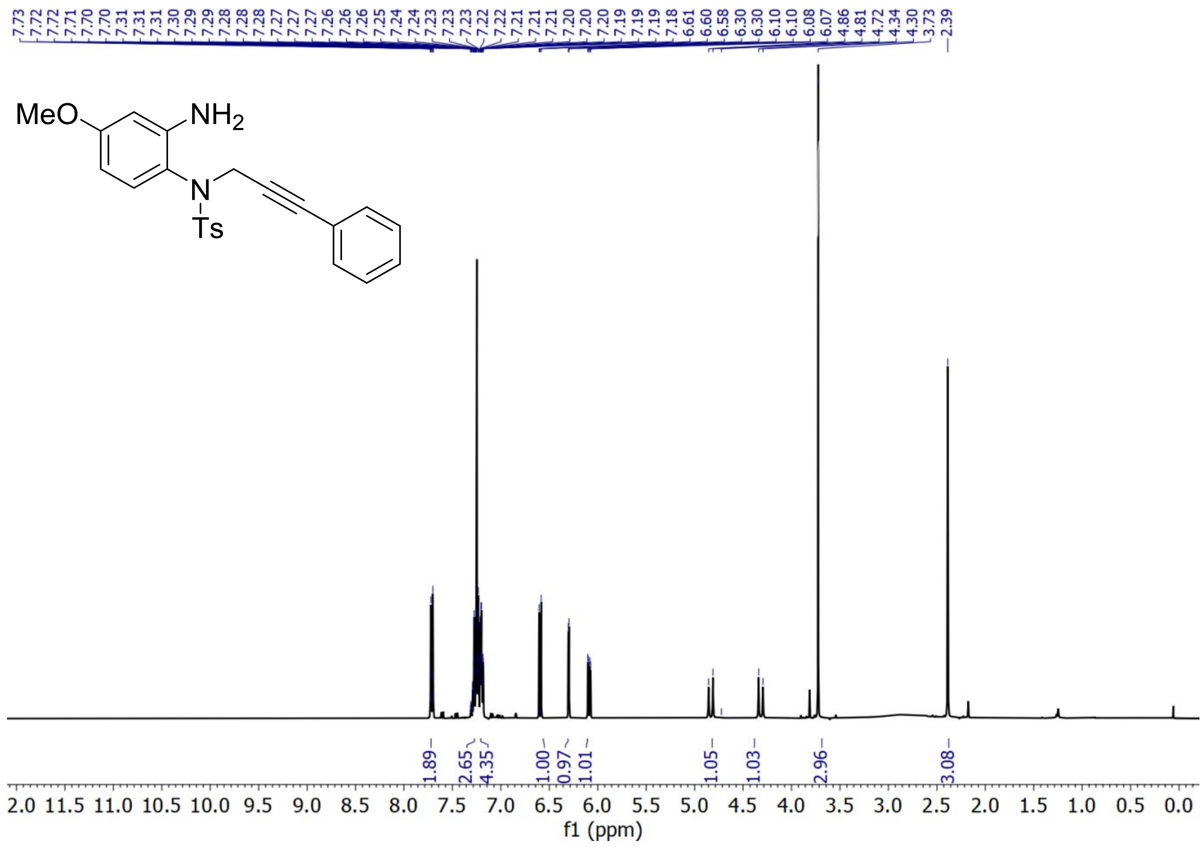

^{1}H NMR spectrum of **10i** (400 MHz, $CDCl_3$)

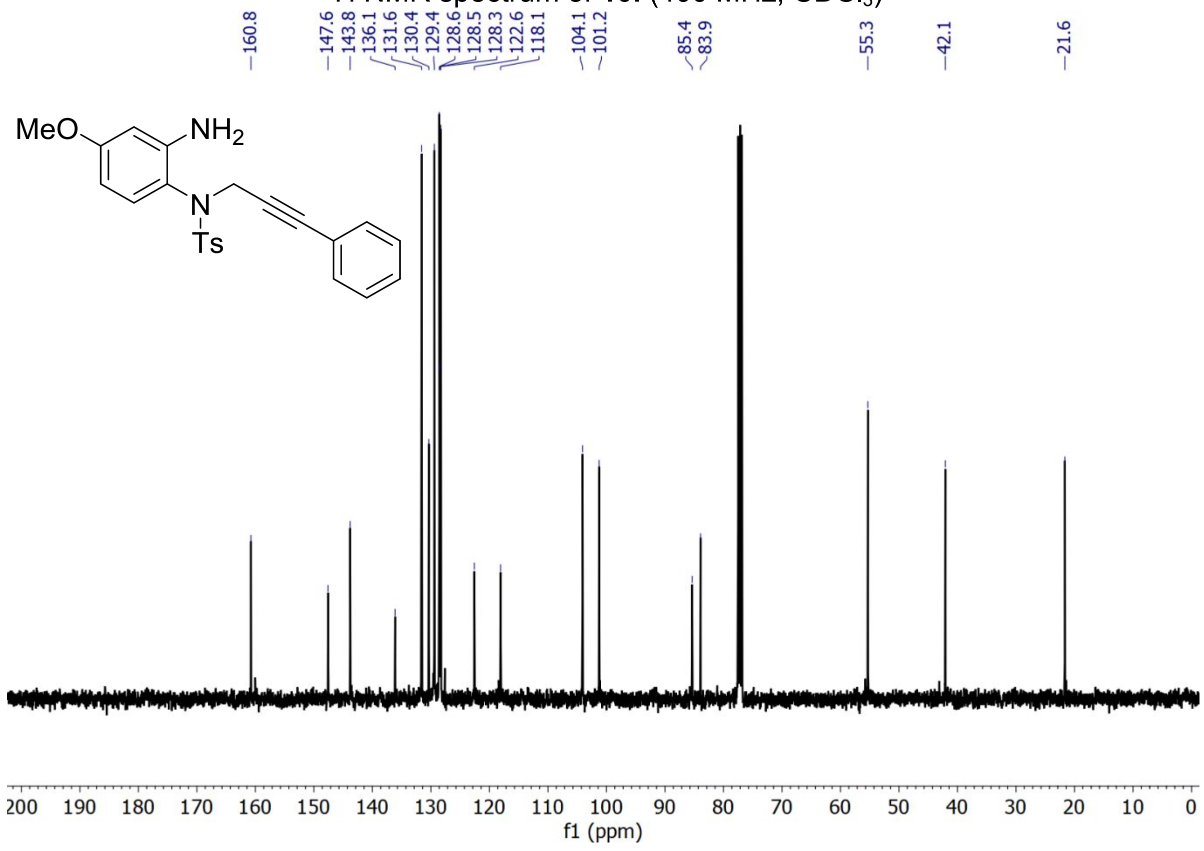

^{13}C NMR spectrum of **10i** (101 MHz, $CDCl_3$)

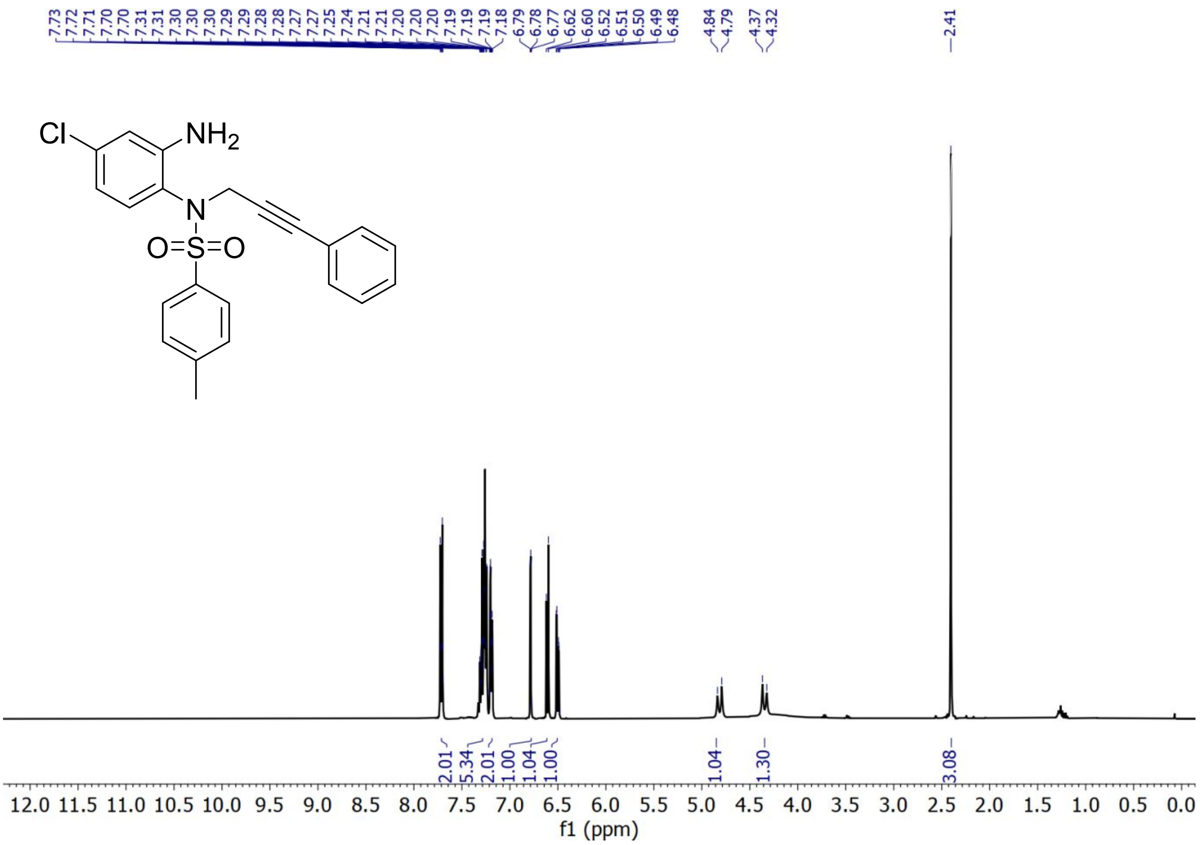

^{1}H NMR spectrum of **10j** (400 MHz, $CDCl_3$)

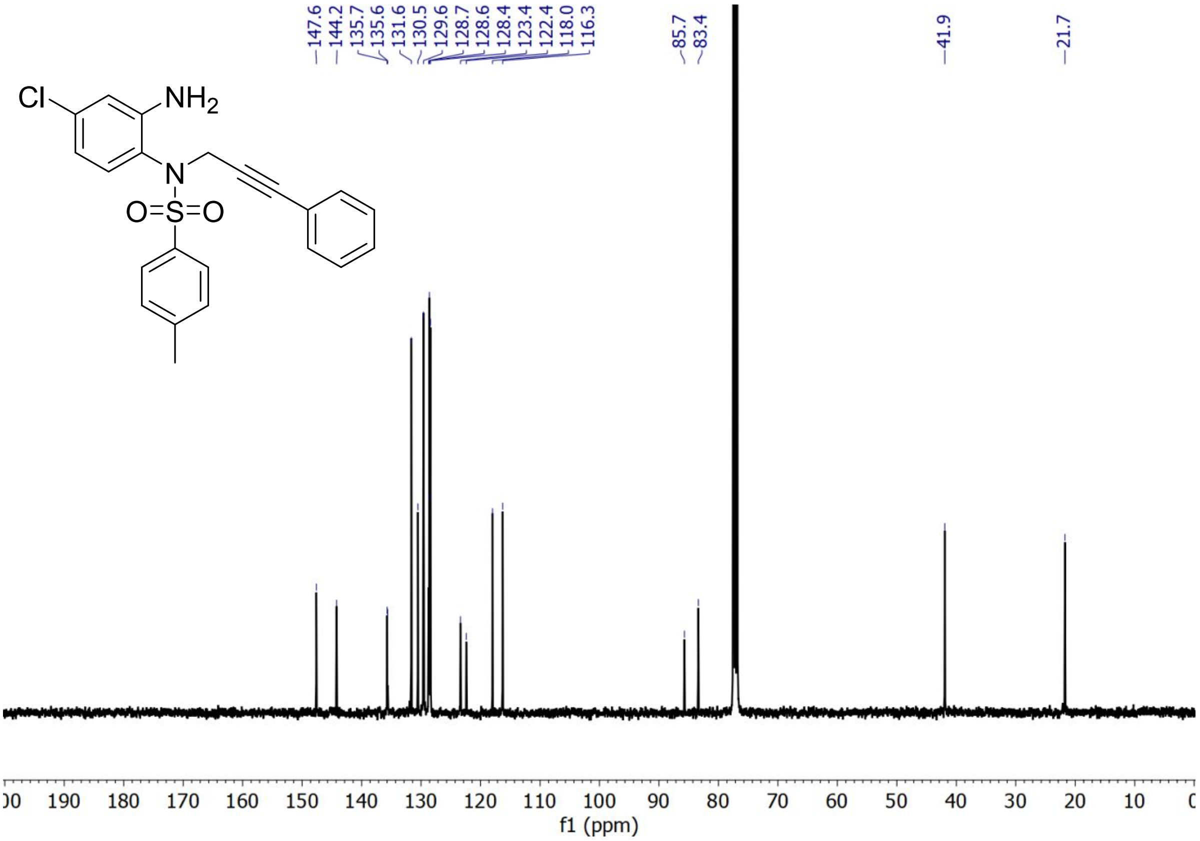

^{13}C NMR spectrum of **10j** (101 MHz, $CDCl_3$)

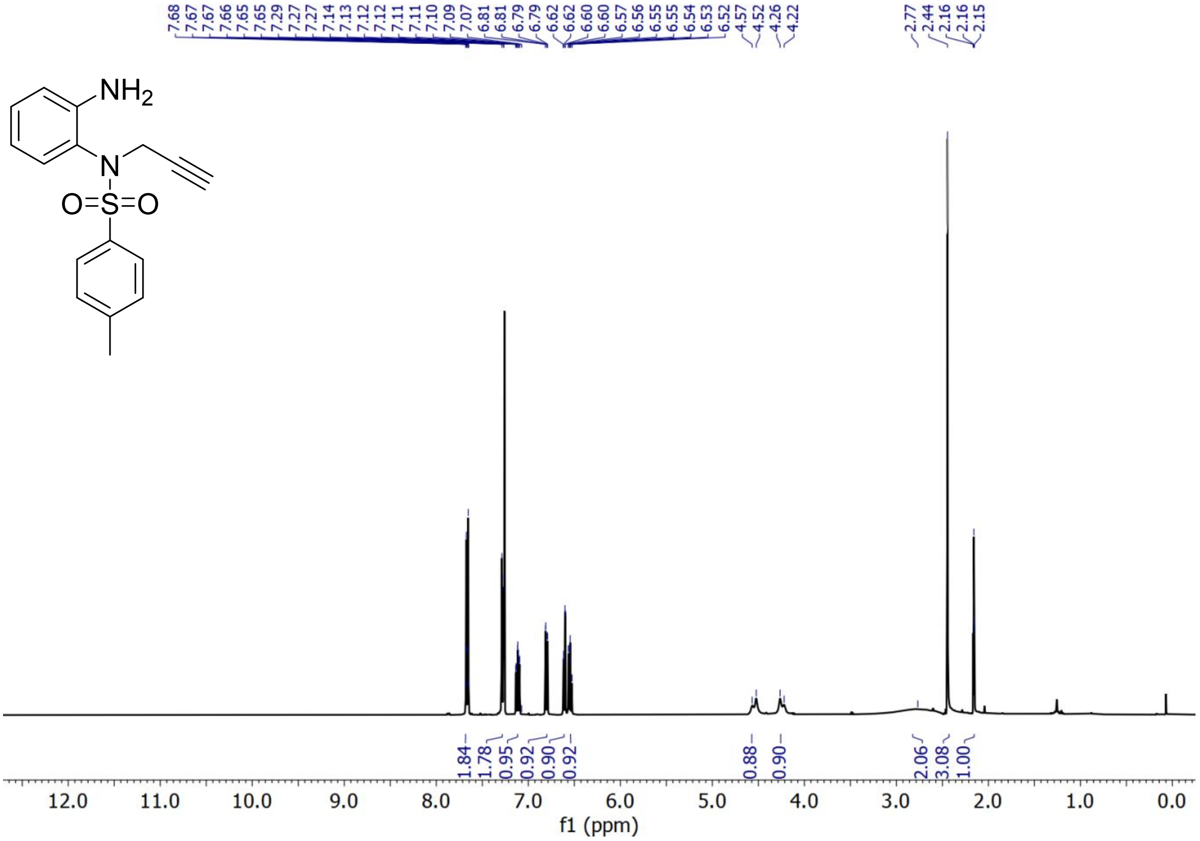

^{1}H NMR spectrum of **10k** (400 MHz, $CDCl_3$)

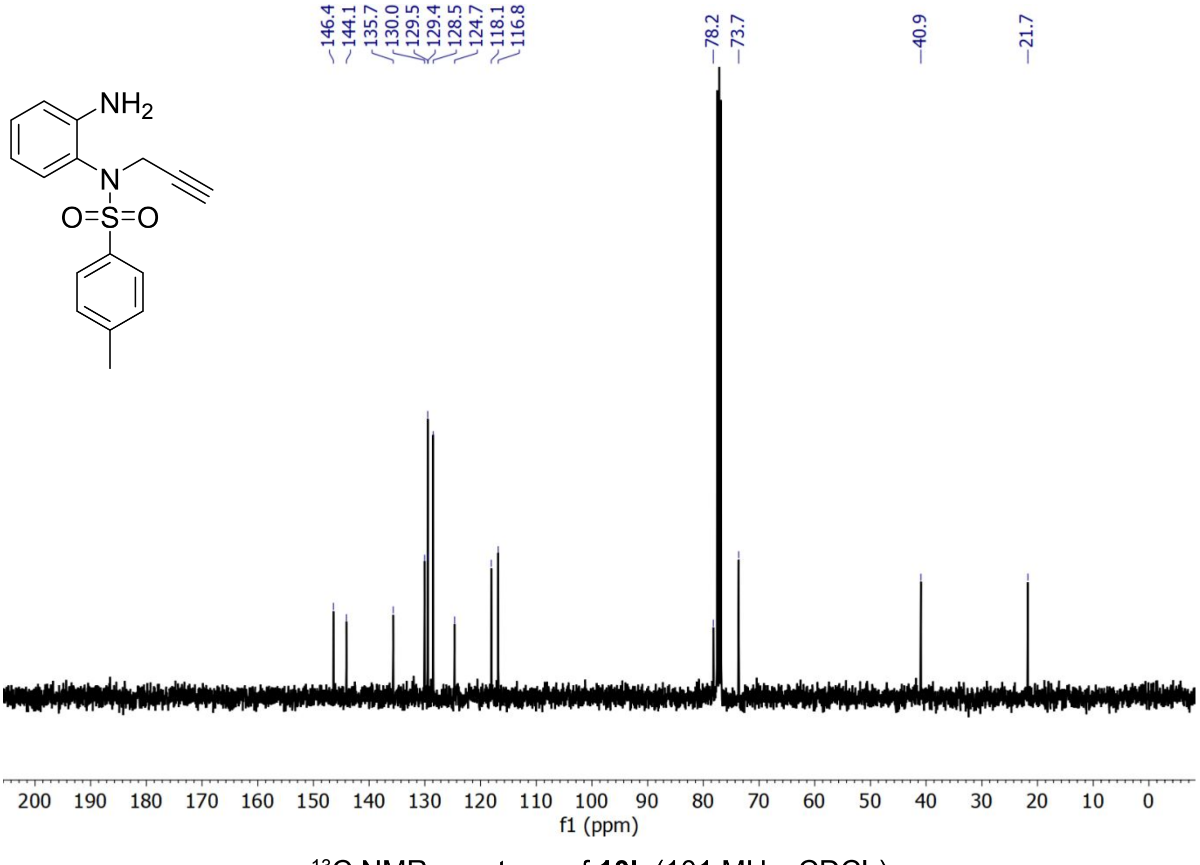

^{13}C NMR spectrum of **10k** (101 MHz, $CDCl_3$)

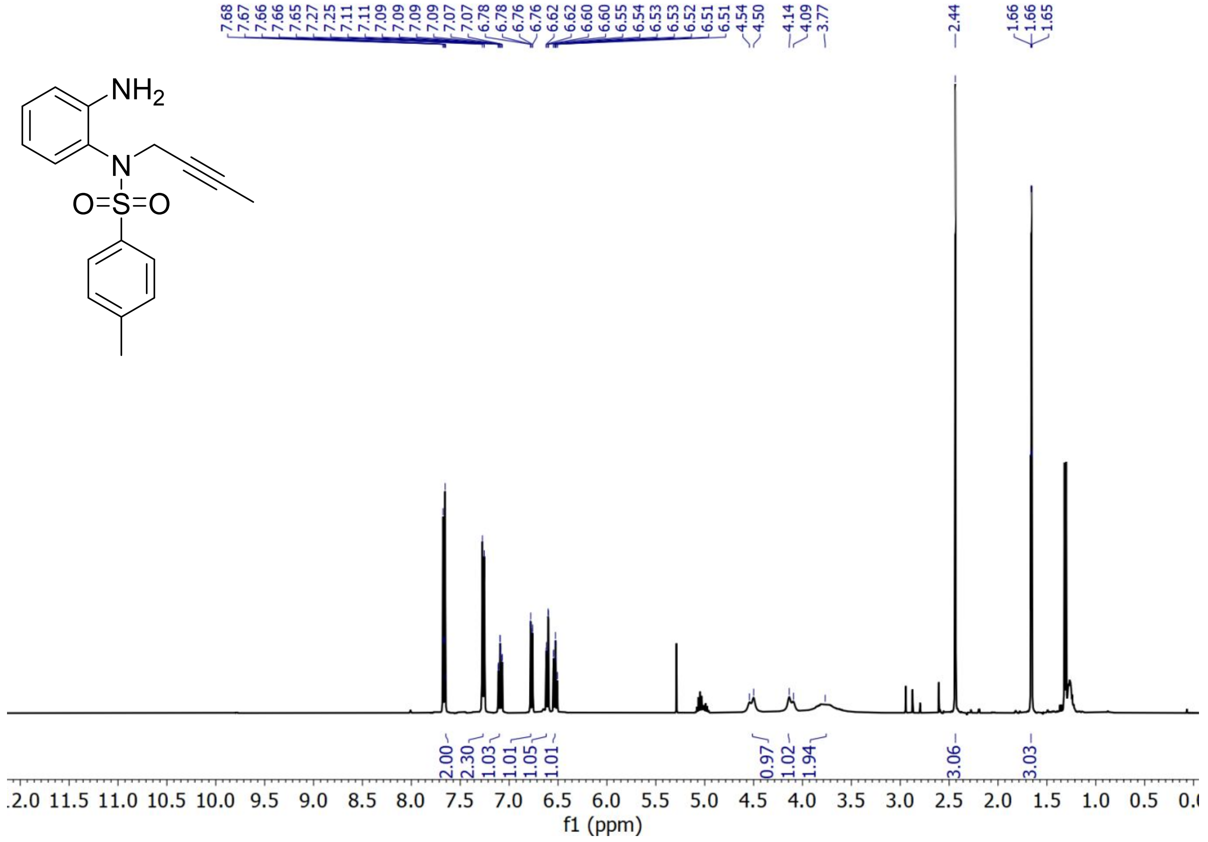

^{1}H NMR spectrum of **10l** (400 MHz, $CDCl_3$) signal at 1.3 ppm belongs to residual DIHD; yield of product **10l** was calculated excluding the mass of DIHD

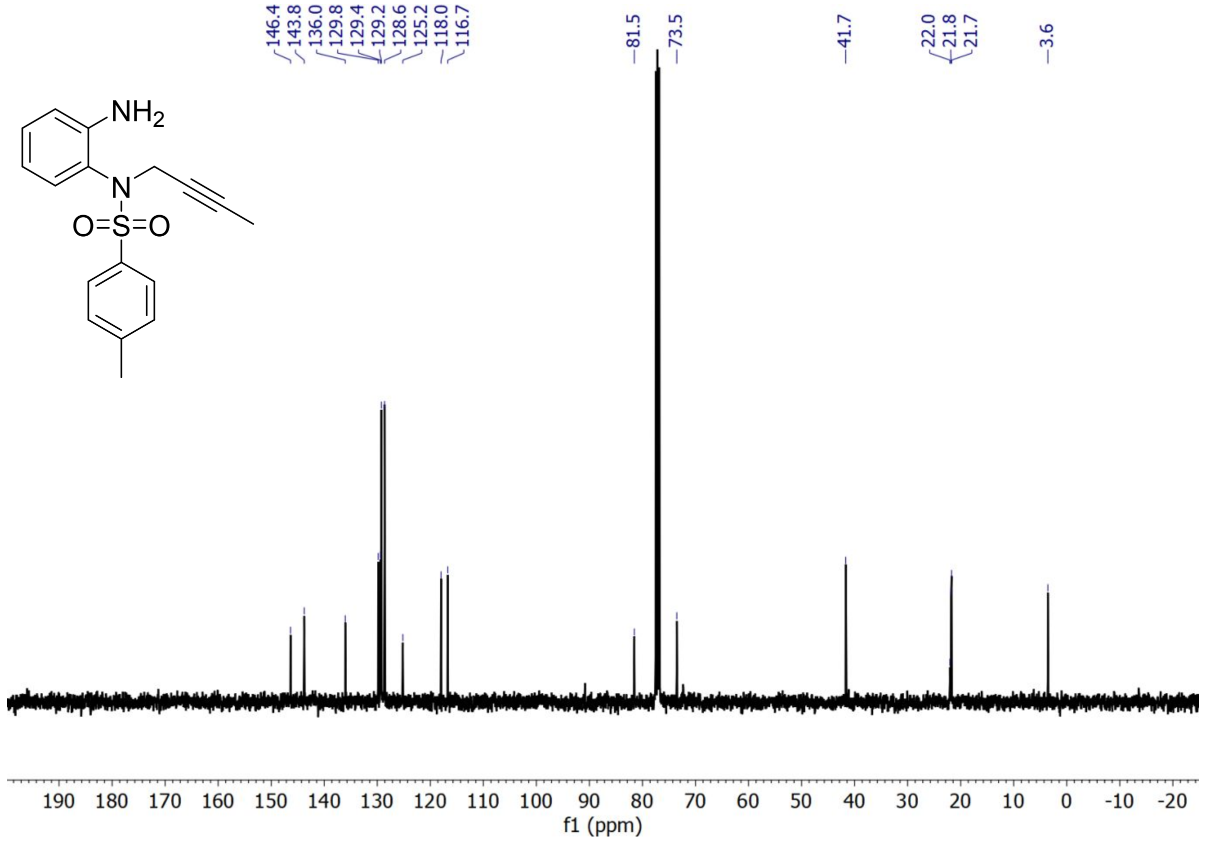

^{13}C NMR spectrum of **10l** (101 MHz, $CDCl_3$)

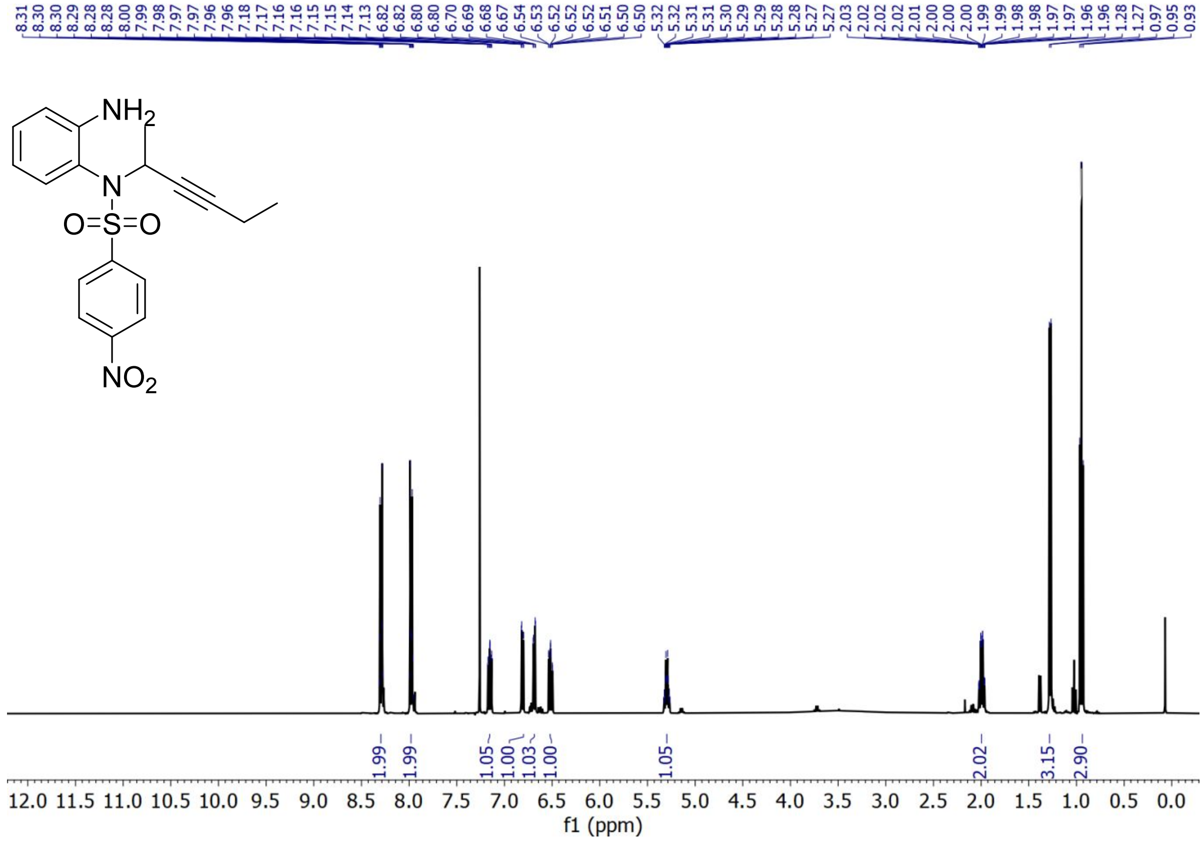

^{1}H NMR spectrum of **10m** (400 MHz, $CDCl_3$), interfering signals belong to the second diastereoisomer

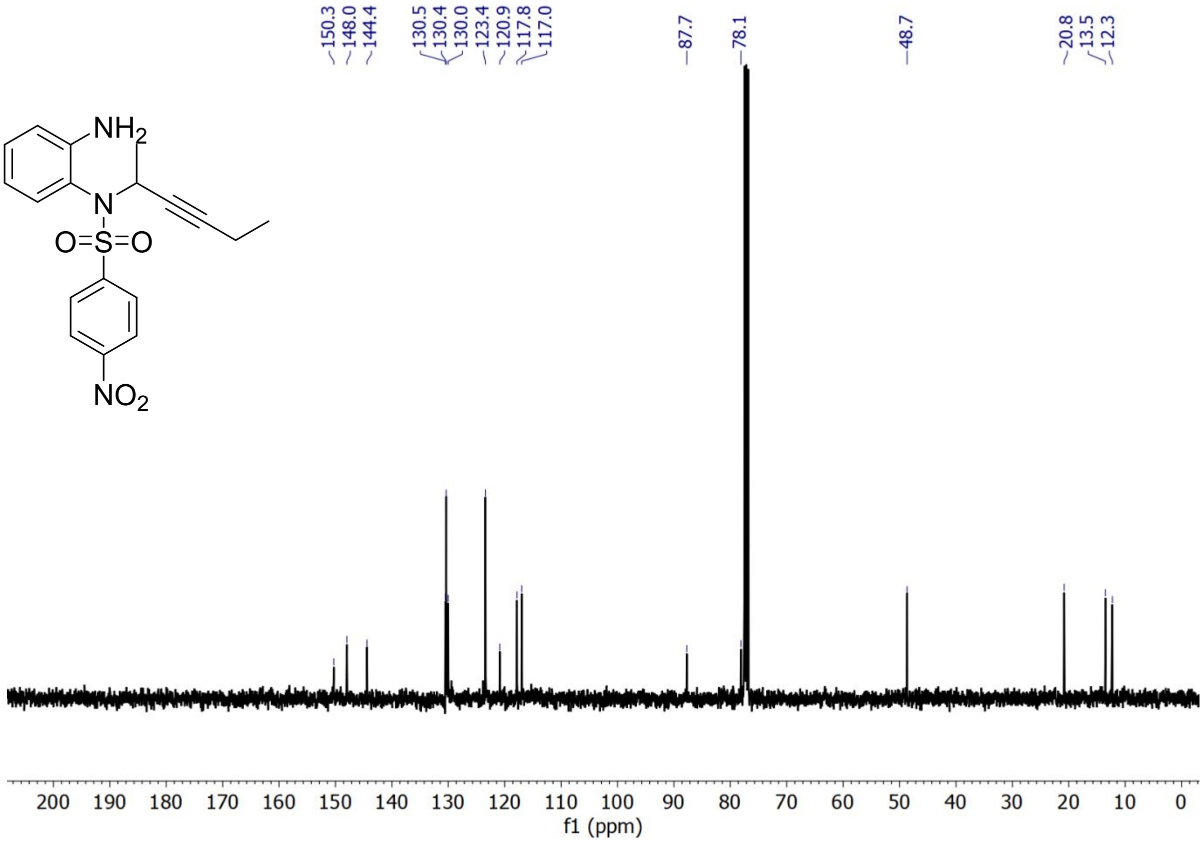

^{13}C NMR spectrum of **10m** (101 MHz, $CDCl_3$)

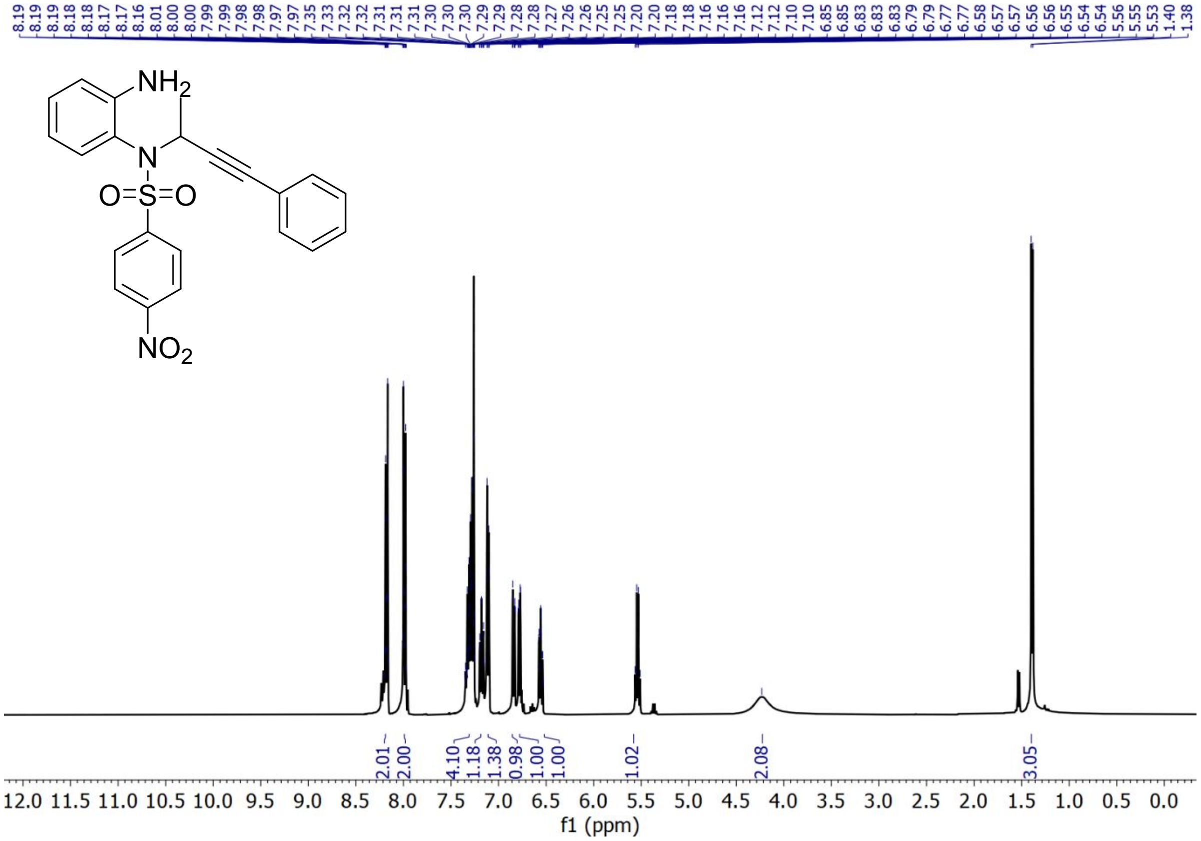

^{1}H NMR spectrum of **10n** (400 MHz, $CDCl_3$) interfering signals belong to the second diastereoisomer

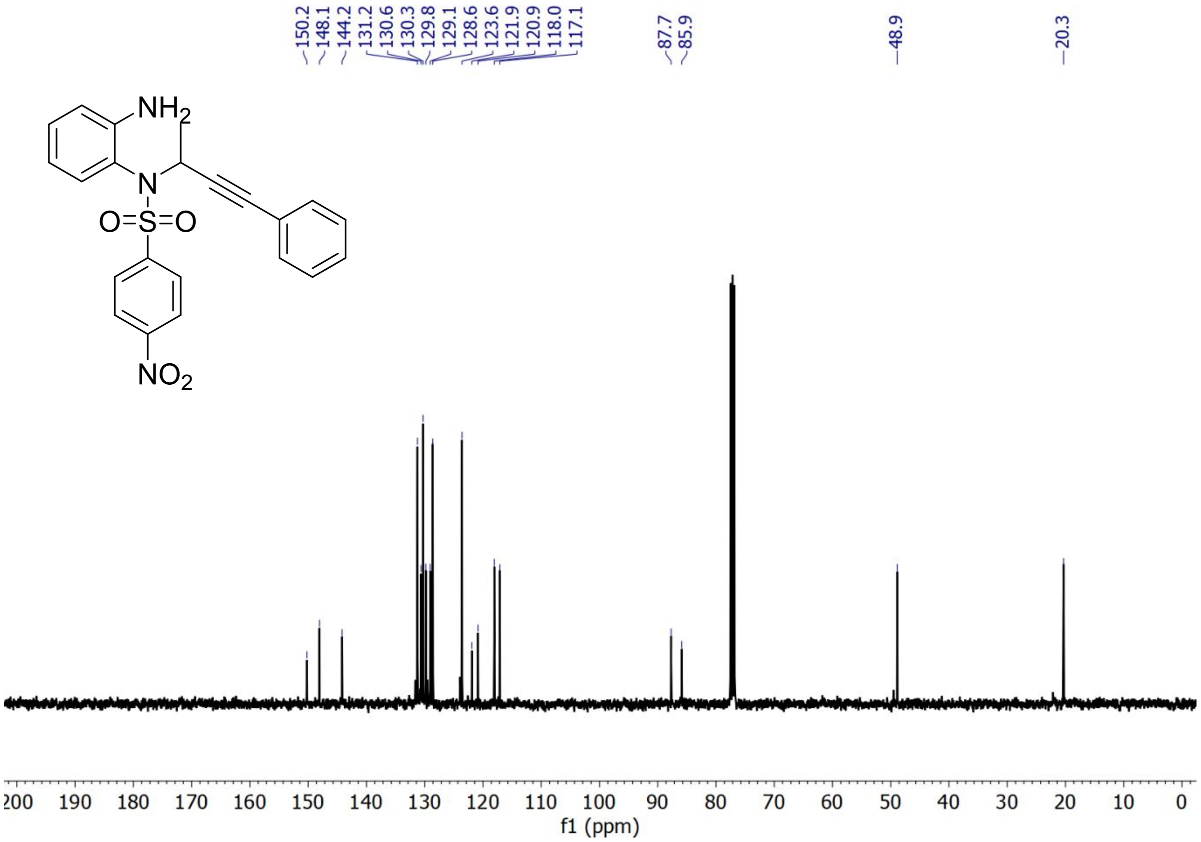

^{13}C NMR spectrum of **10n** (101 MHz, $CDCl_3$)

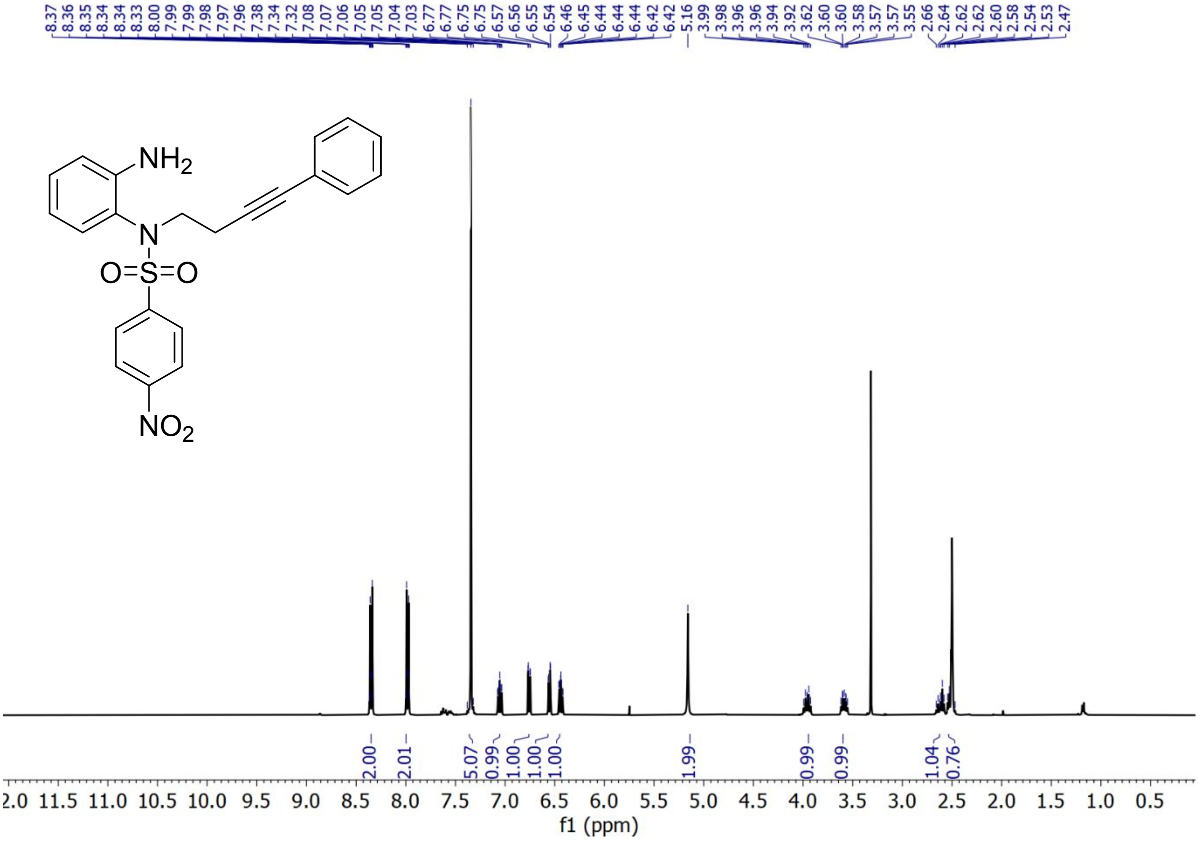

^{1}H NMR spectrum of **13a** (400 MHz, DMSO-d_6)

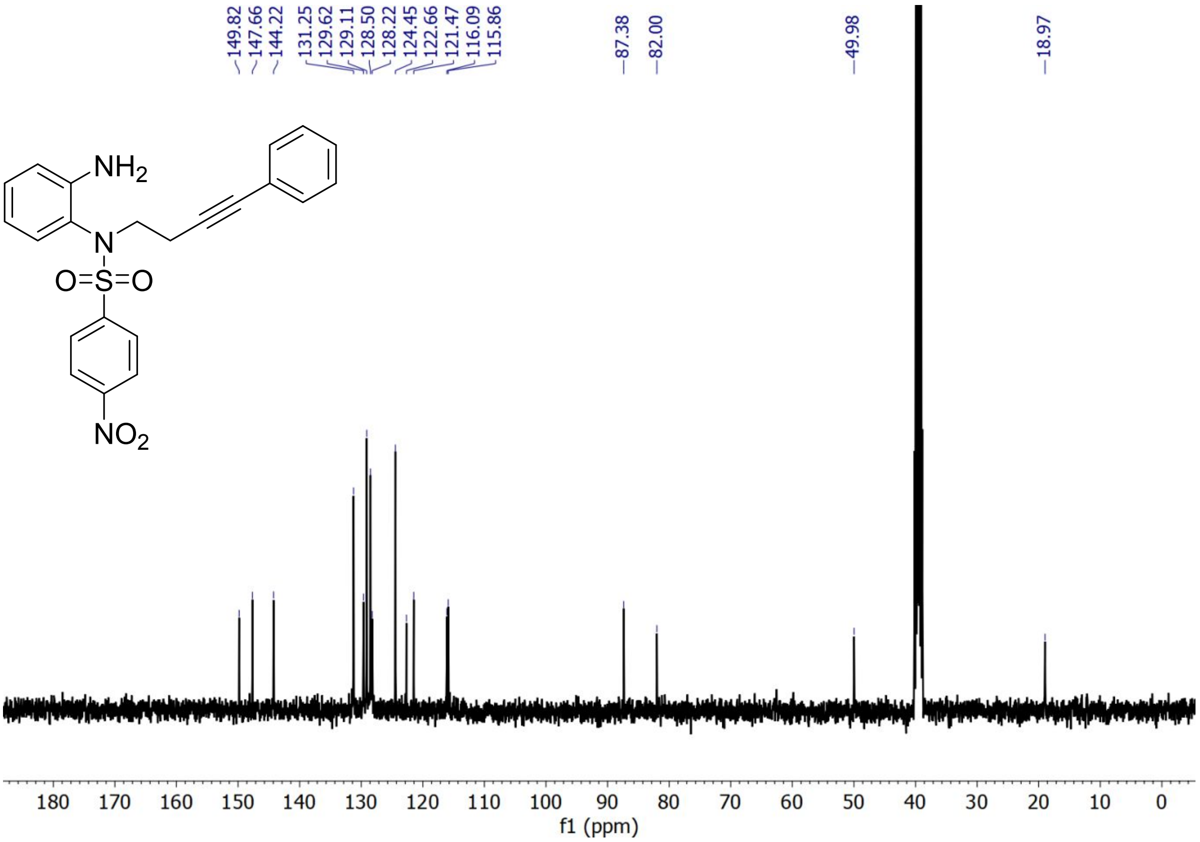

^{13}C NMR spectrum of **13a** (101 MHz, DMSO-d_6)

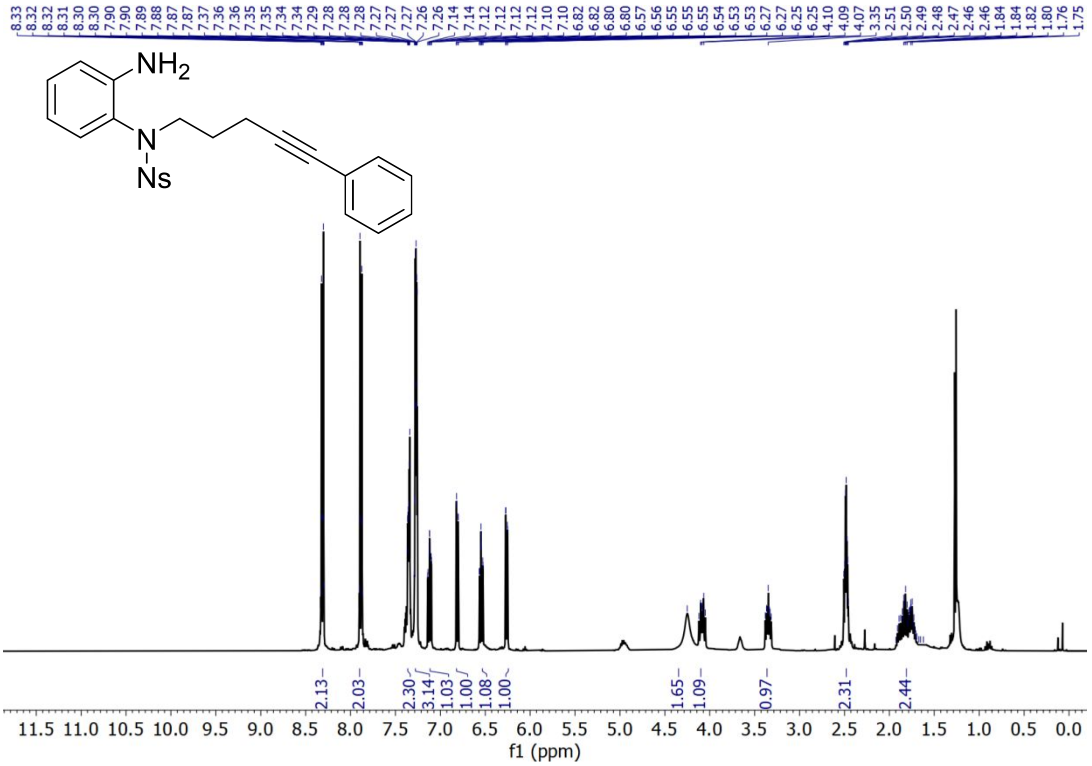

^{1}H NMR spectrum of **13b** (400 MHz, $CDCl_3$) - signal at 4.95 ppm and 1.26 ppm belongs to residual DIHD, signal at 3.66 ppm belongs to starting 5-phenylpent-4-yn-1-ol; yield of product **13b** was calculated excluding the mass of impurities

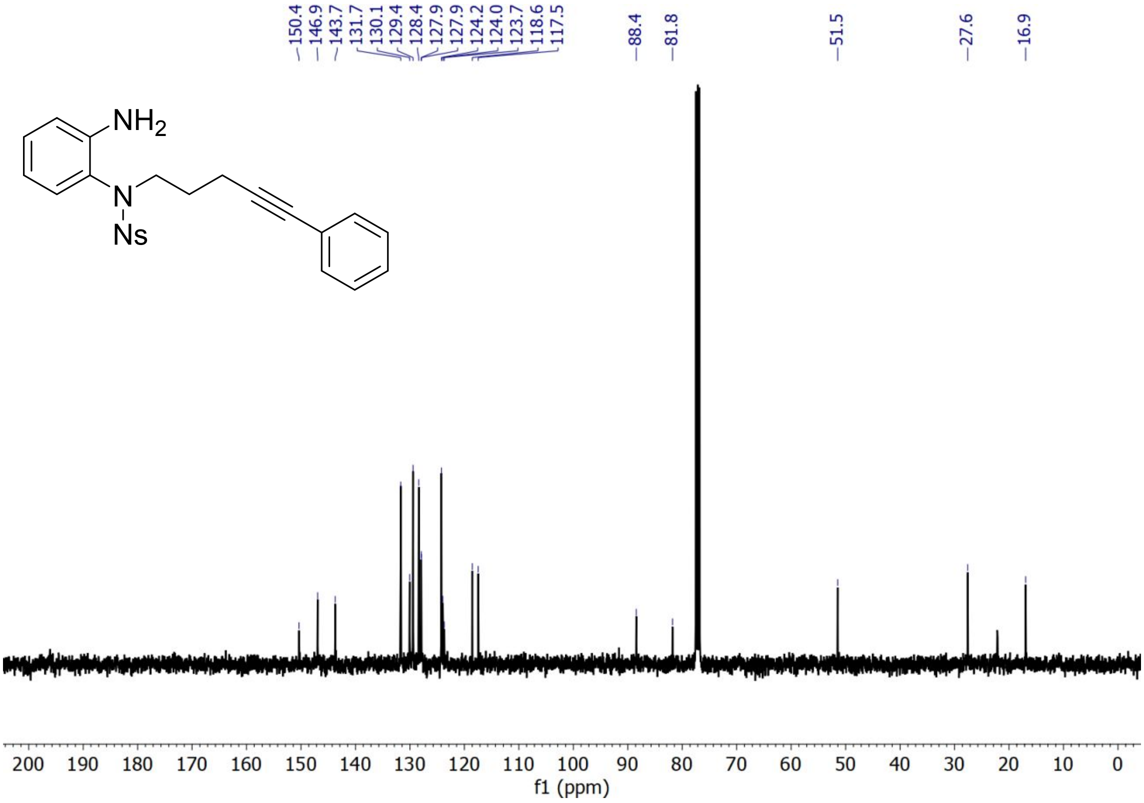

^{13}C NMR spectrum of **13b** (101 MHz, $CDCl_3$) - signal at 22.2 ppm belongs to residual DIHD

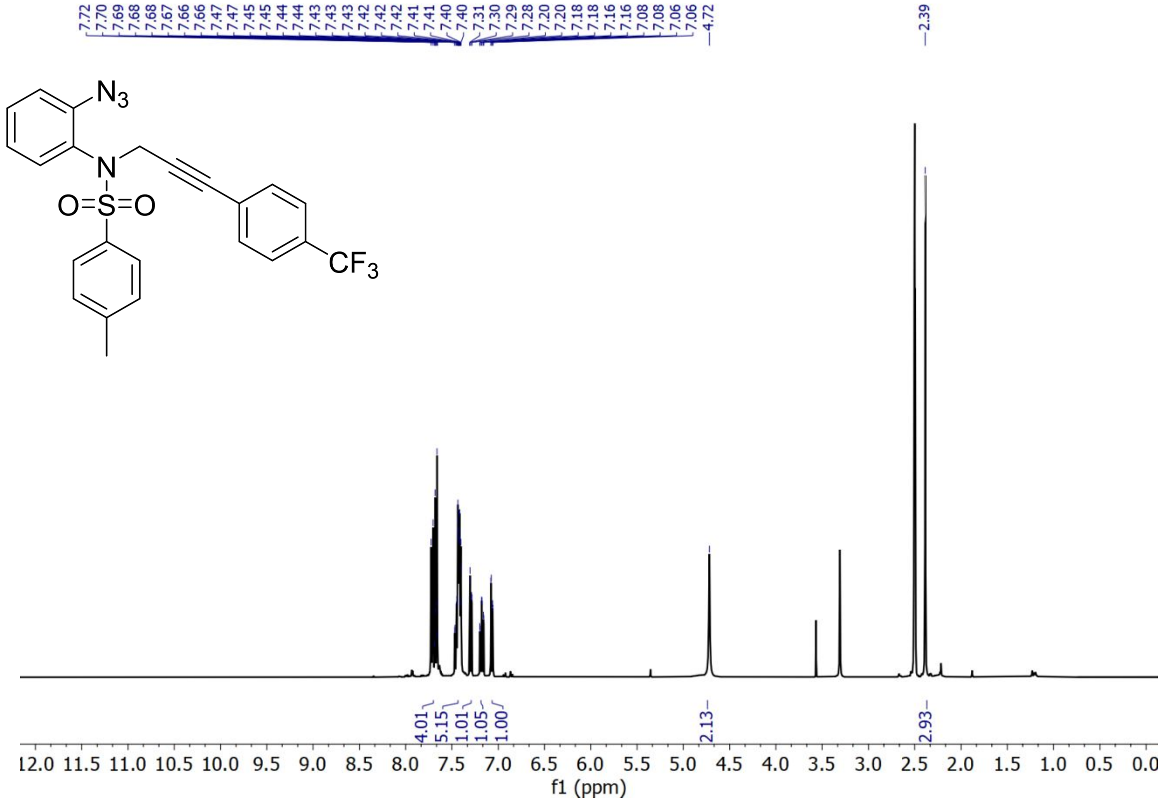

^{1}H NMR spectrum of **11a** (400 MHz, DMSO-d_6) - signal at 3.57 ppm belongs to residual 1,4-dioxane

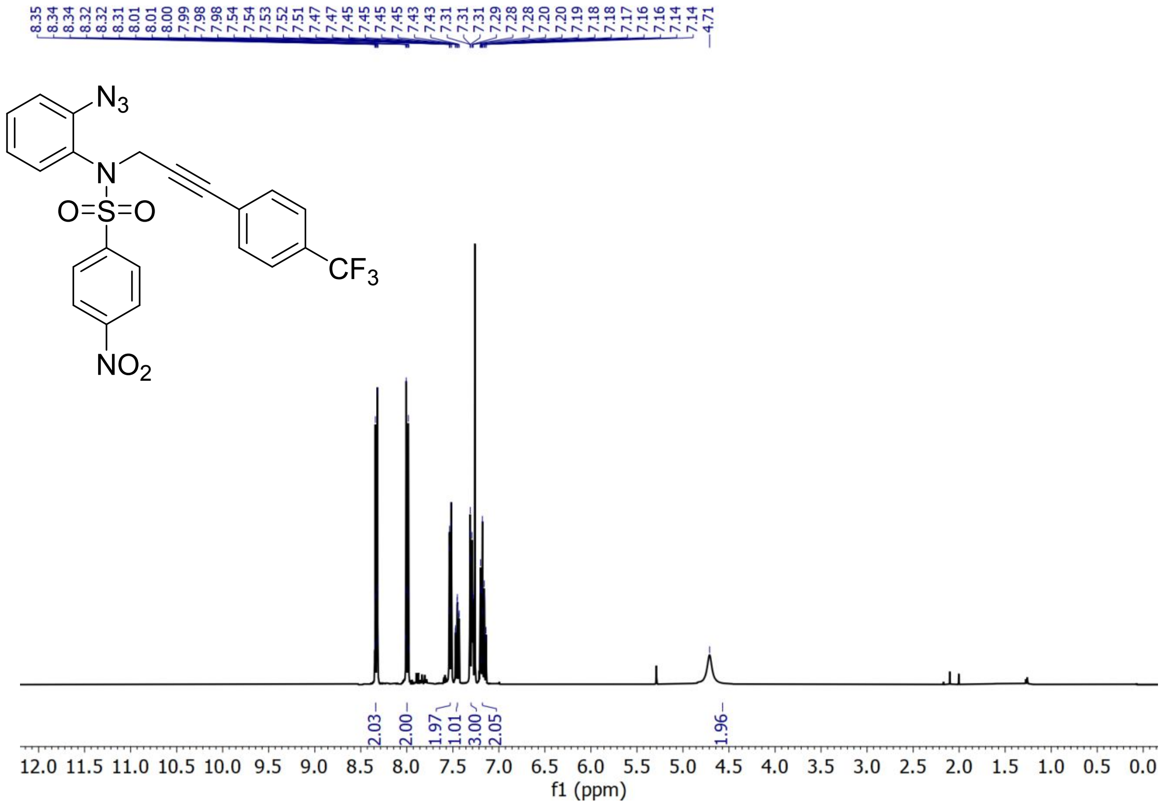

^{1}H NMR spectrum of **11b** (400 MHz, $CDCl_3$) - signal at 5.29 ppm belongs to CH_2 of cyclic form **12b**.

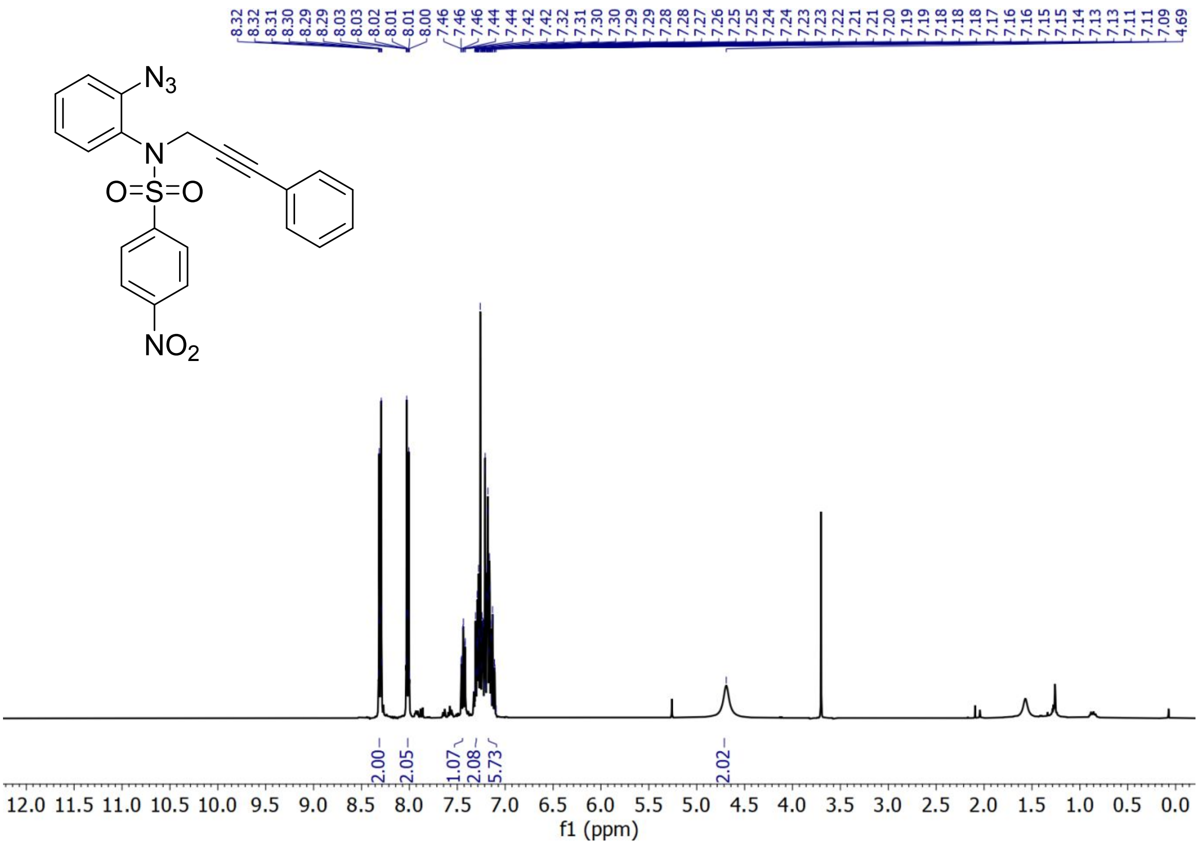

^{1}H NMR spectrum of **11c** (400 MHz, $CDCl_3$) - signal at 5.26 ppm belongs to CH_2 of cyclic form **12c**, signal at 3.71 ppm belongs to residual 1,4-dioxane

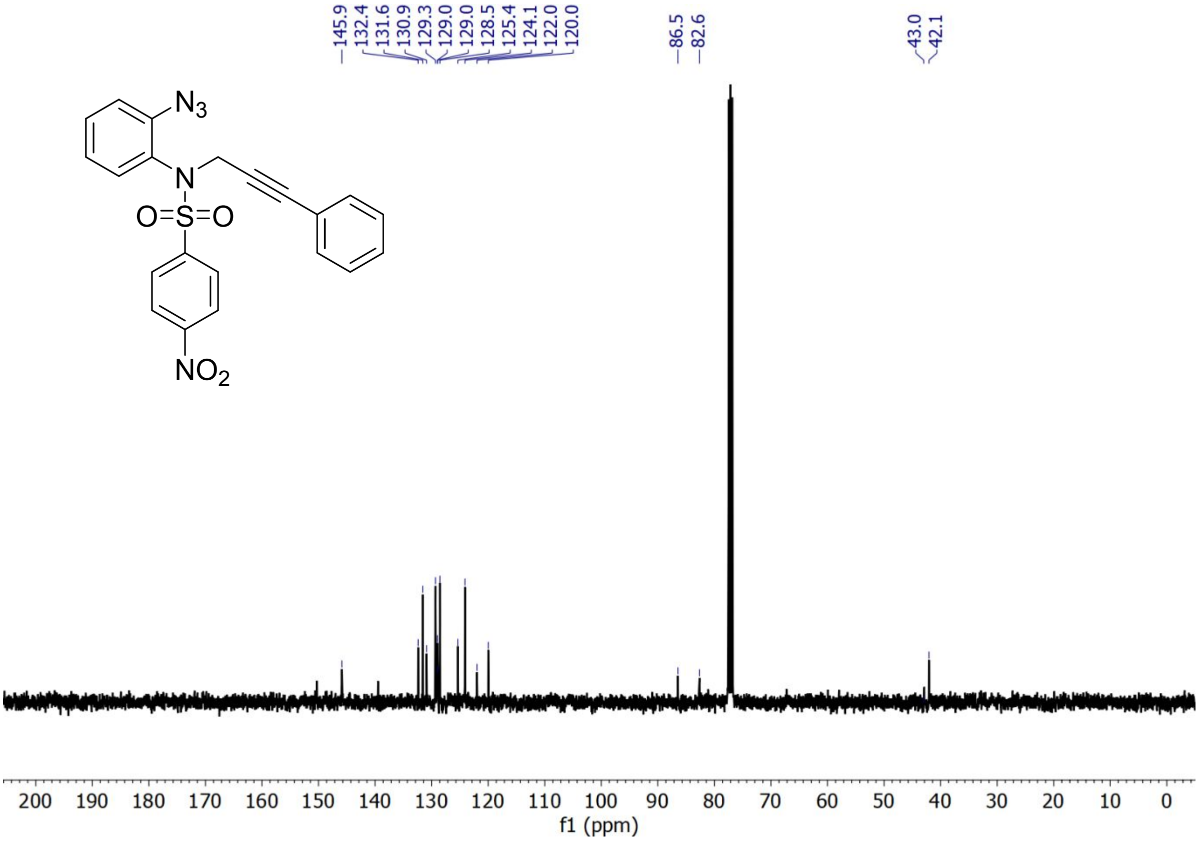

^{13}C NMR spectrum of **11c** (101 MHz, $CDCl_3$)

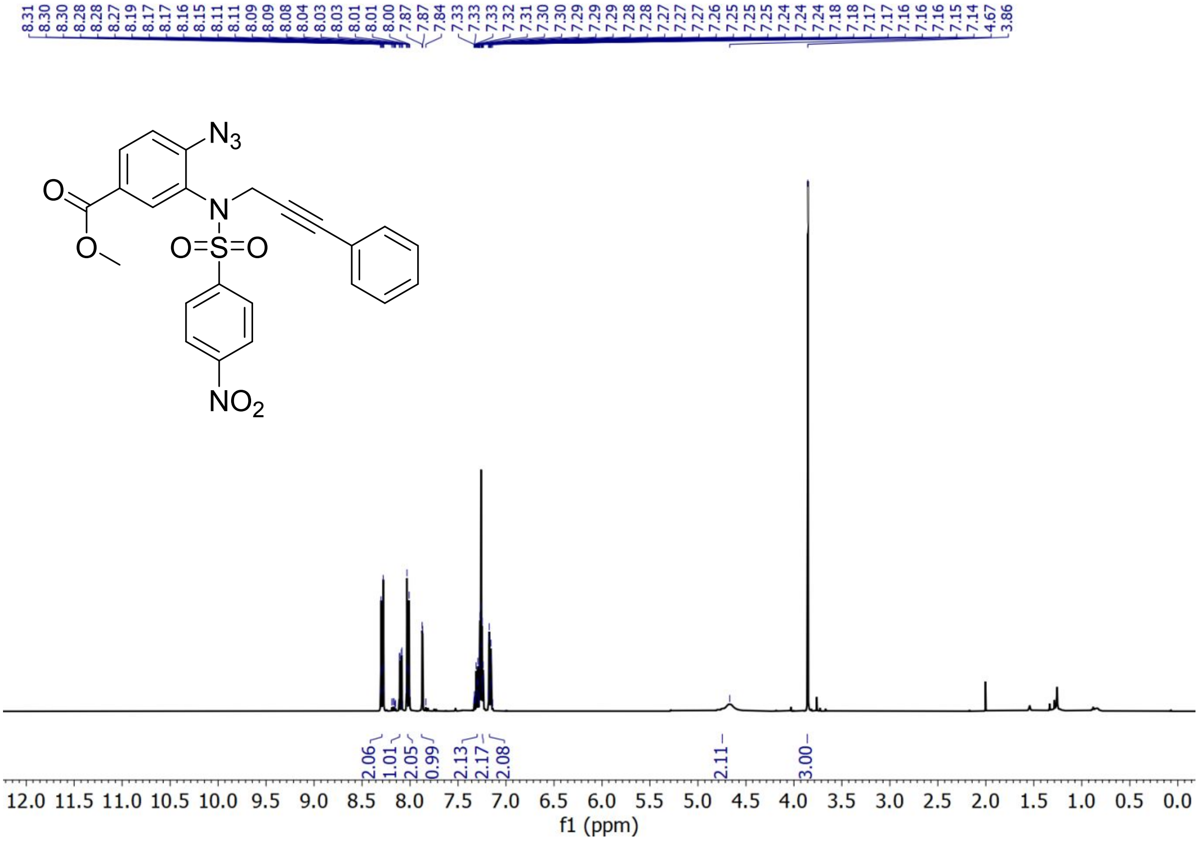

^{1}H NMR spectrum of **11f** (400 MHz, $CDCl_3$)

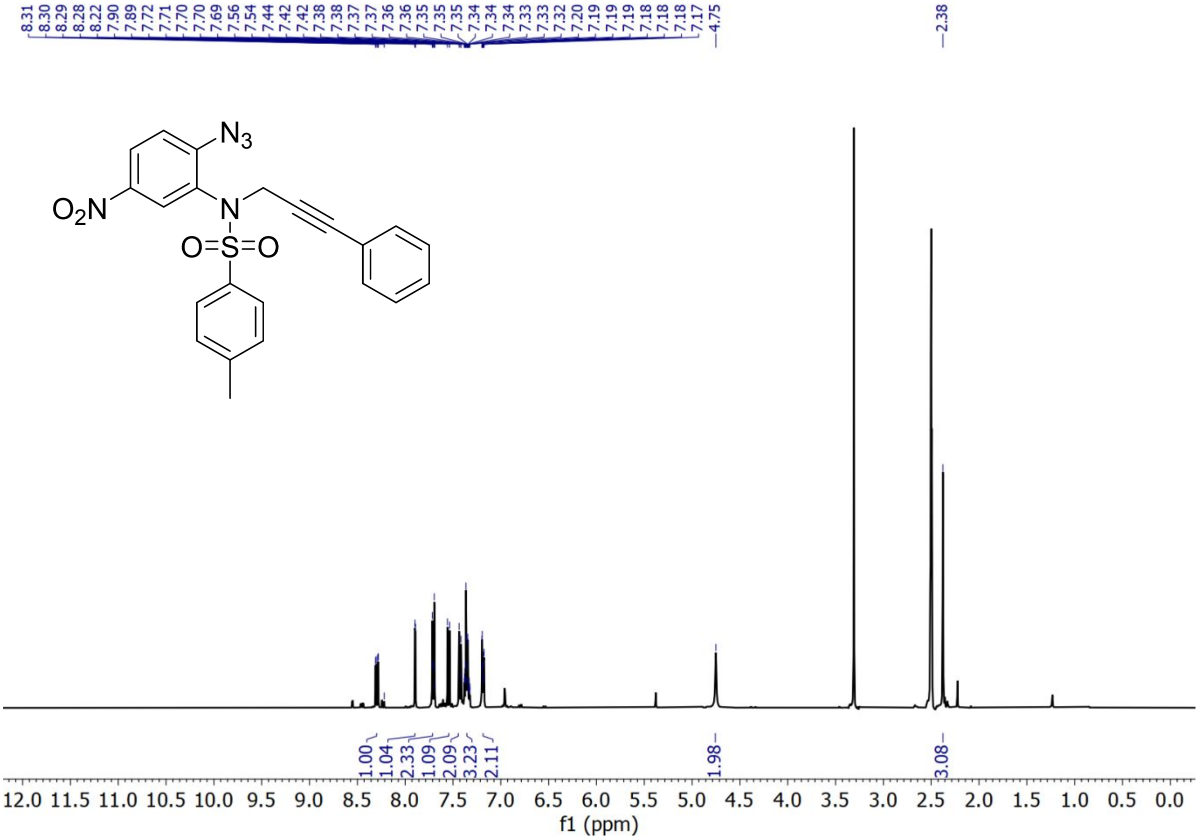

^{1}H NMR spectrum of **11g** (400 MHz, DMSO-d_6)

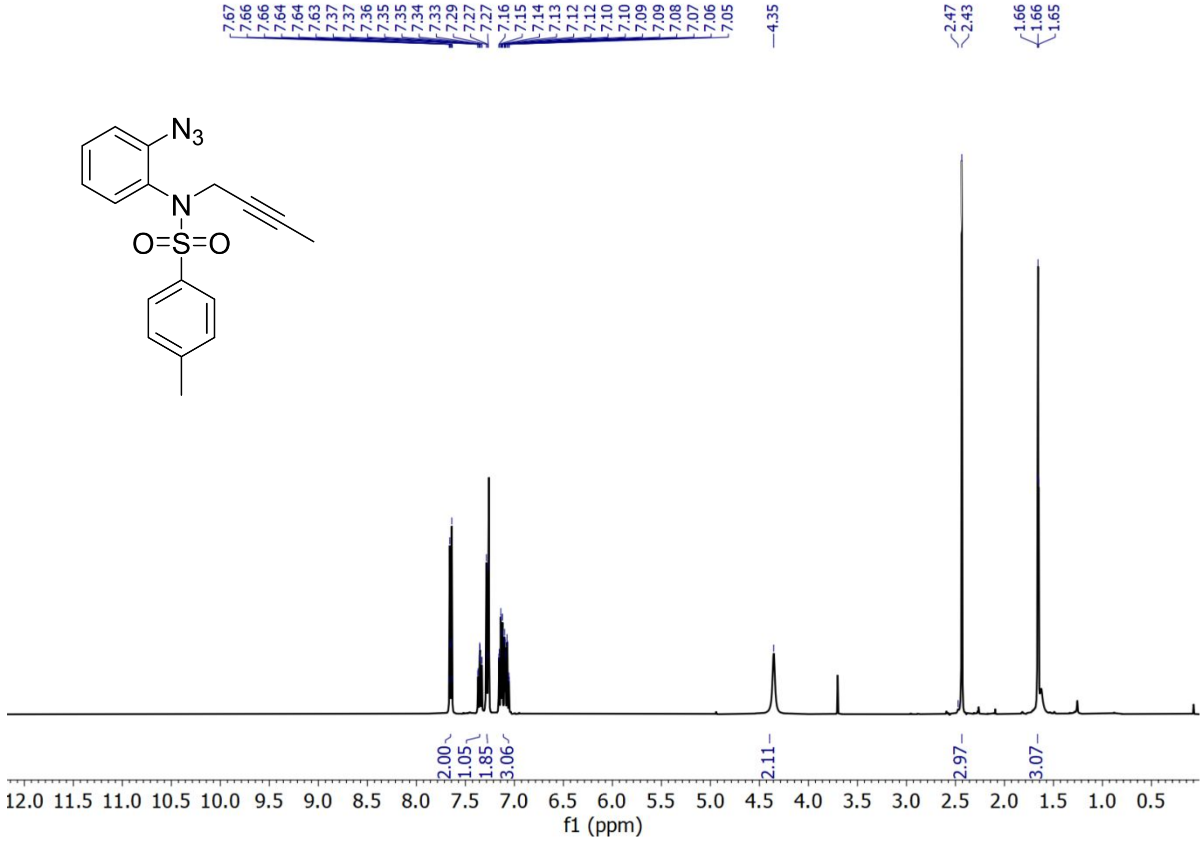

^{1}H NMR spectrum of **11l** (400 MHz, $CDCl_3$)

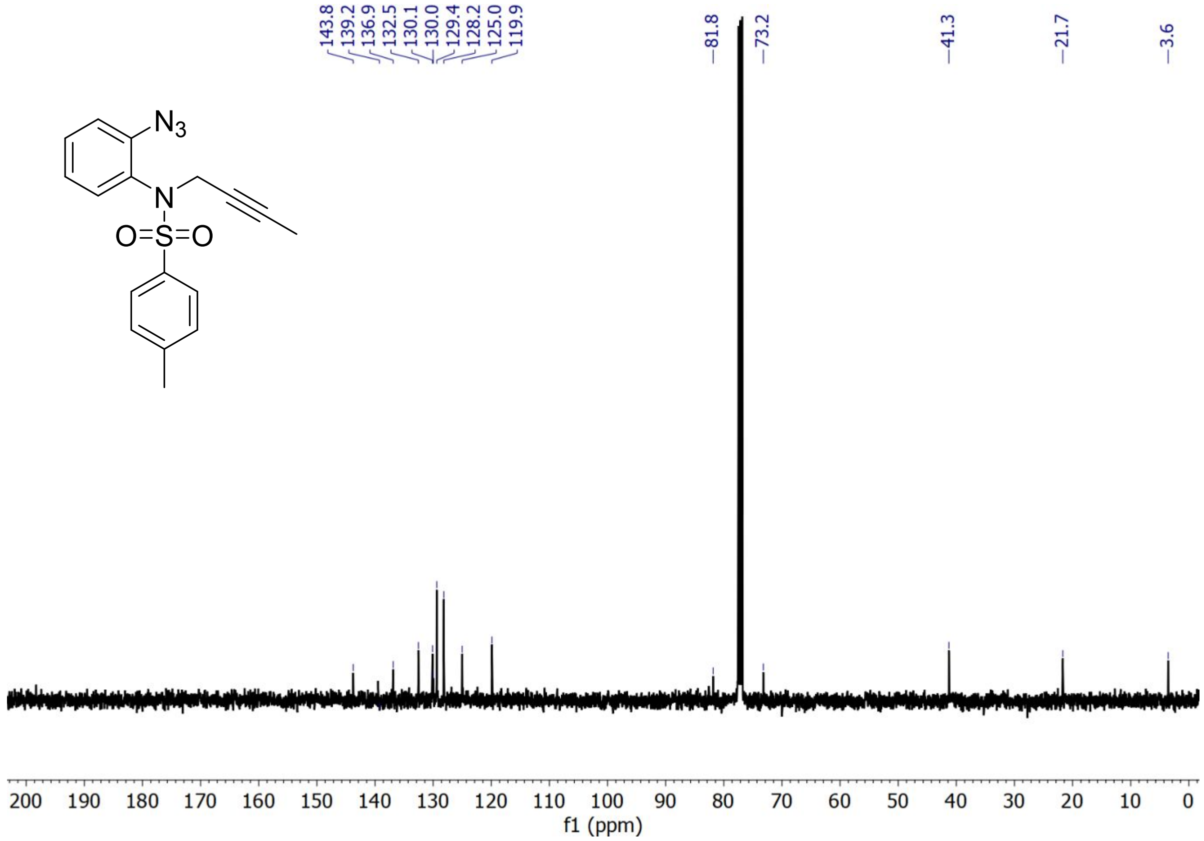

^{13}C NMR spectrum of **11l** (101 MHz, $CDCl_3$)

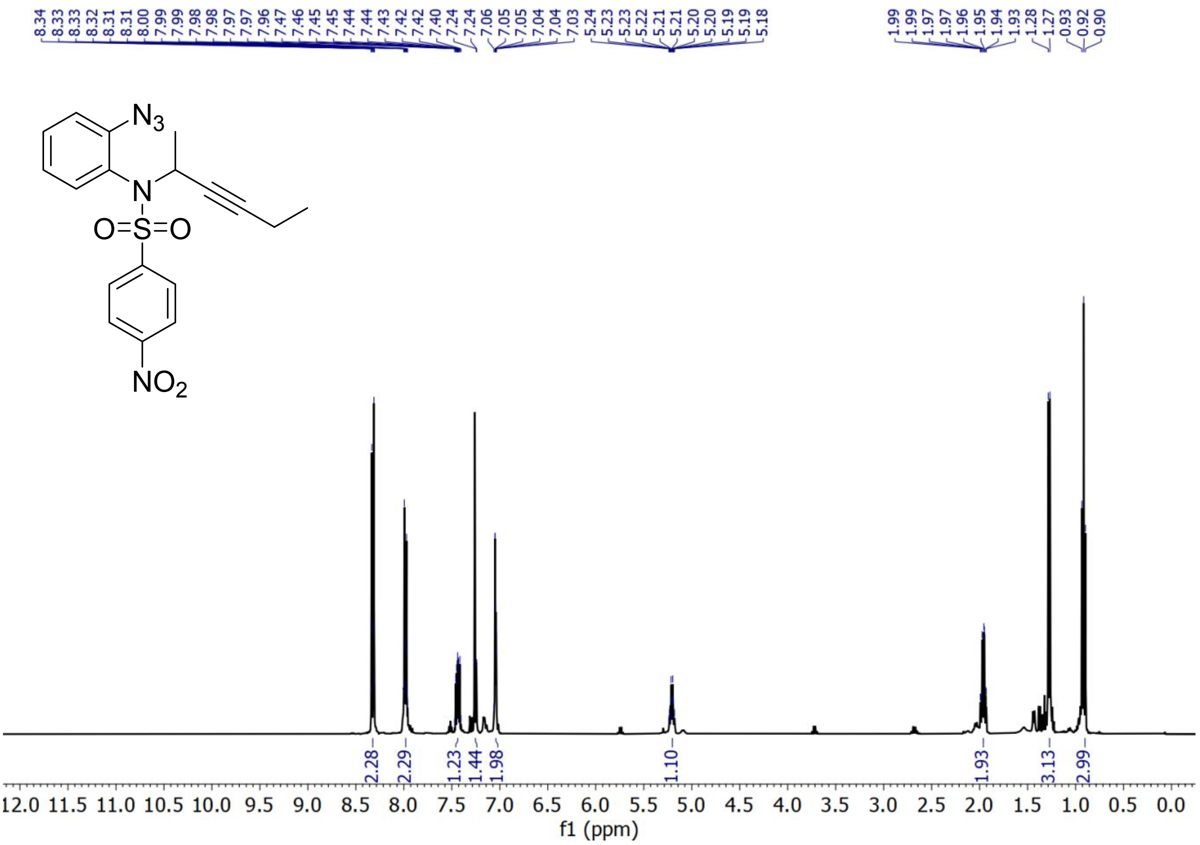

^{1}H NMR spectrum of **11m** (400 MHz, $CDCl_3$) – interfering signals belong to the cyclic product 12m

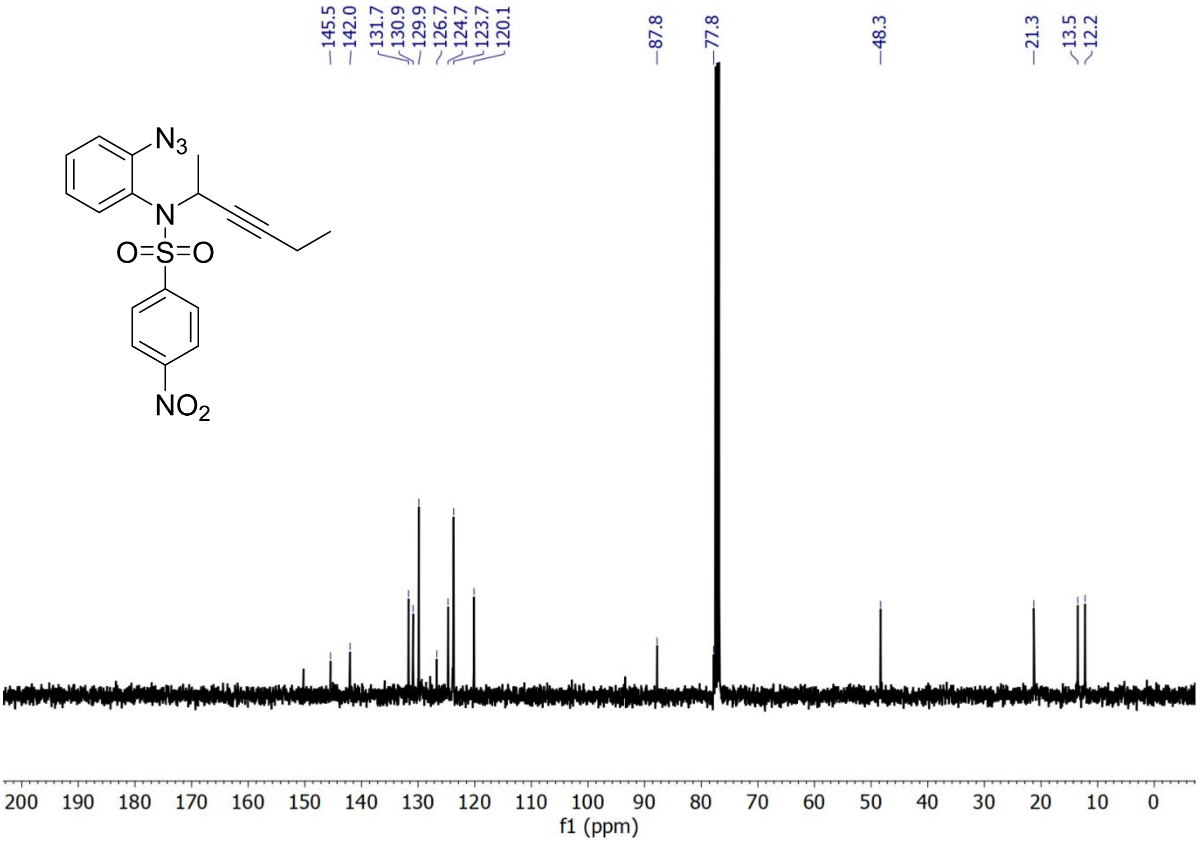

^{13}C NMR spectrum of **11m** (101 MHz, $CDCl_3$)

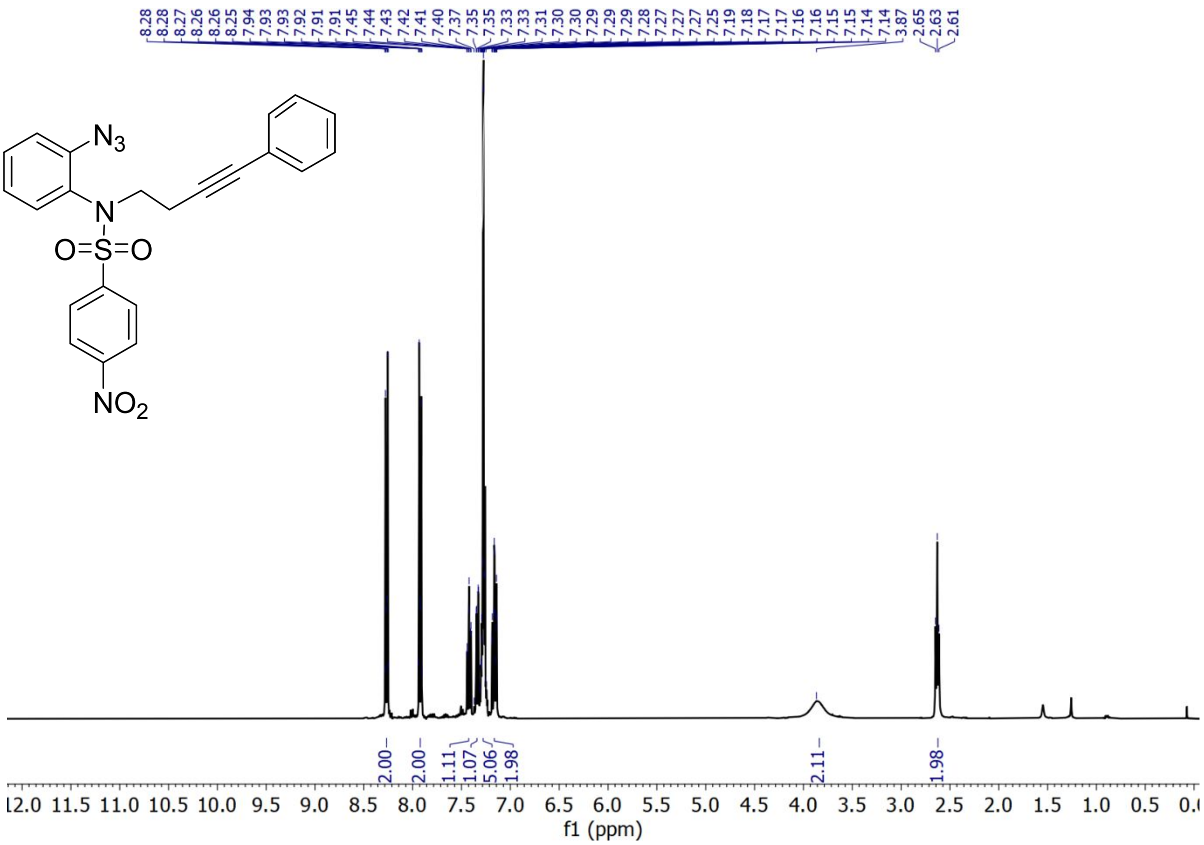

^{1}H NMR spectrum of **14a** (400 MHz, $CDCl_3$)

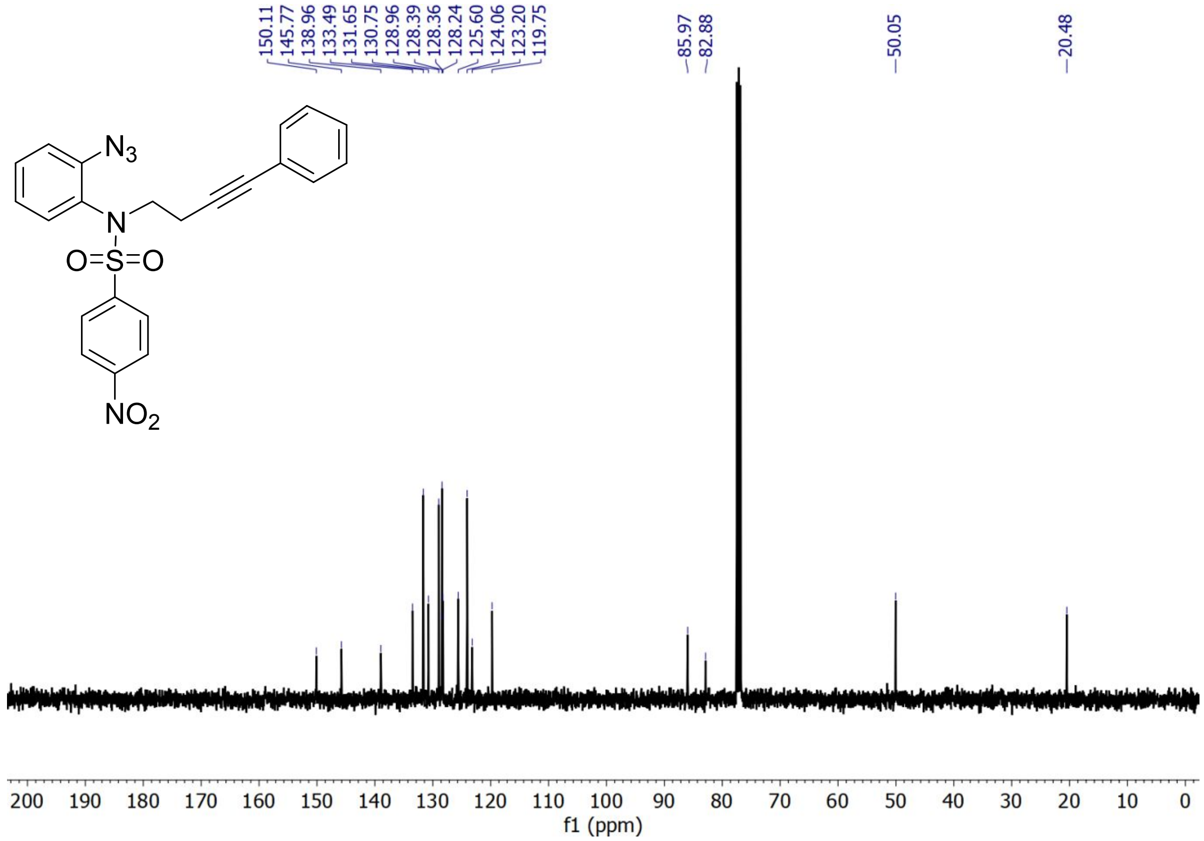

^{13}C NMR spectrum of **14a** (101 MHz, $CDCl_3$)

3. Copies of NMR spectra of products 12a-n and 15a-b

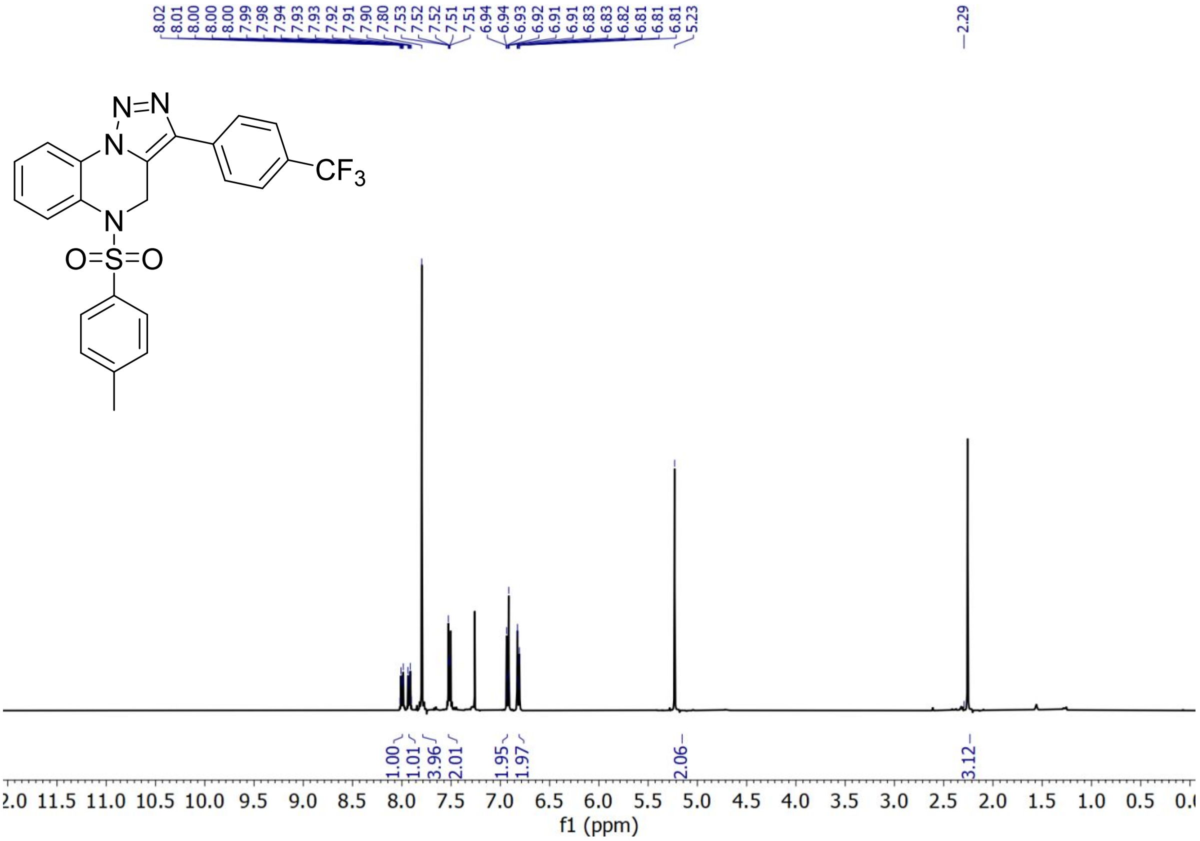

^{1}H NMR spectrum of **12a** (400 MHz, DMSO-d_6)

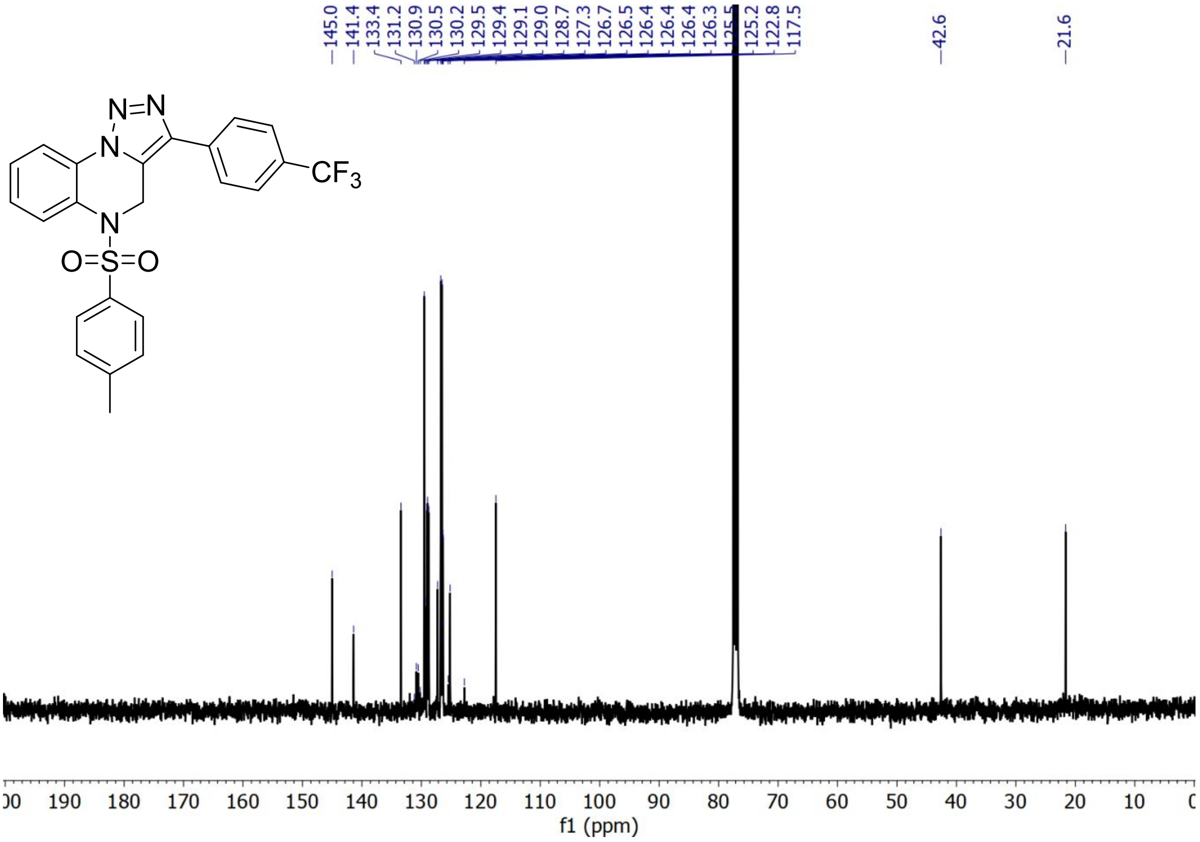

^{13}C NMR spectrum of **12a** (101 MHz, DMSO-d_6)

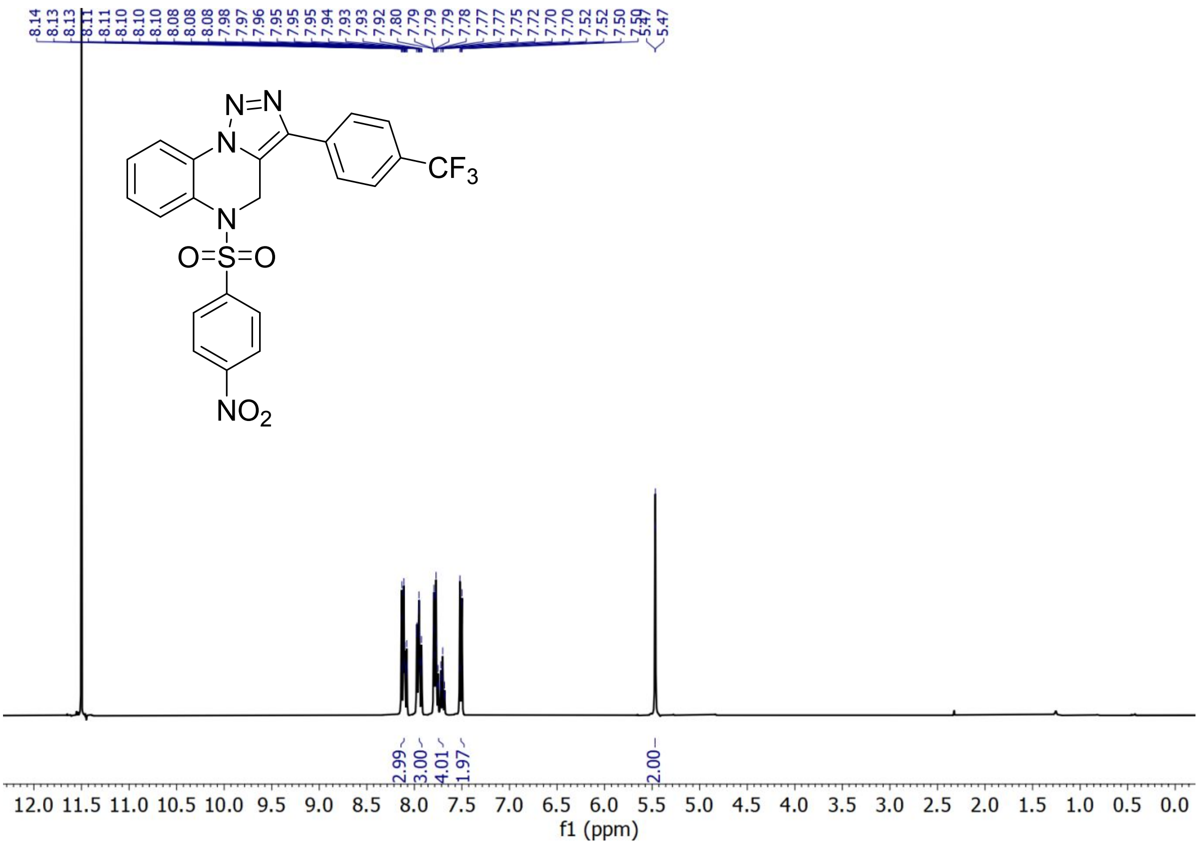

^{1}H NMR spectrum of **12b** (400 MHz, TFA-*d*)

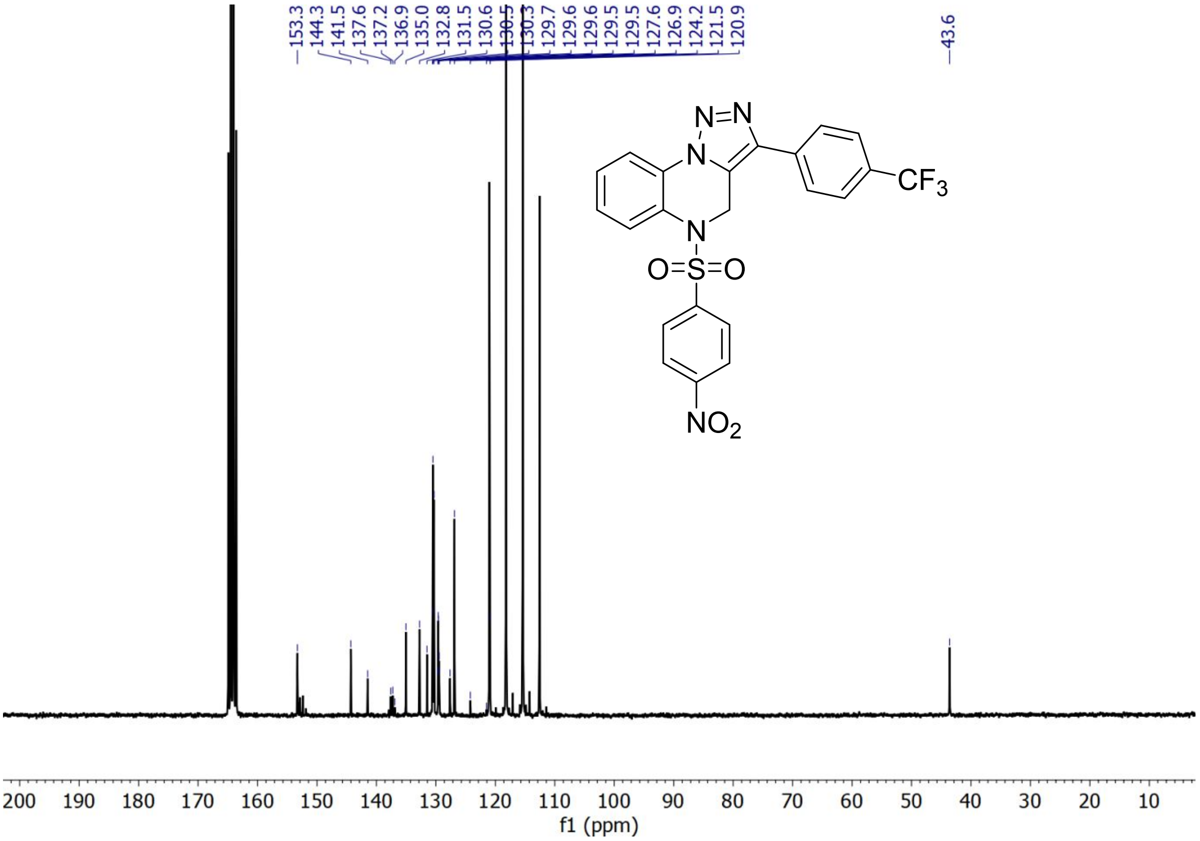

^{13}C NMR spectrum of **12b** (101 MHz, TFA-*d*) – impurities from TFA-*d*: δ 152.8 (q, *J* = 48.8 Hz), 116 (q, *J* = 284.5 Hz)).

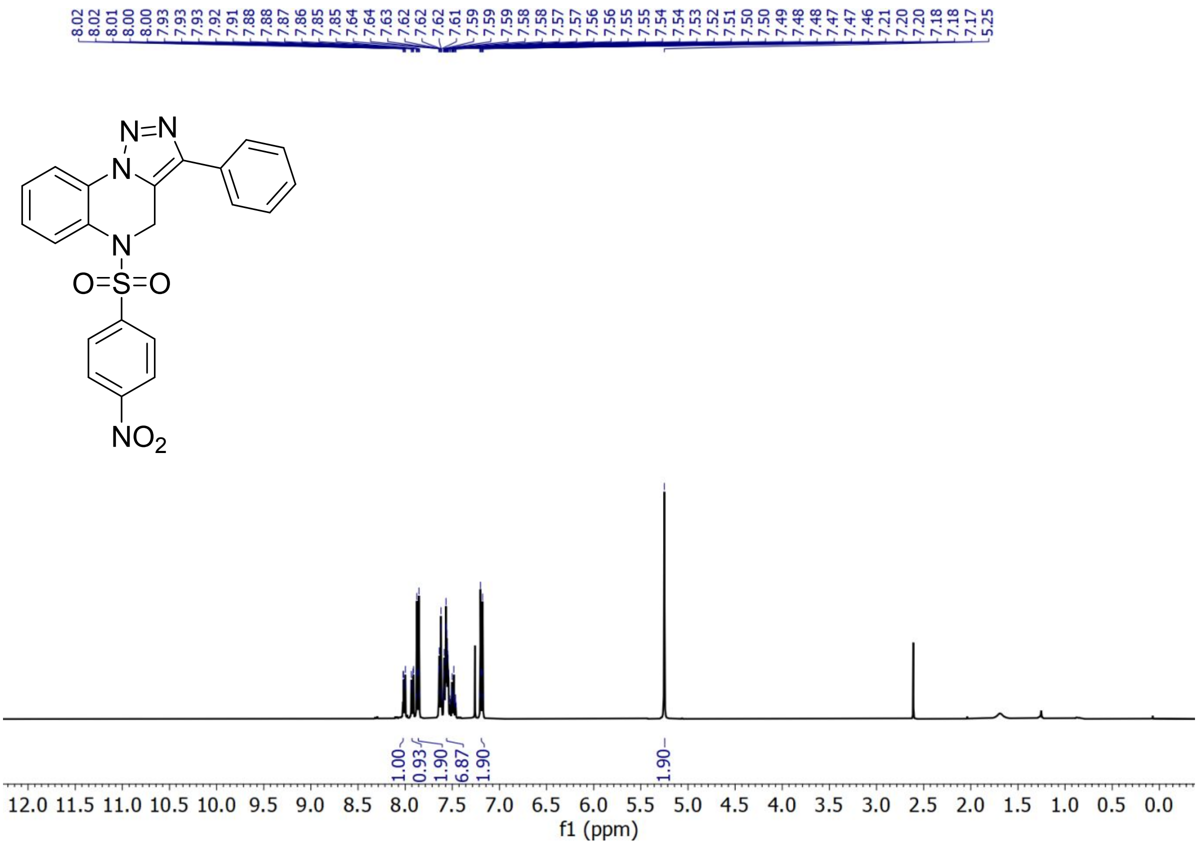

^{1}H NMR spectrum of **12c** (400 MHz, $CDCl_3$)

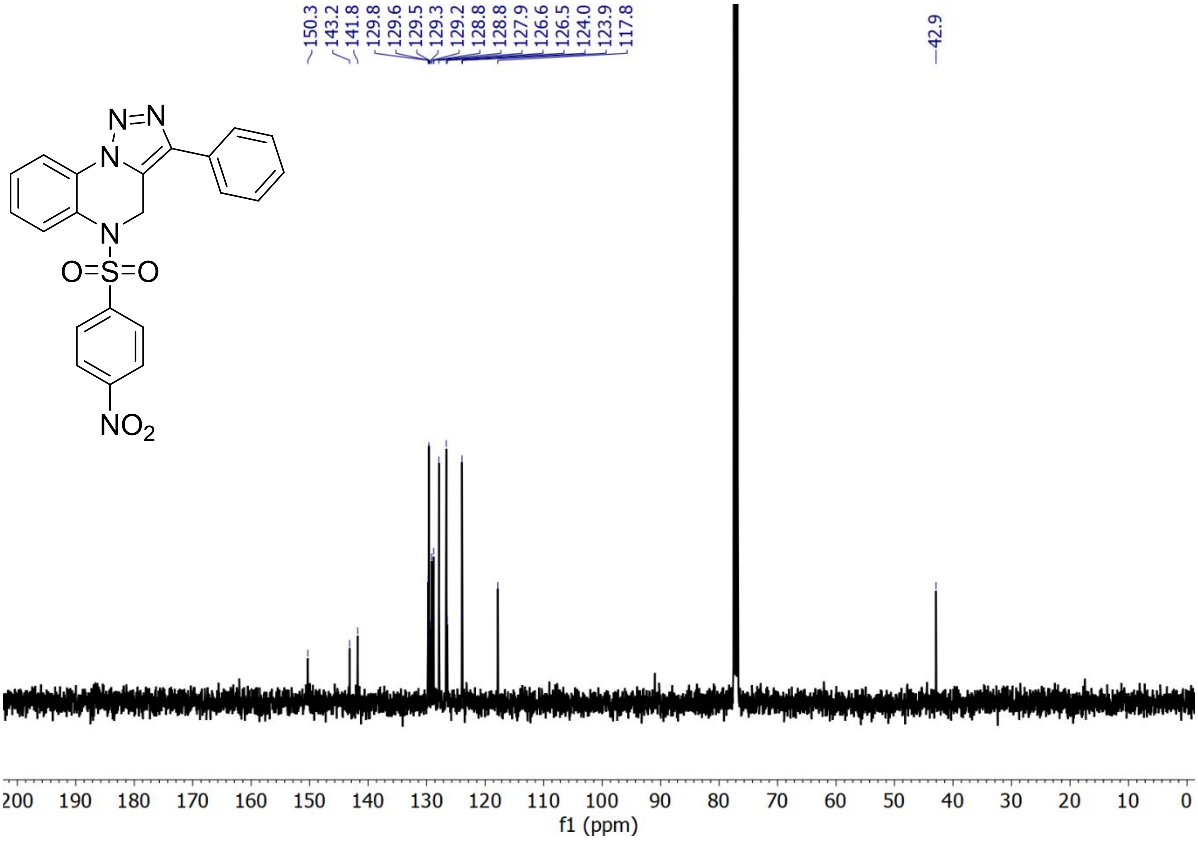

^{13}C NMR spectrum of **12c** (101 MHz, $CDCl_3$)

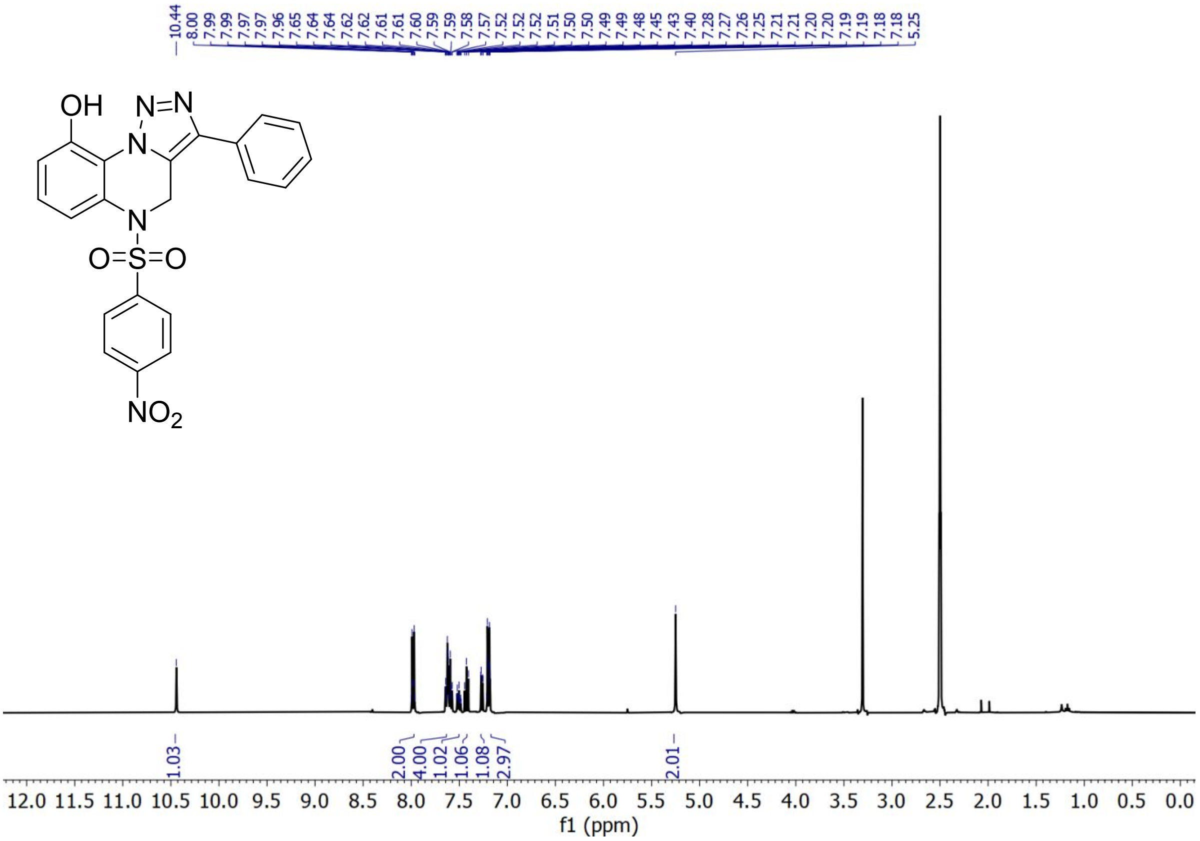

^{1}H NMR spectrum of **12e** (400 MHz, DMSO-d_6)

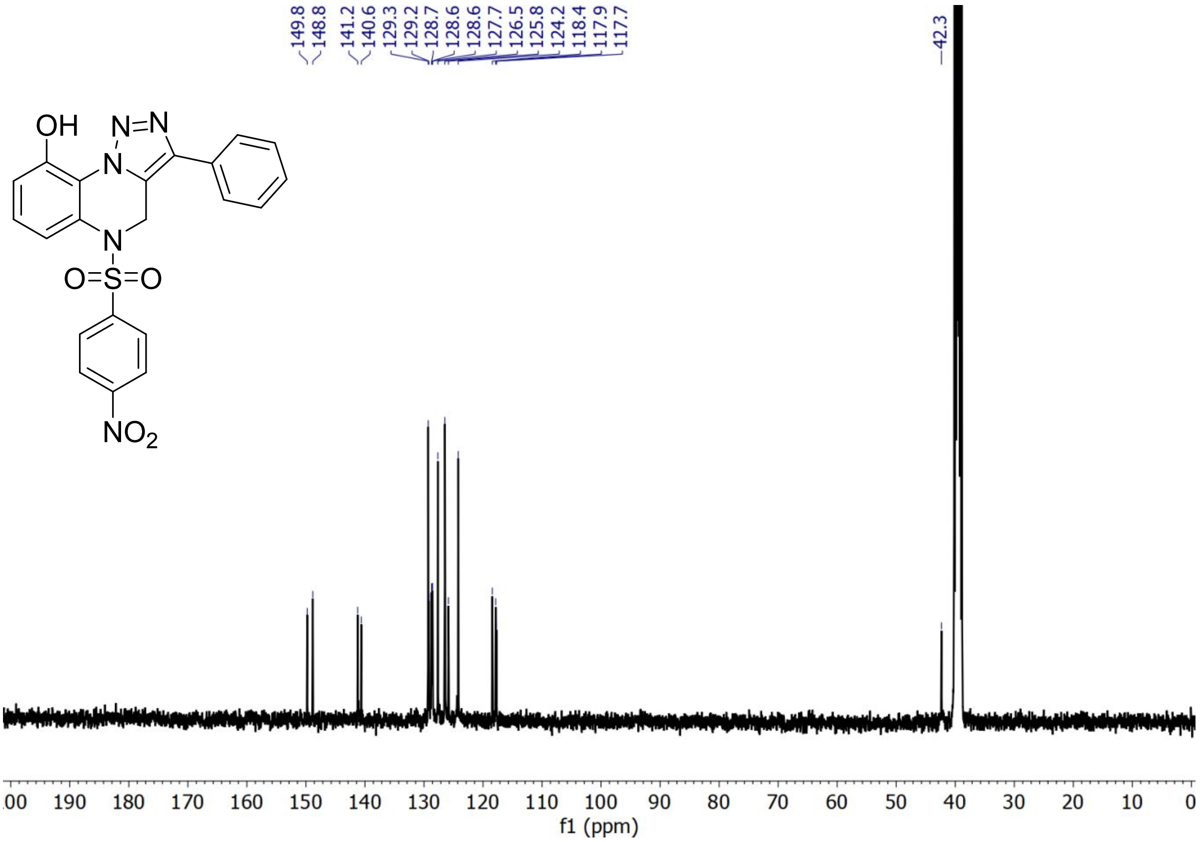

^{13}C NMR spectrum of **12e** (101 MHz, DMSO-d_6)

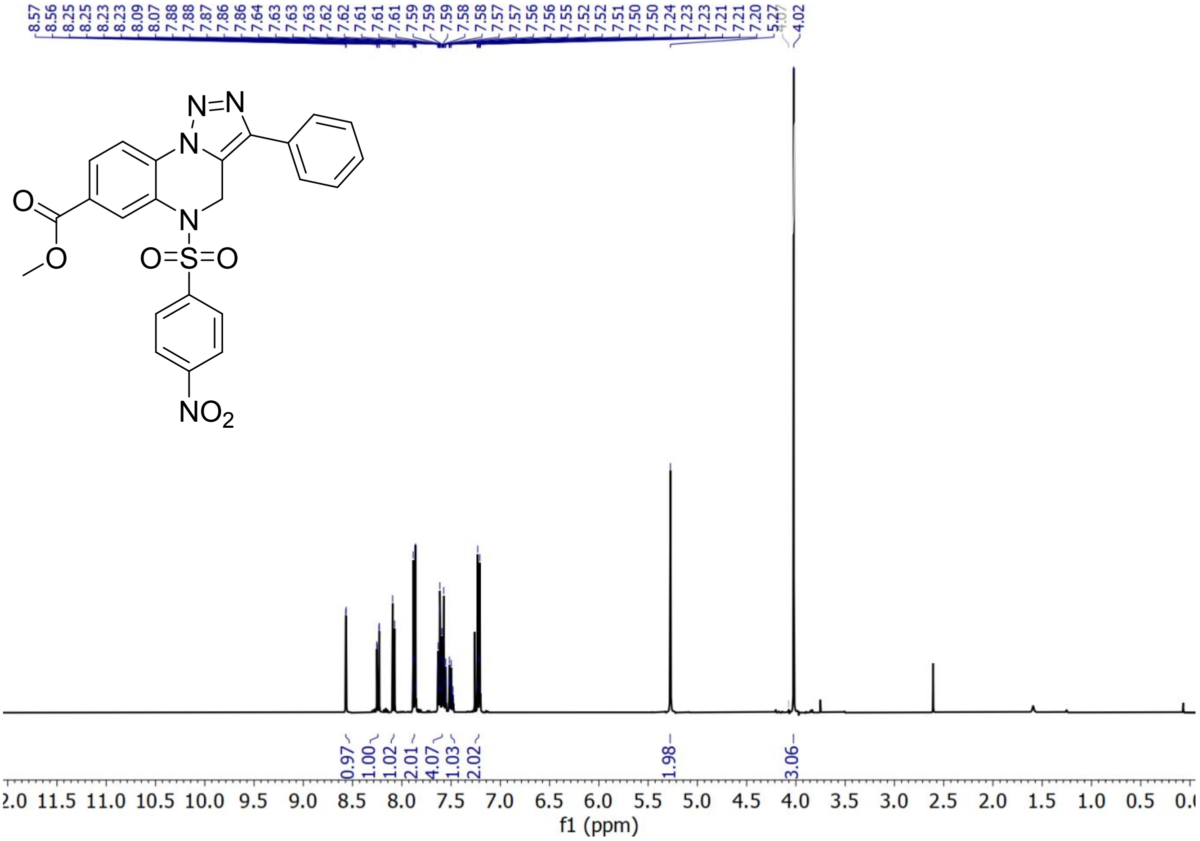

^{1}H NMR spectrum of **12f** (400 MHz, $CDCl_3$)

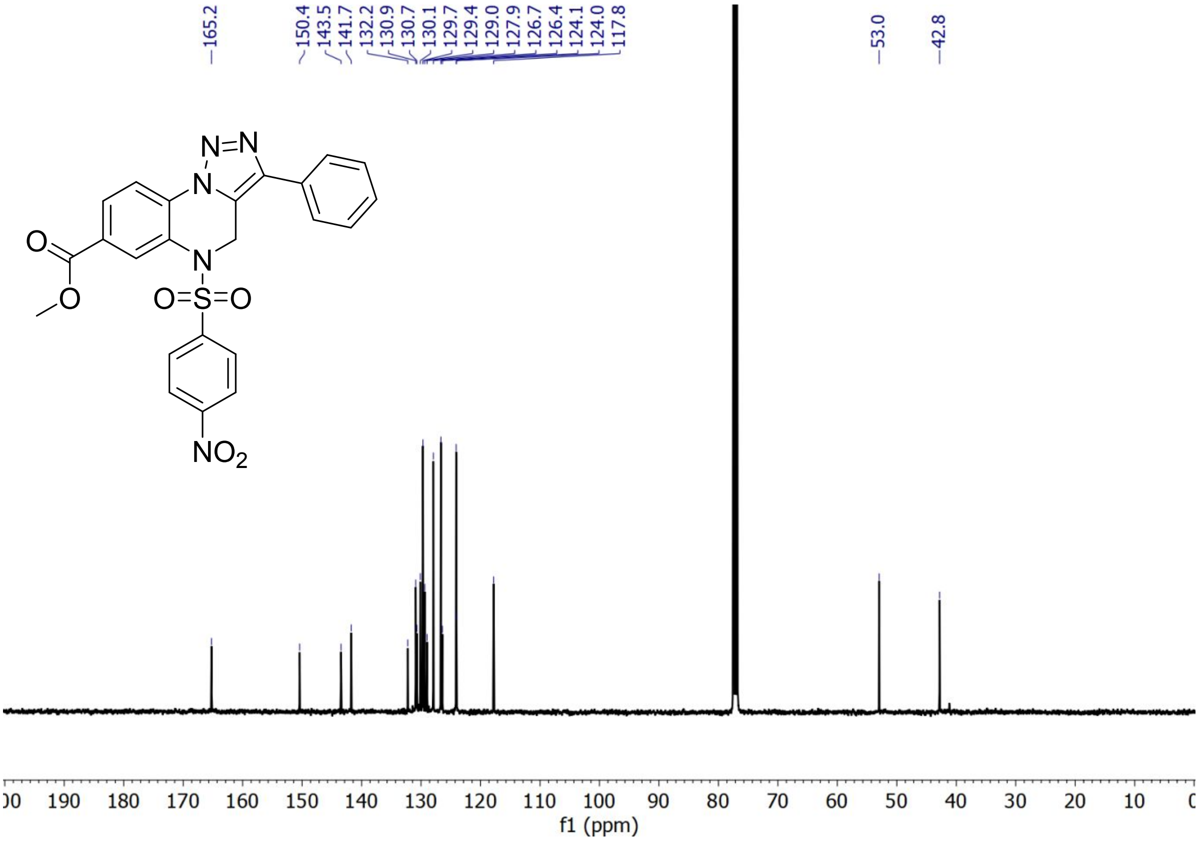

^{13}C NMR spectrum of **12f** (101 MHz, $CDCl_3$)

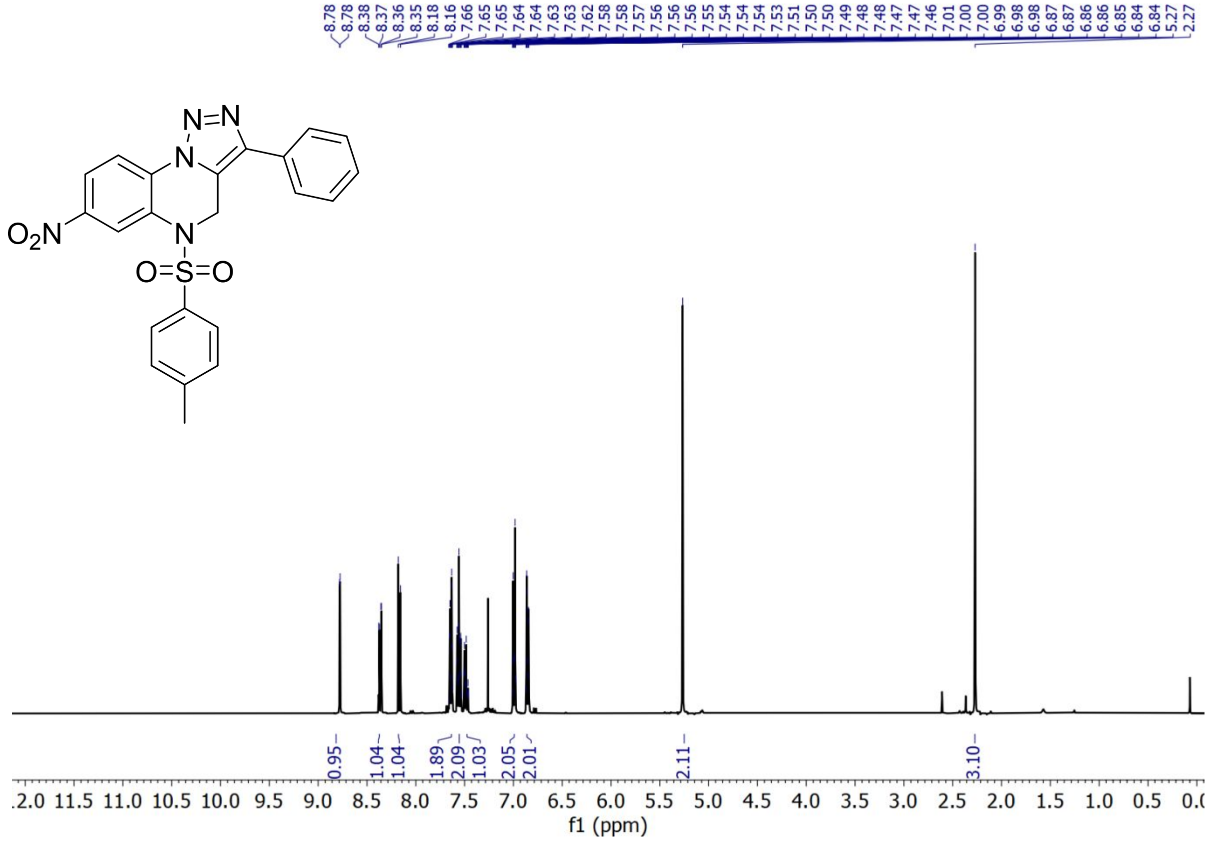

^{1}H NMR spectrum of **12g** (400 MHz, $CDCl_3$)

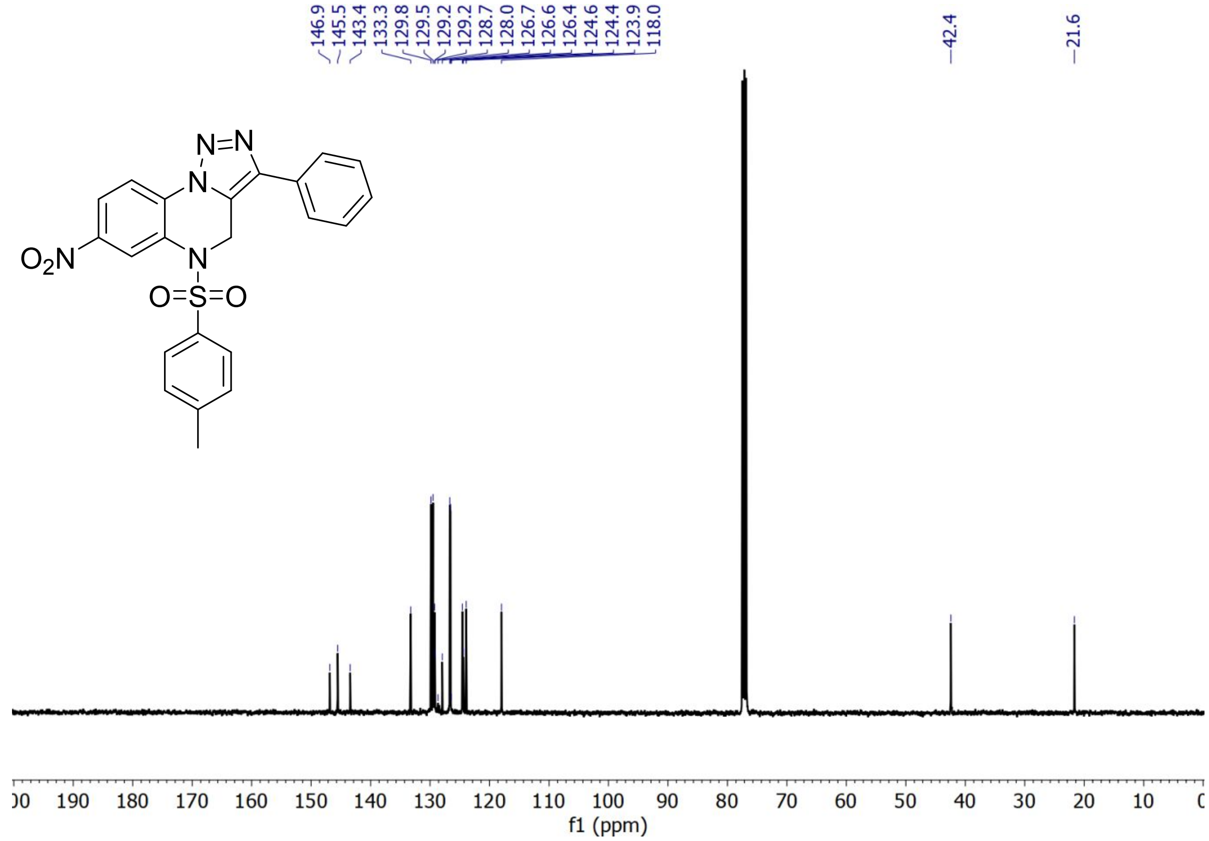

^{13}C NMR spectrum of **12g** (101 MHz, $CDCl_3$)

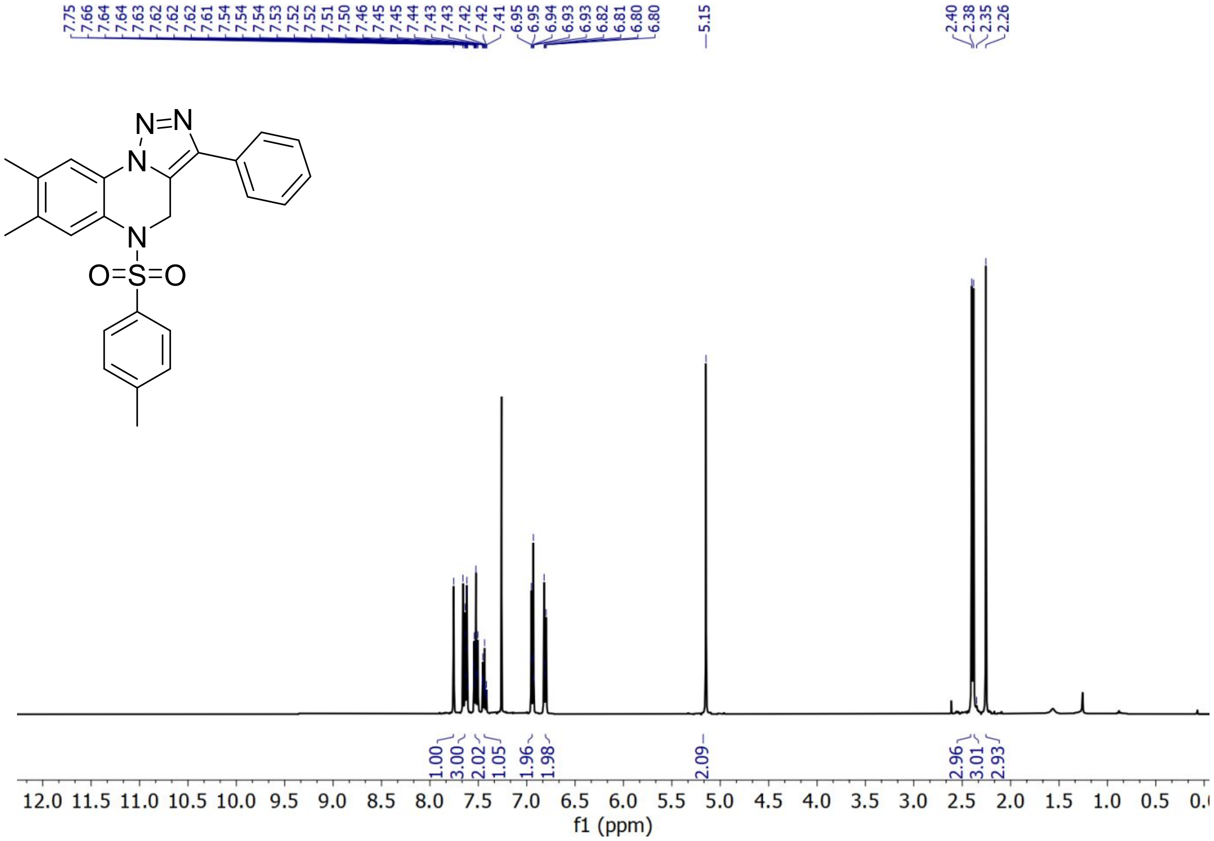

^{1}H NMR spectrum of **12h** (400 MHz, $CDCl_3$)

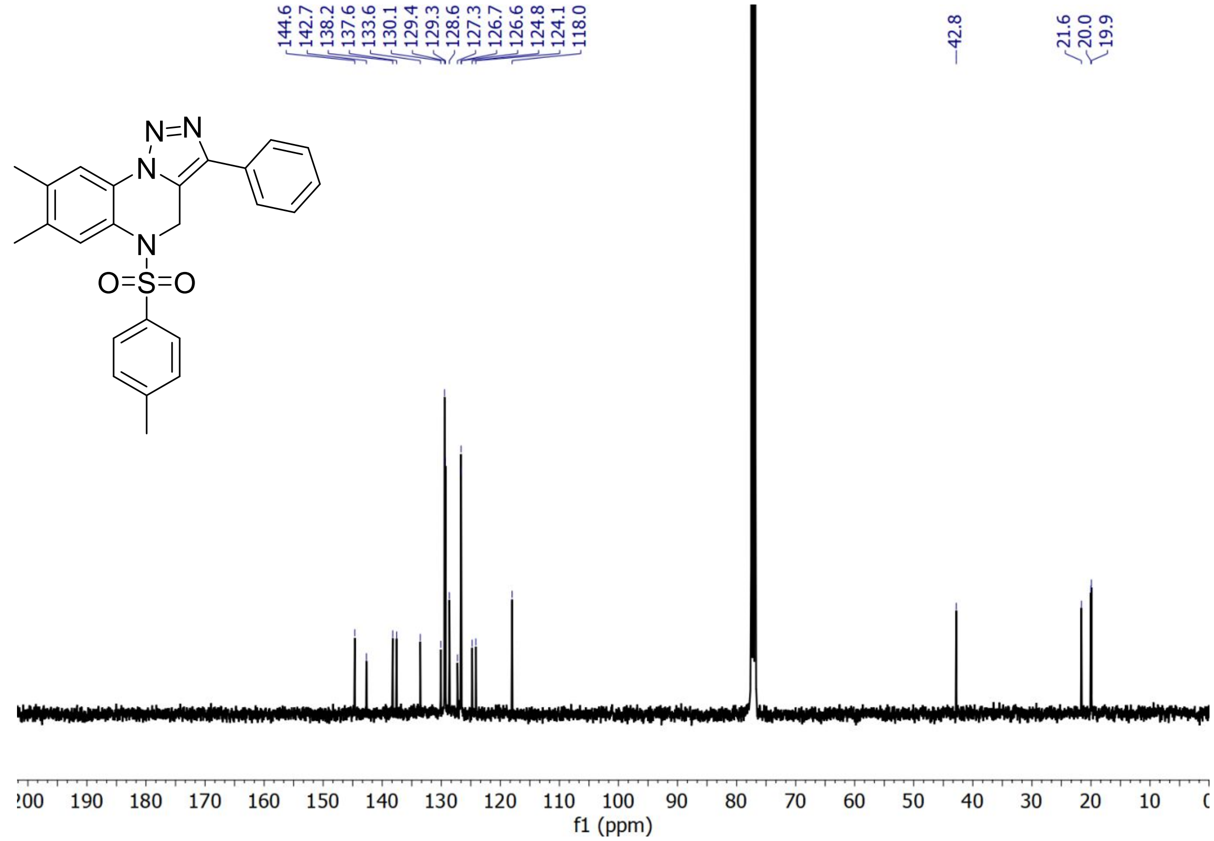

^{13}C NMR spectrum of **12h** (101 MHz, $CDCl_3$)

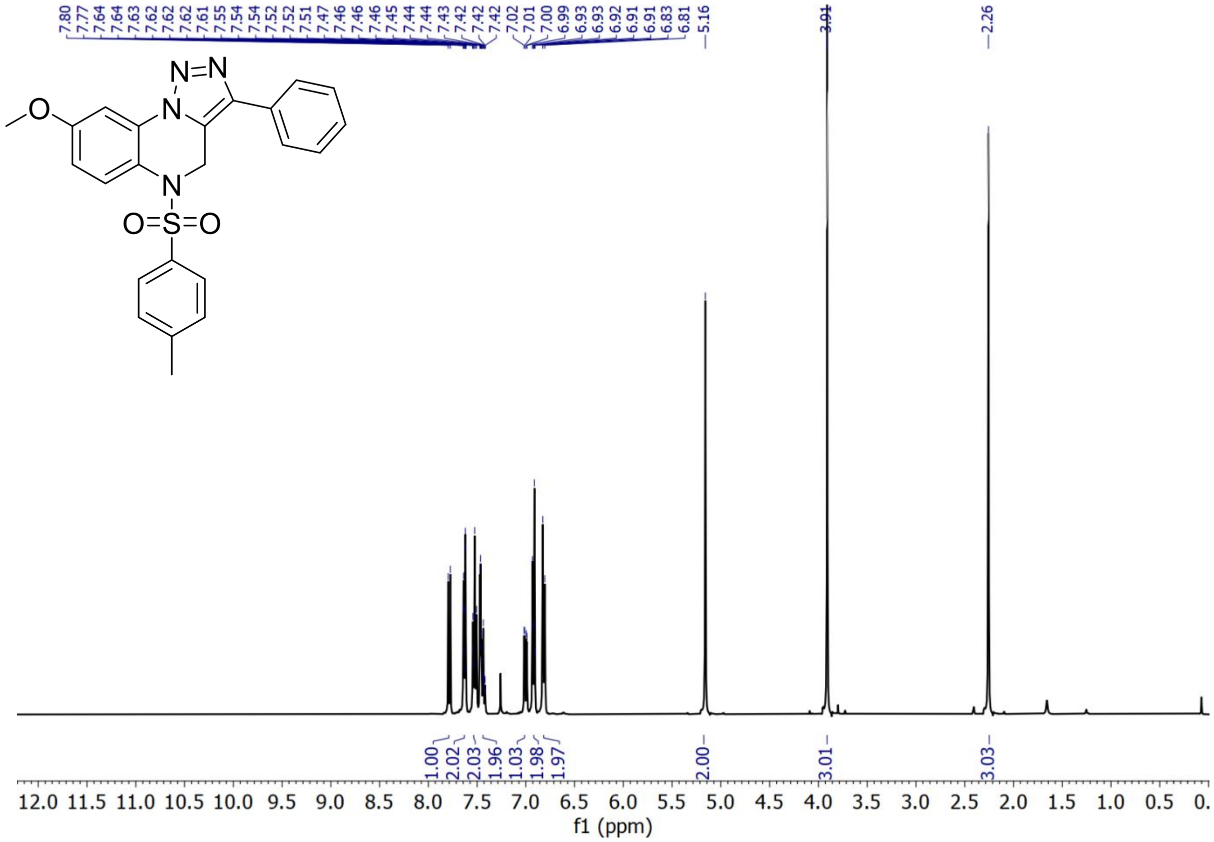

^{1}H NMR spectrum of **12i** (400 MHz, $CDCl_3$)

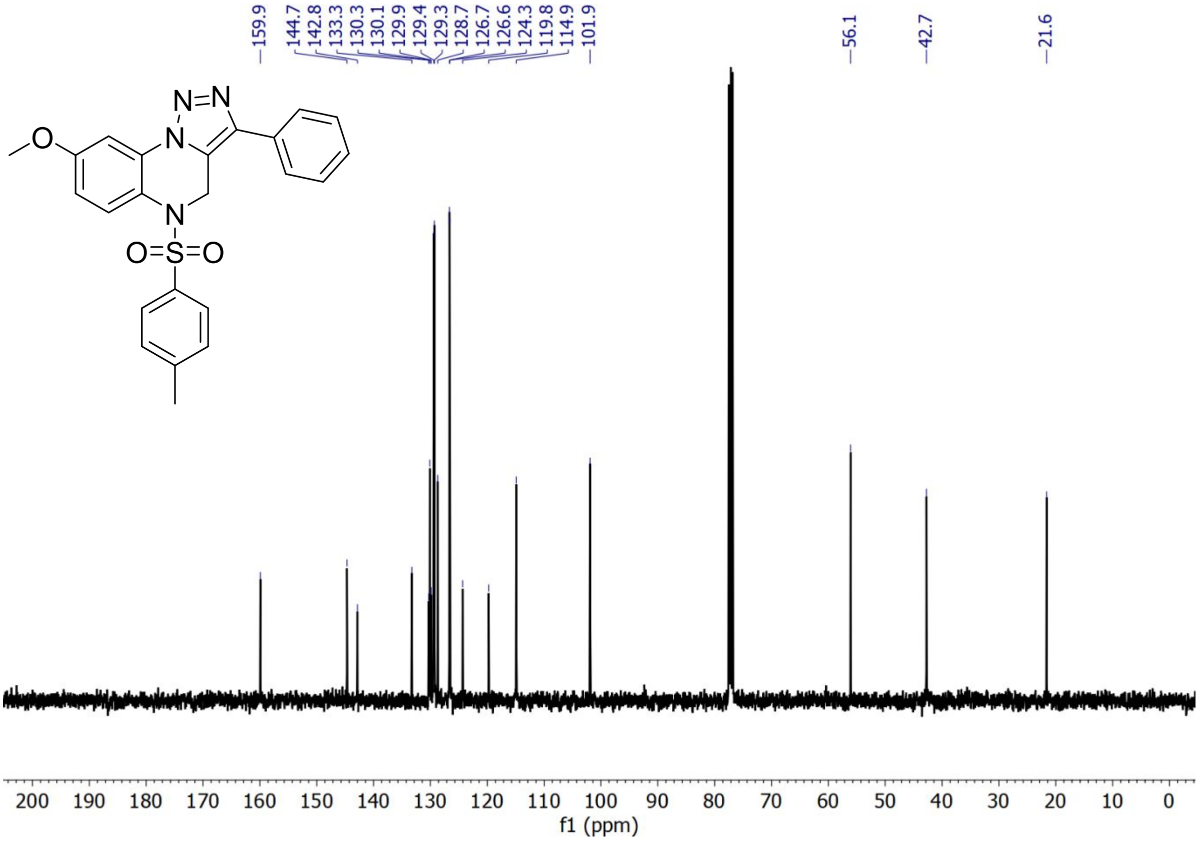

^{13}C NMR spectrum of **12i** (101 MHz, $CDCl_3$)

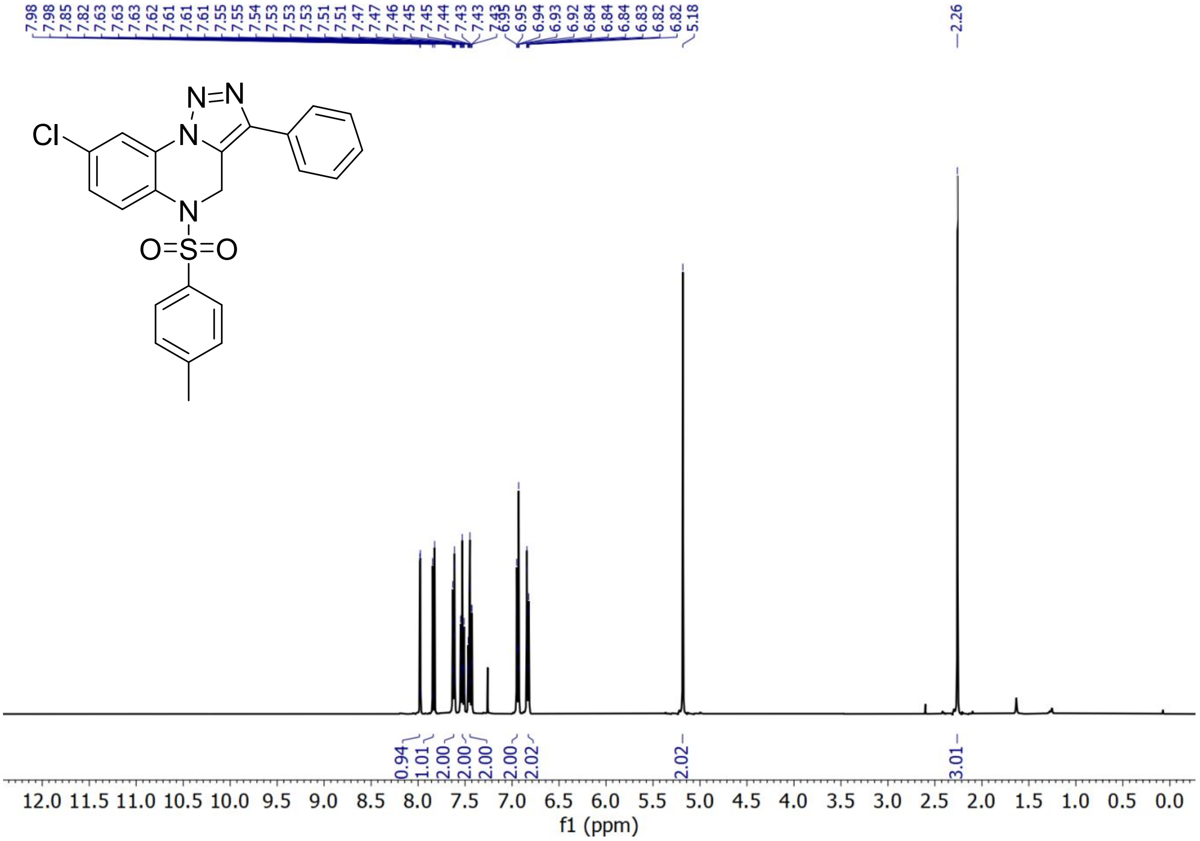

^{1}H NMR spectrum of **12j** (400 MHz, $CDCl_3$)

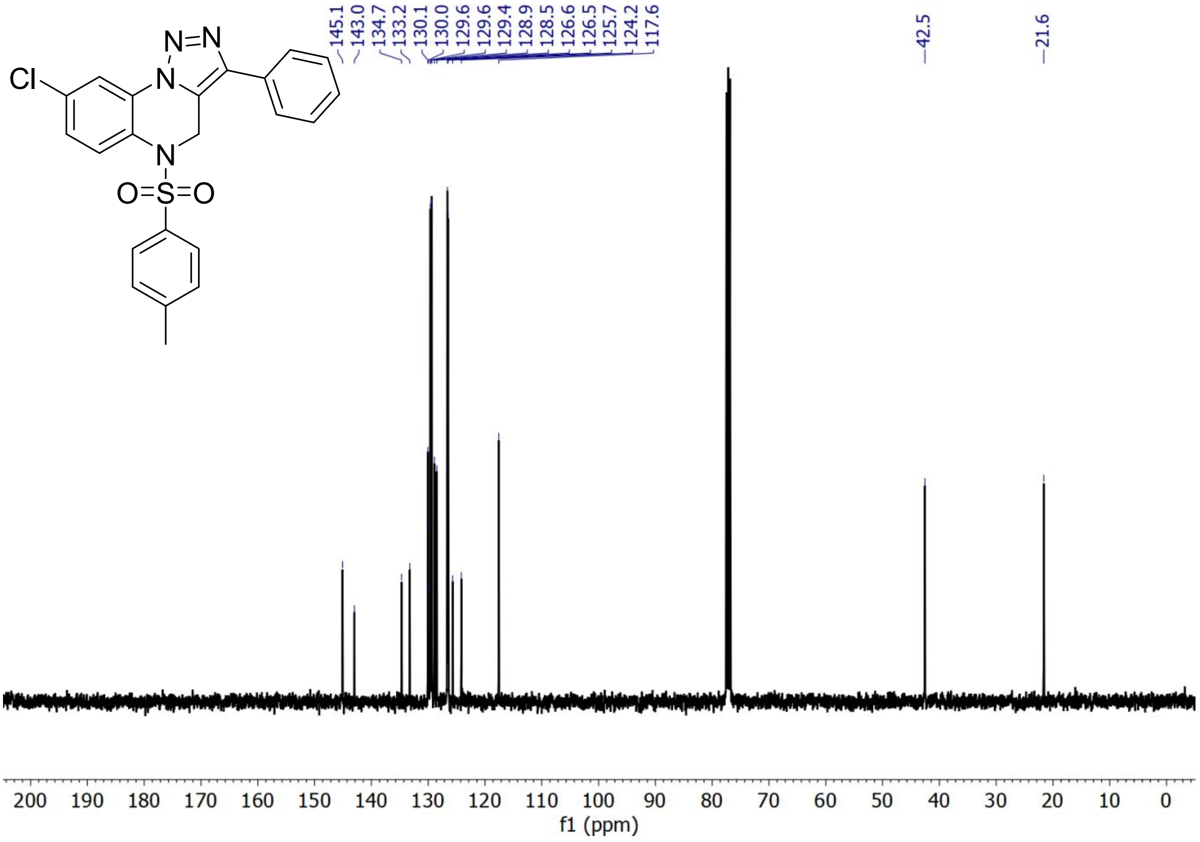

^{13}C NMR spectrum of **12j** (101 MHz, $CDCl_3$)

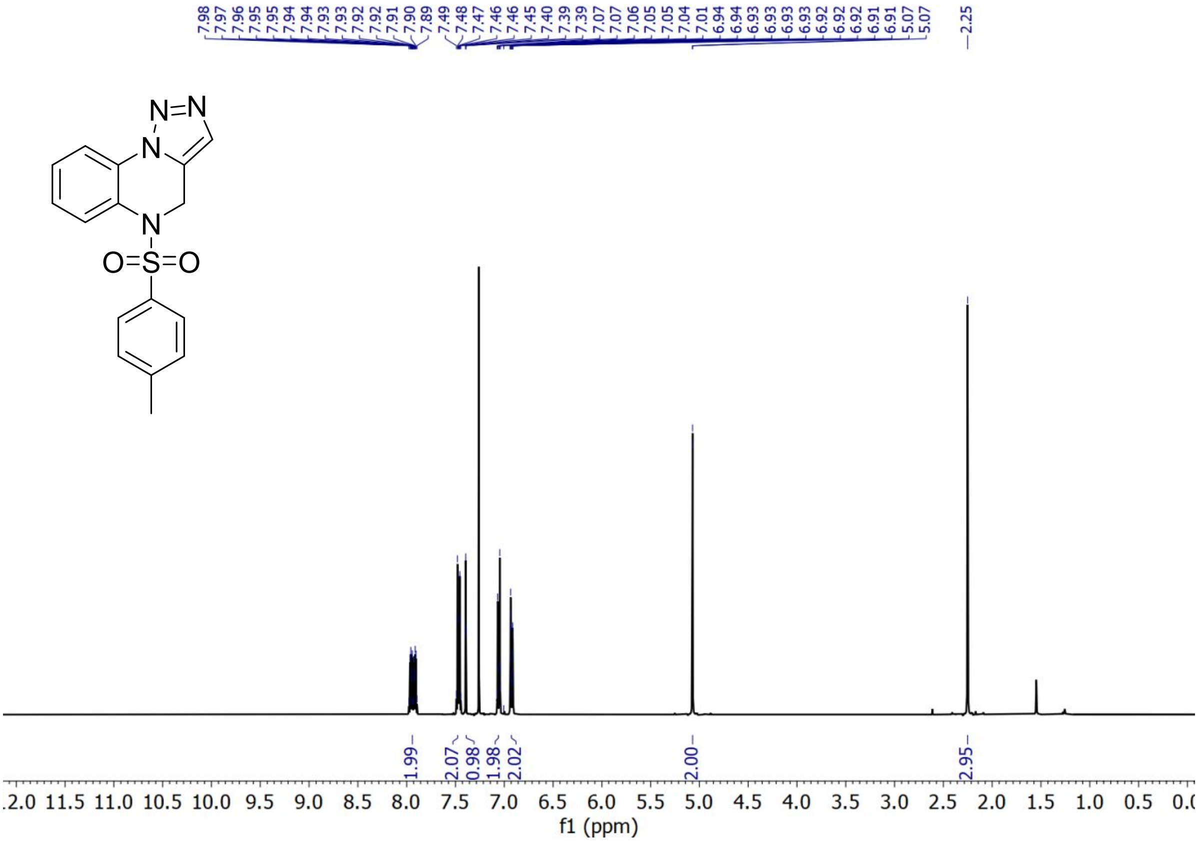

^1H NMR spectrum of **12k** (400 MHz, $CDCl_3$)

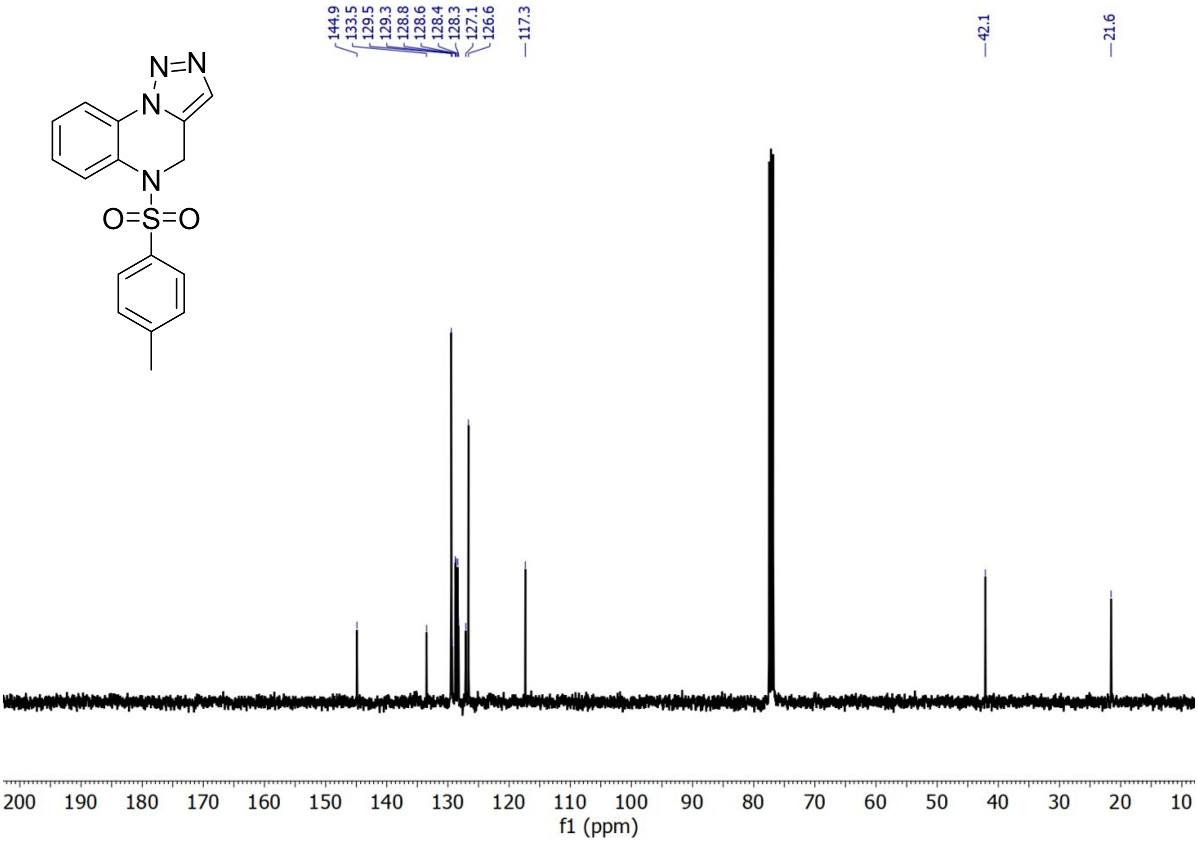

^{13}C NMR spectrum of **12k** (101 MHz, $CDCl_3$)

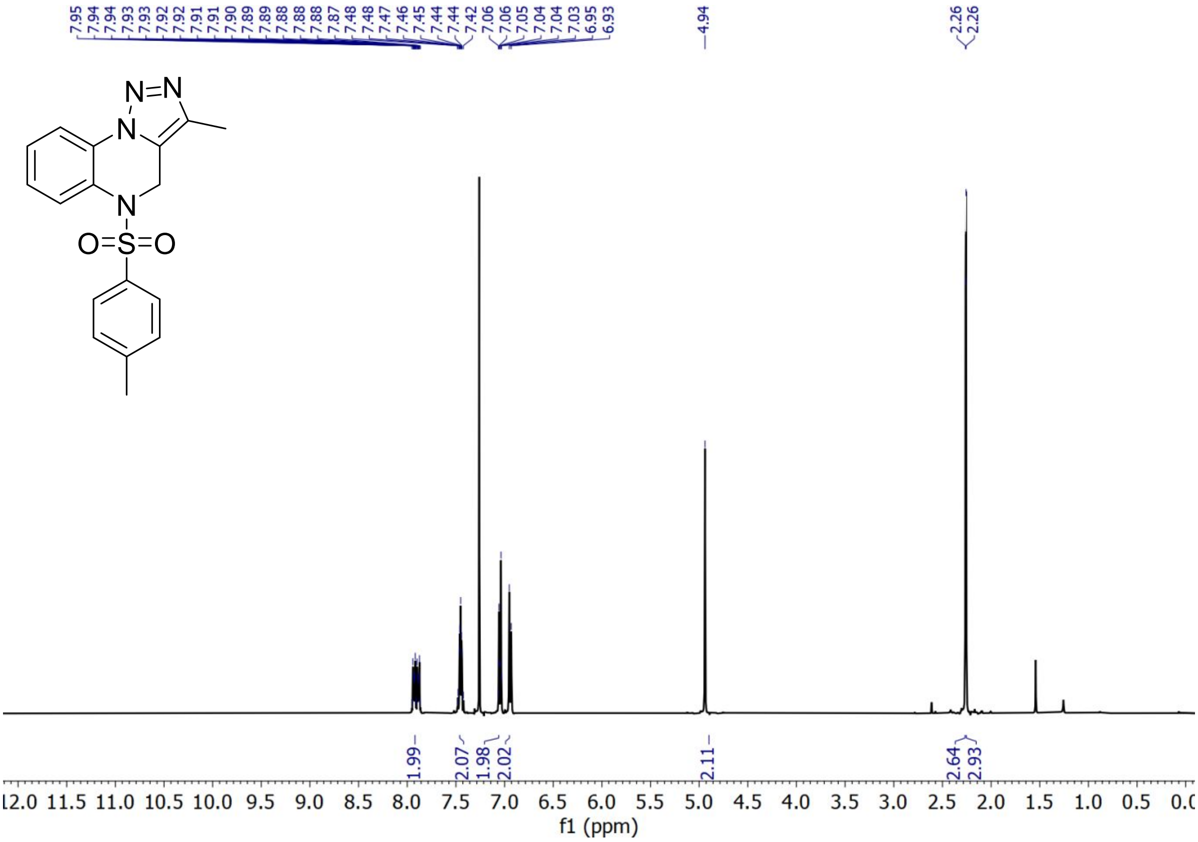

^{1}H NMR spectrum of **12l** (400 MHz, $CDCl_3$)

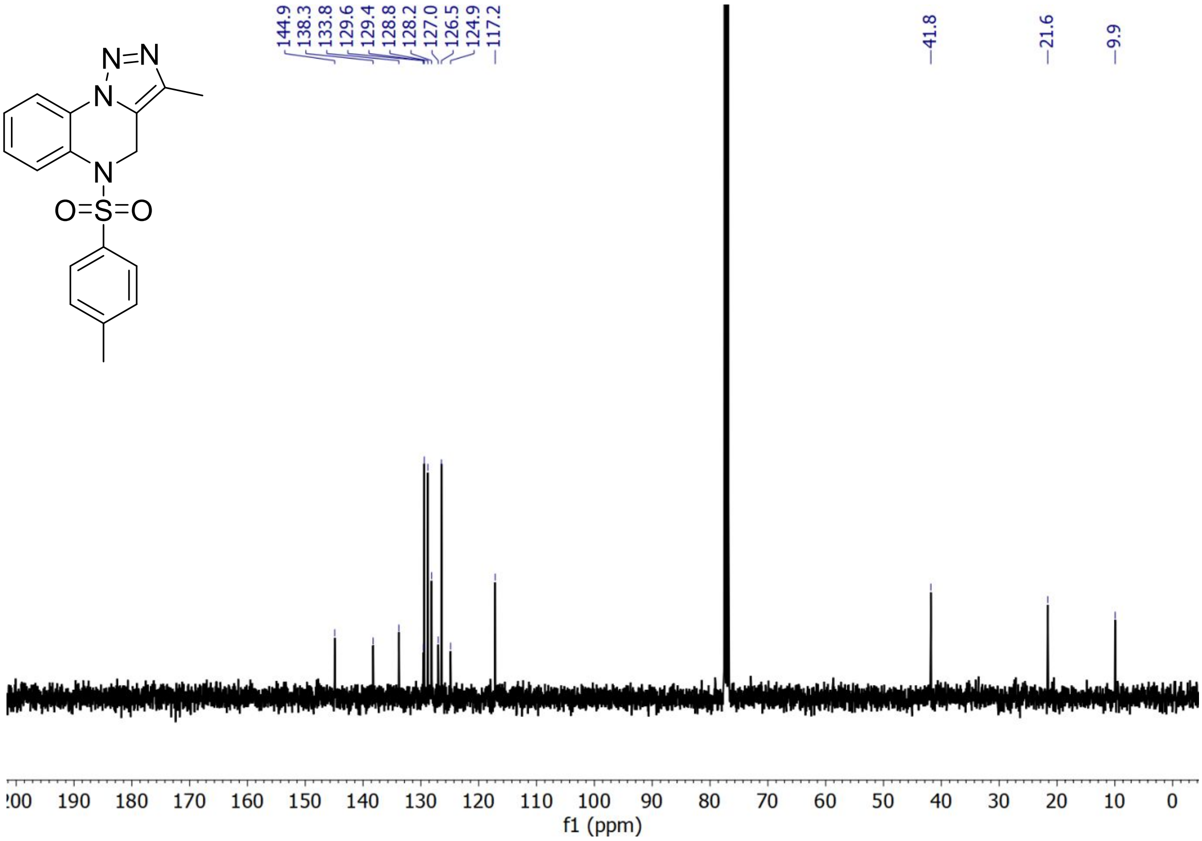

^{13}C NMR spectrum of **12l** (101 MHz, $CDCl_3$)

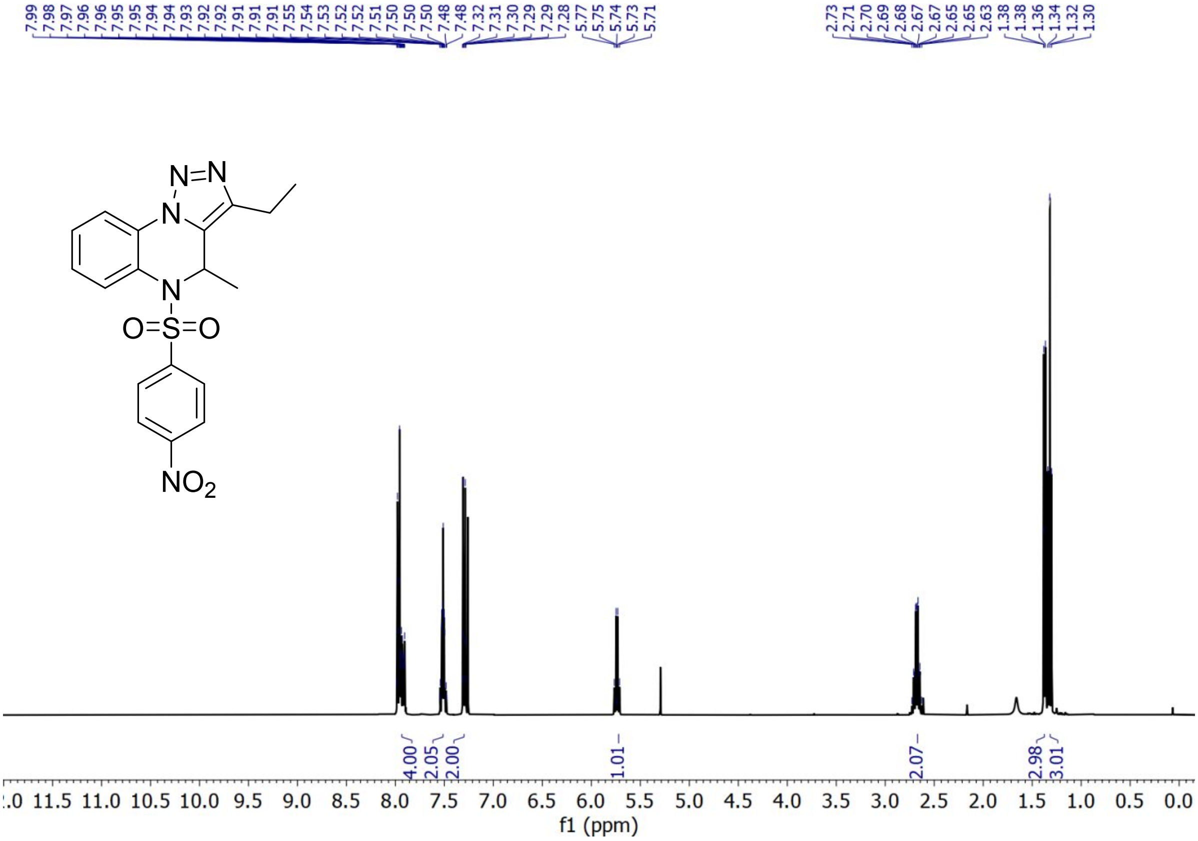

^{1}H NMR spectrum of **12m** (400 MHz, $CDCl_3$)

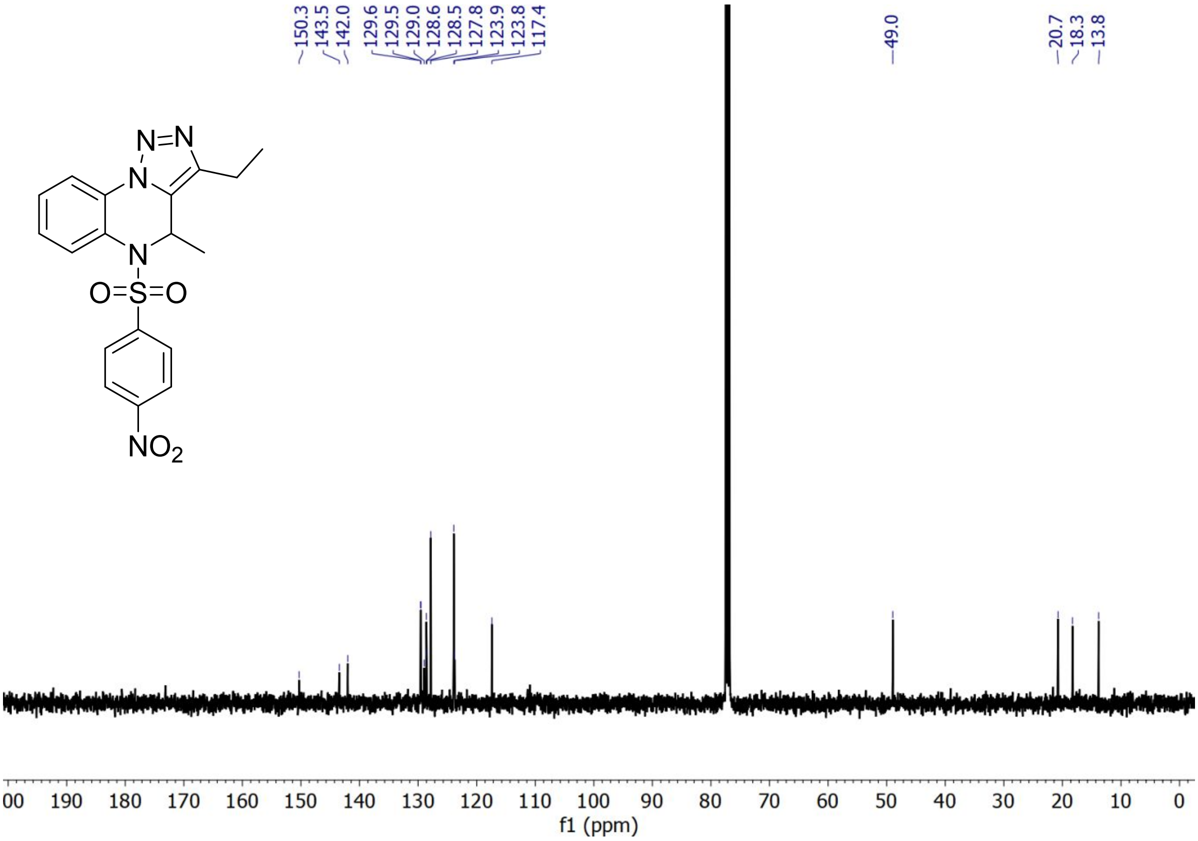

^{13}C NMR spectrum of **12m** (101 MHz, $CDCl_3$)

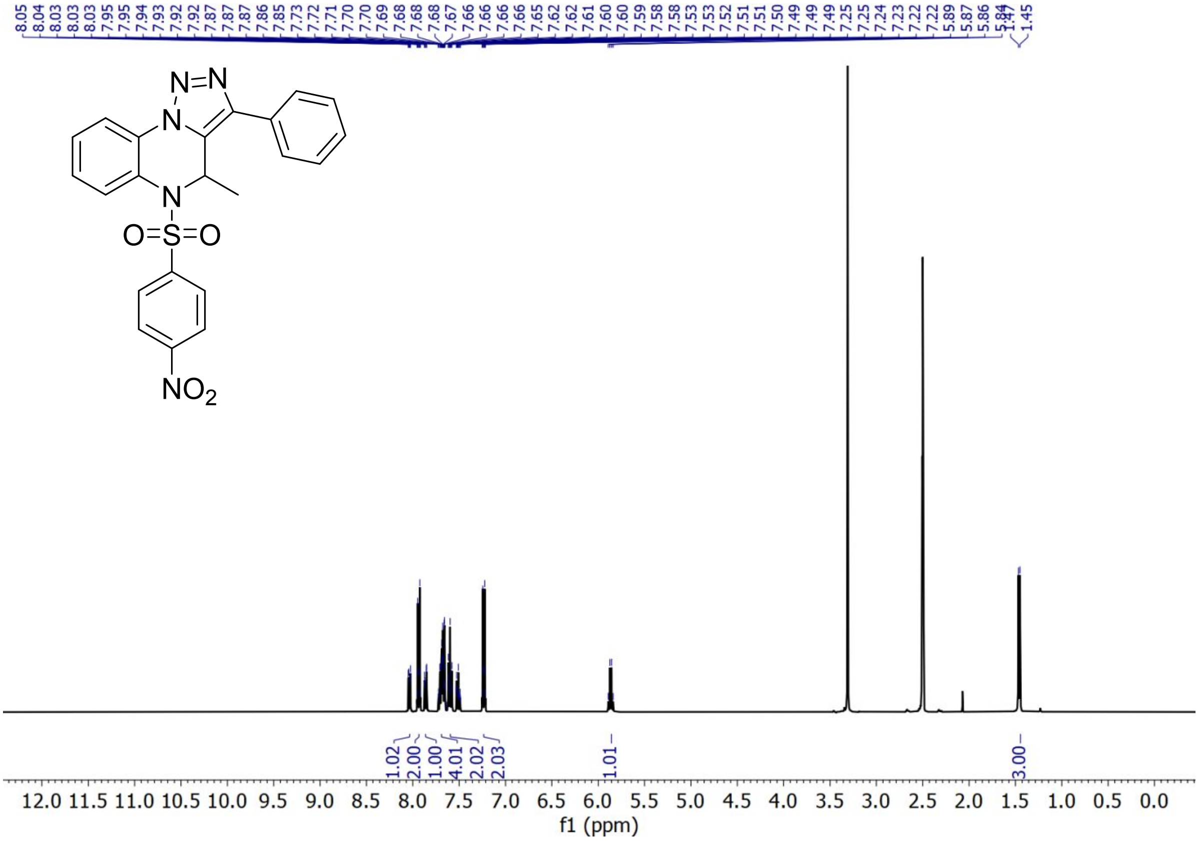

^{1}H NMR spectrum of **12n** (400 MHz, DMSO-d_6)

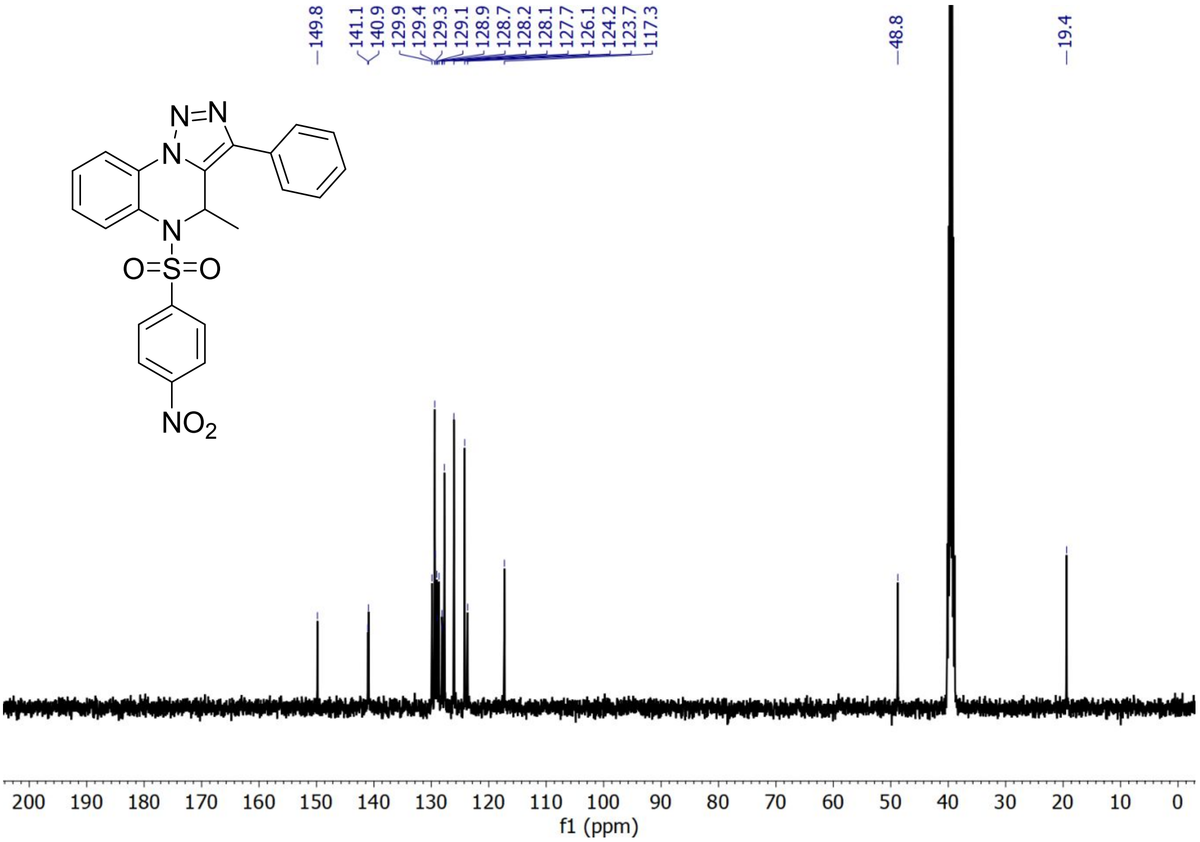

^{13}C NMR spectrum of **12n** (101 MHz, DMSO-d_6)

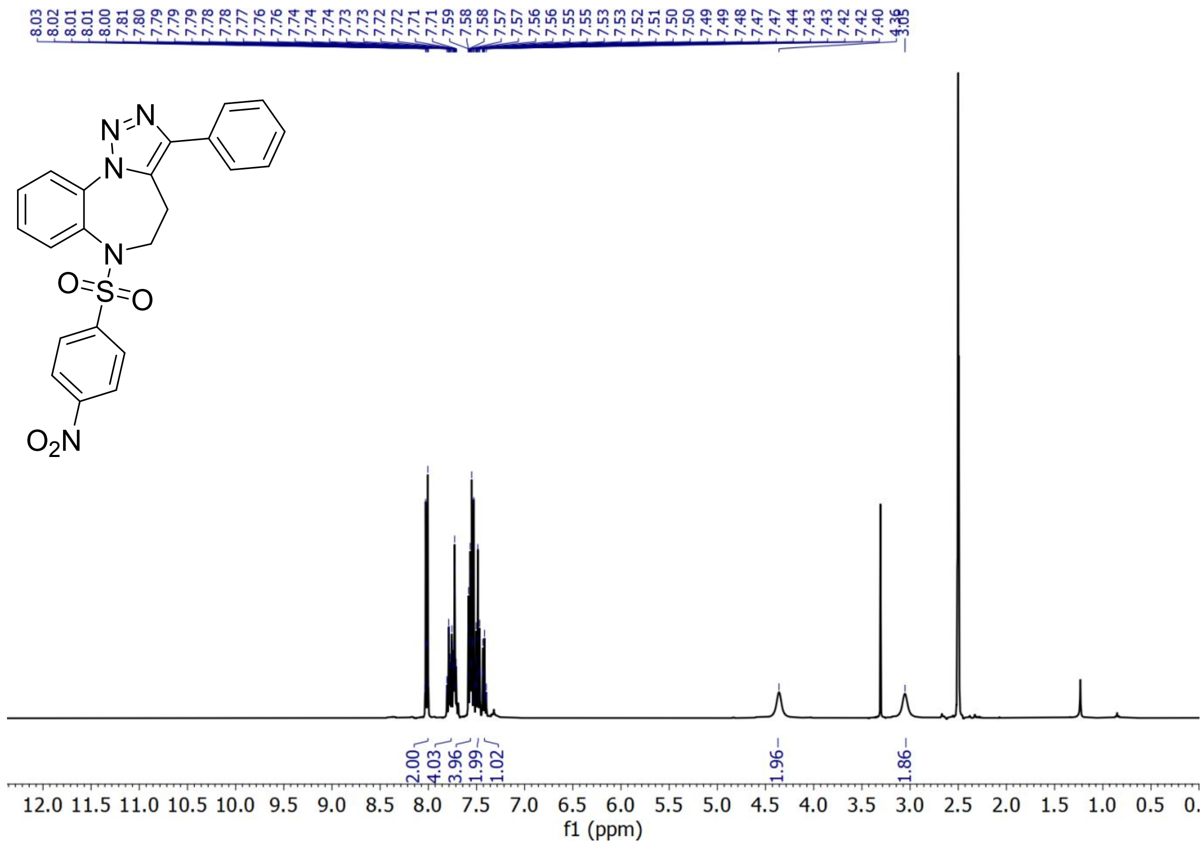

^{1}H NMR spectrum of **15a** (400 MHz, DMSO-d_6)

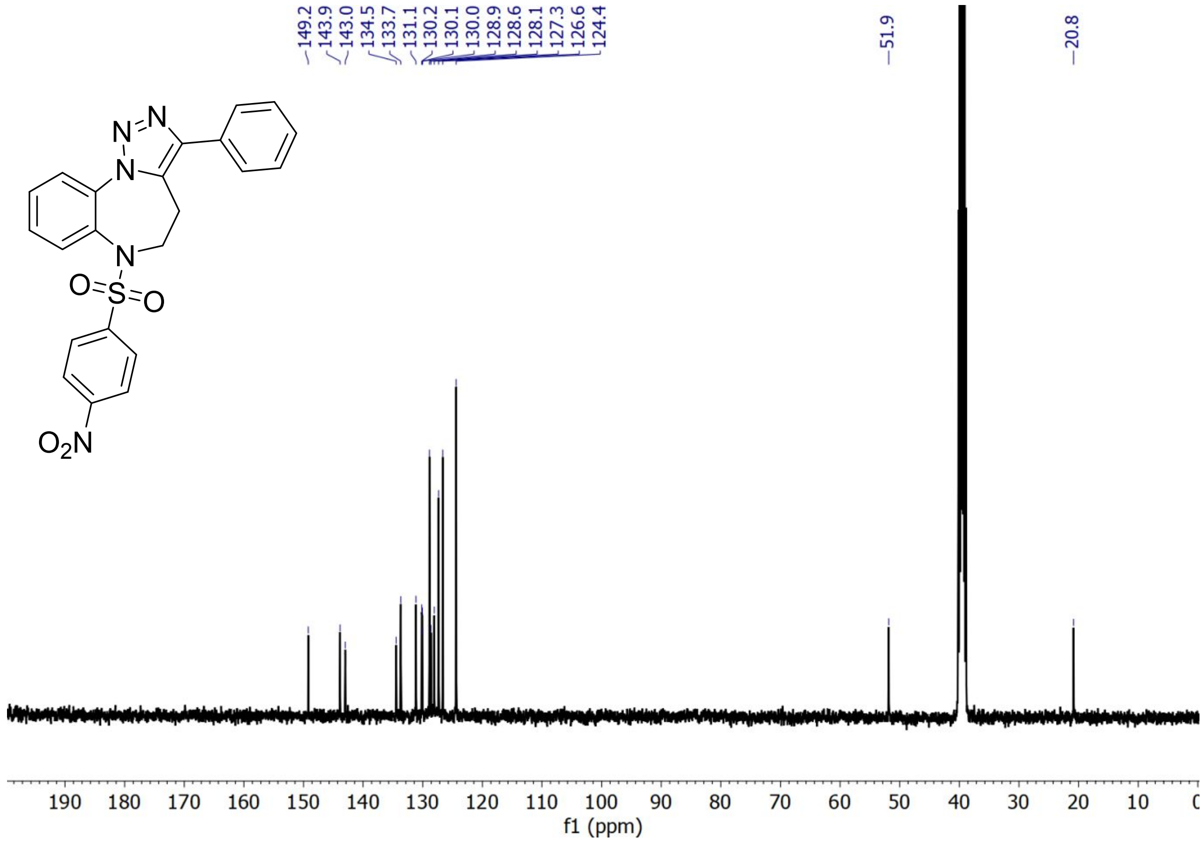

^{13}C NMR spectrum of **15a** (101 MHz, DMSO-d_6)

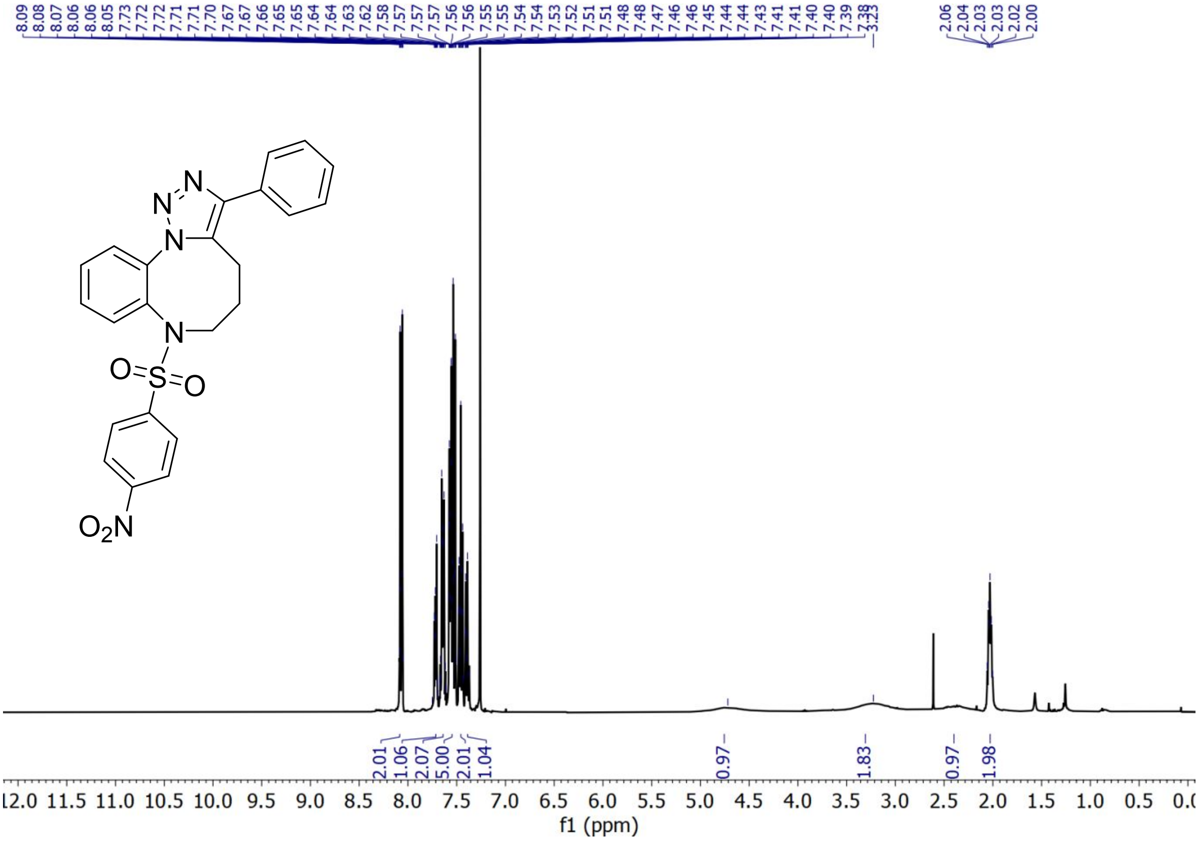

^{1}H NMR spectrum of **15b** (400 MHz, $CDCl_3$) – signal at 2.61 ppm belongs to DMSO

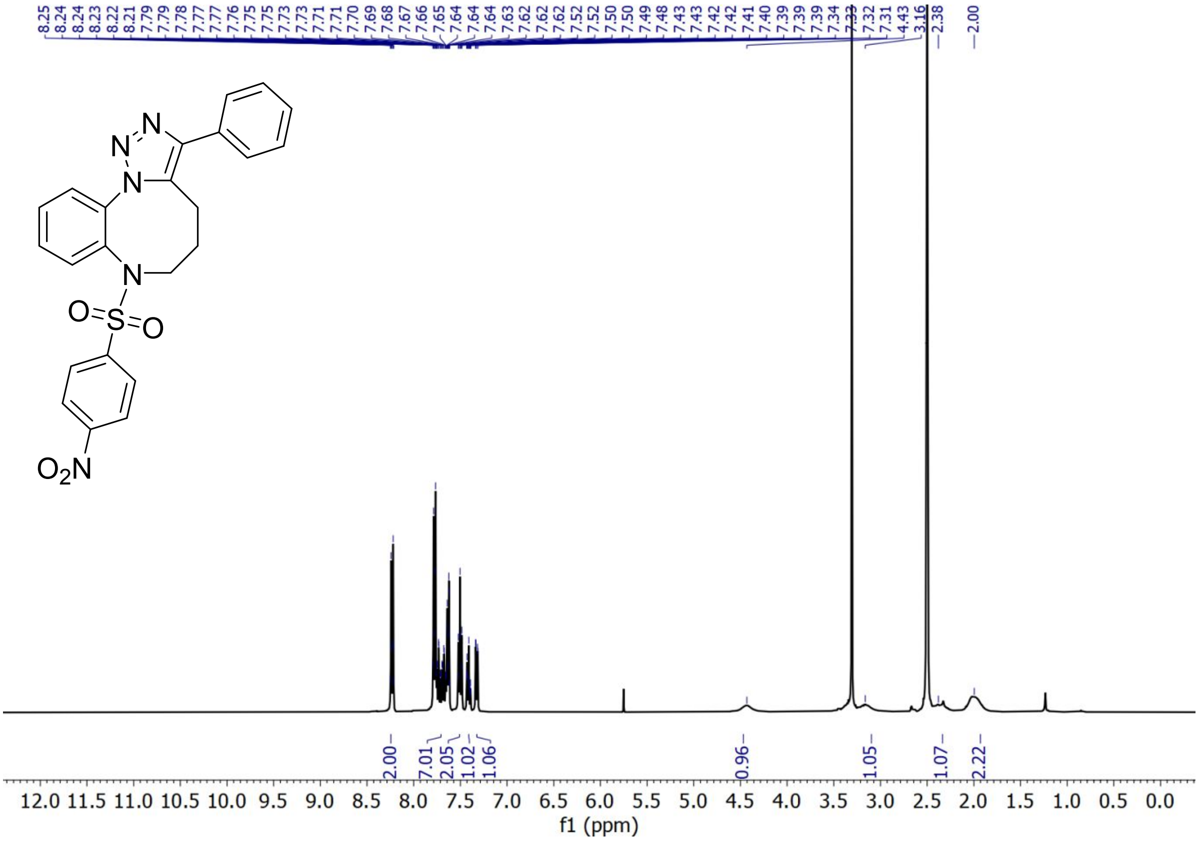

^{1}H NMR spectrum of **15b** (400 MHz, DMSO-d_6)

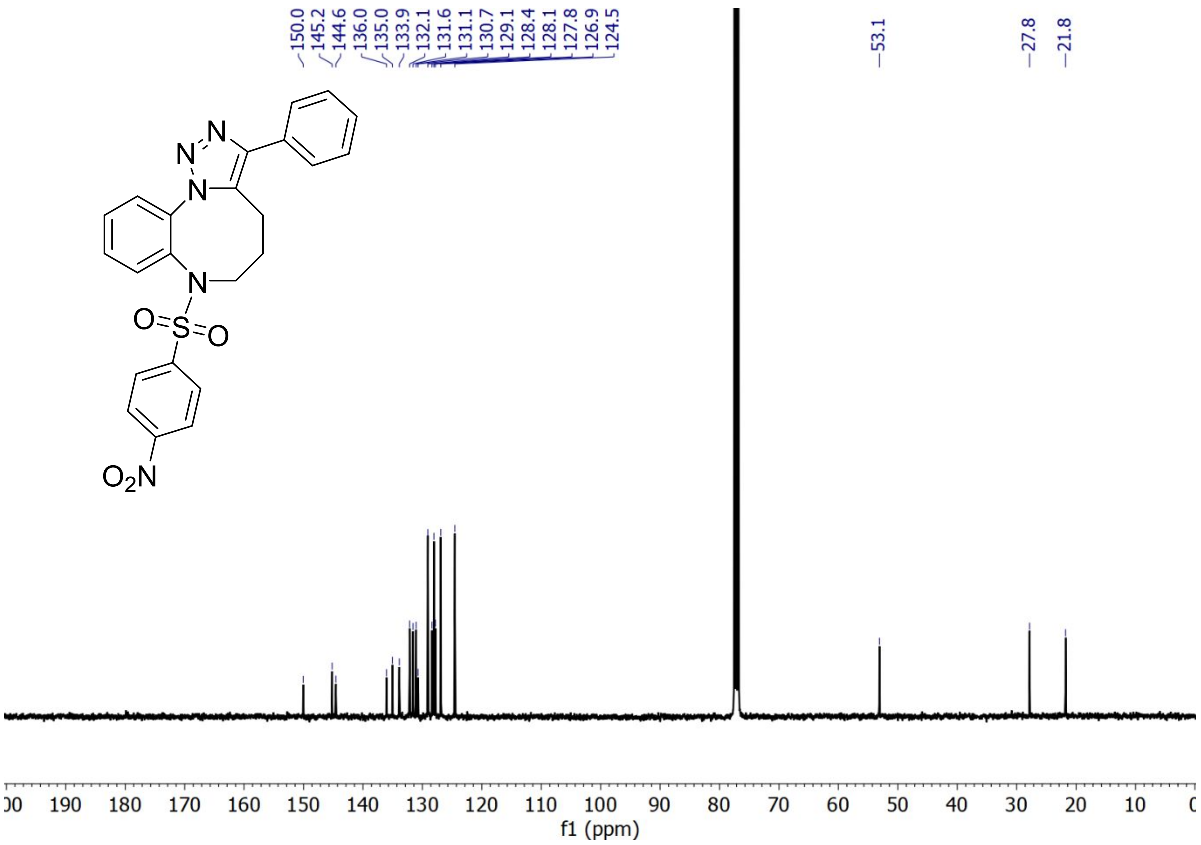

^{13}C NMR spectrum of **15b** (101 MHz, $CDCl_3$)

4. Copies of NMR spectra of products 17a-l, 16a-c, 18, 19, 20, 21, 22, 23, 24

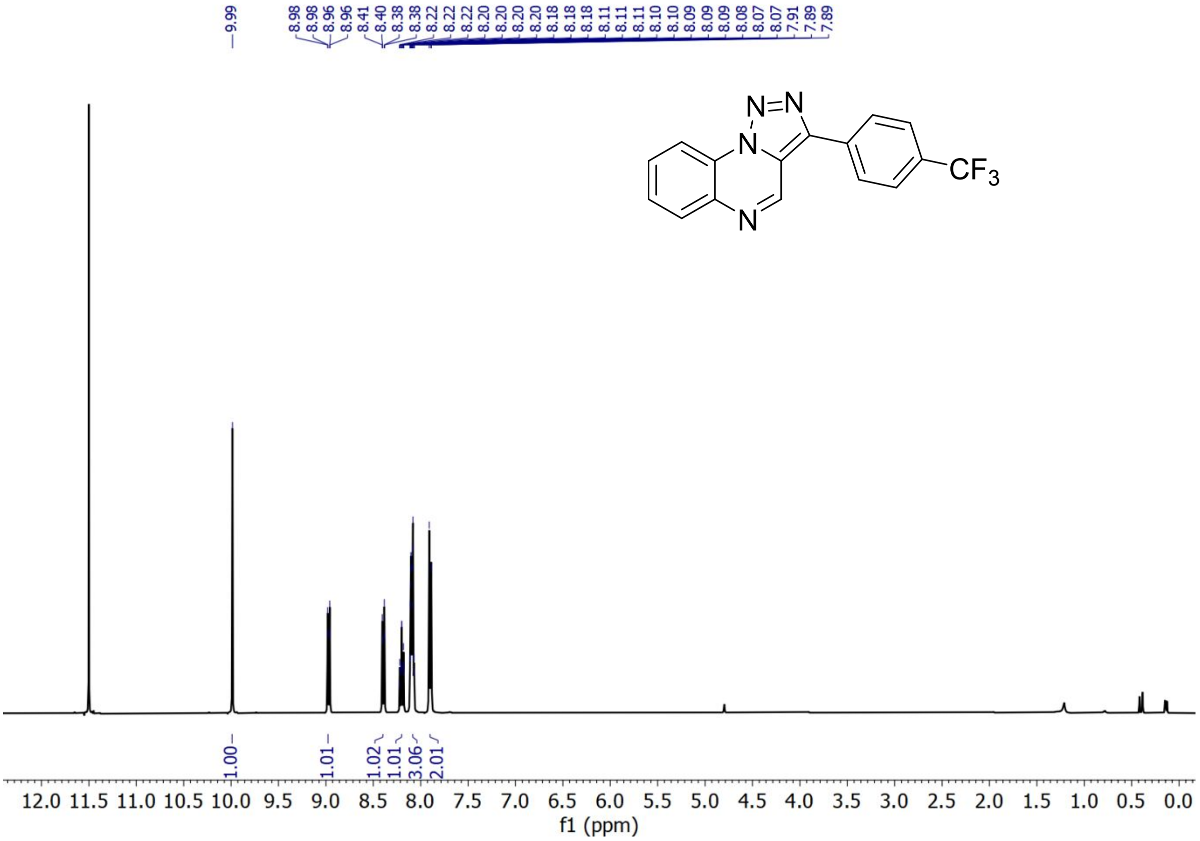

^{1}H NMR spectrum of **17c** (400 MHz, TFA-*d*).

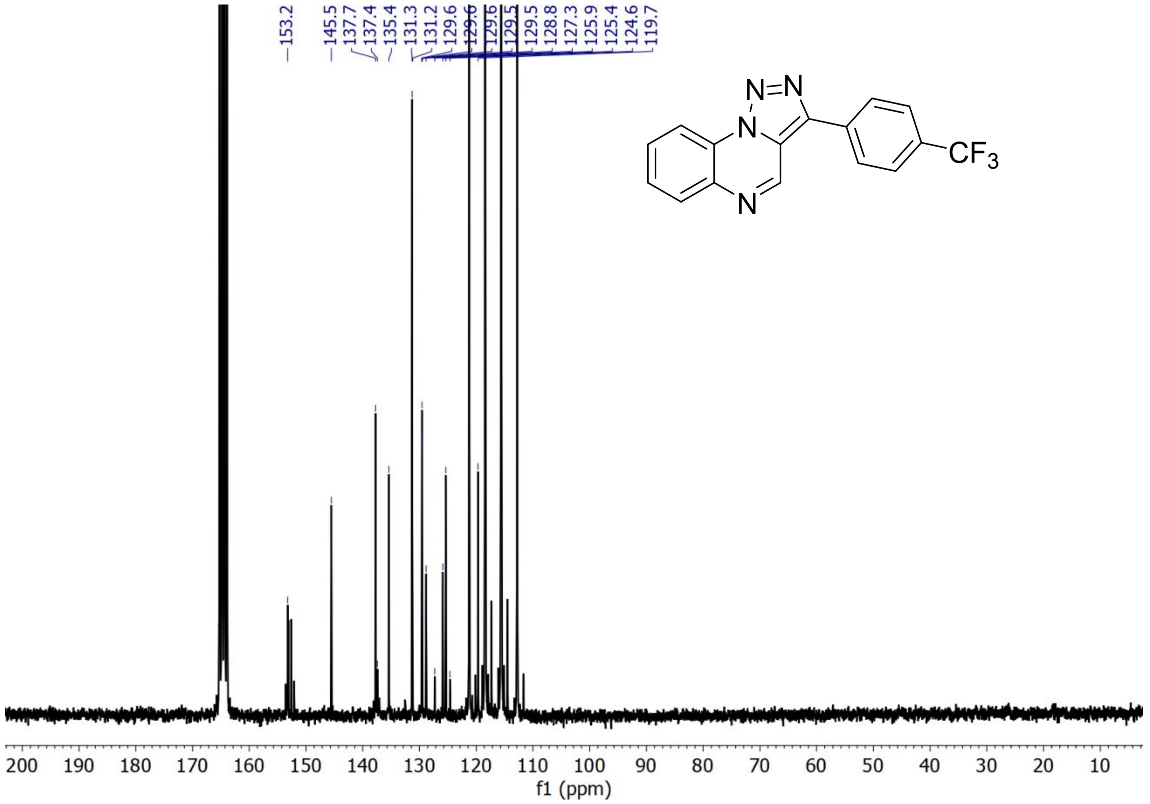

^{13}C NMR spectrum of **17c** (400 MHz, TFA-*d*) – impurities from TFA-*d*: δ 152.8 (q, *J* = 48.8 Hz, 116 (q, *J* = 284.5 Hz)

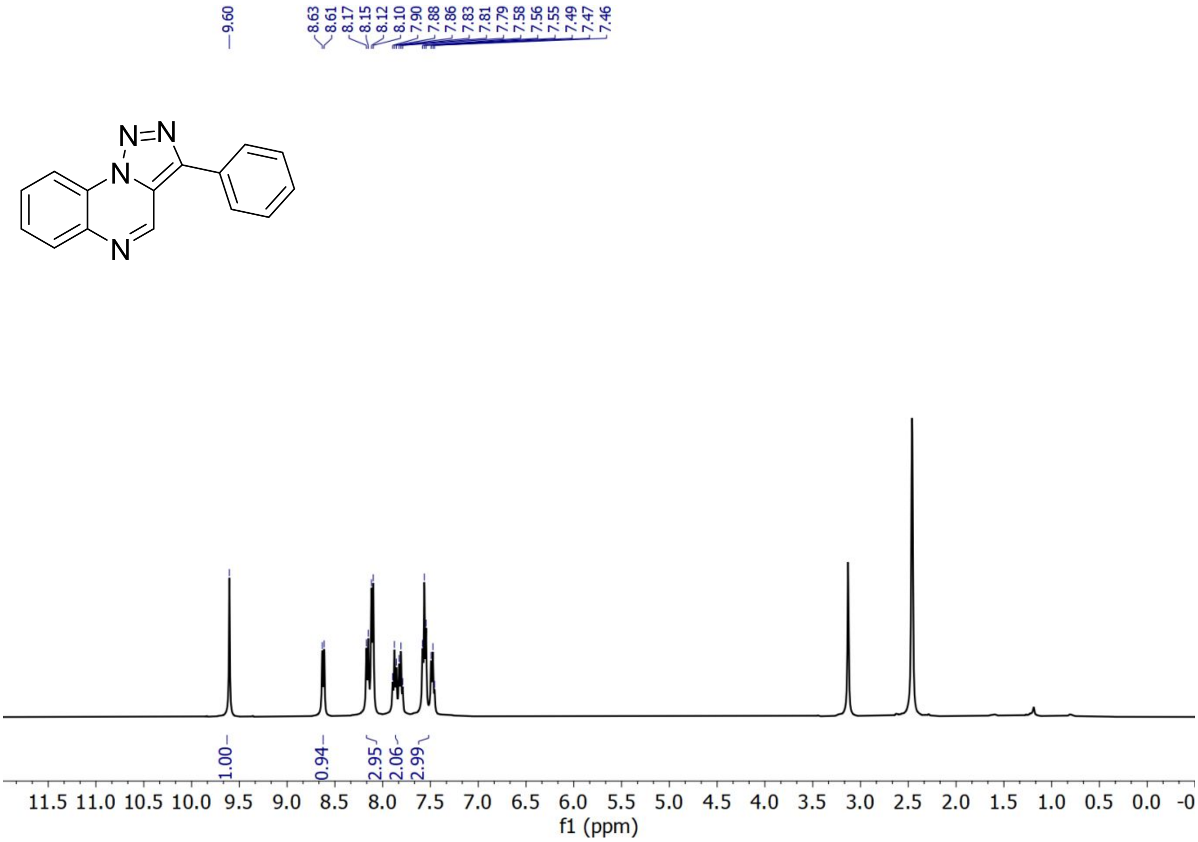

^{1}H NMR spectrum of **17d** (400 MHz, DMSO-d_6)

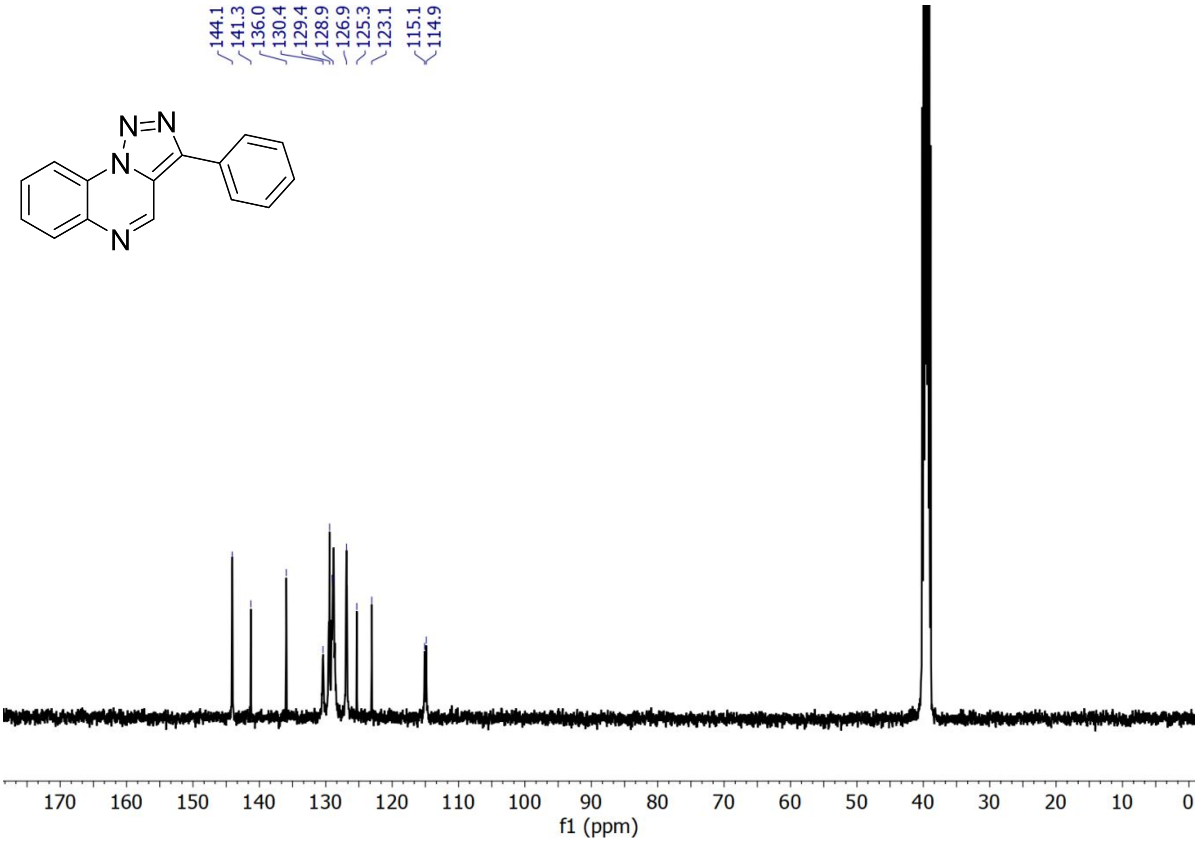

^{13}C NMR spectrum of **17d** (101 MHz, DMSO-d_6)

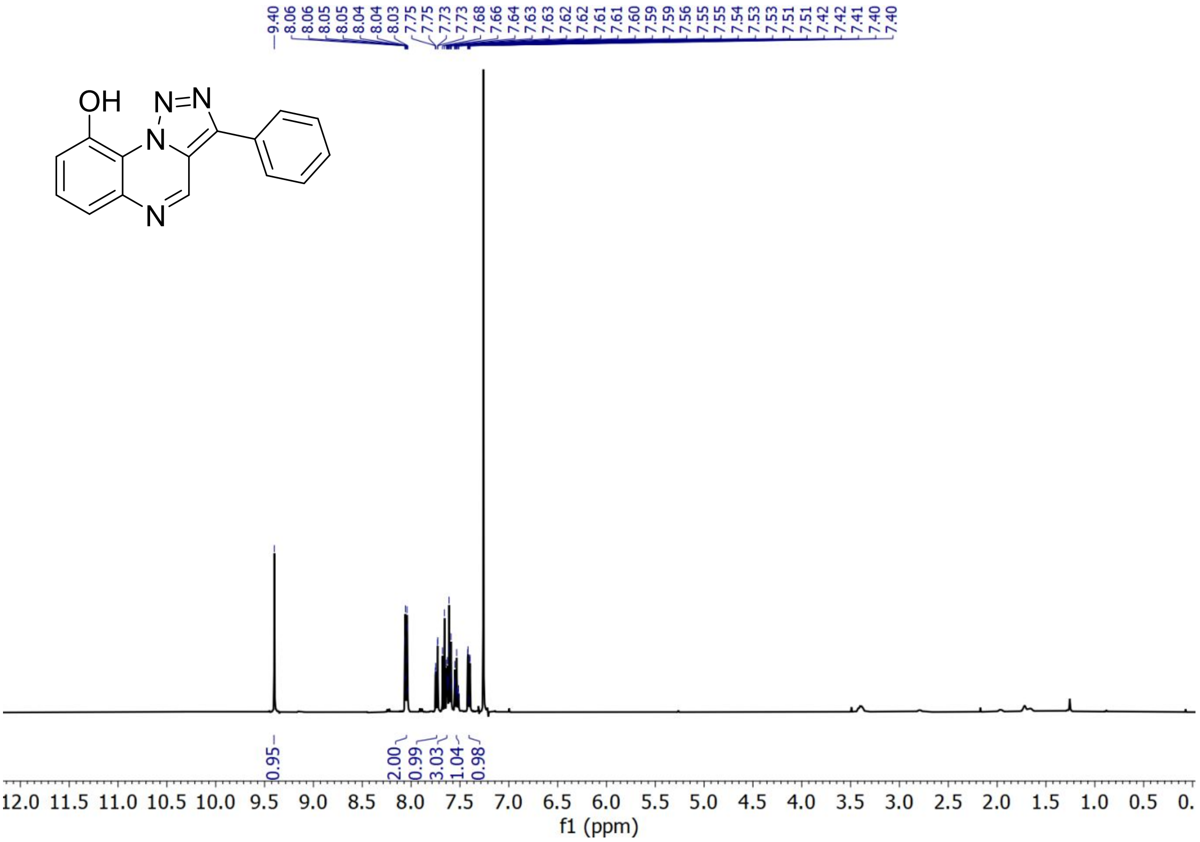

^{1}H NMR spectrum of **17e** (400 MHz, $CDCl_3$)

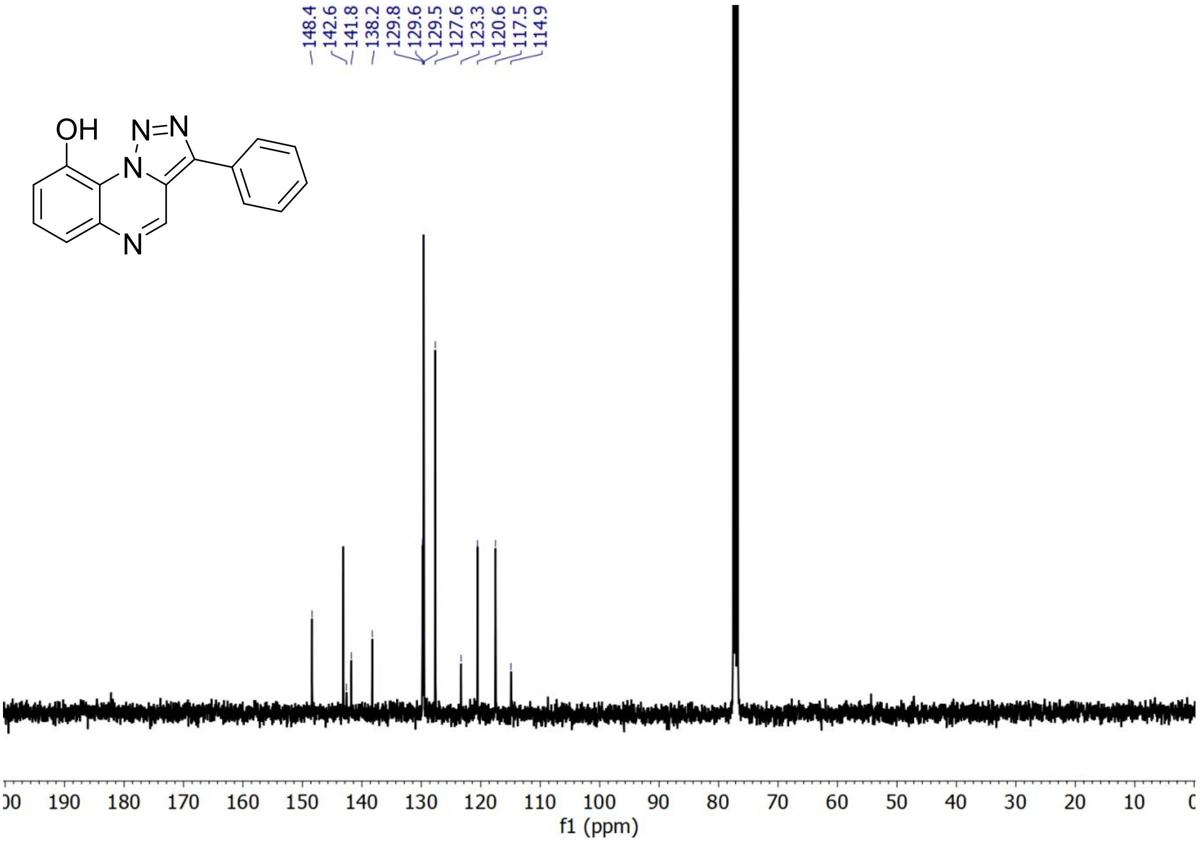

^{13}C NMR spectrum of **17e** (101 MHz, $CDCl_3$)

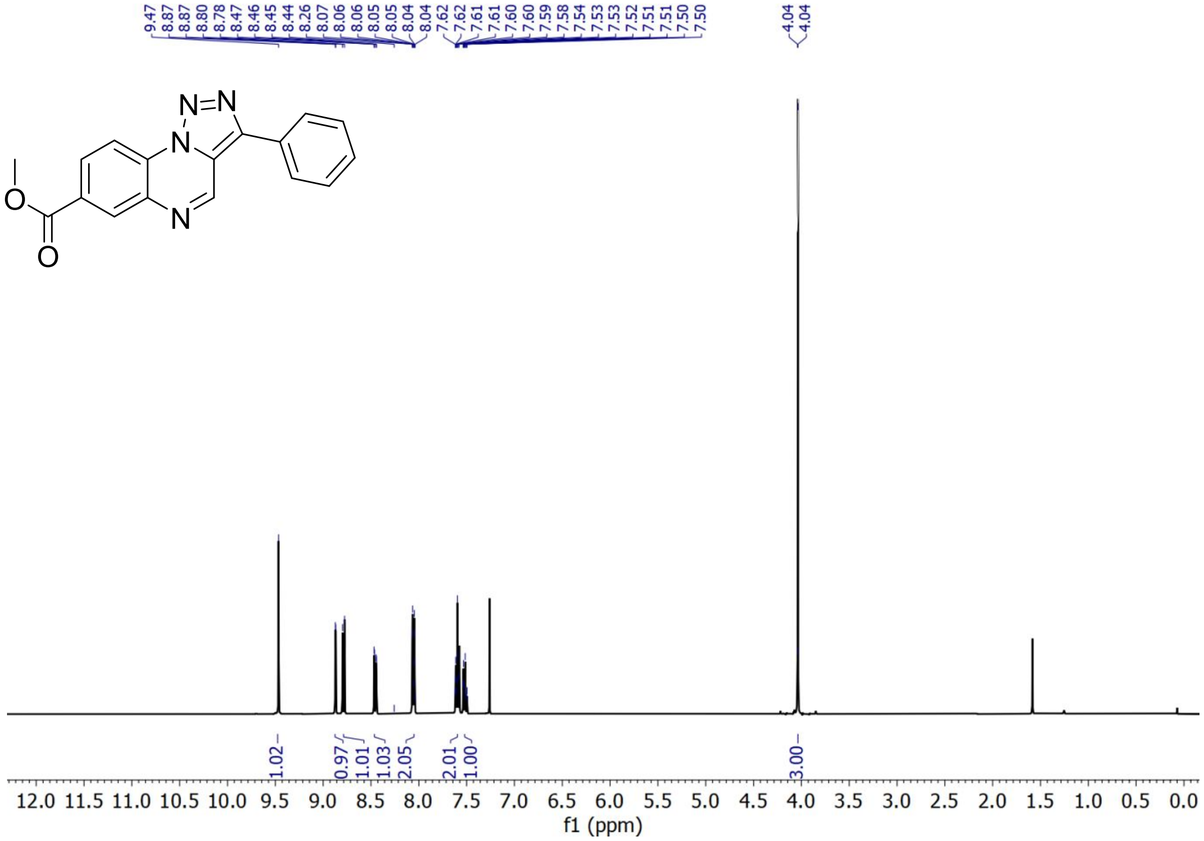

^{1}H NMR spectrum of **17f** (400 MHz, $CDCl_3$)

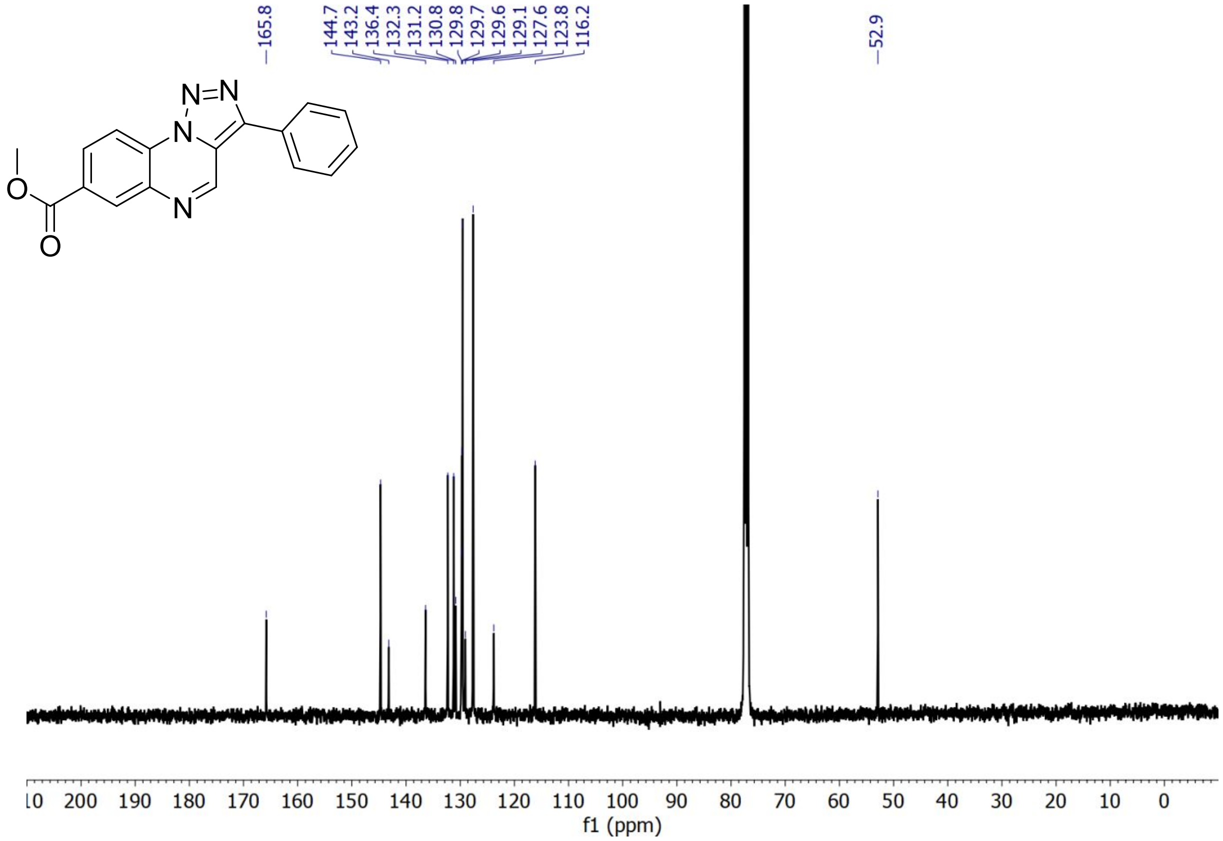

^{13}C NMR spectrum of **17f** (101 MHz, $CDCl_3$)

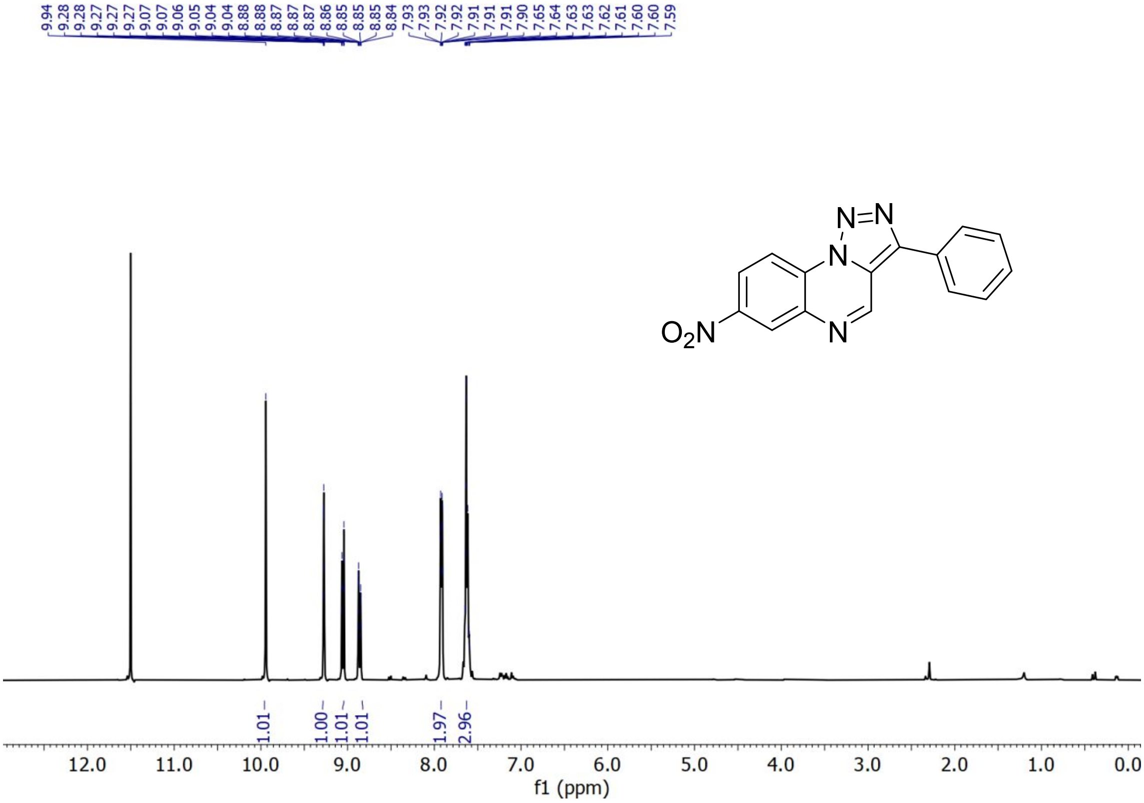

^{1}H NMR spectrum of **17g** in (400 MHz, TFA-*d*)

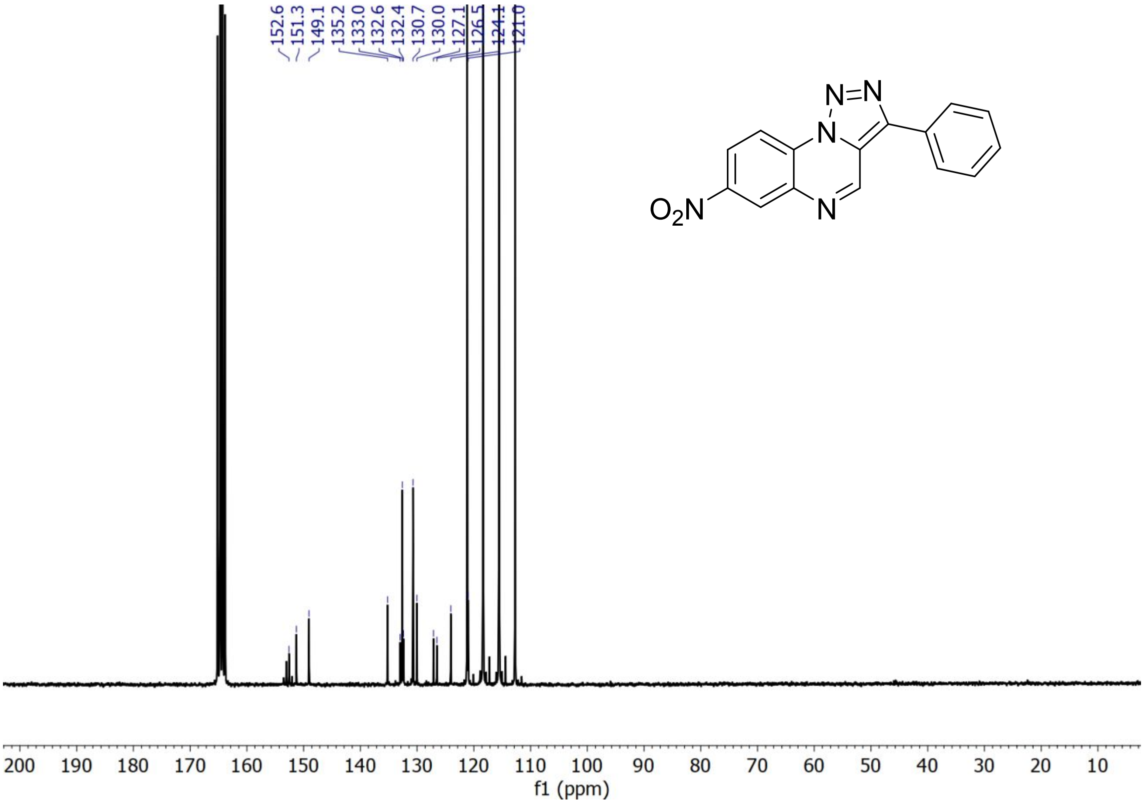

^{13}C NMR spectrum of **17g** (101 MHz, TFA-*d*) impurities from TFA-*d*: δ 152.8 (q, *J* = 48.8 Hz, 116 (q, *J* = 284.5 Hz).

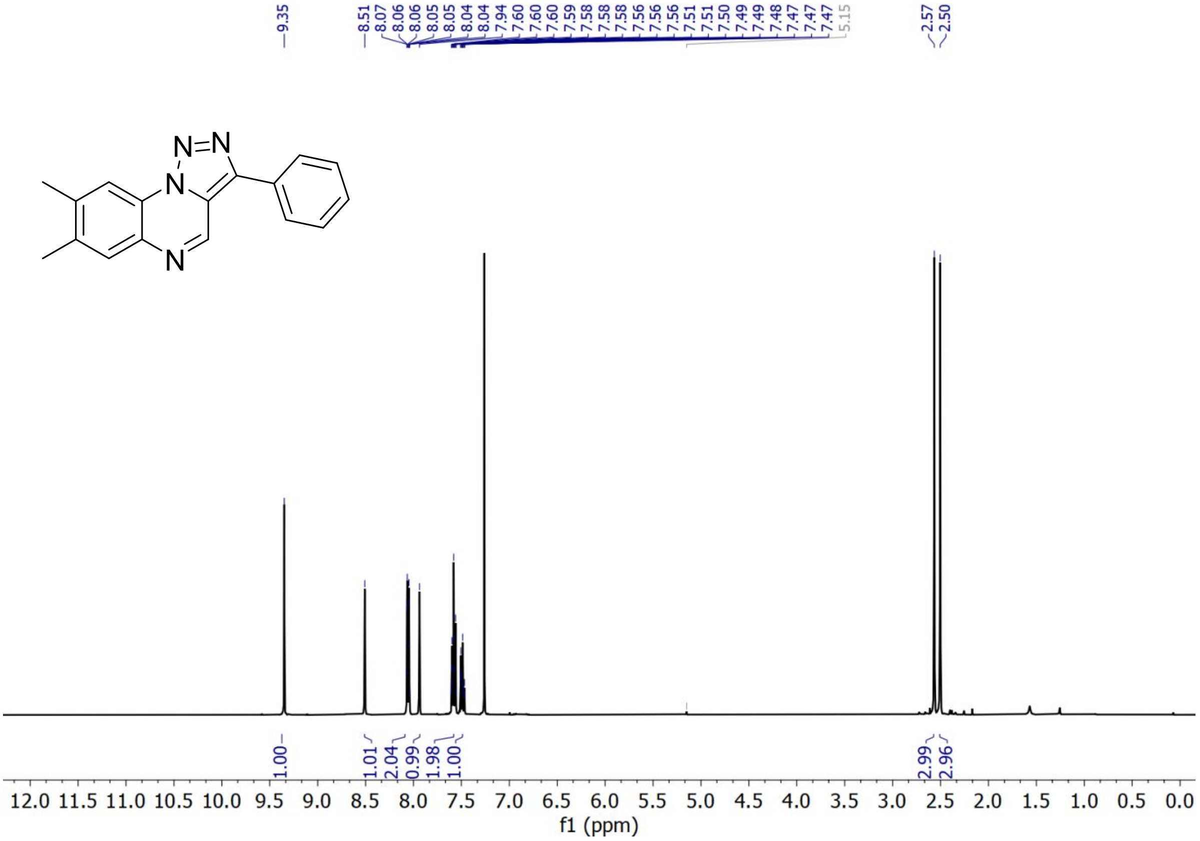

^{1}H NMR spectrum of **17h** (400 MHz, $CDCl_3$)

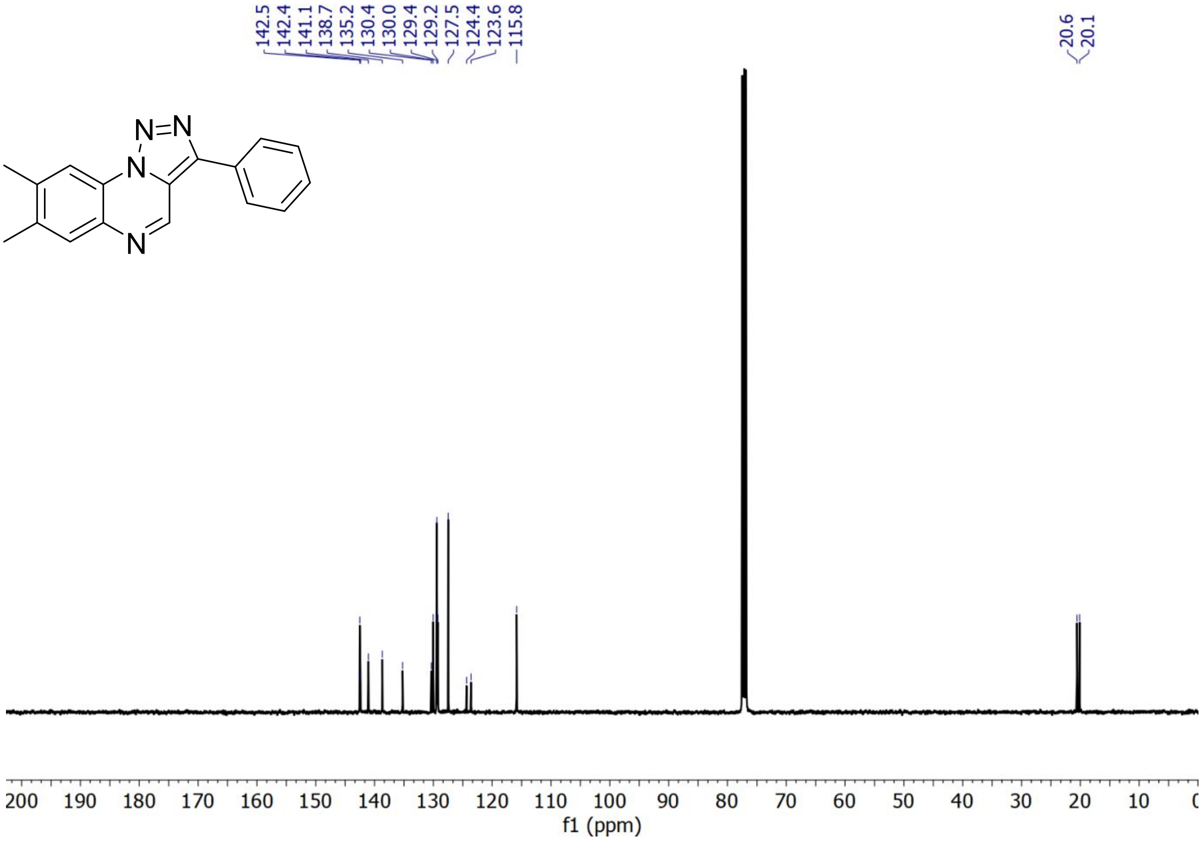

^{13}C NMR spectrum of **17h** (101 MHz, $CDCl_3$)

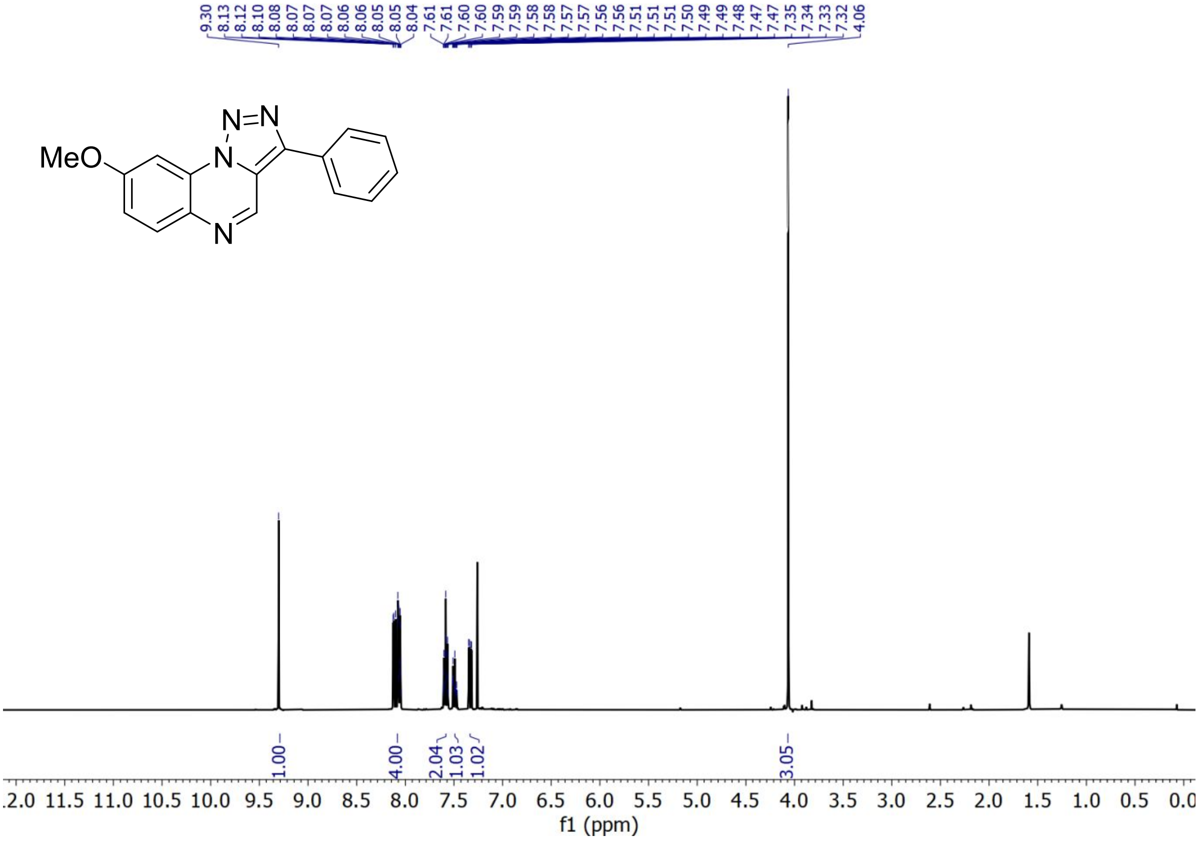

^{1}H NMR spectrum of **17i** (400 MHz, $CDCl_3$)

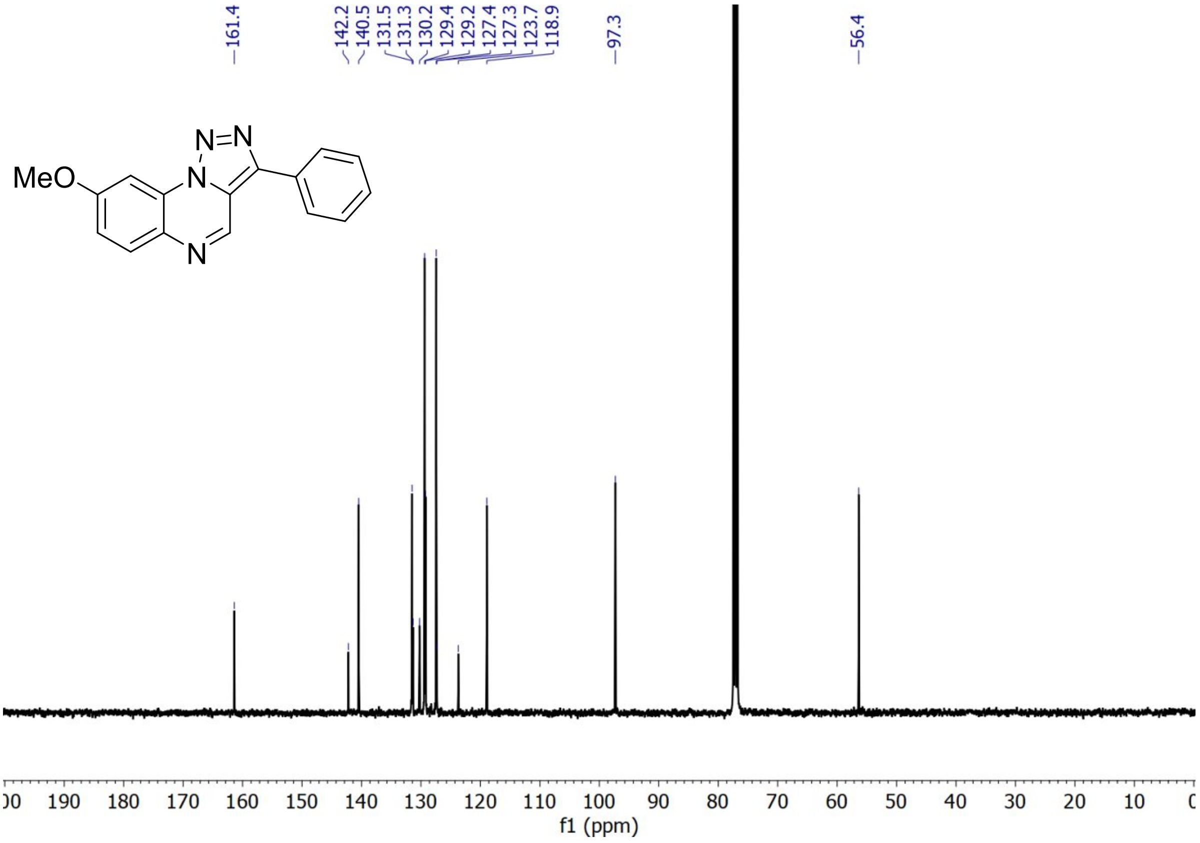

^{13}C NMR spectrum of **17i** (101 MHz, $CDCl_3$)

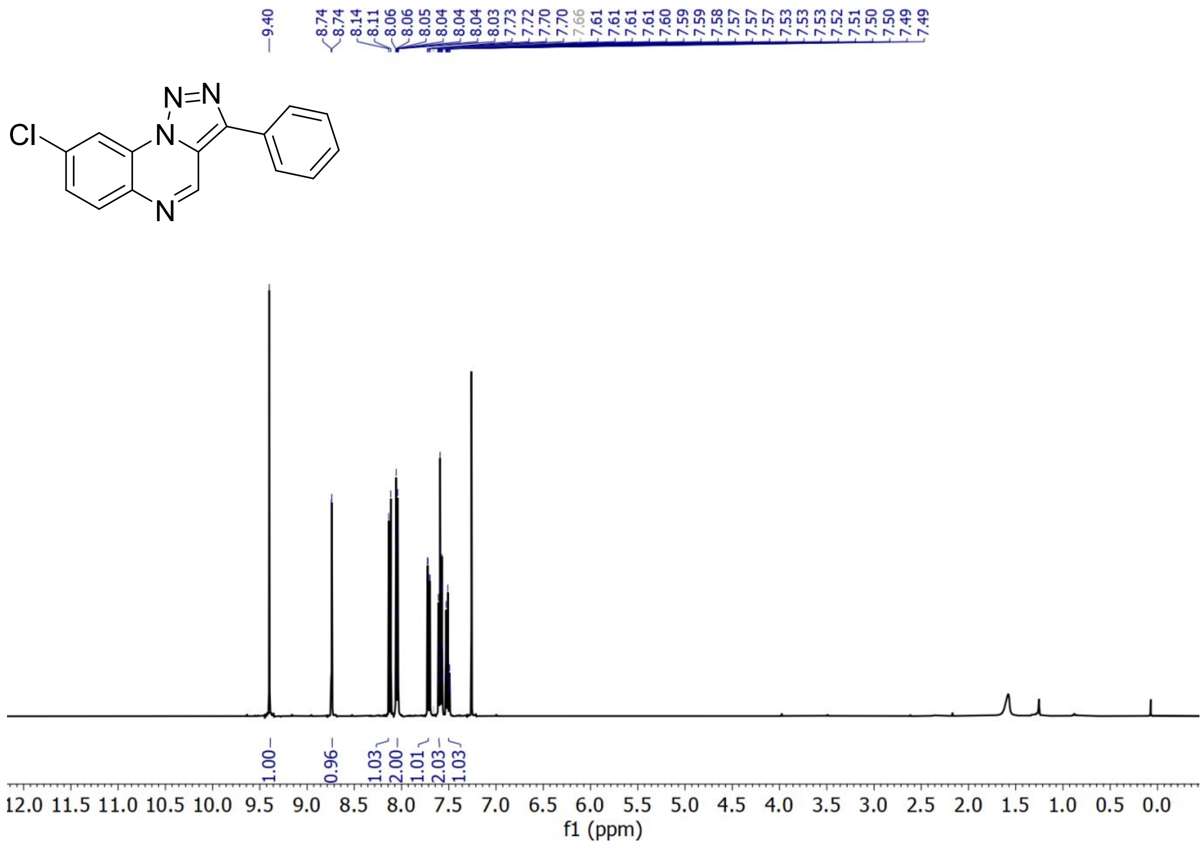

^{1}H NMR spectrum of **17j** (400 MHz, $CDCl_3$)

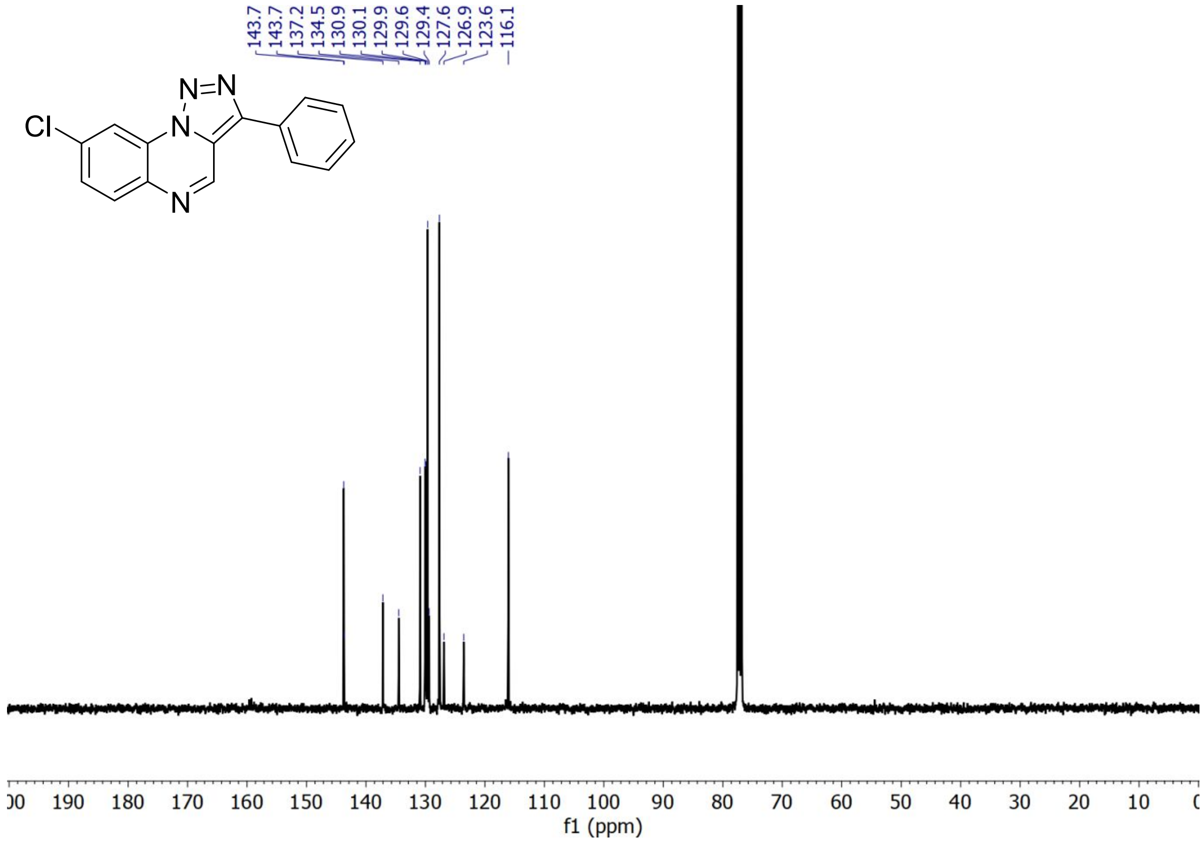

^{13}C NMR spectrum of **17j** (101 MHz, $CDCl_3$)

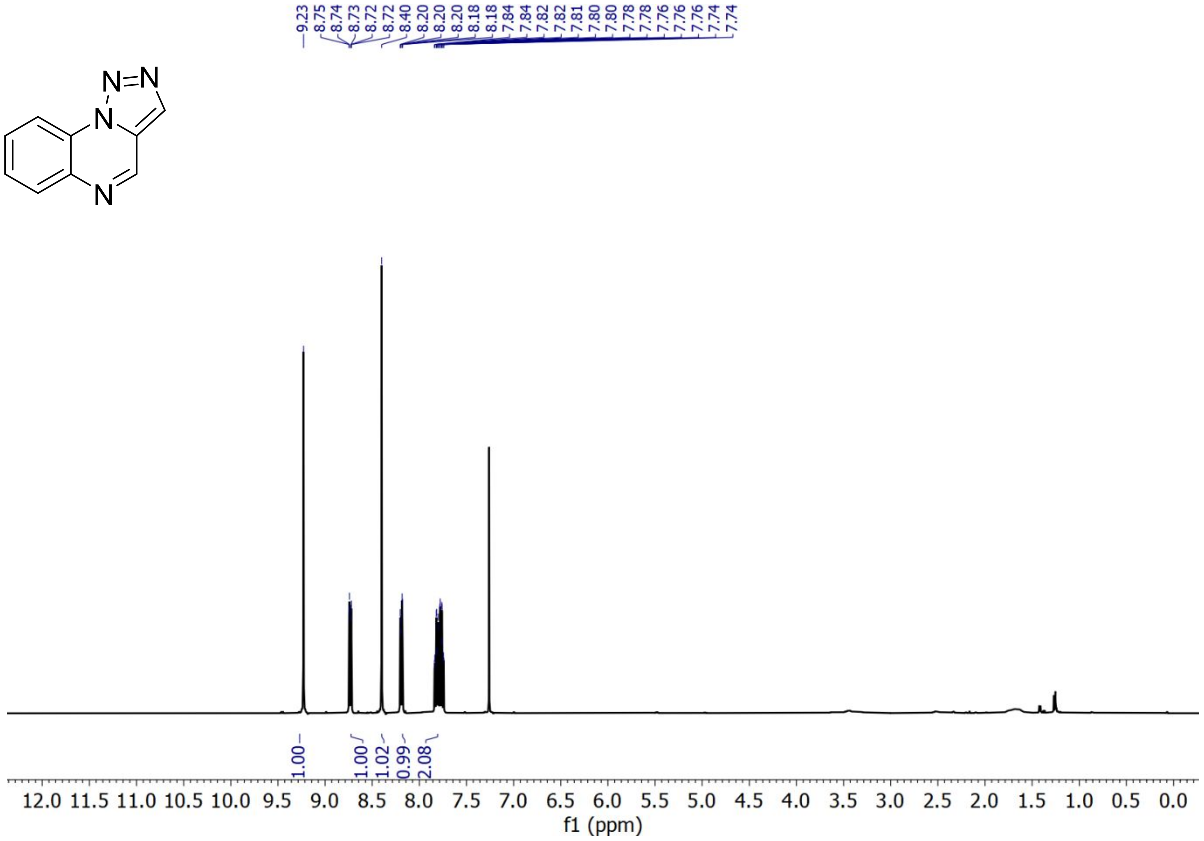

^{1}H NMR spectrum of **17k** (400 MHz, $CDCl_3$)

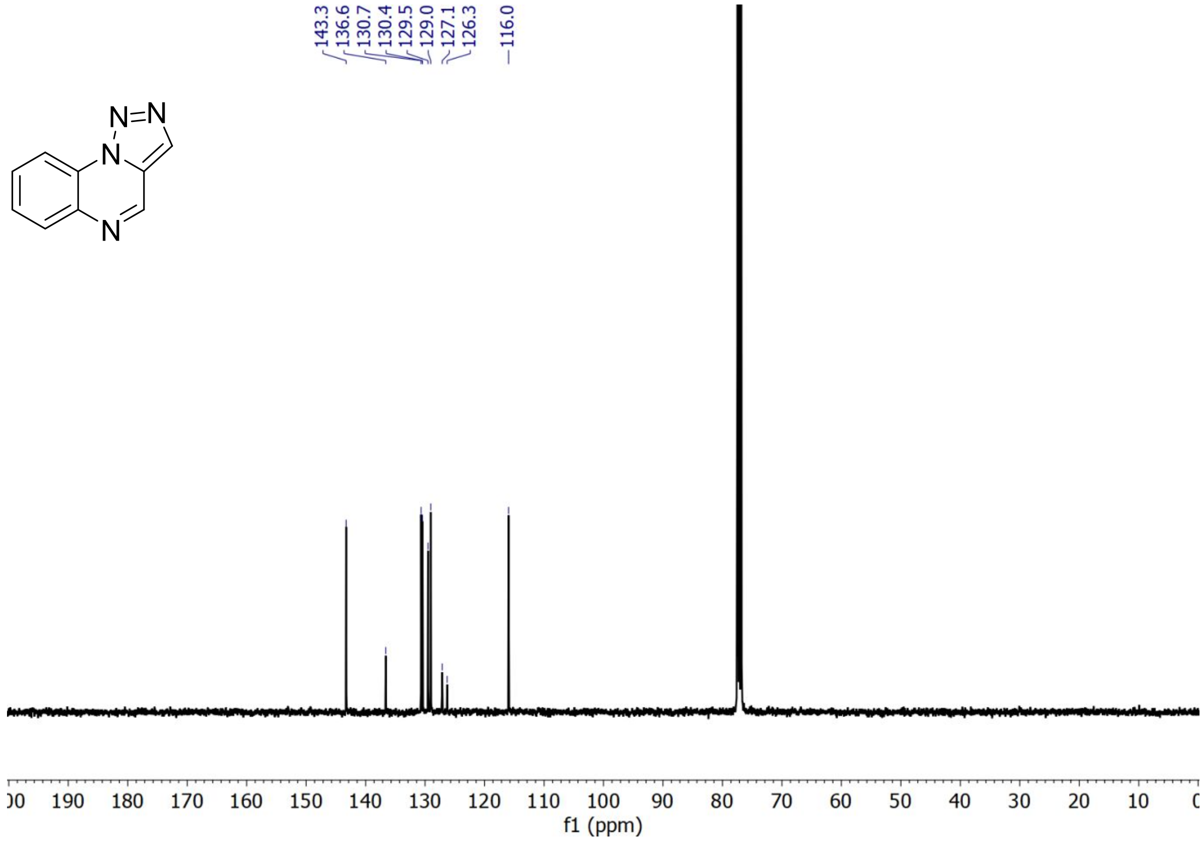

^{13}C NMR spectrum of **17k** (101 MHz, $CDCl_3$)

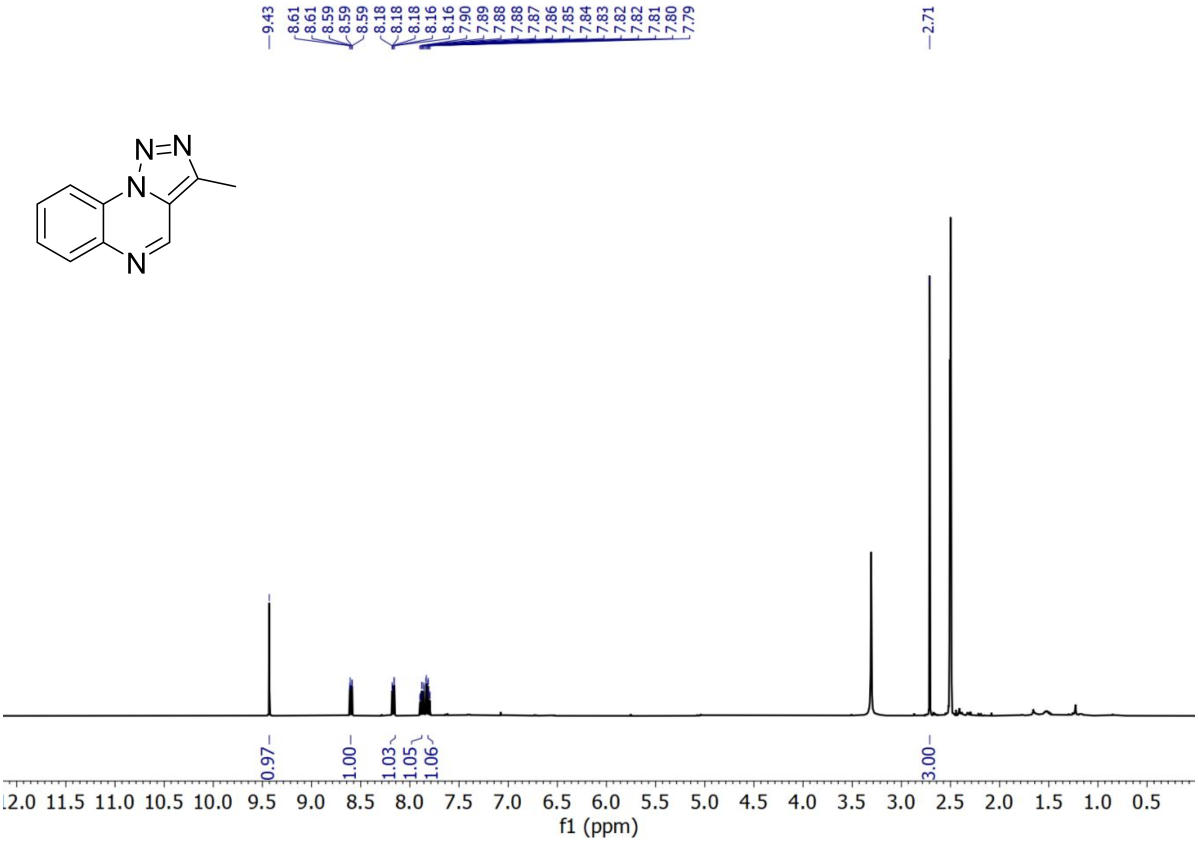

^{1}H NMR spectrum of **17l** (400 MHz, DMSO-d_6)

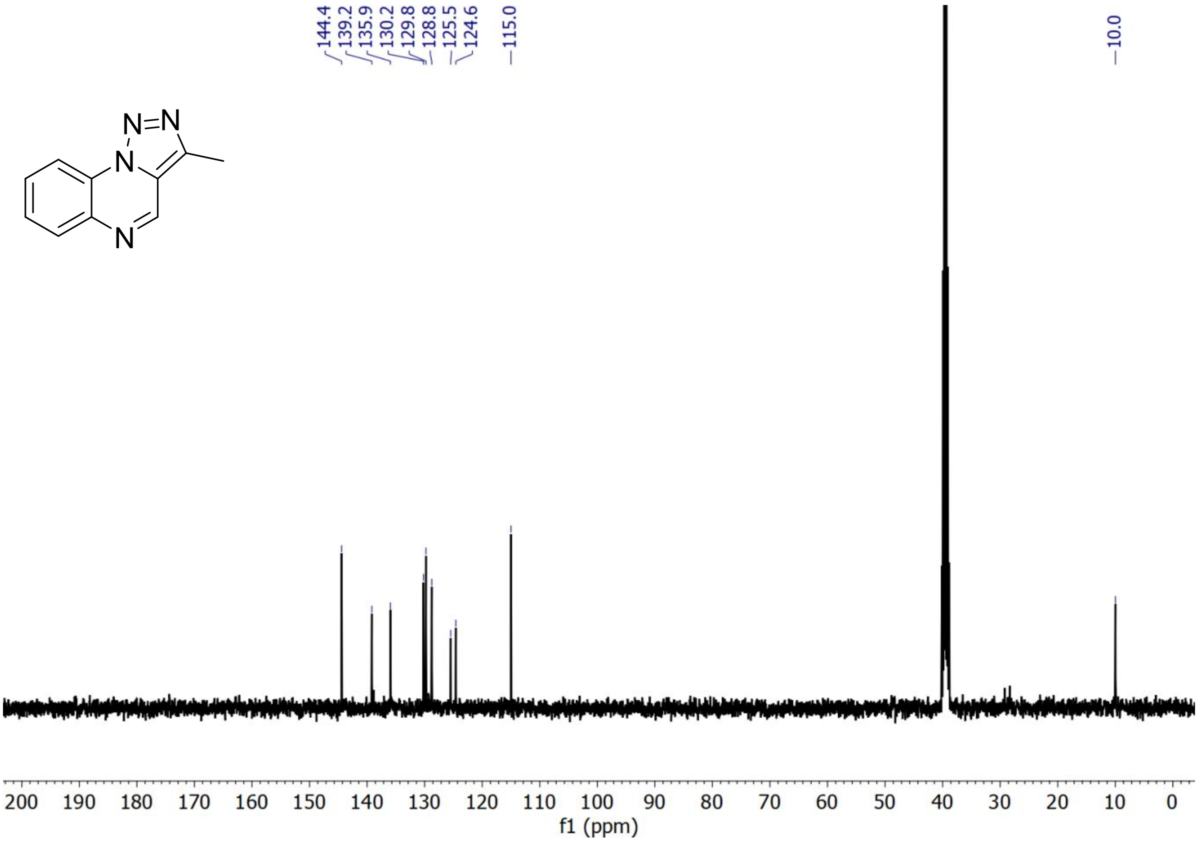

^{13}C NMR spectrum of **17l** (101 MHz, DMSO-d_6)

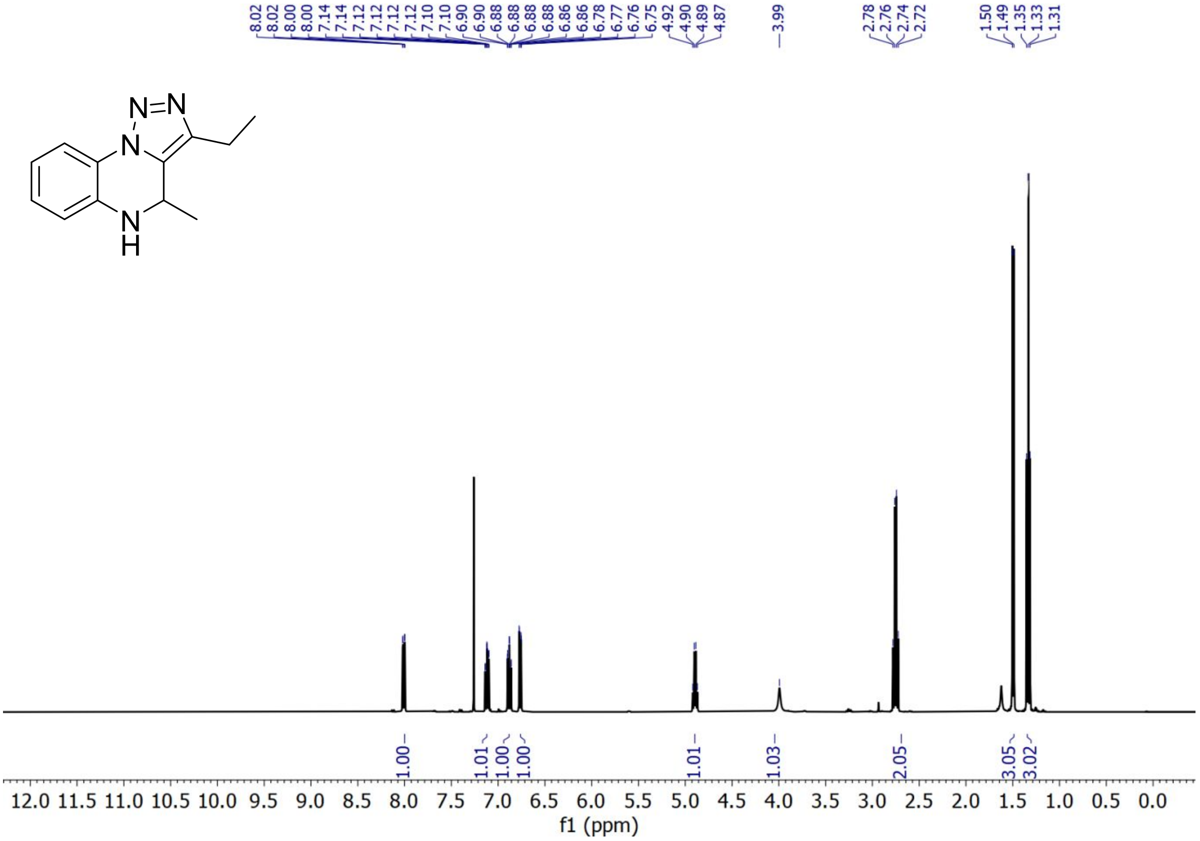

^{1}H NMR spectrum of **16a** (400 MHz, $CDCl_3$)

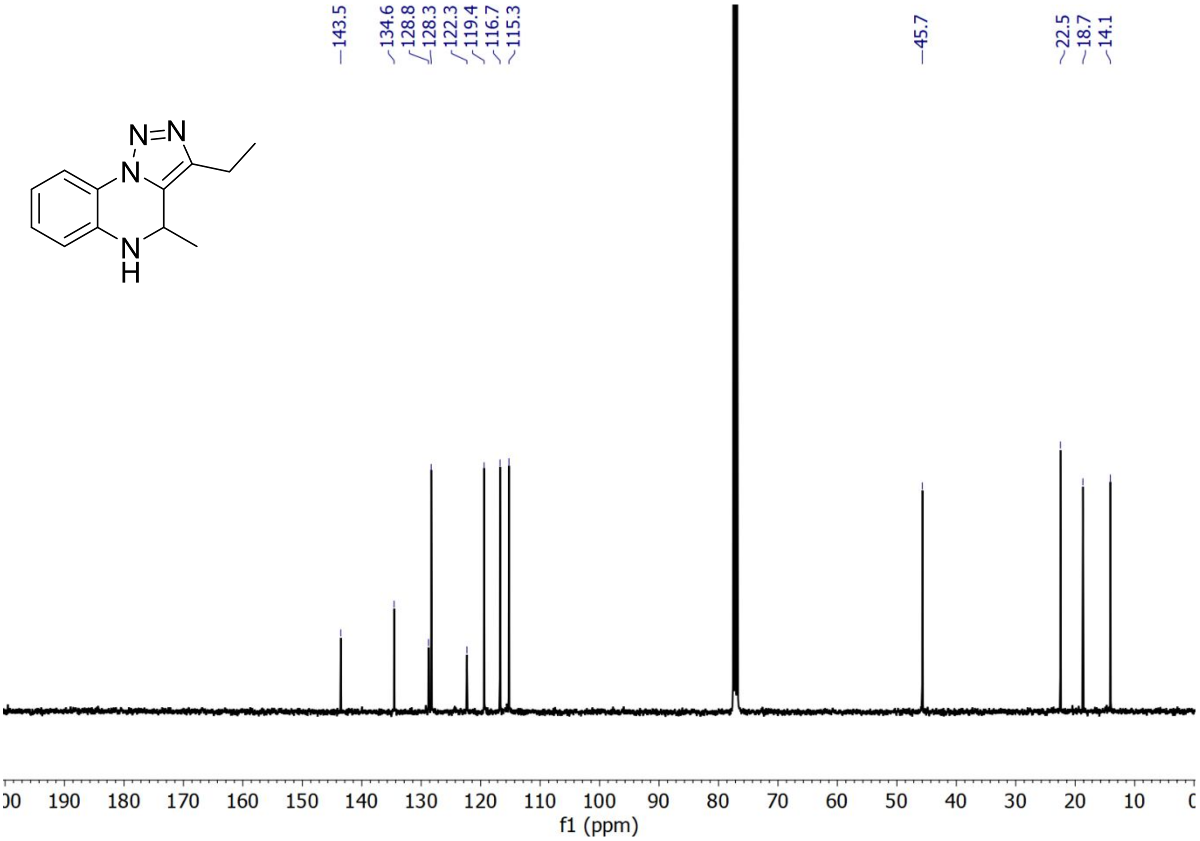

^{13}C NMR spectrum of **16a** (101 MHz, $CDCl_3$)

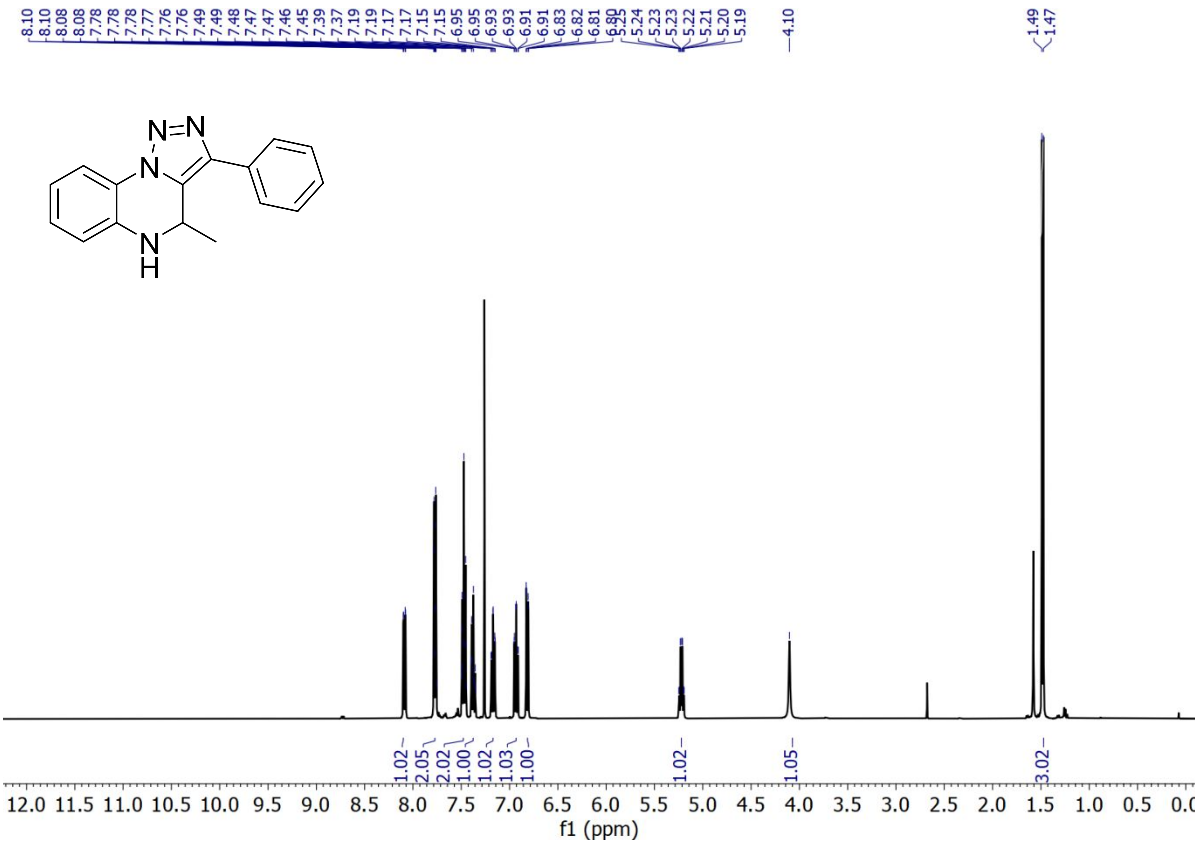

^{1}H NMR spectrum of **16b** (400 MHz, $CDCl_3$)

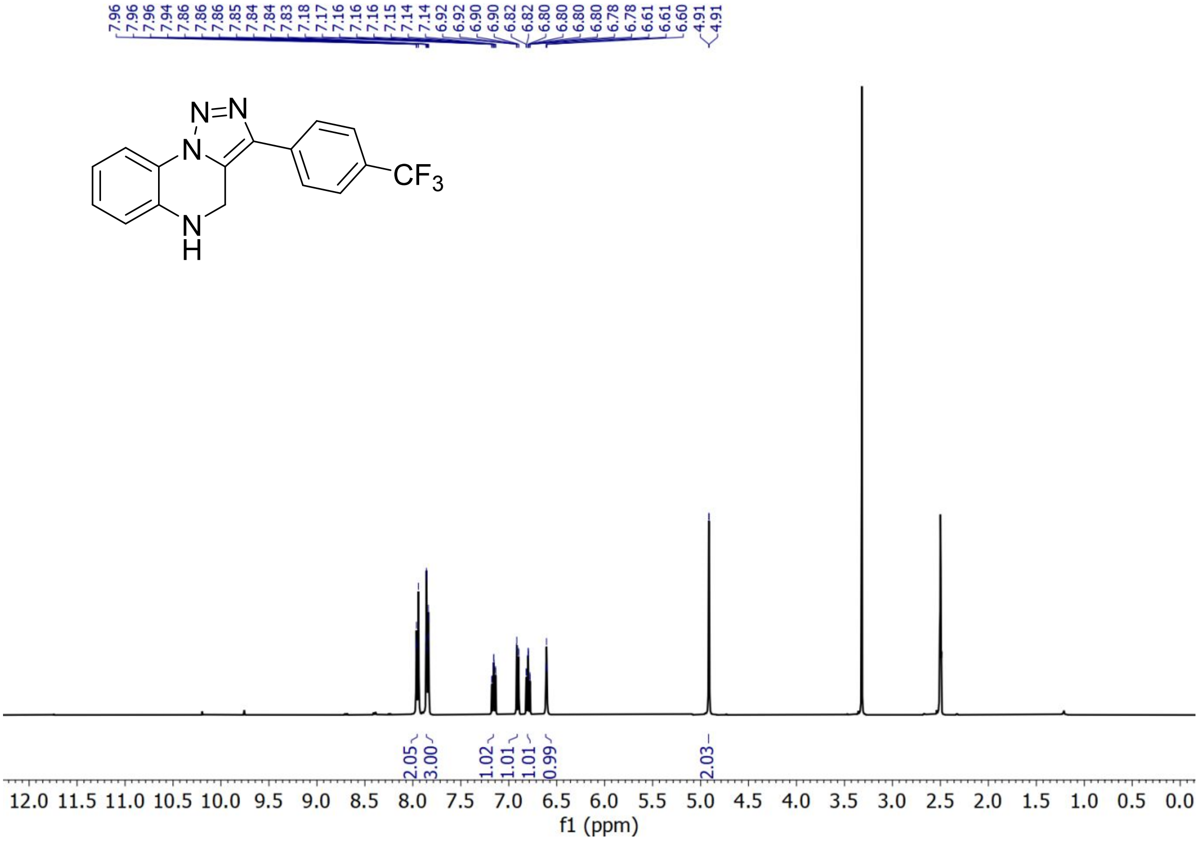

^{1}H NMR spectrum of **16c** (400 MHz, DMSO-d_6)

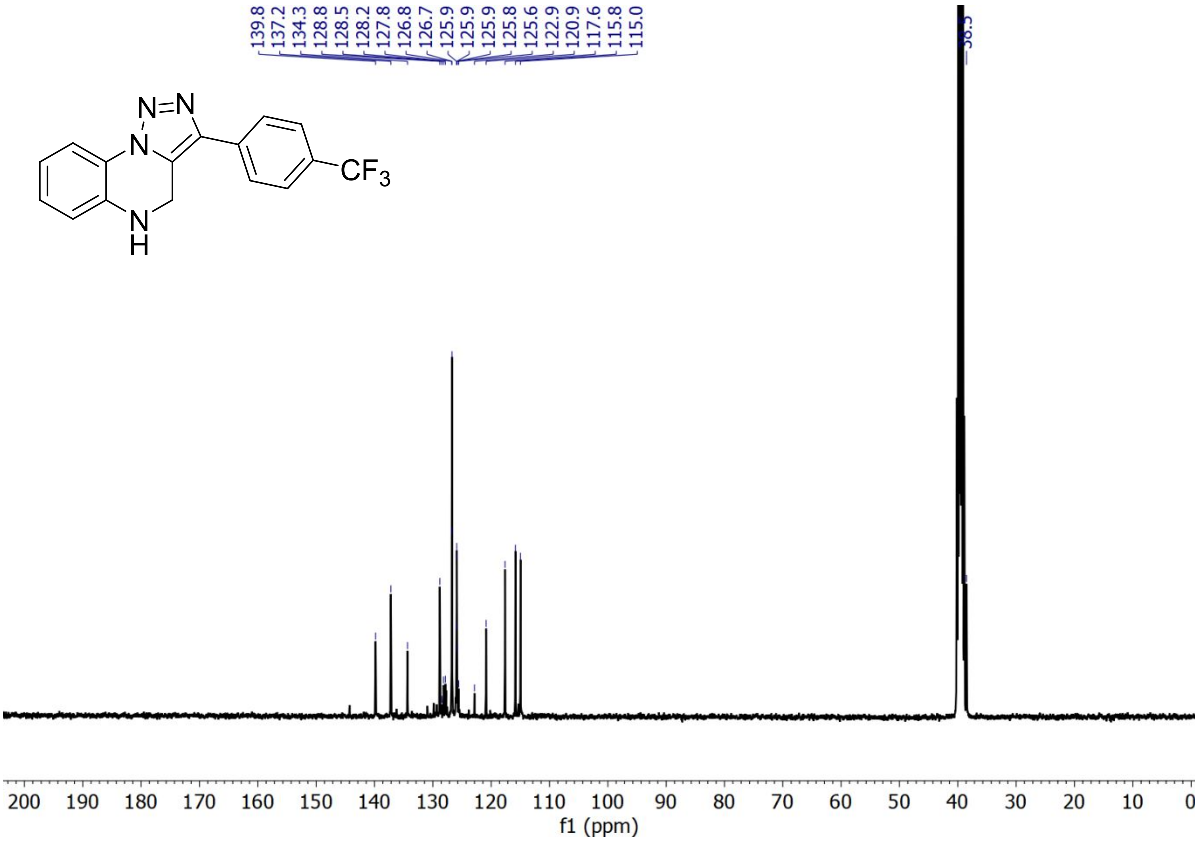

^{13}C NMR spectrum of **16c** (101 MHz, DMSO-d_6) - during the measurement, **16c** was partially oxidized by DMSO, which gave interfering signals

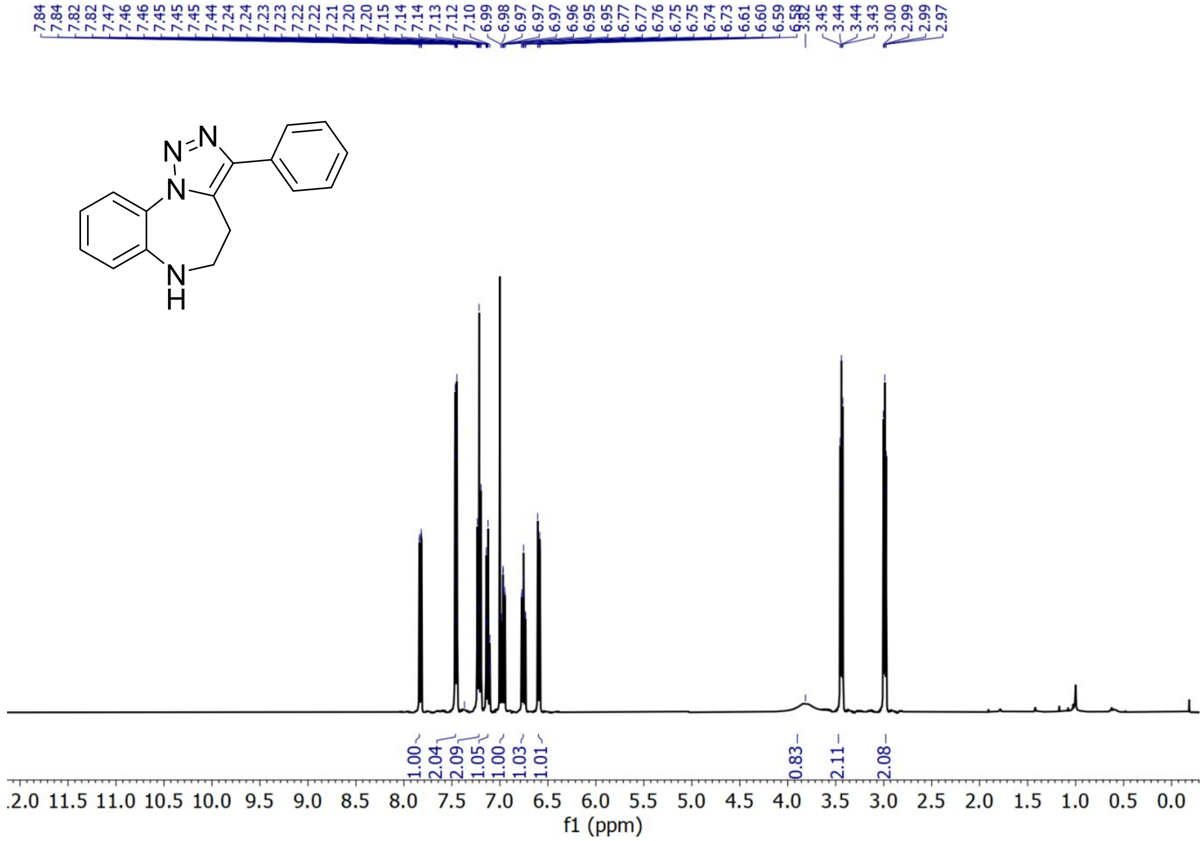

^{1}H NMR spectrum of **18** (400 MHz, $CDCl_3$)

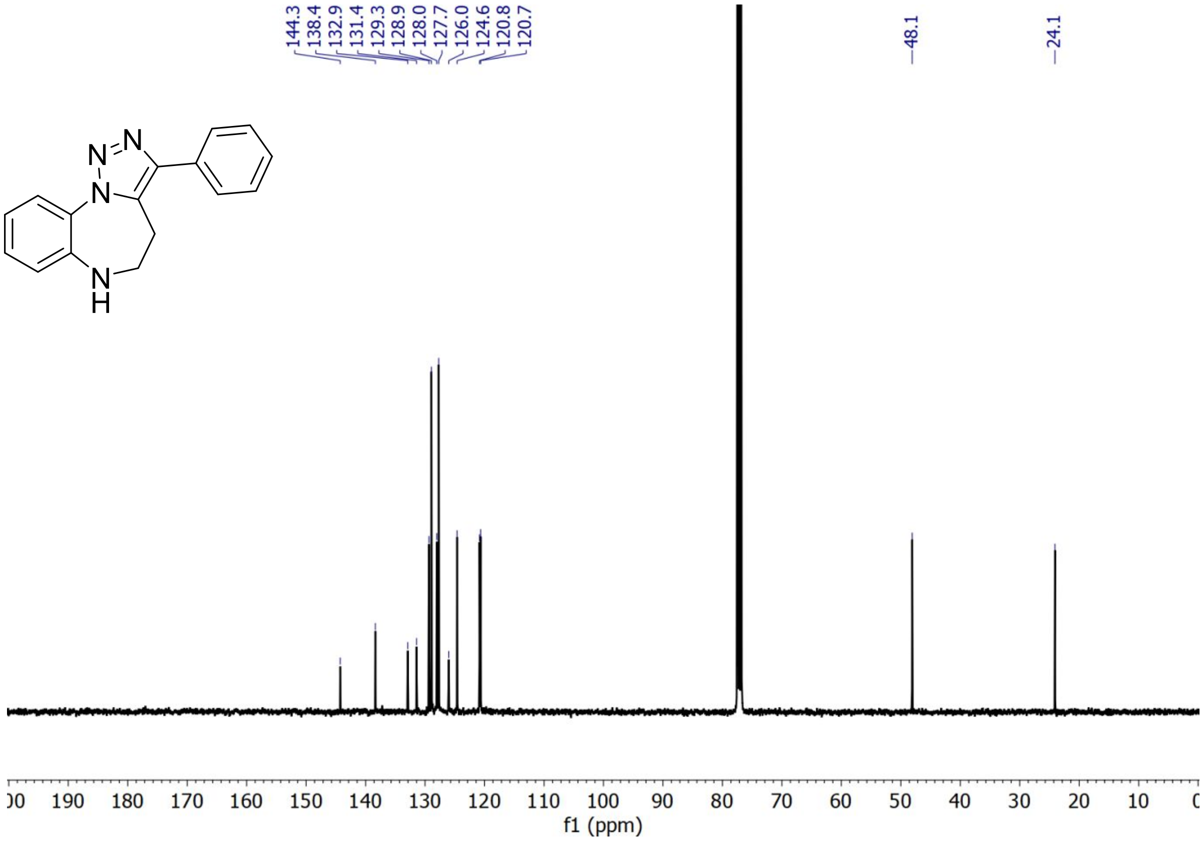

^{13}C NMR spectrum of **18** (101 MHz, $CDCl_3$)

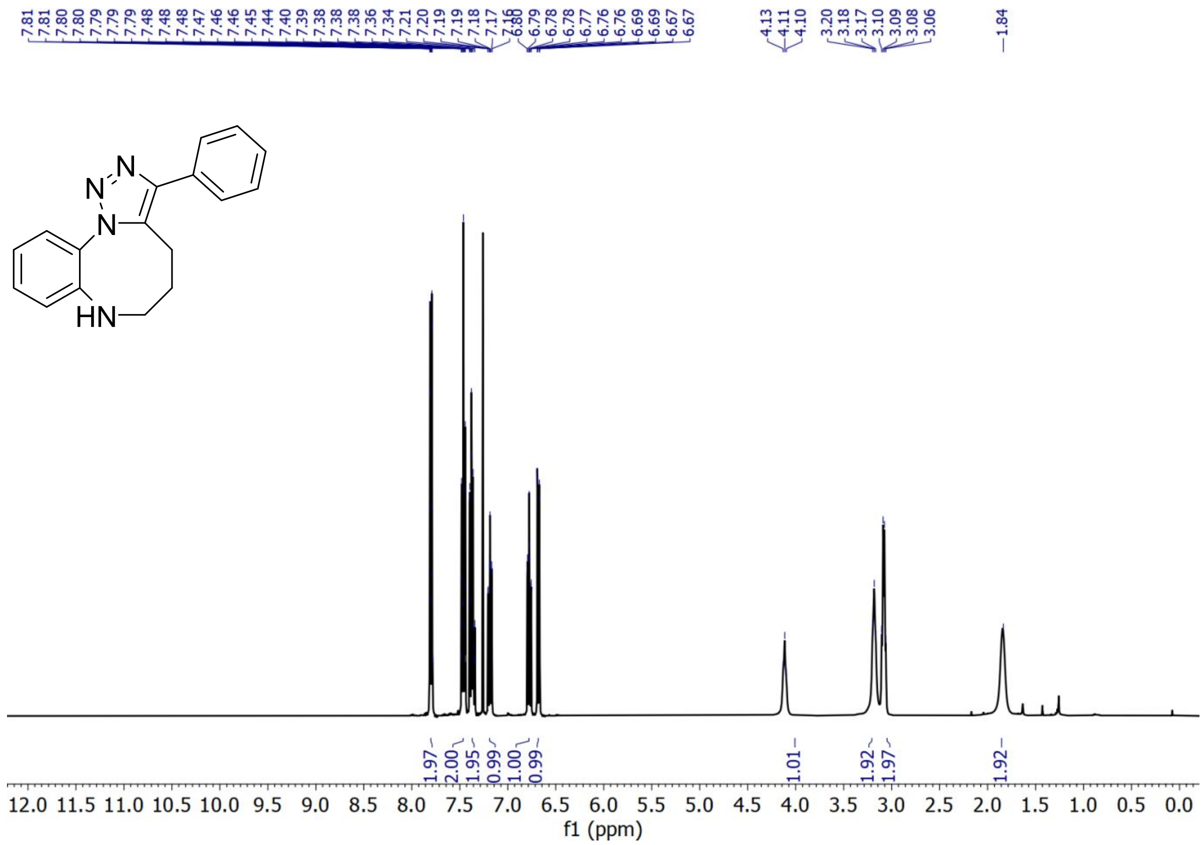

^{1}H NMR spectrum of **19** (400 MHz, $CDCl_3$)

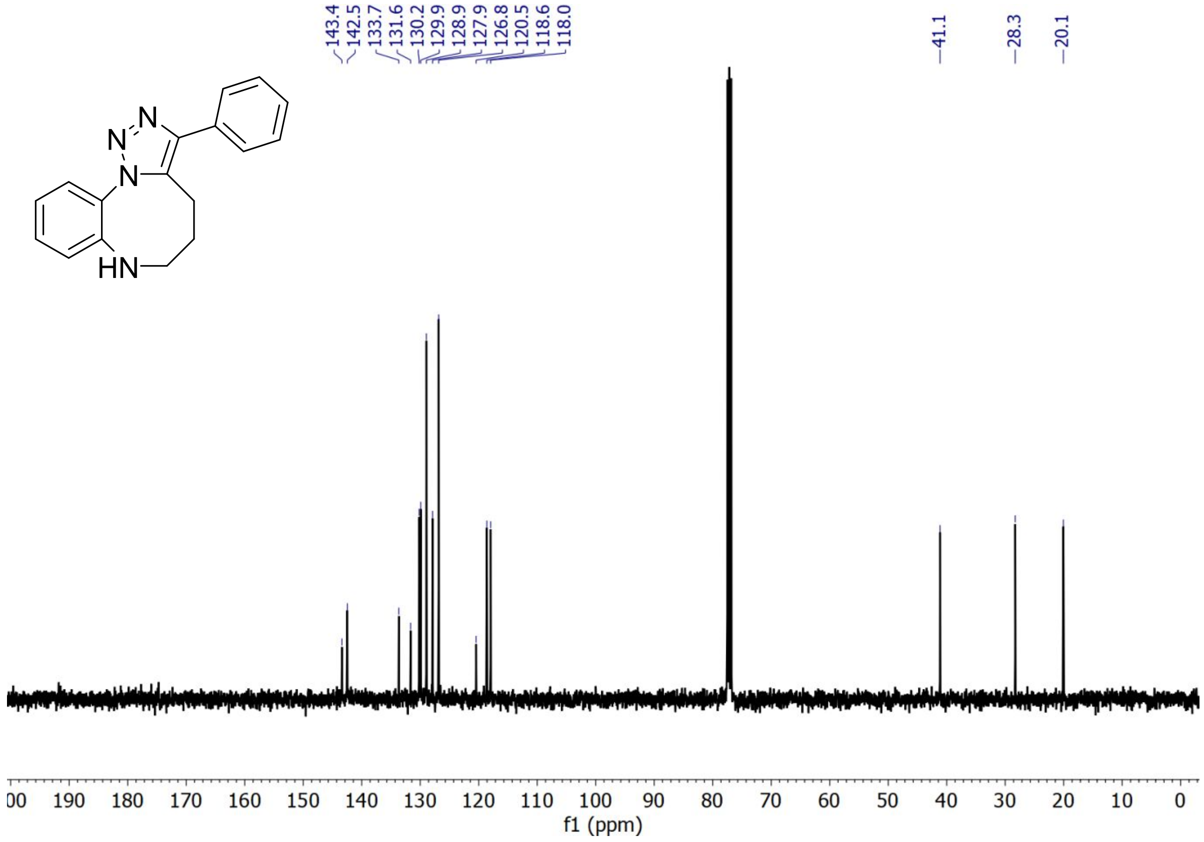

^{13}C NMR spectrum of **19** (101 MHz, $CDCl_3$)

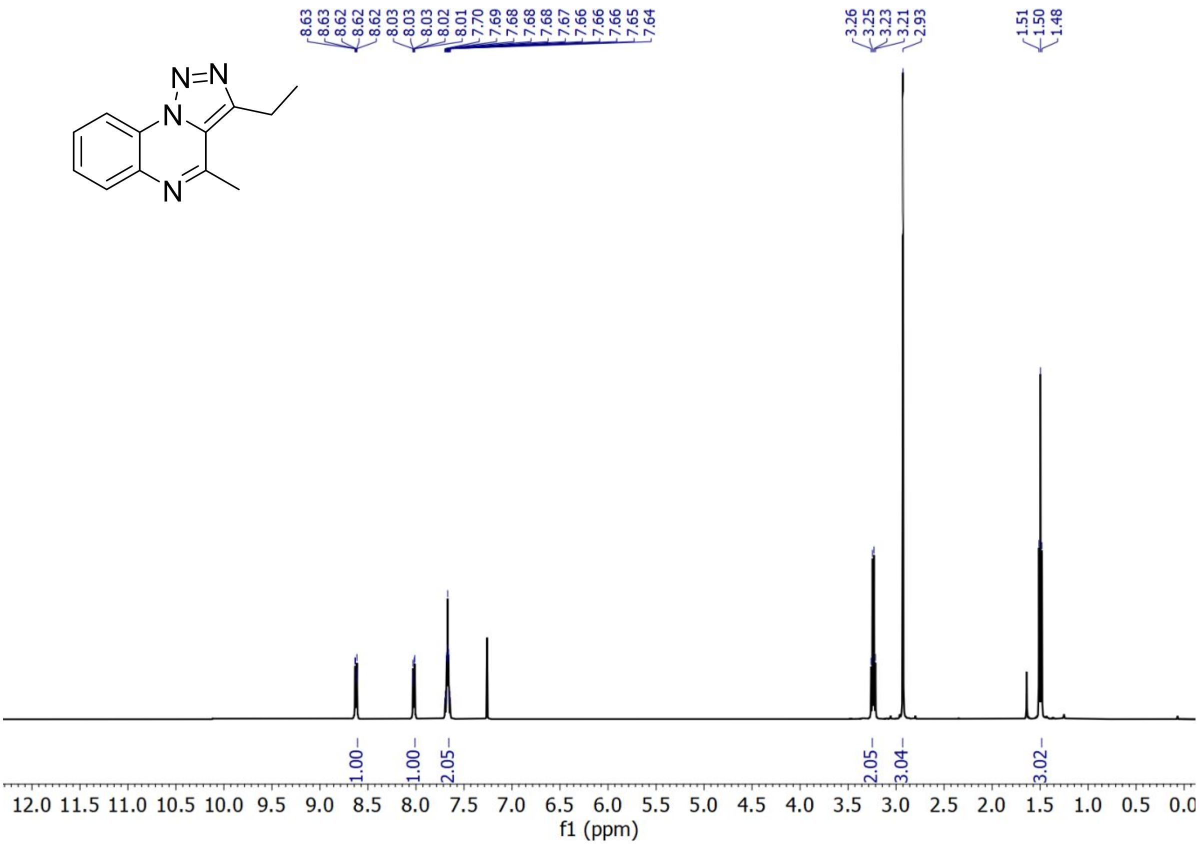

^{1}H NMR spectrum of **17a** (400 MHz, $CDCl_3$)

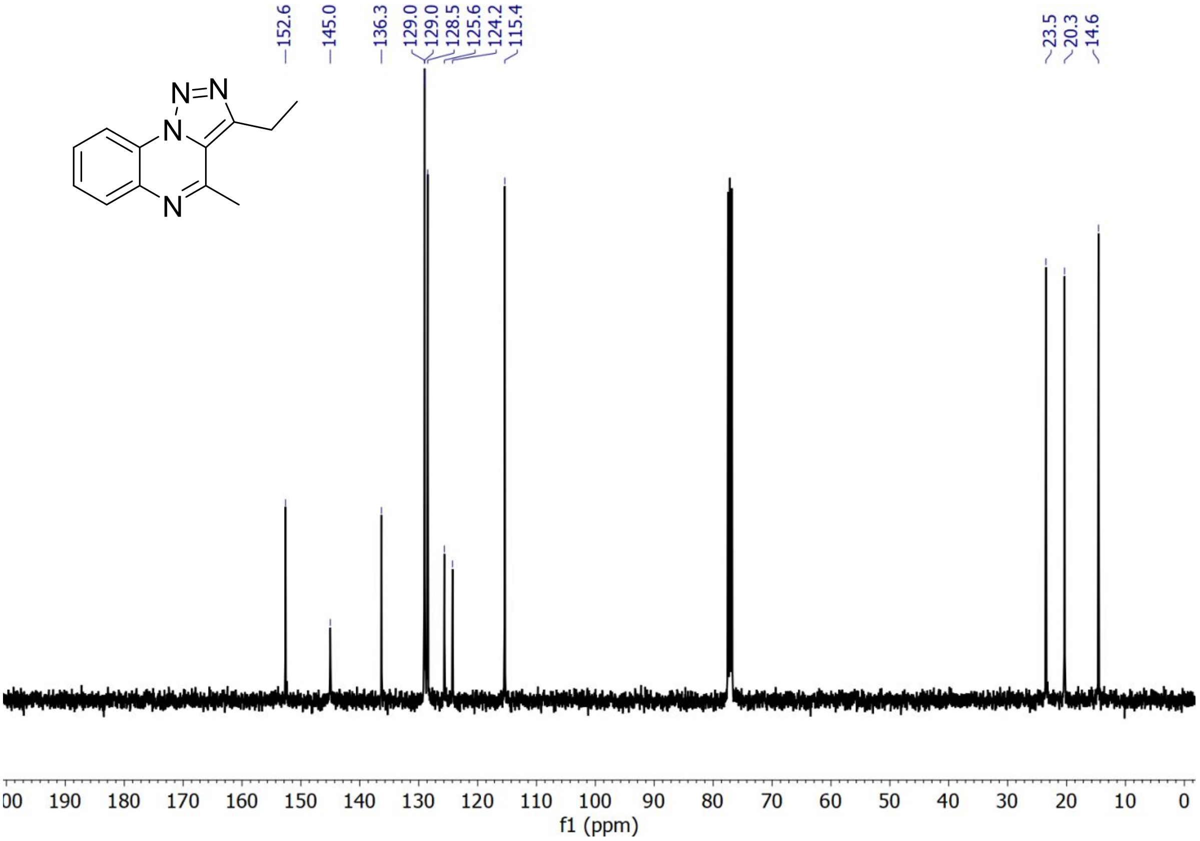

^{13}C NMR spectrum of **17a** (101 MHz, $CDCl_3$)

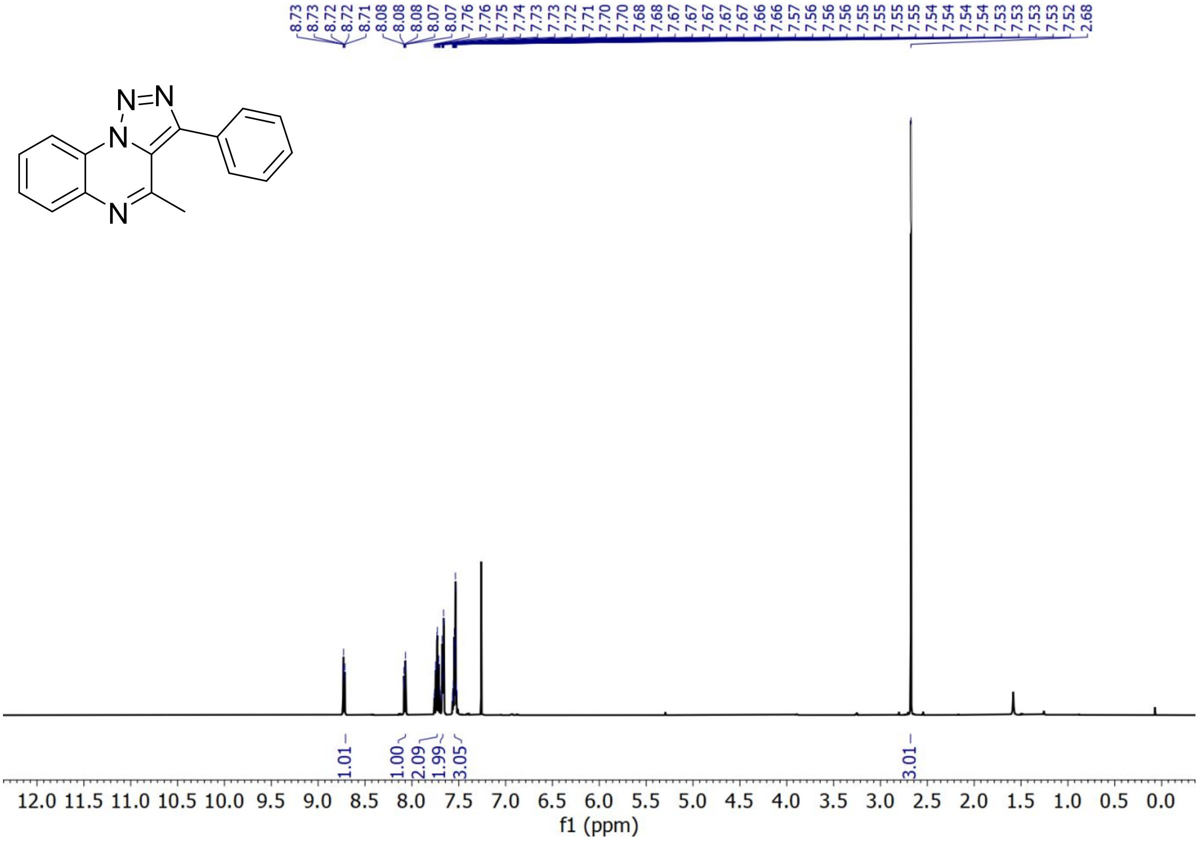

^{1}H NMR spectrum of **17b** (400 MHz, $CDCl_3$)

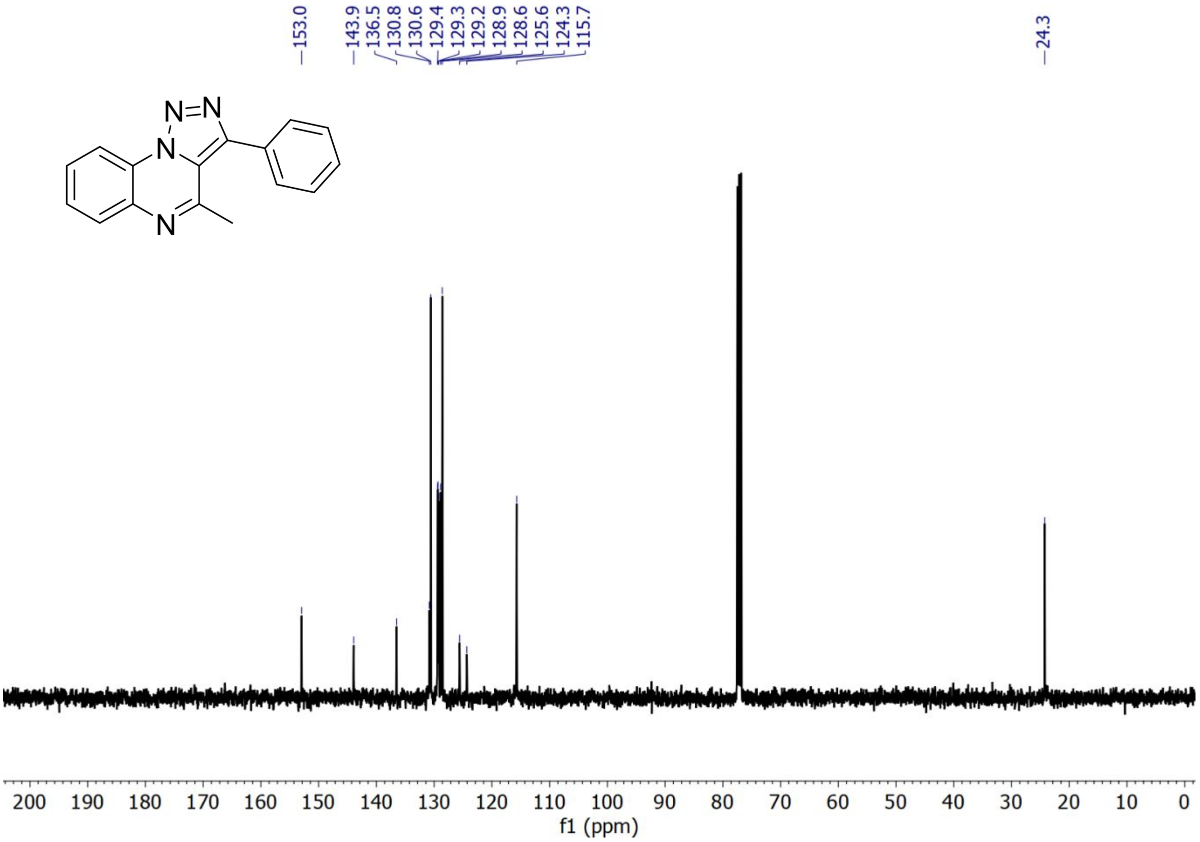

^{13}C NMR spectrum of **17b** (101 MHz, $CDCl_3$)

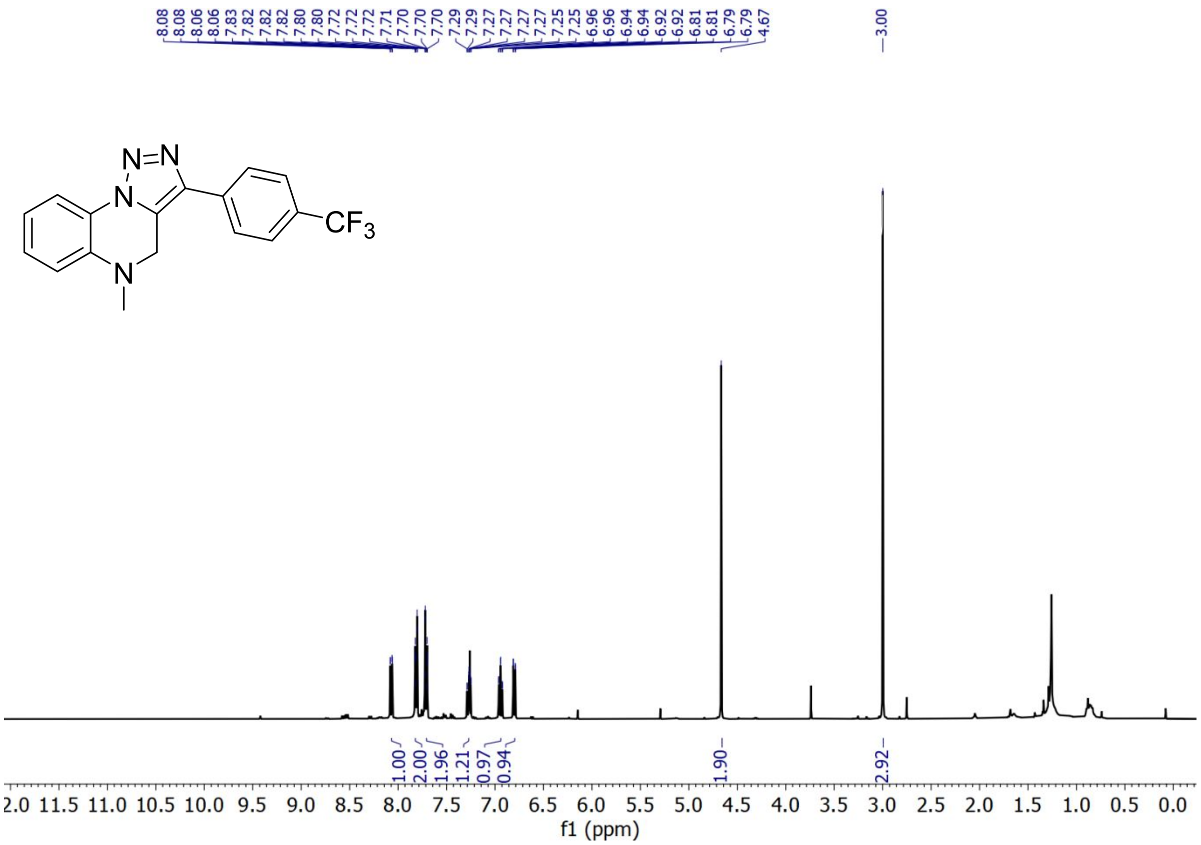

^{1}H NMR spectrum of **20** (400 MHz, $CDCl_3$)

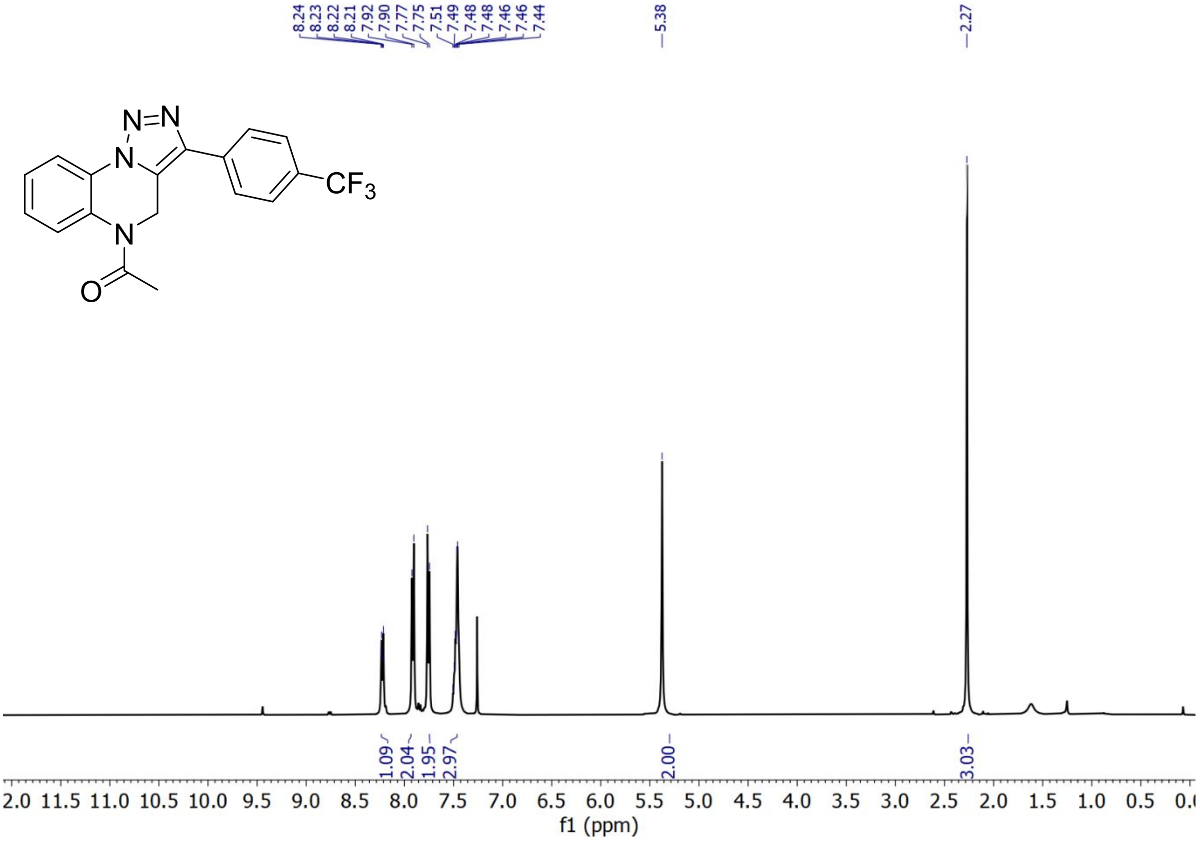

^{1}H NMR spectrum of **21** (400 MHz, $CDCl_3$)

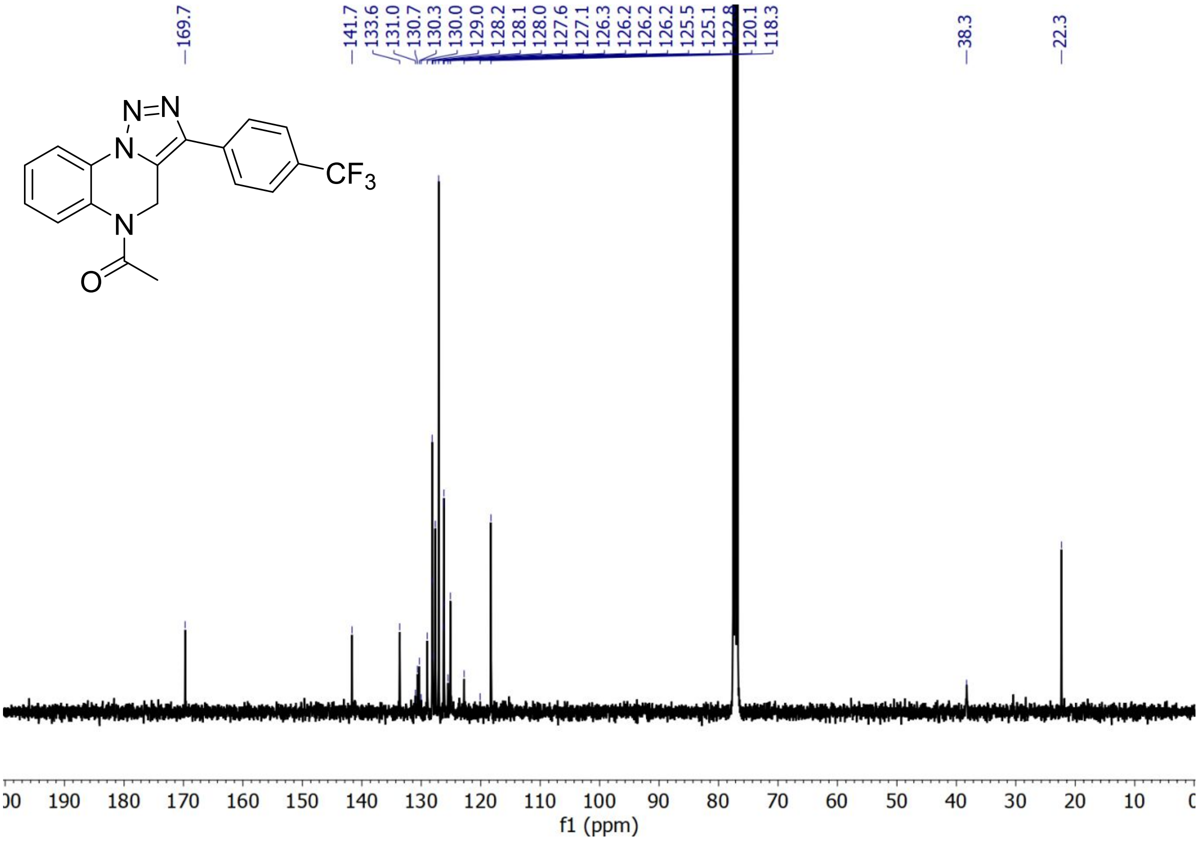

^{13}C NMR spectrum of **21** (101 MHz, $CDCl_3$)

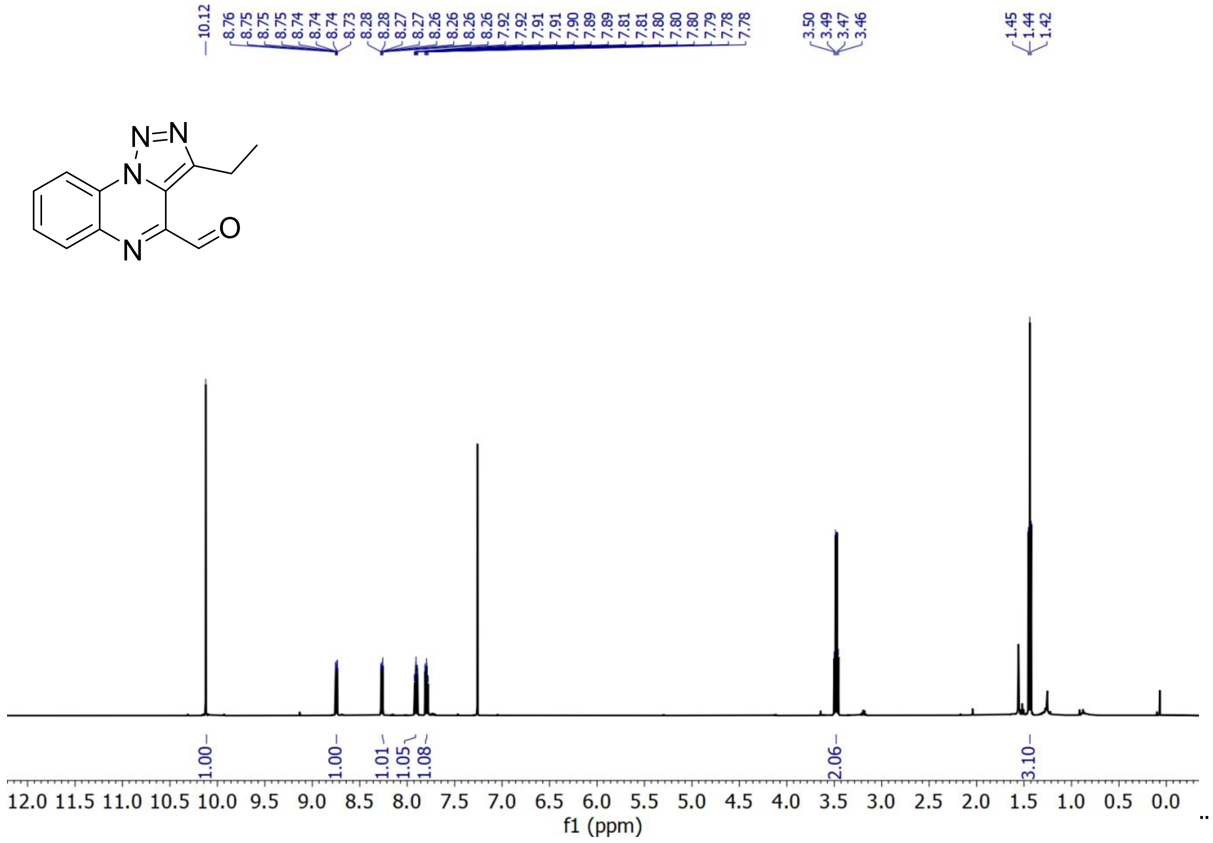

^{1}H NMR spectrum of **22** (400 MHz, $CDCl_3$)

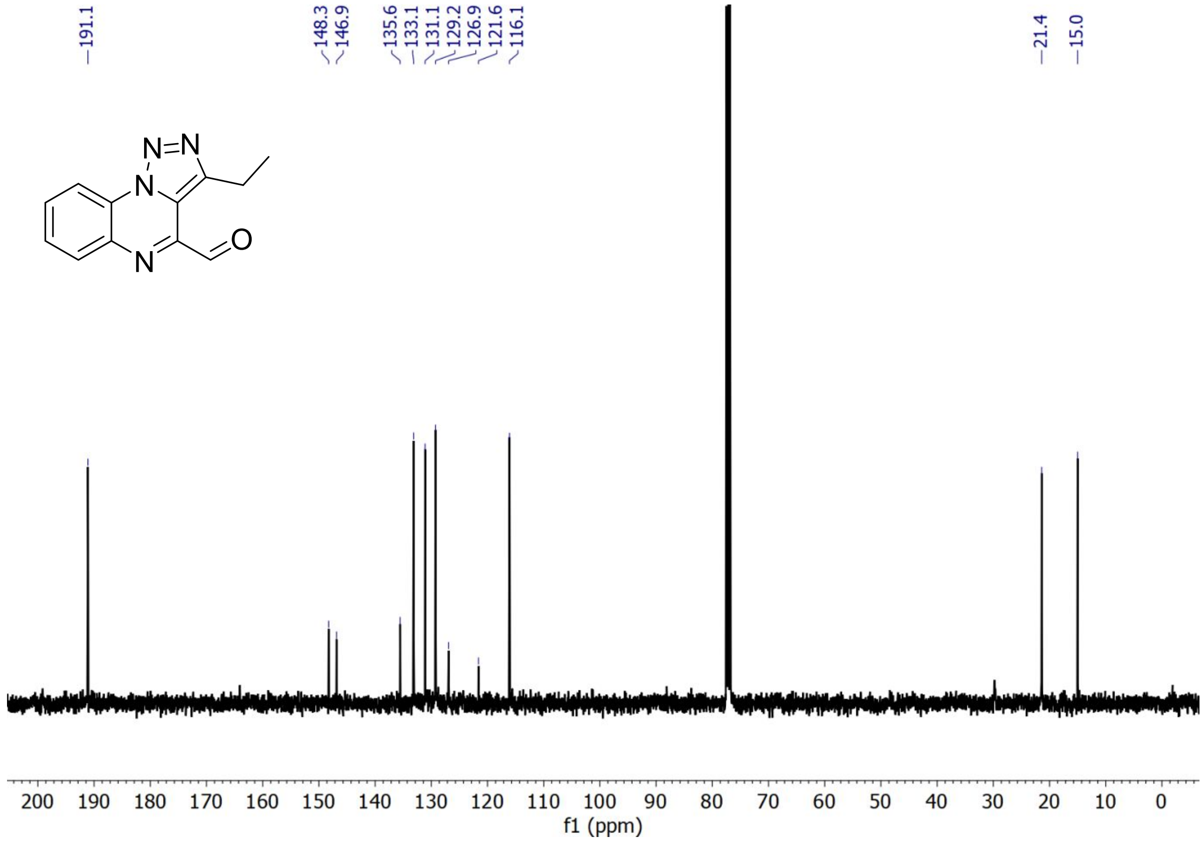

^{13}C NMR spectrum of **22** (101 MHz, $CDCl_3$)

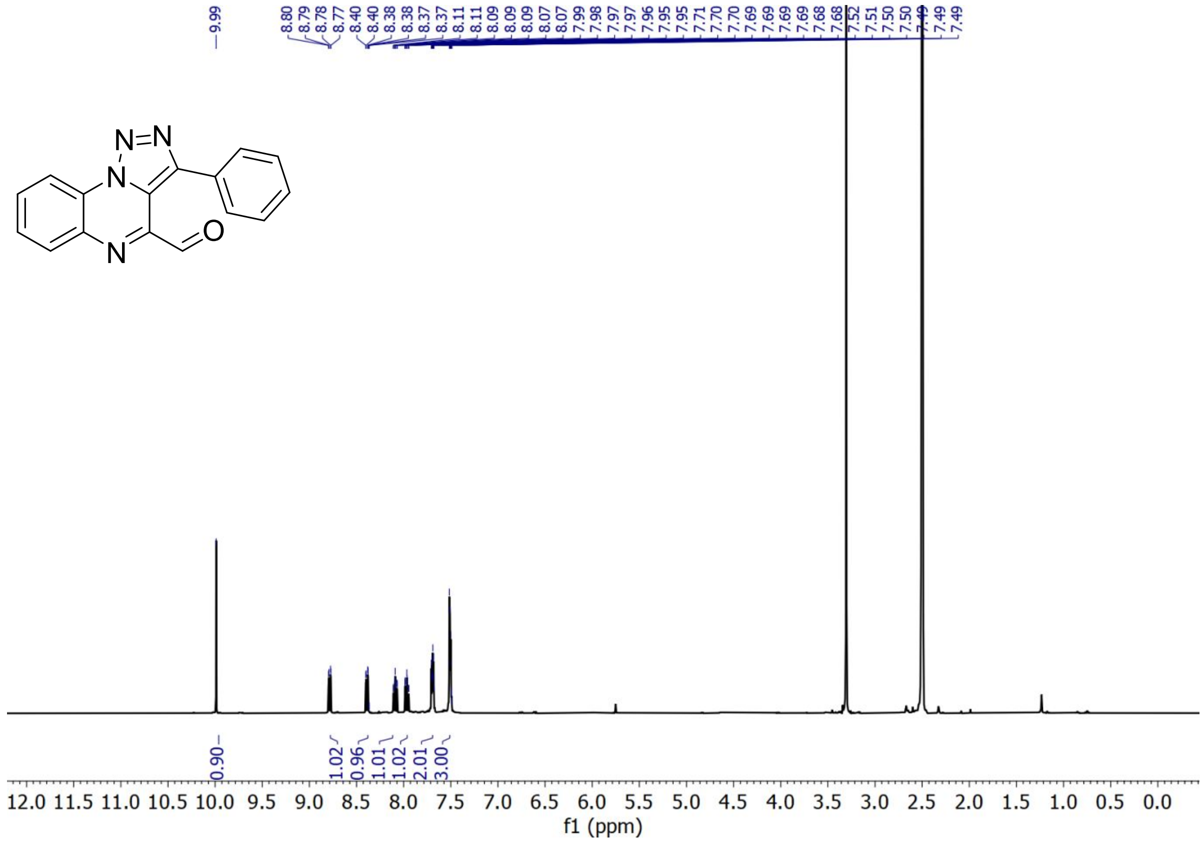

^{1}H NMR spectrum of **22** (400 MHz, DMSO-d_6)

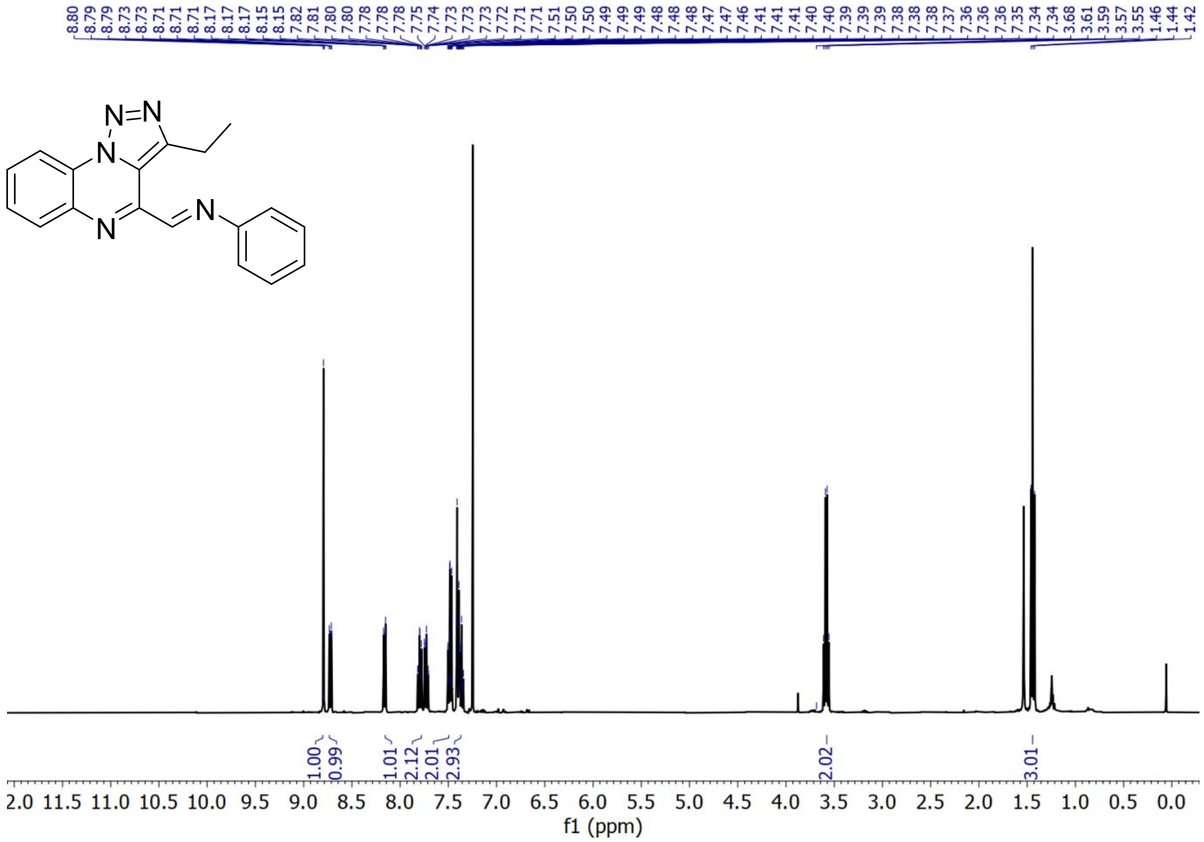

^{1}H NMR spectrum of **24** (400 MHz, $CDCl_3$)

LC-UV-MS traces after heating **15a** with MnO_2 (toluene, 110 ^{o}C) for 6 h

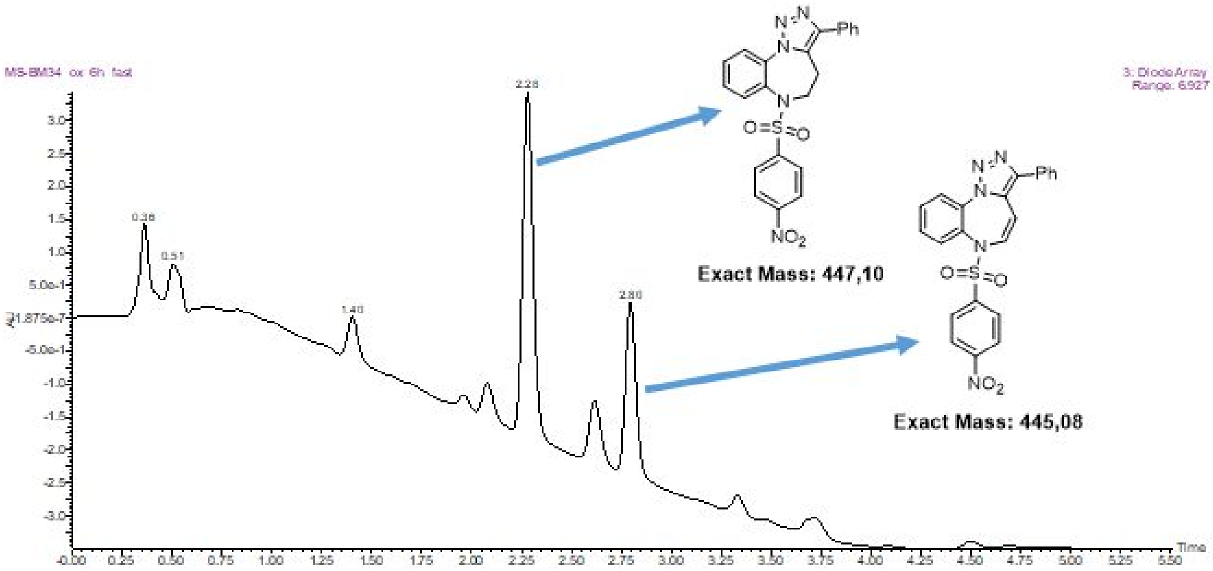

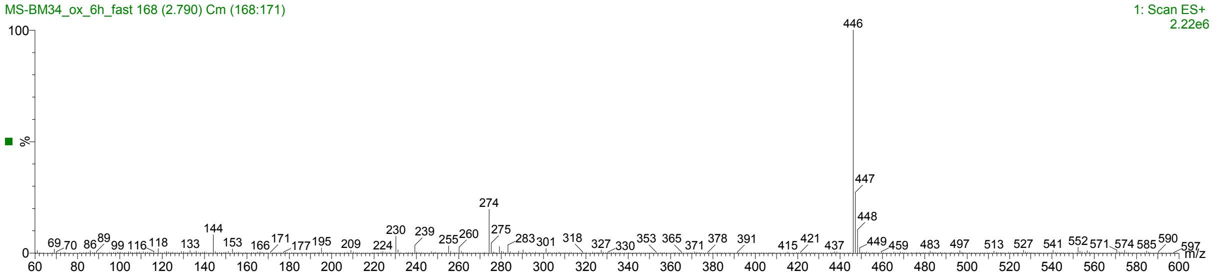

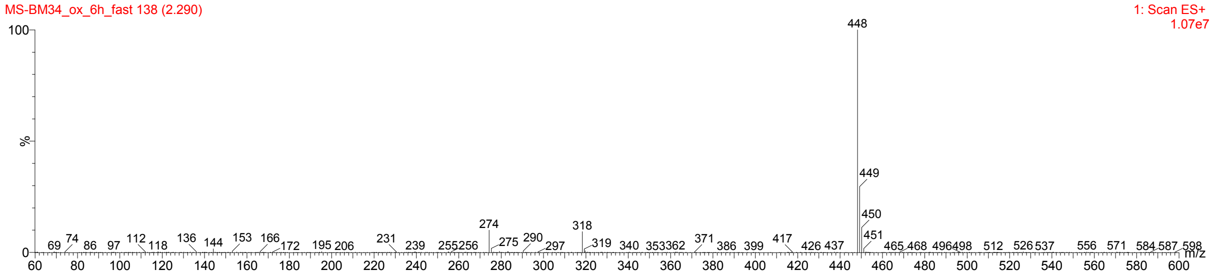

Supplement: Supplementary file 1 — ao4c03979_si_001.pdf [file ao4c03979_si_001.pdf]
